# Visible-Light-Mediated Generation of Nitrogen-Centered Radicals: Metal-Free Hydroimination and Iminohydroxylation Cyclization Reactions

**DOI:** 10.1002/anie.201507641

**Published:** 2015-09-28

**Authors:** Jacob Davies, Samuel G Booth, Stephanie Essafi, Robert A W Dryfe, Daniele Leonori

**Affiliations:** School of Chemistry, University of Manchester Oxford Road, Manchester, M13 9PL (UK) E-mail: daniele.leonori@manchester.ac.uk; School of Chemistry, University of Bristol Cantock's Close, Bristol, BS8 1TS (UK)

**Keywords:** electron transfer, hydroimination, iminohydroxylation, photoredox catalysis, visible light

## Abstract

The formation and use of iminyl radicals in novel and divergent hydroimination and iminohydroxylation cyclization reactions has been accomplished through the design of a new class of reactive *O*-aryl oximes. Owing to their low reduction potentials, the inexpensive organic dye eosin Y could be used as the photocatalyst of the organocatalytic hydroimination reaction. Furthermore, reaction conditions for a unique iminohydroxylation were identified; visible-light-mediated electron transfer from novel electron donor–acceptor complexes of the oximes and Et_3_N was proposed as a key step of this process.

Nitrogen-centered radicals (NCRs) are a versatile class of intermediates that have wide applications in the synthesis of N-containing molecules (Scheme 1 A).[1] However, the difficulties associated with their generation have significantly thwarted their use in synthetic chemistry. In fact, established methods often rely on the homolysis of difficult-to-construct N–X bonds and require the use of toxic and hazardous reagents at elevated temperatures.[1a, 2] The development of a mild, selective, and general method to catalytically generate NCRs from readily available precursors would enable the facile construction of many N-heterocycles, which are privileged motifs in natural products and therapeutic agents.[3]

**Scheme 1:**
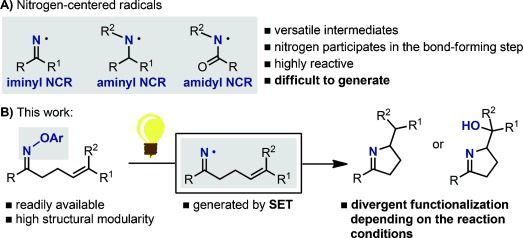
Nitrogen-centered radicals and divergent functionalization processes developed in this work.

Photoredox catalysis has emerged as a powerful technique through which single electron transfer (SET) reactions can be performed under mild conditions.[4] MacMillan[5] and co-workers have developed an asymmetric visible-light-mediated amination of aldehydes by enamine catalysis, and the groups of Sanford,[6] Lee,[7] Yu,[8] and Luo[9] have reported the photoredox generation of phthalimidyl and saccharyl radicals and their use in Minisci-type reactions. The groups of Zheng[10] and Knowles[11] have developed a method for the photoredox generation of diaryl and aryl alkyl aminium radical cations and employed them in C–N bond-forming reactions.

Drawing inspiration from the work of Forrester,[12] Narasaka,[13] and Walton,[14] we speculated that appropriately functionalized *O*-aryl oximes could serve as general, bench-stable NCR precursors that could deliver iminyl radicals upon photoredox activation under mild conditions.[15] Such an approach would clearly benefit from the facile synthesis of aryl oximes, and we hoped that the high structural modularity of the *O*-aryl hydroxylamines would allow us to identify substrates that do not require the use of transition-metal-based photocatalysts.[16] Herein, we describe the successful implementation of this approach and the development of novel, transition-metal-free, visible-light-mediated hydroimination and iminohydroxylation cyclization reactions (Scheme 1 B).

The guiding principle of our photoredox NCR synthesis capitalized on the evidence that electron-poor aromatic compounds have reduction potentials compatible with SET reduction by visible-light-excited photocatalysts,[17] as shown by MacMillan and co-workers.[18] Our envisaged photoredox iminyl NCR generation was initiated by the visible-light-promoted excitation of a photocatalyst (PC→*PC)[19] followed by SET reduction of the aryl unit of oxime **A** to give radical anion **B** (Scheme 2 A). A fragmentation leading to phenoxide **C** and the desired NCR **D** was anticipated to occur next owing to the low bond dissociation energy of the N–O bond.[20] At this stage, we decided to test the viability of this activation mode by combining it with an intramolecular cyclization to synthesize valuable five-membered N-heterocycles.[21] After *5-exo-*trig cyclization, the C-centered radical **E** was expected to abstract a H atom from 1,4-cyclohexadiene (CHD)[20b] to give the desired product **F** and radical **G**, which regenerates the photocatalyst by SET, closing the catalytic cycle. As *5-exo-*trig cyclizations occur rapidly (*k*_c_≈9×10^3^ s^−1^ at RT),[22] we expected that the reaction yields would correlate with the efficiency of the photoredox system.

**Scheme 2:**
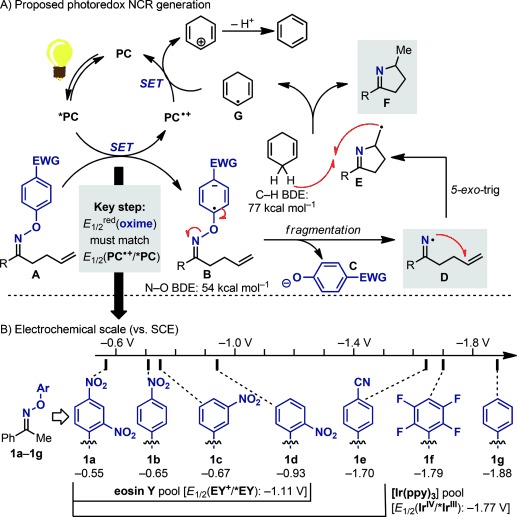
Proposed photoredox cycle and electrochemical studies. EY=eosin Y, ppy=2-phenylpyridine.

The SET between the visible-light-excited photocatalyst and the aryl oxime became a focal point. As the reduction potentials of many photocatalysts are known,[4a] we started our investigations by evaluating the redox profiles of various aryl oximes with the goal of identifying the most suitable/active substrates. Analysis of oximes **1 a**–**1 g** by cyclic voltammetry revealed irreversible reduction profiles that are in accordance with the expected fragmentation process. According to our electrochemical scale (Scheme 2 B), almost all of the examined oximes are expected to undergo SET reduction by *Ir^III^; whereas only the nitro-substituted substrates **1 a**–**1 d** have *E*_1/2_^red^ potentials suitable for SET with the excited state of the organic dye eosin Y.[23]

Based on these results, we selected oximes **2 a**–**2 c** as representative substrates for the evaluation of the proposed radical cyclization reaction (Table 1). To our delight, visible-light irradiation of **2 a**–**2 c** in the presence of [Ir(ppy)_3_], cyclohexadiene, and K_2_CO_3_ gave pyrroline **3 a** in good to excellent yields (entries 2, 4, and 6). As predicted by the electrochemical studies, **2 a** and **2 b** furnished **3 a** when eosin Y was used as the photocatalyst (entries 3 and 5), thus setting the stage for a fully organocatalytic photoredox hydroimination cyclization.

**Table 1:** Optimization of the hydroimination cyclization
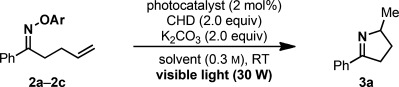

Entry	Substrate^[a]^	Photocatalyst	Solvent	Yield [%]^[b]^
1	**2 a**	[Ir(ppy)_3_]	DMF	81
2	**2 a**	eosin Y	DMF	68
3	**2 a**	eosin Y	acetone	93^[c]^
				
4	**2 b**	[Ir(ppy)_3_]	DMF	53
5	**2 b**	eosin Y	DMF	15
				
6	**2 c**	[Ir(ppy)_3_]	DMF	91
7	**2 c**	eosin Y	DMF	7

[a] **2 a**: Ar=2,4-(NO_2_)_2_C_6_H_3_; **2 b**: Ar=4-(NO_2_)C_6_H_4_; **2 a**: Ar= 4-(CN)C_6_H_4_. [b] Determined by ^1^H NMR analysis; see the Supporting Information for control experiments. [c] 2,4-Dinitrophenol (**8**-H) was also isolated; see the Supporting Information.

The substrate scope was evaluated with a focus on 2,4-dinitro-substituted aryl oximes owing to four favorable aspects: 1) The required hydroxylamine is commercially available, 2) these oximes are typically purified by crystallization, 3) their photoredox reactions do not require a transition-metal catalyst, and 4) the products can be purified by a simple acid–base wash (no chromatographic purification needed on the way from the ketone to the final product).

A broad range of oximes with diverse electronic and steric properties participated efficiently in the visible-light-promoted process (Scheme 3). Bicyclic heterocycles were also obtained in good yields as well as products arising from the cyclization onto di- and trisubstituted olefins.

**Scheme 3:**
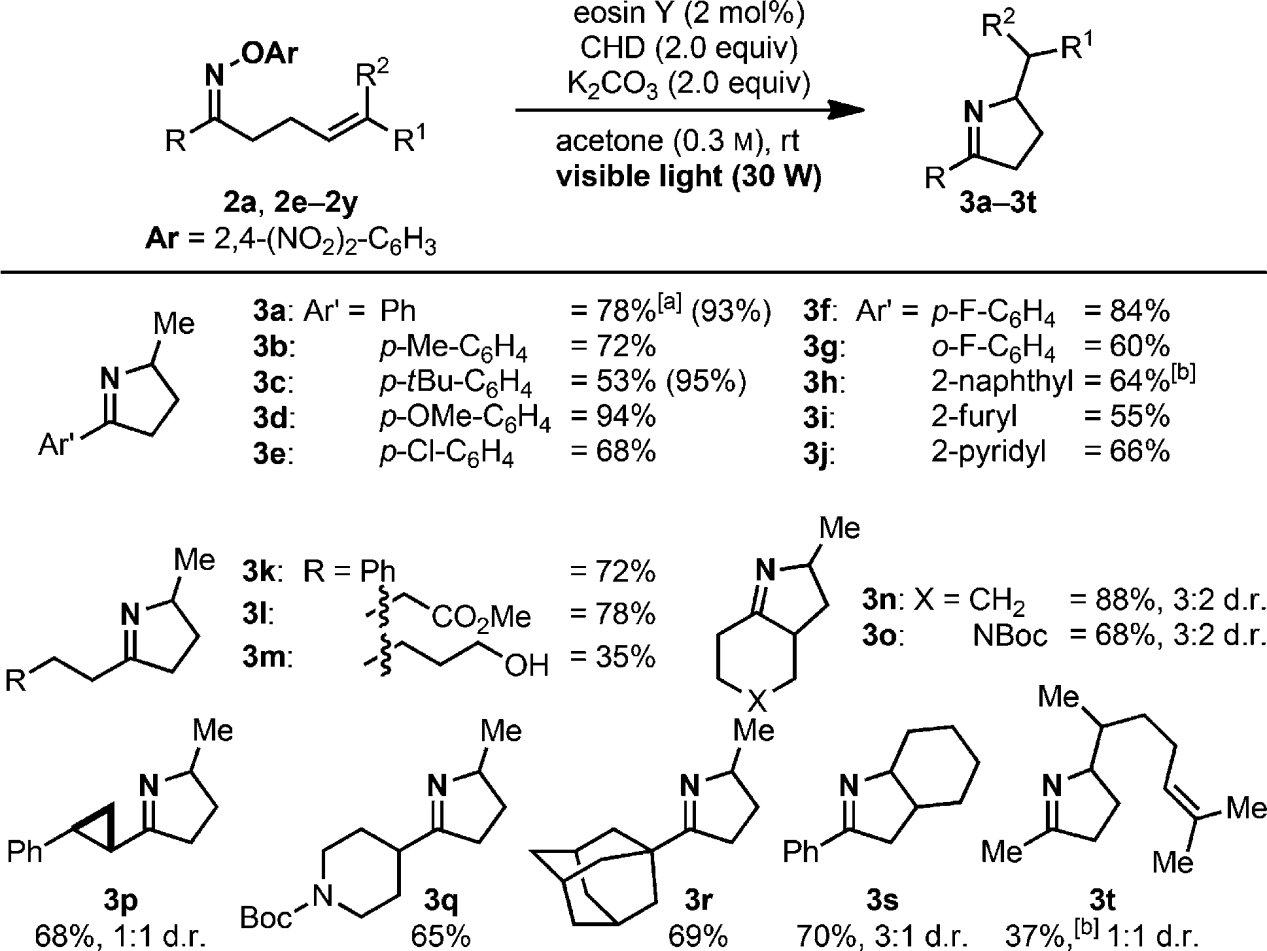
Reaction scope. Yields of isolated products after acid–base wash are given. Yields determined by NMR spectroscopy are given in parentheses. [a] 1 mmol scale. [b] Reaction run in DMF. Boc=*tert*-butyloxycarbonyl.

Intrigued by the low reduction potential and LUMO energy[24] of the 2,4-dinitro-substituted aryl oxime **1 a**, and inspired by the reports of Kochi,[25] Cossy,[26] and Melchiorre[27] on SET, we wondered whether a complementary activation mode could be exploited for the generation of NCRs by visible-light irradiation. As illustrated in Scheme 4 A, we speculated that a simple tertiary amine would be able to reversibly interact with **2 a** to give an electron donor–acceptor complex **H**.[28] Visible-light irradiation should then initiate a SET process to give the radical ion pair **J**.[29] Fragmentation to give **D**, *5*-*exo*-trig cyclization, and H atom abstraction would deliver pyrroline **3 a**. By using the Rehm–Weller equation for electron transfer [Δ*G*_ET_= 0.24 F(*E*_1/2_
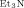
−*E*_1/2_^**2a**^)−Δ*E*_excit_+Δ*E*_coul_],[30] the process was calculated to be exergonic (Δ*G*≈−30 kcal mol^−1^), which indicates a very favorable SET. UV/Vis spectroscopy data further corroborated this proposal. When a CH_3_CN[31] solution of **2 a** was treated with Et_3_N, a bathochromic shift was observed, which indicates the formation of a donor–acceptor complex (Scheme 4 B). The formation of such complexes has not been studied extensively, prompting us to evaluate the strength of this key interaction. By using Job’s method, the **2 a**/Et_3_N stoichiometry in the complex was confirmed to be 1:1, and titration experiments gave an association constant of *K*≈22 m^−1^ (Scheme 4 B). TD-DFT calculations [CAM-B3LYP/6-311++G(d,p) in CH_3_CN] confirmed that absorption at approximately 440 nm is due to a transition from the nitrogen lone pair to the π* orbital of the aromatic unit of the oxime.[24] Exposure of **2 b** and **2 c** to Et_3_N (up to 10 equiv) did not lead to significant bathochromic shifts, which suggests that there is limited or no donor–acceptor complex formation.[24]

**Scheme 4:**
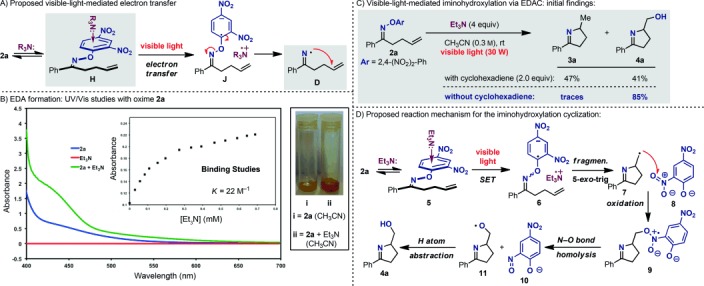
A) Proposed visible-light-mediated electron transfer via an electron donor–acceptor complex for the hydroimination of *O*-aryl oximes. B) UV/Vis studies. C) Initial findings. D) Proposed reaction mechanism for the iminohydroxylation cyclization.

Encouraged by the UV/Vis studies, we decided to evaluate the ability of **2 a** to undergo the proposed visible-light- and Et_3_N-mediated SET process. Irradiation of a solution of **2 a**, Et_3_N, and cyclohexadiene in CH_3_CN furnished the desired product **3 a** (47 %) together with iminoalcohol **4 a** (41 %; Scheme 4 C). The unforeseen formation of **4 a** opened the way to the development of the first visible-light-mediated iminohydroxylation cyclization reaction. By simply excluding cyclohexadiene from the reaction mixture, the yield of **4 a** was increased to 85 %. Other amines were evaluated, and they also selectively provided **4 a**, albeit in lower yields. As suggested by the UV/Vis studies, substrate **2 b** gave the desired product in low yield whereas **2 c** did not react.[24]

The formation of **4 a** raised additional questions about the underlying mechanism and the origin of the oxygen atom in the final product (Scheme 4 D). The involvement of adventitious O_2_ or H_2_O was excluded by running the reaction under rigorously moisture- and oxygen-free conditions.[24] In contrast to the hydroimination cyclization, 2,4-dinitrophenol (**8**-H) was not formed, but we obtained 2-NO-4-NO_2_-C_6_H_3_OH (**10**-H). This observation indicates a unique trifunctional role of the aromatic unit of the *O*-aryl oximes, which sequentially serves as a sensitizer, an electron acceptor, and an oxidant. Initial rate kinetics revealed the reaction to be first order in **2 a** and to display saturation behavior in Et_3_N (1st order at 0<[Et_3_N]<1 equiv and zero order at [Et_3_N]>1 equiv). Based on these findings, we propose the following mechanism: Fast and reversible binding of Et_3_N and **2 a** gives intermediate **5**, which undergoes SET upon visible-light excitation to give the dipolar species **6**. Fragmentation and 5-*exo-*trig cyclization give the C-centered radical **7** and the stable phenoxide **8** (p*K*_a_≈4). Subsequent oxidation by attack of the radical onto the NO_2_ group[32] leads to **9**, and successive N–O bond homolysis furnishes **10** and the O-centered radical **11**, which undergoes a fast hydrogen atom abstraction.[24]

With this very simple optimized procedure in hand, the scope of the iminohydroxylation was evaluated with the aryl oximes **2 a** and **2 e**–**2 y**. All examined substrates reacted well and provided the desired iminoalcohols **4 a**–**4 u** in good to high yields (Scheme 5). Bicyclic products could be obtained, and substrates containing di- and trisubstituted olefins also reacted well, giving access to products containing up to three contiguous stereogenic centers.

**Scheme 5:**
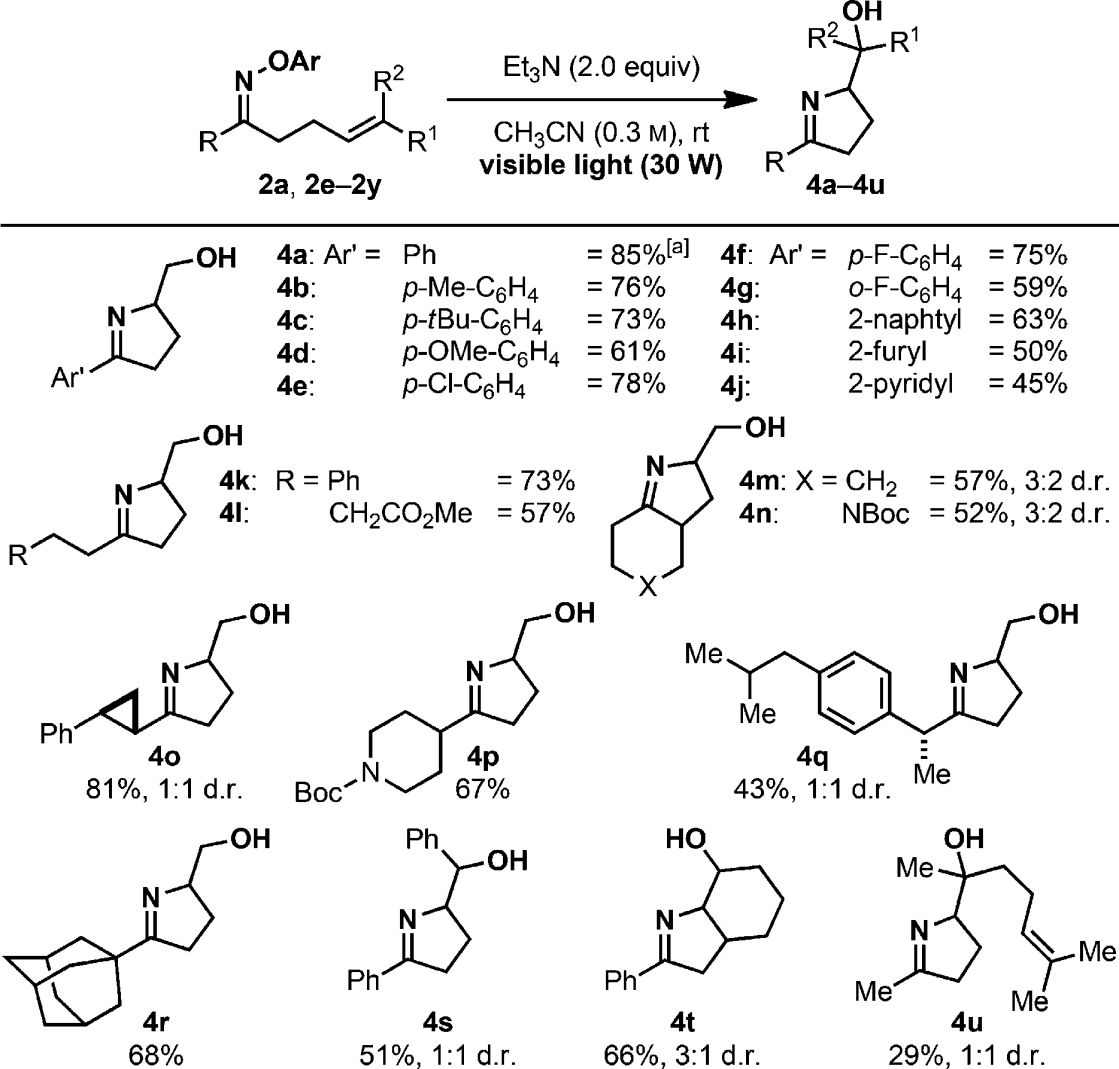
Scope of the iminohydroxylation cyclization reaction. [a] 2 mmol scale.

In conclusion, we have developed a divergent strategy for the hydroimination and iminohydroxylation cyclization of unactivated olefins. Electrochemical studies facilitated the identification of a very reactive class of *O*-aryl oximes that obviate the need for a transition-metal photocatalyst and undergo organocatalytic hydroimination cyclizations. The unprecedented ability of the aryl unit to sequentially act as a sensitizer, electron acceptor, and oxidant enabled the development of a unique Et_3_N- and visible-light-mediated iminohydroxylation cyclization. Future studies will focus on applying this method to other nitrogen-centered radicals and on developing asymmetric variants of the hydroimination and iminohydroxylation cyclizations.

## Supporting Information

As a service to our authors and readers, this journal provides supporting information supplied by the authors. Such materials are peer reviewed and may be re-organized for online delivery, but are not copy-edited or typeset. Technical support issues arising from supporting information (other than missing files) should be addressed to the authors.

miscellaneous_information

## References

[1a] Zard SZ (2008). Chem. Soc. Rev.

[1b] Cassayre J, Gagosz F, Zard SS (2002). Angew. Chem. Int. Ed.

[b01] (2002). Angew. Chem.

[1c] Sharp L, Zard SZ (2006). Org. Lett.

[2a] Faulkner A, Race NJ, Scott JS, Bower JF (2014). Chem. Sci.

[2b] Bingham M, Moutrille C, Zard SS (2014). Heterocycles.

[2c] Kitamura M, Shintaku Y, Kudo D, Okauchi T (2010). Tetrahedron Lett.

[2d] Minozzi M, Nanni D, Spagnolo P (2009). Chem. Eur. J.

[2e] Beaume A, Courillon C, Derat E, Malacria M (2008). Chem. Eur. J.

[2f] Noack M, Göttlich R (2002). Chem. Commun.

[2g] Guindon Y, Guerin B, Landry SR (2001). Org. Lett.

[2h] Lin X, Artman GD, Stien D, Weinreb SM (2001). Tetrahedron.

[3] Welsch ME, Snyder SA, Stockwell BR (2010). Curr. Opin. Chem. Biol.

[4a] Prier CK, Rankic DA, MacMillan DWC (2013). Chem. Rev.

[4b] Xi Y, Yi H, Lei A (2013). Org. Biomol. Chem.

[4c] Hopkinson MN, Sahoo B, Li J-L, Glorius F (2014). Chem. Eur. J.

[4d] Narayanam JMR, Stephenson C (2011). Chem. Soc. Rev.

[5] Cecere G, König CM, Alleva JL, MacMillan DWC (2013). J. Am. Chem. Soc.

[6] Allen LJ, Cabrera PJ, Lee M, Sanford MS (2014). J. Am. Chem. Soc.

[7] Kim H, Kim T, Lee DG, Roh SW, Lee C (2014). Chem. Commun.

[8] Qin Q, Yu S (2014). Org. Lett.

[9] Song L, Zhang L, Luo S, Cheng J-P (2014). Chem. Eur. J.

[10a] Maity S, Zheng N (2012). Angew. Chem. Int. Ed.

[b02] (2012). Angew. Chem.

[10b] 10.1002/anie.201504076.

[11] Musacchio AJ, Nguyen LQ, Beard H, Knowles RR (2014). J. Am. Chem. Soc.

[12a] Atmaram S, Forrester AR, Gill M, Thomson RH (1981). J. Chem. Soc. Perkin Trans. 1.

[12b] Forrester AR, Gill M, Sadd JS, Thomson RH (1979). J. Chem. Soc. Perkin Trans. 1.

[13a] Mikami KNT (2000). Chem. Lett.

[13b] Kitamura M, Narasaka K (2008). Bull. Chem. Soc. Jpn.

[14a] McBurney RT, Walton JC (2013). J. Am. Chem. Soc.

[14b] Walton JC (2014). Acc. Chem. Res.

[15] Jiang H, An X, Tong K, Zheng T, Zhang Y, Yu S (2015). Angew. Chem. Int. Ed.

[b03] (2015). Angew. Chem.

[16a] Ravelli D, Fagnoni M, Albini A (2013). Chem. Soc. Rev.

[16b] Brimioulle R, Lenhart D, Maturi MM, Bach T (2015). Angew. Chem. Int. Ed.

[b04] (2015). Angew. Chem.

[16c] Wilger DJ, Grandjean J-MM, Lammert TR, Nicewicz DA (2014). Nat. Chem.

[16d] Hari DP, Schroll P, König B (2012). J. Am. Chem. Soc.

[16e] Neumann M, Fuldner S, König B, Zeitler K (2011). Angew. Chem. Int. Ed.

[b05] (2011). Angew. Chem.

[16f] Bauer A, Westkmäper F, Grimme S, Bach T (2005). Nature.

[16g] Brimioulle R, Bach T (2013). Science.

[17] Wakasa M, Sakaguchi Y, Nakamura J, Hayashi H (1992). J. Phys. Chem.

[18a] Terrett JA, Clift MD, MacMillan DWC (2014). J. Am. Chem. Soc.

[18b] Zuo Z, MacMillan DWC (2014). J. Am. Chem. Soc.

[18c] McNally A, Prier CK, MacMillan DWC (2011). Science.

[18d] Pirnot MT, Rankic DA, Martin DBC, MacMillan DWC (2013). Science.

[19] Vogler A, Kunkely H (2000). Coord. Chem. Rev.

[20a] Lorance ED, Kramer WH, Gould IR (2002). J. Am. Chem. Soc.

[20b] Luo Y-R (2003). Handbook of Bond Dissociation Energies in Organic Compounds.

[21] Boivin JB, Fouquet E, Zard SZ (1994). Tetrahedron.

[22] Agabito F, Nunes PM, Cabral BJCosta, Borges dos Santos RM, Simões JAMartinho (2007). J. Org. Chem.

[23] Hari DP, König B (2014). Chem. Commun.

[25a] Rosokha SV, Kochi JK (2008). Acc. Chem. Res.

[25b] Rathore R, Lindeman SV, Kochi JK (1997). J. Am. Chem. Soc.

[26] Cossy J, Belotti D (2006). Tetrahedron.

[27a] Kandukuri SR, Bahamonde A, Chatterjee I, Jurberg ID, Escudero-Adan EC, Melchiorre P (2015). Angew. Chem. Int. Ed.

[b06] (2015). Angew. Chem.

[27b] Arceo E, Jurberg ID, Alvarez-Fernadez A, Melchiorre P (2013). Nat. Chem.

[27c] Nappi M, Bergonzini G, Melchiorre P (2014). Angew. Chem. Int. Ed.

[b07] (2014). Angew. Chem.

[28a] Kavarnos GJ, Turro NJ (1986). Chem. Rev.

[28b] Mattay J (1987). Angew. Chem. Int. Ed. Engl.

[b08] (1987). Angew. Chem.

[28c] Berionni G, Bertelle P-A, Marrot J, Goumont R (2009). J. Am. Chem. Soc.

[28d] Nad S, Pal H (2000). J. Phys. Chem. A.

[29] Costentin C, Robert M, Saveant J-M (2006). Chem. Phys.

[30] Farid S, Dinnocenzo JP, Merkel PB, Young RH, Shukla D, Guirado G (2011). J. Am. Chem. Soc.

[31a] Turro NJ, Engel R (1969). J. Am. Chem. Soc.

[31b] Abe T, Kawai A, Kaji Y, Shibuya K, Obi K (1999). J. Phys. Chem. A.

[32a] Zhang Y, Du Y, Huang Z, Xu J, Wu X, Wang Y, Wang M, Yang S, Webster RD, Chi YR (2015). J. Am. Chem. Soc.

[32b] White NA, Rovis T (2014). J. Am. Chem. Soc.

