# Supplementary material for: Visible-Light-Mediated Generation of Nitrogen-Centered Radicals: Metal-Free Hydroimination and Iminohydroxylation Cyclization Reactions
Source: Angew Chem Int Ed Engl. 2015 Sep 28;54(47):14017–21. doi: 10.1002/anie.201507641 (PMC4648045; doi:10.1002/anie.201507641)
Supplement: Supplementary file 1 — miscellaneous_information [file anie0054-14017-sd1.pdf]

Angewandte Chemie
Eine Zeitschrift der Gesellschaft Deutscher Chemiker

Supporting Information

Visible-Light-Mediated Generation of Nitrogen-Centered Radicals: Metal-Free Hydroimination and Iminohydroxylation Cyclization Reactions

*Jacob Davies, Samuel G. Booth, Stephanie Essafi, Robert A. W. Dryfe, and Daniele Leonori**

anie_201507641_sm_miscellaneous_information.pdf

Table of Contents

1 General Experimental Details 2

2 Starting Material Synthesis 3
2.1 Synthesis of *O*-aryloximes..3

3 Electrochemical studies 35
3.1 General Experimental Detail 35
3.2 Electrochemical potentials..36
3.3 Cyclic voltammograms 37

4 Hydroimination-cyclization 41
4.1 Reaction optimization with oxime 2a 41
4.2 Mechanistic Studies..46
4.2.1 Proposed Mechanism 46
4.2.2 Emission quenching experiments (Stern-Volmer Studies) 48
4.2.3 Light ON/OFF reaction 49
4.2.4 Reaction with monochromatic LEDs 50
4.3 Substrate scope 51

5 Iminohydroxilation-cyclization 59
5.1 Electron-Donor-Acceptor Complex (EDAC) Formation 59
5.1.1 UV/Vis studies 59
5.1.2 Binding studies..63
5.2 Reaction optimization 67
5.3 Substrate scope 69
5.4 Mechanistic Studies..79
5.4.1 Proposed mechanism..79
5.4.2 Light ON/OFF reaction 81
5.4.3 Kinetic studies 82
5.4.4 Glove-box reaction..84
5.4.5 Reaction with monochromatic LEDs 85

6 Computational Studies 86
6.1 Determination of LUMO energies for oximes 1a–g 86
6.2 Energy minimization for the 1a•Me$_3$N EDAC complex..88
6.2.1 Calculation details..88
6.2.2 Results. 89

7 NMR Spectra..96

8 X-ray crystal structure 163

9 References..166

1 General Experimental Details

All required fine chemicals were used directly without purification unless stated otherwise. All air and moisture sensitive reactions were carried out under nitrogen atmosphere using standard Schlenk manifold technique. THF was distilled from sodium/benzophenone, CH_2Cl_2 and was distilled from CaH_2, CH_3CN was distilled from activated 4Å molecular sieves, Et_3N was distilled over KOH. *O*-(2,4-dinitrophenyl)hydroxylamine was purchased by Fluorochem and used without further purification. ^1H and ^{13}C Nuclear Magnetic Resonance (NMR) spectra were acquired at various field strengths as indicated and were referenced to $CHCl_3$ (7.27 and 77.0 ppm for ^1H and ^{13}C respectively). ^1H NMR coupling constants are reported in Hertz and refer to apparent multiplicities and not true coupling constants. Data are reported as follows: chemical shift, integration, multiplicity (s = singlet, br s = broad singlet, d = doublet, t = triplet, q = quartet, qi = quintet, sx = sextet, sp = septet, m = multiplet, dd = doublet of doublets, etc.), proton assignment (determined by 2D NMR experiments: COSY, HSQC and HMBC) where possible. High-resolution mass spectra were obtained using a JEOL JMS-700 spectrometer or a Fissions VG Trio 2000 quadrupole mass spectrometer. Spectra were obtained using electron impact ionization (EI) and chemical ionization (CI) techniques, or positive electrospray (ES). Infra-red spectra were recorded using a JASCO FT/IR 410 spectrometer or using an ATI Mattson Genesis Seris FTIR spectrometer as evaporated films or liquid films. Analytical TLC: aluminum backed plates pre-coated (0.25 mm) with Merck Silica Gel 60 F254. Compounds were visualized by exposure to UV-light or by dipping the plates in permanganate ($KMnO_4$) stain followed by heating. Flash column chromatography was performed using Merck Silica Gel 60 (40–63 μm). All mixed solvent eluents are reported as v/v solutions. UV/Vis spectra were obtained using an Agilent 6453 spectrometer and 1 mm High Precision Cell made of quartz from Hellma Analytics.

The light bulbs were standard 30 W house-hold bought from ASDA Stores Limited.

The LEDs were bought from LEDLightZone.

Reactions up to 50 mg were run in 50 x 12 mm glass tubes from Fisher. Larger scale reactions were conducted in CEM 10 mL glass microwave tubes (see Section 9 for pictures of the reaction set-up).

2 Starting Material Synthesis

2.1 Synthesis of *O*-aryloximes

General Procedure for the synthesis of *O*-arylhydroxylamines S1–4 – GP1

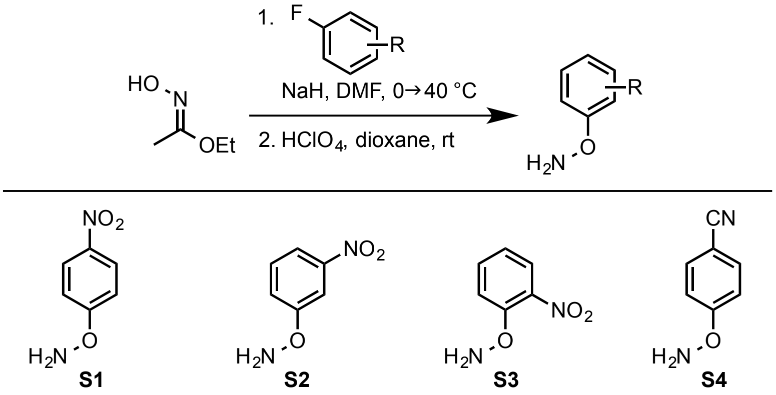

A solution of ethyl *N*-hydroxylacetimidate (1.0 equiv.) in dry DMF (0.4M) under nitrogen was cooled to 0 °C and treated with NaH (1.2 equiv., 60% dispersion in mineral oil). The mixture was allowed to warm to room temperature over 1 h, then the fluorobenzene derivative (1.2 equiv.) was added and the mixture warmed to 40 °C and stirred overnight. The mixture was cooled to room temperature and diluted with H_2O and EtOAc. The layers were separated and the aqueous layer was extracted with EtOAc (x 2). The combined organic layers were dried ($MgSO_4$), filtered and evaporated. Purification by column chromatography on silica gel (petrol–EtOAc 99:1→97:3), gave the corresponding ethyl *N*-aryloxyacetimidate. A solution of ethyl *N*-aryloxyacetimidate (1.0 equiv.) in dioxane (1M) was cooled to 0 °C, treated with $HClO_4$ (10 equiv., 70% in H_2O) and stirred for 4 h while warming to room temperature. The mixture was poured onto cold H_2O and the pH adjusted to 7 with NaOH pellets. The layers were separated and the aqueous layer was extracted EtOAc (x 2). The combined organic layers were dried ($MgSO_4$), filtered and evaporated. Purification by column chromatography on silica gel (CH_2Cl_2:NH_4OH 99.9:0.1), gave the corresponding *O*-arylhydroxylamine.

***O*-(4-Nitrophenyl)hydroxylamine (S1)**

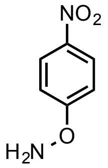

Following **GP1**, 4-fluoro-nitrobenzene (440 mg, 3.12 mmol) gave O-(4-nitrophenyl)hydroxylamine (290 mg, 60%) as an amorphous solid. ^{1}H NMR (400 MHz, $CDCl_3$) δ 7.98 (2H, d, *J* = 9.0 Hz), 7.07 (2H, d, *J* = 9.0 Hz), 6.90 (2H, br s). Data in accordance with literature.[1]

***O*-(3-Nitrophenyl)hydroxylamine (S2)**

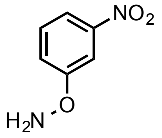

Following **GP1**, 3-fluoronitrobenzene (330 mg, 2.34 mmol) gave O-(3-nitrophenyl)hydroxylamine (140 mg, 75%) as an amorphous solid. ^{1}H NMR (400 MHz, $CDCl_3$) δ 8.08 (1H, s), 7.82–7.76 (1H, m), 7.46–7.35 (2H, m), 6.03 (2H, br s); ^{13}C NMR (101 MHz, $CDCl_3$) δ 162.1, 149.1 129.8, 119.9, 116.2, 108.5; HRMS (APCI): Found MH^+ 155.0457 $C_6H_7O_3N_2$: requires 155.0456.

***O*-(2-Nitrophenyl)hydroxylamine (S3)**

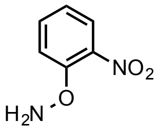

Following **GP1**, 2-fluoronitrobenzene (330 mg, 2.34 mmol) gave O-(2-nitrophenyl)hydroxylamine (160 mg, 75%) as an amorphous solid. ^{1}H NMR (400 MHz, $CDCl_3$) δ 7.89 (1H, dd, J = 8.2, 1.6 Hz), 7.81 (1H, dd, J = 8.6, 1.2 Hz), 7.55 (1H, ddd, J = 8.7, 7.3, 1.7 Hz), 7.02 (1H, ddd, J = 8.4, 7.4, 1.3 Hz), 6.18 (2H, br s); ^{13}C NMR (101 MHz, $CDCl_3$) δ 155.4, 137.4, 134.8, 125.7, 121.0, 116.0; HRMS (APCI): Found MH^+ 155.0457 $C_6H_7O_3N_2$: requires 155.0455. This compound has been reported in the literature,[2] but no spectroscopic data were given.

***O*-(4-Cyanophenyl)hydroxylamine (S4)**

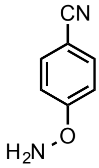

Following **GP1**, 4-fluoro-benzonitrile (363 mg, 3.00 mmol) gave (240 mg, 93 %) as an amorphous solid. ^{1}H NMR (400 MHz, $CDCl_3$) δ 7.50 (2H, d, J = 8.8 Hz), 7.18 (2H, d, J = 8.8 Hz), 6.02 (2H, br s); ^{13}C NMR (101 MHz, $CDCl_3$) δ 164.6, 133.7, 119.4, 113.9, 103.8. HRMS (EI): Found MH^+ 135.0556 $C_7H_7ON_2$ requires 135.0558. Data in accordance with the literature.[3]

O-Phenylhydroxylamine (S6)

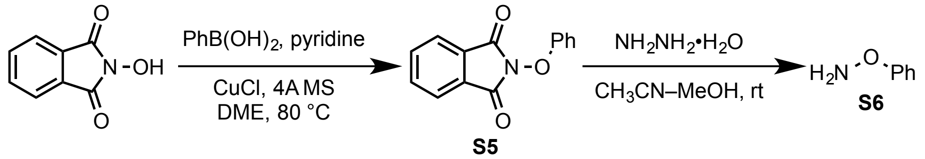

To a solution of *N*-hydroxyphthalimide (0.66 g, 4.05 mmol, 1.0 equiv.) in DCE (20 mL) were added CuCl (0.40 g, 4.05 mmol, 1.0 equiv.), $PhB(OH)_2$ (1.0 g, 8.10 mmol, 2.0 equiv.), pyridine (0.35 mL, 4.45 mmol, 1.1 equiv.) and 4Å molecular sieves (~1 g). The mixture was warmed to 80 °C and stirred for 48 h. The mixture was cooled to room temperature and the molecular sieves removed by filtration. The crude was adsorbed on silica and purified by column chromatography on silica gel, eluting with petrol–EtOAc 99:1→95:5, to **S5** (0.75 g, 81%) as a solid. ^{1}H NMR (400 MHz, $CDCl_3$) δ 7.91 (2H, dd, J = 5.4, 3.1 Hz), 7.81 (2H, dd, J = 5.4, 3.1 Hz), 7.34 (2H, dd, J = 8.6, 7.4 Hz), 7.16 (2H, d, J = 8.6 Hz), 7.13 (1H, d, J = 7.4 Hz). Data in accordance with the literature.[4]

A solution of **S5** (0.75 g, 3.14 mmol, 1.0 equiv.) in $CHCl_3$ (30 mL) was treated with $NH_2NH_2{\bullet}H_2O$ (0.7 mL, 9.42 mmol, 3 equiv.) and stirred overnight. The mixture was adsorbed onto silica gel and purified by column chromatography on silica gel, eluting with petrol–EtOAc 90:10 → 70:30, to give **S6** (0.30 g, 87%) as an oil. ^{1}H NMR (400 MHz, $CDCl_3$) δ 7.18 (2H, dd, J = 8.8, 7.3 Hz), 7.03 (2H, dd, J = 8.8, 1.1 Hz), 6.84 (1H, tt, J = 7.3, 1.1 Hz), 5.73 (2H, br s). Data in accordance with the literature.[3]

General Procedure for the condensation of acetophenone with O-aryl-hydroxylamines 1a–e and 1g – GP2

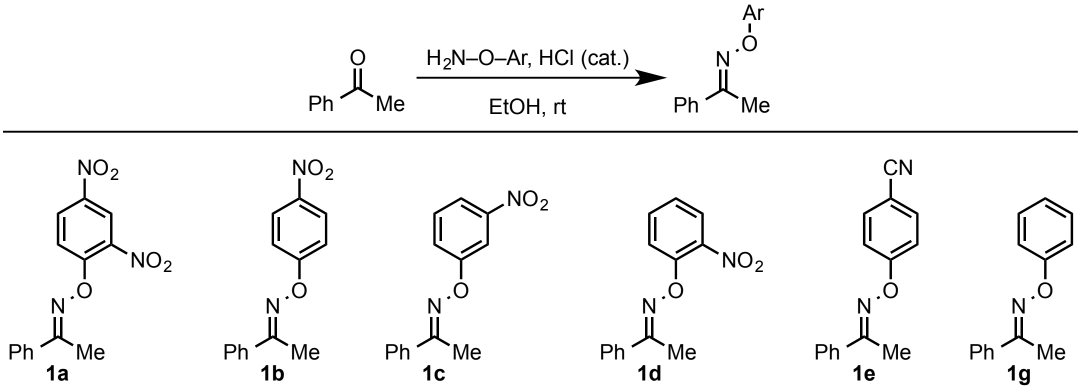

A solution of acetophenone (1.0 equiv.) in EtOH (0.1M) was treated with the O-arylhydroxylamine (1.0 equiv.) and stirred at room temperature overnight. H_2O was added and the EtOH was evaporated. The mixture was diluted with EtOAc and the layers were separated. The aqueous layer was extracted with EtOAc (x 3) and the combined organic layers were dried ($MgSO_4$), filtered and evaporated. Purification by column chromatography on silica gel, eluting with petrol–EtOAc (99:1→97:3), gave the O-aryloxime.

1-Phenylethan-1-one *O*-(2,4-Dinitrophenyl) Oxime (1a)

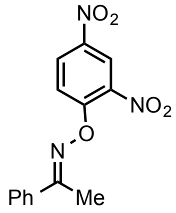

Following **GP2**, acetophenone (500 mg, 4.16 mmol) gave **1a** (1.1 g, 86%) as an amorphous solid. R_f 0.33 [petrol–EtOAc (90:10)]; m.p. 168-170 °C; ^{1}H NMR (400 MHz, $CDCl_3$) δ 8.91 (1H, d, *J* = 2.3 Hz), 8.45 (1H, dd, *J* = 9.4, 2.4 Hz), 8.08 (1H, d, *J* = 9.4 Hz), 7.78 (2H, d, *J* = 7.1 Hz), 7.54 – 7.44 (3H, m), 2.60 (3H, s); ^{13}C NMR (101 MHz, $CDCl_3$) δ 163.6, 157.6, 140.9, 136.2, 134.2, 131.2, 129.6, 129.0, 127.0, 122.3, 117.5, 15.0. This compound has been reported in the literature,[5] but no spectroscopic data were given.

1-Phenylethan-1-one *O*-(4-Nitrophenyl) Oxime (1b)

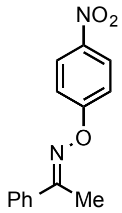

Following **GP2**, acetophenone (38 µL, 0.32 mmol) gave **1b** (45 mg, 54%) as an oil; *E:Z* 13:1. ^{1}H NMR (400 MHz, $CDCl_3$) δ 8.25 (2H, d, *J* = 9.3 Hz), 7.78 (2H, dd, *J* = 7.7, 1.9 Hz), 7.48–7.45 (3H, m), 7.40 (2H, d, *J* = 9.3 Hz), 2.50 (3H, s); ^{13}C NMR (101 MHz, $CDCl_3$) δ 164.3, 160.3, 142.5, 135.2, 130.6, 128.8, 126.8, 125.9, 114.6, 13.9. HRMS (APCI): Found MH^+ 257.0926 $C_{14}H_{13}O_3N_2$: requires 257.0938. Data in accordance with the literature.[6]

1-Phenylethan-1-one O-(3-Nitrophenyl) Oxime (1c)

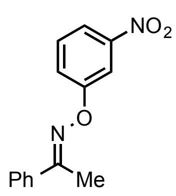

Following **GP2**, acetophenone (38 µL, 0.32 mmol) gave **1c** (45 mg, 54%) as an amorphous solid; ^{1}H NMR (400 MHz, $CDCl_3$) δ 8.18 (1H, t, *J* = 2.2 Hz), 7.90 (1H, ddd, *J* = 8.1, 2.1, 0.8 Hz), 7.81–7.77 (2H, m), 7.59 (1H, ddd, *J* = 8.3, 2.3, 0.8 Hz), 7.51 (1H, d, *J* = 8.2 Hz), 7.50 – 7.46 (3H, m)2.49 (3H, s); ^{13}C NMR (101 MHz, $CDCl_3$) δ 160.1, 159.6, 149.1, 135.3, 130.4, 129.9, 128.8, 126.7, 121.1, 117.0, 110.0, 13.8. HRMS (APCI): Found MH^+ 257.0918 $C_{14}H_{13}O_3N_2$: requires 257.0926. Data in accordance with the literature.[7]

1-Phenylethan-1-one *O*-(2-Nitrophenyl) Oxime (1d)

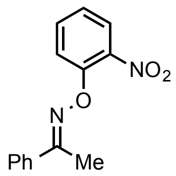

Following **GP2**, acetophenone (38 μL, 0.32 mmol) gave **1d** (76 mg, 92%) as an oil; ^{1}H NMR (400 MHz, $CDCl_3$) δ 7.98 (1H, dd, *J* = 8.2, 1.6 Hz), 7.87 (1H, dd, *J* = 8.5, 1.2 Hz), 7.79–7.76 (2H, m), 7.58 (1H, ddd, *J* = 8.7, 7.3, 1.7 Hz), 7.48–7.41 (3H, m), 7.09 (1H, ddd, *J* = 8.4, 7.4, 1.2 Hz), 2.55 (3H, s); ^{13}C NMR (101 MHz, $CDCl_3$) δ 161.1, 153.3, 137.3, 135.0, 134.8, 130.5, 128.7, 126.8, 125.6, 121.5, 117.2, 14.4; HRMS (APCI): Found MH^+ 257.0938 $C_{14}H_{13}O_3N_2$: requires 257.0926. Data in accordance with the literature.[7]

1-Phenylethan-1-one *O*-(4-Cyanophenyl) Oxime (1e)

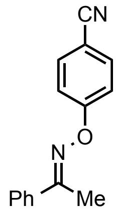

Following **GP2**, acetophenone (43 μL, 0.37 mmol) gave **1e** (89 mg, 99%) as an amorphous solid. R_f 0.46 [petrol–EtOAc (90:10)]; m.p. 104-106 °C; FT-IR ν_{max} (film)/cm^{-1} 2066, 2923, 2223, 1601, 1500, 1443, 1375, 1320, 1235, 1161; ^{1}H NMR (400 MHz, $CDCl_3$) δ 7.77 (2H, dd, *J* = 7.6, 2.0 Hz), 7.62 (2H, d, *J* = 8.9 Hz), 7.47–7.44 (3H, m), 7.37 (2H, d, *J* = 8.9 Hz), 2.47 (3H, s); ^{13}C NMR (101 MHz, $CDCl_3$) δ 162.6, 159.7, 135.2, 133.9, 130.3, 128.7, 126.6, 119.3, 115.3, 105.2, 13.7; HRMS (APCI): Found MH^+ 237.1028 $C_{15}H_{13}ON_2$: requires 237.1035.

1-Phenylethan-1-one *O*-Phenyl Oxime (1g)

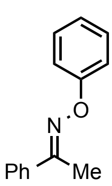

Following **GP2**, acetophenone (32 μL, 0.27 mmol) gave **1g** (94 mg, 99%) as an oil; ^{1}H NMR (500 MHz, $CDCl_3$) δ 7.81–7.77 (2H, m), 7.44–7.41 (3H, m), 7.36–7.32 (2H, m), 7.32–7.29 (2H, m), 7.04 (1H, tt, *J* = 7.0, 1.1 Hz), 2.47 (3H, s); ^{13}C NMR (101 MHz, $CDCl_3$) δ 159.7, 157.9, 136.1, 129.9, 129.4, 128.6, 126.6, 122.2, 114.9, 13.5; LRMS (APCI): 212.1 (MH^+). Data in accordance with the literature.[8]

General Procedure for the synthesis of O-aryl oximes 1f and S7 – GP3

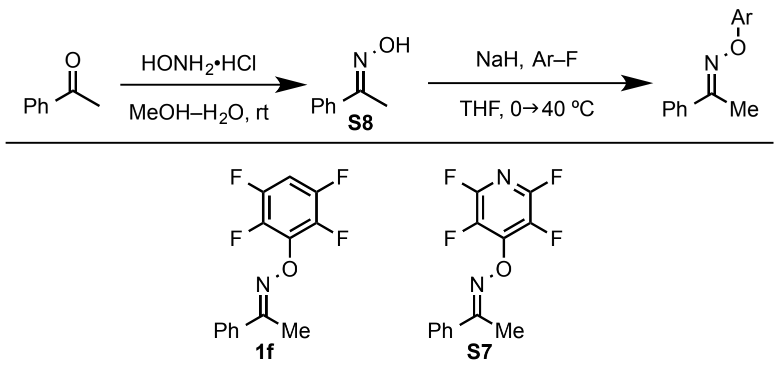

A solution of **S8** (1.0 equiv.) in dry THF (0.5M) under nitrogen was cooled to 0 °C and treated with NaH (1.1 equiv., 60% dispersion in mineral oil). The mixture was stirred at this temperature for 30 minutes before being allowed to warm to room temperature and stirred for 1 h. The fluoro-aryl derivative (1.2 equiv.) was added and the mixture warmed to 40 °C and stirred overnight. The mixture was cooled to room temperature and diluted with H_2O and Et_2O. The layers were separated and the aqueous layer was extracted with Et_2O (x 3). The combined organic layers were dried ($MgSO_4$), filtered and evaporated. Purification by column chromatography on silica gel (petrol–EtOAc 99:1→97:3), gave the corresponding O-aryl oxime.

1-Phenylethan-1-one *O*-(2,3,5,6-Tetrafluorophenyl) Oxime (1f)

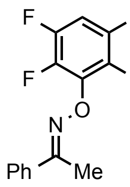

Following **GP3**, **S8** (100 mg, 0.74 mmol) gave **1f** (152 mg, 72%) as an amorphous brown solid. R_f 0.72 [petrol–EtOAc (90:10)]; m.p. 39-42 °C; FT-IR ν_{max} (film)/cm^{-1} 3093, 2923, 1641, 1516, 1499, 1480, 1445, 1373, 1308, 1177, 1062, ; ^{1}H NMR (500 MHz, $CDCl_3$) δ 7.67 (2H, dd, *J* = 8.0, 1.6 Hz), 7.44–7.37 (3H, m), 6.90 (1H, tt, *J* = 10.0, 7.0 Hz), 2.52 (3H, s); ^{13}C NMR (101 MHz, $CDCl_3$) δ 160.9, 146.1 (dtd, *J* = 247.2, 12.4, 4.1 Hz), 141.3 (dddd, *J* = 250.2, 14.8, 4.5, 2.3 Hz), 134.6, 130.3, 128.7, 126.7, 101.4 (t, *J* = 23.0 Hz), 13.6; ^{19}F NMR (376 MHz, $CDCl_3$, decoupled) δ –140.0, –154.8; HRMS (APCI): Found MH^+ 284.0702 $C_{14}H_{10}ONF_4$: requires 284.0699.

1-Phenylethan-1-one *O*-Perfluoropyridin-4-yl Oxime (S7)

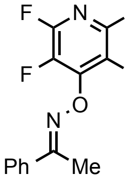

Following **GP3**, **S8** (100 mg, 0.74 mmol) gave **S7** (140 mg, 66%) as an amorphous solid. R_f 0.69 [petrol–EtOAc (90:10)]; m.p. 57-59 °C; FT-IR ν_{max} (film)/cm^{-1} 1643, 1494, 1462, 1446, 1374, 1314, 1139, 1069; ^{1}H NMR (400 MHz, $CDCl_3$) δ 7.72 (1H, d, *J* = 6.7 Hz), 7.51–7.41 (2H, m), 2.53 (2H, s, *J* = 7.9 Hz); ^{13}C NMR (101 MHz, $CDCl_3$) δ 162.6, 147.5 (tt, *J* = 10.9, 10.9, 4.8, 4.8 Hz), 144.1 (ddd, *J* = 242.2, 16.3, 3.4 Hz), 135.0 (ddd, *J* = 262.4, 23.5, 6.1 Hz), 131.0, 130.9 (d, *J* = 25.8 Hz), 128.9, 126.8, 13.7; ^{19}F NMR (471 MHz, $CDCl_3$, decoupled) δ –90.2, –155.2; HRMS (APCI): Found MH^+ 285.0651 $C_{13}H_9F_4O_1N_2$: requires 285.0658.

General Procedures for the synthesis of ketones S9–14 from acid chlorides – GP4

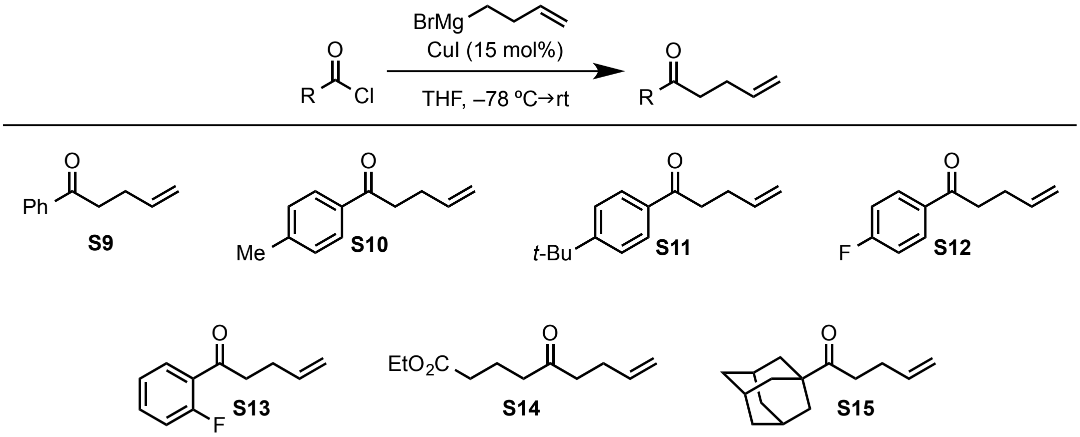

A dry Schlenk tube equipped with a stirring bar was charged with CuI (0.15 equiv.) and then the flask was evacuated and refilled with N_2 (x 3). The acid chloride (1.0 equiv.) and THF (0.1M) were added and the mixture was stirred at room temperature for 10 min and then cooled to –78 ºC. A freshly prepared solution of but-3-en-1-ylmagnesium bromide (1.1 equiv.) was added by dropwise [Grignard preparation: a dry Schlenck tube equipped with a stirring bar was charged with Mg turnings (1.1 equiv.) and then the flask was evacuated and refilled with N_2 (x 3). The minimum amount of THF was added and then 4-bromobut-1-ene (0.3 equiv.) was added neat. Once the Grignard reaction started the remaining 4-bromobut-1-ene (0.7 equiv.) were added as a solution in THF (0.7M). The corresponding mixture was stirred for 1 additional hour]. The mixture was allowed to warm to room temperature overnight. NH_4Cl was added and the mixture was diluted with Et_2O. The layers were separated and the organic layer was dried ($MgSO_4$), filtered and evaporated. Purification by column chromatography on silica gel, eluting with petrol–Et_2O 99:1, gave the ketone.

1-Phenylpent-4-en-1-one (S9)

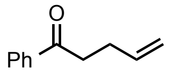

Following **GP4**, benzoyl chloride (3.8 mL, 32.74 mmol) gave **S9** (3.4 g, 65%) as an oil. ^{1}H NMR (400 MHz, $CDCl_3$) δ 7.97 (2H, d, *J* = 7.2 Hz), 7.59–7.43 (3H, m), 5.98–5.84 (1H, m), 5.13–4.99 (2H, m), 3.11–3.06 (2H, m), 2.54–2.46 (2H, m); ^{13}C NMR (101 MHz, $CDCl_3$) δ 199.1, 137.1, 136.8, 132.9, 128.4, 127.9, 115.2, 37.8, 28.2. Data in accordance with the literature.[9]

1-(4-Methylphenyl)pent-4-en-1-one (S10)

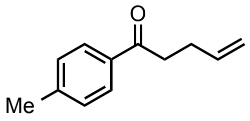

Following **GP4**, *p*-Me-benzoyl chloride (0.3 mL, 2.25 mmol) gave **S10** (320 mg, 89%) was obtained as an oil. ^{1}H NMR (400 MHz, $CDCl_3$) δ 7.89 (2H, d, *J* = 8.2 Hz), 7.30–7.26 (2H, d, *J* = 8.0 Hz), 5.93 (1H, ddt, *J* = 16.8, 10.2, 6.5 Hz), 5.11 (1H, dq, *J* = 17.1, 1.6 Hz), 5.08 (dq, *J* = 17.1, 1.6, 1.3 Hz, 1H), 5.01 (ddd, *J* = 10.2, 3.0, 1.3 Hz, 1H), 2.52 (2H, ddt, *J* = 13.8, 8.2, 1.4 Hz) 2.44 (3H, s); ^{13}C NMR (100 MHz, $CDCl_3$) δ 199.2, 143.8, 137.4, 134.5, 129.3, 128.2, 115.2, 37.6, 28.3, 21.7; LRMS *m/z* (ESI): 174 (M^+), 119, 91. Data in accordance with the literature.[10]

1-(4-*tert*-Butylphenyl)pent-4-en-1-one (S11)

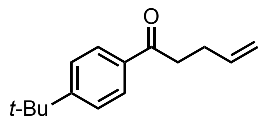

Following **GP4**, *p*-(*t*-Bu)-benzoyl chloride (500 mg, 2.54 mmol) gave **S11** (370 mg, 67%) as an oil. ^{1}H NMR (400 MHz, $CDCl_3$) δ 7.87–7.81 (2H, d, *J* = 8.7 Hz), 7.44–7.37 (2H, d, *J* = 8.7 Hz), 5.84 (1H, ddt, *J* = 16.8, 10.2, 6.5 Hz), 5.02 (1H, dq, *J* = 17.1, 1.6 Hz), 4.94 (1H, dq, *J* = 10.2, 1.3 Hz), 3.02–2.95 (2H, m), 2.46–2.38 (2H, m), 1.27 (9H, s); ^{13}C NMR (100 MHz, $CDCl_3$) δ 199.2, 156.7, 137.5, 134.4, 128.0, 125.5, 115.2, 37.7, 35.1, 31.1, 28.3. Data in accordance with the literature.[11]

1-(4-Fluorophenyl)pent-4-en-1-one (S12)

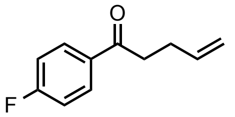

Following **GP4**, *p*-F-benzoyl chloride (0.27 mL, 2.25 mmol) gave **S12** (370 mg, 93%) as an oil. ^{1}H NMR (400 MHz, $CDCl_3$) δ 7.92 (2H, dd, *J* = 8.8, 5.5 Hz), 7.06 (2H, t, *J* = 8.6 Hz),

5.83 (1H, ddt, J = 16.8, 10.2, 6.5 Hz), 5.02 (1H, dd, J = 17.1, 1.5 Hz), 4.95 (1H, dd, J = 10.2, 1.1 Hz), 2.98 (2H, t, J = 7.4 Hz), 2.42 (2H, dd, J = 14.5, 6.8 Hz); ^{13}C NMR (101 MHz, $CDCl_3$) δ 197.8, 165.7 (d, J = 254.5 Hz), 137.2, 133.4 (d, J = 3.0 Hz), 130.7 (d, J = 9.3 Hz), 115.7 (d, J = 21.8 Hz), 115.4, 37.7, 28.1; ^{19}F NMR (376 MHz, $CDCl_3$, decoupled) δ –105.44; LRMS *m/z* (ESI): 178 (M+), 159, 123, 95, 75. Data in accordance with the literature.[11]

1-(2-Fluorophenyl)pent-4-en-1-one (S13)

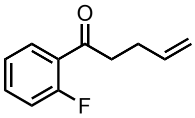

Following **GP4**, *o*-F-benzoyl chloride (0.27 mL, 2.25 mmol) gave **S13** (0.33 g, 83 %) as an oil. ^{1}H NMR (400 MHz, $CDCl_3$) δ 8.04–7.95 (2H, m), 7.18–7.09 (2H, m), 5.90 (1H, ddt, J = 16.9, 10.2, 6.5 Hz), 5.09 (1H, dq, J = 17.2, 1.7 Hz), 5.02 (1H, dq, J = 10.2, 1.4 Hz), 3.10–3.01 (2H, m), 2.55–2.43 (2H, m); ^{13}C NMR (101 MHz, $CDCl_3$) δ 137.4, 134.6 (d, J = 9.0 Hz), 130.7, 124.6 (d, J = 3.4 Hz), 116.8 (d, J = 23.9 Hz), 115.4, 42.9, 28.1; ^{19}F NMR (376 MHz, $CDCl_3$, decoupled) δ -109.5. Data in accordance with the literature.[11]

1-(Adamantan-1-yl)pent-4-en-1-one (S15)

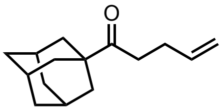

Following **GP4**, adamantoyl chloride (505 mg, 2.54 mmol) gave **S15** (330 mg, 79%) as an oil. FT-IR ν_{max} (film)/cm^{-1} 2901, 2849, 1702, 1640, 1344, 1234, 1195, 1076; ^{1}H NMR (400 MHz, $CDCl_3$) δ 5.74 (1H, ddt, J = 16.8, 10.2, 6.6 Hz), 4.95 (1H, dq, J = 17.1, 1.6 Hz), 4.89 (1H, dq, J = 10.2, 1.2 Hz), 2.47 (2H, t, J = 7.4 Hz), 2.21 (2H, dtt, J = 8.1, 6.9, 1.2 Hz), 1.97 (3H, m), 1.74 (6H, d, J = 2.7 Hz), 1.65 (6H, q, J = 12.2 Hz); ^{13}C NMR (100 MHz, $CDCl_3$) δ 214.8, 137.7, 114.7, 46.3, 38.2, 36.6, 35.3, 28.0, 27.7; HRMS *m/z* (APCI): Found MH^+ 219.1747 $C_{15}H_{23}O$ requires 219.1749.

General Procedures for the synthesis of ketones S22–S28 from carboxylic acids – GP5

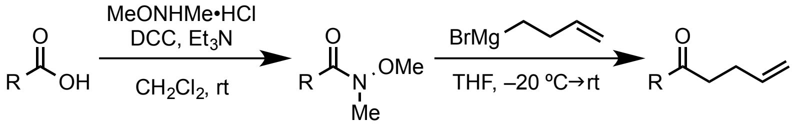

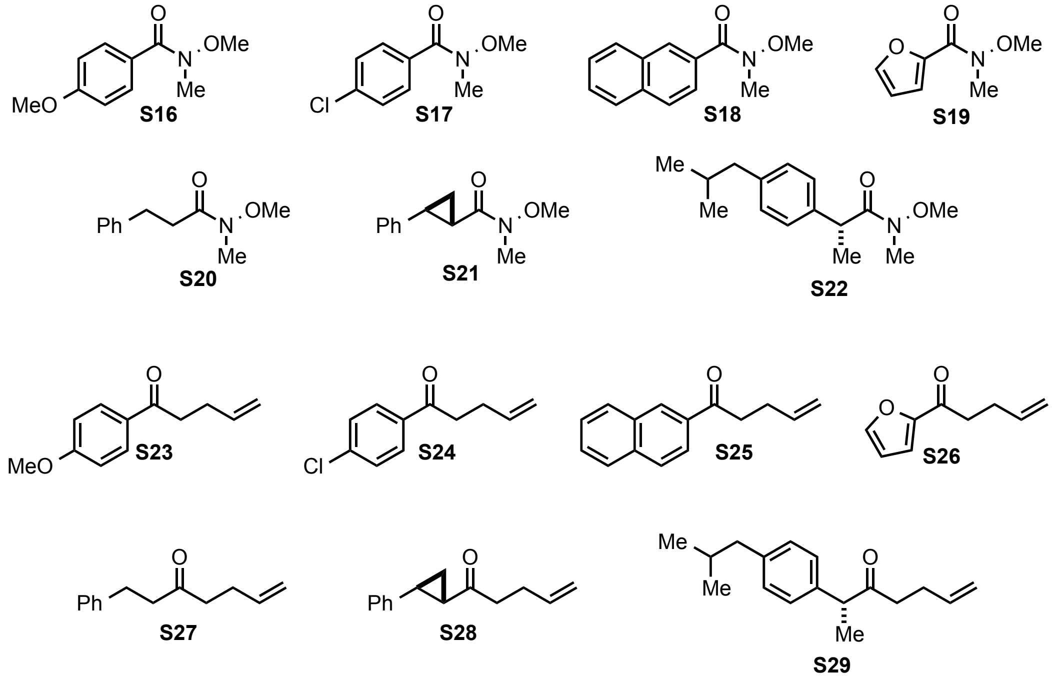

General Procedure for Weinreb amide synthesis – GP5.1

A solution of the carboxylic acid (1.0 equiv.) in CH_2Cl_2 (0.1M) was treated with DCC (1.0 equiv.), *N*,*O*-dimethylhydroxylamine hydrochloride (1.0 equiv.) and Et_3N (1.0 equiv.) and was stirred at room temperature until completion (as determined by TLC analysis). The mixture was diluted with H_2O, the layers were separated and the aqueous layer was extracted with CH_2Cl_2 (x 3). The combined organic layers were dried ($MgSO_4$), filtered and evaporated. Purification by column chromatography on silica gel gave the corresponding Weinreb amides.

General Procedure for Allyl ketone synthesis via Grignard addition – GP5.2

A solution of the Wenreib amide (1.0 equiv.) in THF (0.1M) was cooled to –20 ºC and treated with a freshly prepared solution of but-3-en-1-ylmagnesium bromide (1.1 equiv.) [Grignard preparation: a dry Schlenck tube equipped with a stirring bar was charged with Mg turnings (1.1 equiv.) and then the flask was evacuated and refilled with N_2 (x 3). The minimum amount of THF was added and then 4-bromobut-1-ene (0.3 equiv.) was added neat. Once the Grignard reaction started the remaining 4-bromobut-1-ene (0.7 equiv.) were added as a solution in THF (0.7M). The corresponding mixture was stirred for 1 additional hour]. The mixture was allowed to warm to room temperature overnight. NH_4Cl was added and the mixture was diluted with Et_2O. The layers were separated and the organic layer was dried

($MgSO_4$), filtered and evaporated. Purification by column chromatography on silica gel, eluting with petrol–Et_2O 99:1, gave the ketone.

4-Methoxy-*N*-methoxy-*N*-methylbenzamide (S16)

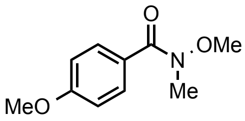

Following **GP5.1**, *p*-OMe-benzoic acid (0.50 g, 3.29 mmol) gave **S16** (570 mg, 89%) as an oil. ^{1}H NMR (400 MHz, $CDCl_3$) δ 7.66 (2H, d, J = 9.0 Hz), 6.84 (2H, d, J = 9.0 Hz), 3.78 (3H, s), 3.49 (3H, s), 3.29 (3H, s); ^{13}C NMR (101 MHz, $CDCl_3$) δ 169.7 161.3, 130.6, 125.9, 113.3, 60.9, 55.2, 33.9; LRMS *m/z* (ESI): 165, 135, 107, 77. Data in accordance with the literature.[12]

4-Chloro-*N*-methoxy-*N*-methylbenzamide (S17)

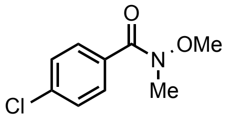

Following **GP5.1**, *p*-Cl-benzoic acid (0.50 g, 3.29 mmol) gave **S17** (450 mg, 71%) as an oil. ^{1}H NMR (400 MHz, $CDCl_3$) δ 7.65 (2H, d, J = 8.6 Hz), 7.38 (2H, d, J = 8.7 Hz), 3.53 (3H, s), 3.36 (3H, s); C NMR (100 MHz, $CDCl_3$): δ 168.7, 136.7, 132.3, 129.8, 128.3, 61.1, 33.5; LRMS *m/z* (ESI): 199 (M^+). Data in accordance with the literature.[13]

***N*-Methoxy-*N*-methyl-2-naphthamide (S18)**

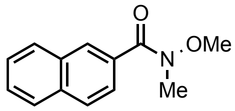

Following **GP5.1**, 2-naphtoic acid (0.3 g, 1.74 mmol) gave **S18** (290 mg, 77%) as an oil. ^{1}H NMR (400 MHz, $CDCl_3$) δ 8.25 (1H, s), 7.93 (1H, dd, J = 7.5, 1.7 Hz), 7.89 (2H, d, J = 8.4 Hz), 7.78 (1H, dd, J = 8.5, 1.7 Hz), 7.57 (2H, dqd, J = 8.4, 6.9, 1.6 Hz), 3.59 (3H, s), 3.44 (3H, s); ^{13}C NMR (101 MHz, $CDCl_3$) δ 169.9, 134.2, 132.5, 131.4, 128.9, 128.7, 127.7, 127.6, 127.4, 126.5, 125.1, 61.1, 33.9; LRMS *m/z* (ESI): 215.1 (M^+). Data in accordance with the literature.[14]

***N*-Methoxy-*N*-methylfuran-2-carboxamide (S19)**

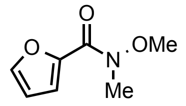

Following **GP5.1**, 2-furoic acid (0.3 g, 2.63 mmol) gave **S19** (310 mg, 76%) as an oil. ^{1}H NMR (400 MHz, $CDCl_3$) δ 7.61 (1H, dd, *J* = 1.7, 0.8 Hz), 7.17 (1H, dd, *J* = 3.5, 0.8 Hz), 6.53 (1H, dd, *J* = 3.5, 1.7 Hz), 3.79 (3H, s), 3.37 (3H, s); ^{13}C NMR (101 MHz, $CDCl_3$) δ 159.1, 145.7, 145.3, 117.4, 111.6, 61.4, 33.2; LRMS *m/z* (ESI): 155.1 (M^+). Data in accordance with the literature.[15]

***N*-Methoxy-*N*-methyl-3-phenylpropanamide (S20)**

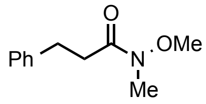

Following **GP5.1**, hydrocynnamic acid (0.30 g, 2.03 mmol) gave **S20** (290 mg, 74%) as an oil. ^{1}H NMR (400 MHz, $CDCl_3$) δ 7.34–7.29 (2H, m), 7.28–7.19 (3H, m), 3.63 (3H, s), 3.20 (3H, s), 2.98 (2H, t, *J* = 7.8 Hz), 2.77 (2H, t, *J* = 7.8 Hz). LRMS *m/z* (ESI): 193.1 (M^+). Data in accordance with the literature.[13]

***N*-Methoxy-*N*-methyl-2-phenylcyclopropane-1-carboxamide (S21)**

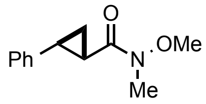

Following **GP5.1**, 2-phenylcyclopropane-1-carboxylic acid (800 mg, 4.93 mmol) gave **S21** (570 mg, 57%) as an oil. ^{1}H NMR (400 MHz, $CDCl_3$) δ 7.32–7.10 (5H, m), 3.68 (3H, s), 3.26 (3H, s), 2.61–2.53 (1H, m), 2.46 (1H, br s), 1.69 (1H, dt, *J* = 8.9, 4.6 Hz), 1.34 (1H, ddd, *J* = 8.3, 6.2, 4.1 Hz); ^{13}C NMR (101 MHz, $CDCl_3$) δ 173.1, 140.7, 128.5, 126.56, 126.37, 126.25, 61.7, 32.6, 26.0, 21.7, 16.5; LRMS *m/z* (ESI): 193.1 (M^+). Data in accordance with the literature.[16]

2-(4-Isobutylphenyl)-*N*-methoxy-*N*-methylpropanamide (S22)

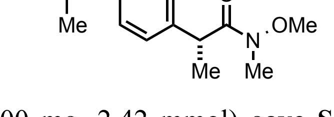

Following **GP5.1**, ibuprofen (500 mg, 2.42 mmol) gave **S22** (470 mg, 78%) as an oil. ^{1}H NMR (400 MHz, $CDCl_3$) δ 7.11 (2H, d, *J* = 8.1 Hz), 6.98 (2H, d, *J* = 8.1 Hz), 4.01 (1H, dd, *J* = 9.0, 5.3 Hz), 3.27 (3H, s), 3.04 (3H, s), 2.33 (2H, d, *J* = 7.2 Hz), 1.73 (1H, sept, *J* = 6.8 Hz), 1.33 (3H, d, *J* = 7.1 Hz), 0.78 (6H, d, *J* = 6.6 Hz); ^{13}C NMR (101 MHz,

$CDCl_3$) δ 175.5, 140.0, 139.1, 129.3, 127.3, 61.0, 45.0, 41.5, 30.2, 22.38, 22.36, 19.6; LRMS *m/z* (ESI): 249.1 (M^+). Data in accordance with the literature.[17]

1-(4-Methoxyphenyl)pent-4-en-1-one (S23)

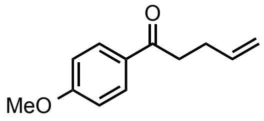

Following **GP5.2**, **S16** (0.44 g, 2.25 mmol) gave **S23** (290 mg, 74%) as an oil. ^{1}H NMR (400 MHz, $CDCl_3$) δ 7.93 (2H, d, *J* = 9.0 Hz), 6.91 (2H, d, *J* = 9.0 Hz), 5.93 (1H, ddt, *J* = 16.8, 10.2, 6.5 Hz), 5.11 (1H, dq, *J* = 17.1, 1.7 Hz), 5.03 (1H, dq, *J* = 10.2, 1.5 Hz), 3.90 (3H, s), 3.01 (2H, t, *J* = 7.2 Hz), 2.59–2.44 (2H, m); ^{13}C NMR (101 MHz, $CDCl_3$) δ 198.1, 163.6, 137.5, 130.3, 130.1, 115.2, 113.7, 55.5, 37.4, 28.4; LRMS *m/z* (ESI): 190 (M^+). Data in accordance with the literature.[18]

1-(4-Chlorophenyl)pent-4-en-1-one (S24)

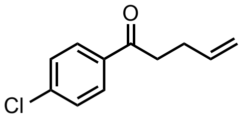

Following **GP5.2**, **S17** (0.45 g, 2.25 mmol) gave **S24** (300 mg, 75%) as an oil. ^{1}H NMR (400 MHz, $CDCl_3$) δ 7.93 (2H, d, *J* = 8.7 Hz), 7.46 (2H, d, *J* = 8.7 Hz), 5.92 (1H, ddt, *J* = 16.8, 10.2, 6.5 Hz), 5.11 (1H, dq, *J* = 17.1, 1.6 Hz), 5.04 (1H, dq, *J* = 10.2, 1.4 Hz), 3.06 (2H, t, *J* = 7.3 Hz), 2.58–2.43 (2H, m); ^{13}C NMR (100 MHz, $CDCl_3$) δ 198.4, 139.5, 137.1, 135.1, 129.5, 128.9, 115.5, 37.7, 28.1; LRMS *m/z* (ESI): 195 (M^+). Data in accordance with the literature.[10]

1-(Naphthalen-2-yl)pent-4-en-1-one (S25)

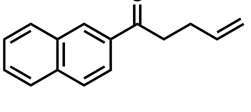

Following **GP5.2**, **S18** (0.29 g, 1.35 mmol) gave **S25** (280 mg, 79%) as an oil. ^{1}H NMR (400 MHz, $CDCl_3$) δ 8.51 (1H, s), 8.07 (1H, dd, *J* = 8.6, 1.5 Hz), 7.99 (1H, d, *J* = 8.0 Hz), 7.92 (2H, t, *J* = 8.0 Hz), 7.66–7.54 (2H, m), 5.98 (1H, ddt, *J* = 16.8, 10.2, 6.5 Hz), 5.15 (1H, dd, *J* = 17.1, 1.5 Hz), 5.07 (1H, d, *J* = 10.2 Hz), 3.25 (2H, t, *J* = 7.0 Hz), 2.59 (2H, q, *J* = 7.0 Hz); ^{13}C NMR (101 MHz, $CDCl_3$) δ 199.5, 137.4, 135.6, 134.2, 132.5, 129.7, 129.6, 128.5, 128.5, 127.8, 126.8, 123.9, 115.4, 37.8, 28.3; LRMS *m/z* (ESI): 210.1 (M^+). Data in accordance with literature.[19]

1-(Furan-2-yl)pent-4-en-1-one (S26)

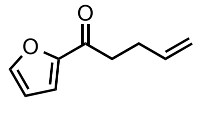

Following **GP5.2**, **S19** (0.31 g, 1.97 mmol) gave **S26** (210 mg, 71%) as an oil. ^{1}H NMR (400 MHz, CDCl$_3$) δ 7.60 (1H, d, *J* = 0.8 Hz), 7.21 (1H, d, *J* = 3.5 Hz), 6.55 (1H, dd, *J* = 3.5, 1.6 Hz), 5.90 (1H, ddt, *J* = 16.8, 10.2, 6.5 Hz), 5.10 (1H, dd, *J* = 17.1, 1.5 Hz), 5.03 (1H, dd, *J* = 10.2, 1.1 Hz), 2.95 (2H, t, *J* = 7.5 Hz), 2.50 (2H, q, *J* = 7.5 Hz); ^{13}C NMR (101 MHz, CDCl$_3$) δ 188.8, 152.7, 146.4, 137.0, 117.1, 115.5, 112.2, 37.6, 28.1; LRMS *m/z* (ESI): 150.1 (M$^+$). Data in accordance with literature.[20]

1-Phenylhept-6-en-3-one (S27)

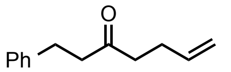

Following **GP5.2**, **S20** (0.29 g, 1.49 mmol) gave **S27** (190 mg, 67%) as an oil. ^{1}H NMR (400 MHz, CDCl$_3$) δ 7.30 (2H, t, *J* = 7.9 Hz), 7.20 (2H, t, *J* = 8.0 Hz), 5.81 (1H, ddt, *J* = 16.8, 10.2, 6.5 Hz), 5.09–4.71 (2H, m), 2.93 (2H, t, *J* = 7.6 Hz), 2.76 (2H, t, *J* = 7.6 Hz), 2.52 (2H, t, *J* = 7.4 Hz), 2.34 (2H, q, *J* = 6.8 Hz); ^{13}C NMR (101 MHz, CDCl$_3$) δ 209.4, 141.1, 137.1, 128.5, 128.4, 126.1, 115.3, 44.4, 42.0, 29.7, 27.7; LRMS *m/z* (ESI): 188.1 (M$^+$). Data in accordance with literature.[21]

1-(2-Phenylcyclopropyl)pent-4-en-1-one (S28)

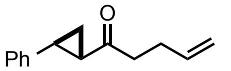

Following **GP5.2**, **S21** (570 mg, 2.78 mmol) gave **S28** (330 mg, 79%) as an oil. ^{1}H NMR (400 MHz, CDCl$_3$) δ 7.34–7.28 (2H, m), 7.26–7.21 (1H, m), 7.13–7.10 (2H, m), 5.85 (1H, ddt, *J* = 17.0, 10.3, 6.6 Hz), 5.09–4.99 (2H, m), 2.73 (2H, t, *J* = 7.4 Hz), 2.54 (1H, ddd, *J* = 9.0, 6.6, 4.0 Hz), 2.41 (2H, dtt, *J* = 7.9, 6.6, 1.3 Hz), 2.23 (1H, ddd, *J* = 8.1, 5.3, 4.0 Hz), 1.70 (1H, ddd, *J* = 9.1, 5.2, 4.1 Hz), 1.40 (1H, ddd, *J* = 8.1, 6.6, 4.2 Hz); LRMS *m/z* (ESI): 200.1 (M$^+$).

2-(4-Isobutylphenyl)hept-6-en-3-one (S29)

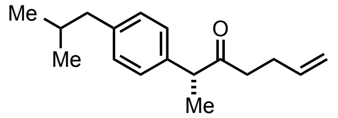

Following **GP5.2**, **S22** (324 mg, 1.30 mmol) gave **S29** (240 mg, 75%) as an oil. ^{1}H NMR (400 MHz, CDCl$_3$) δ 7.03 (4H, s), 5.62 (1H, ddt, *J* = 17.0, 10.3, 6.6 Hz), 4.87–4.80 (2H,

m), 3.65 (1H, q, J = 7.0 Hz), 2.40–2.35 (4H, m), 2.24–2.08 (2H, m), 1.82–1.72 (1H, m), 1.31 (3H, d, J = 7.0 Hz), 0.82 (6H, d, J = 6.6 Hz); ^{13}C NMR (101 MHz, $CDCl_3$) δ 210.3, 140.6, 137.7, 137.2, 129.6, 127.6, 115.0, 52.7, 45.0, 40.0, 30.2, 27.9, 22.4, 17.4; LRMS *m/z* (ESI): 244.2 (M^+). HRMS *m/z* (APCI): Found MH^+ 245.1907. $C_{17}H_{25}O$ requires 245.1905.

1-(Pyridin-3-yl)pent-4-en-1-one (S31)

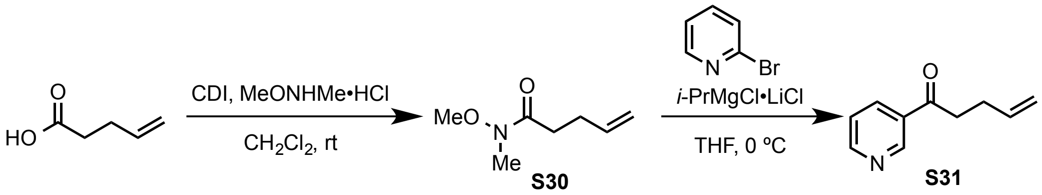

A solution of 4-pentanoic acid (1.8 mL, 18 mmol, 1 equiv.) in CH_2Cl_2 (180 mL) was cooled to 0 °C, treated with carbonyl diimidazole (3.9 g, 22 mmol, 1.2 equiv.) and stirred at the same temperature for 30 min. *N-O*-Dimethylhydroxylamine hydrogenchloride (4.9 g, 45 mmol, 2.5 equiv.) was added and the mixture was allowed to warm to room temperature overnight. H_2O (50 mL) was added and the layers separated. The aqueous layer was extracted with CH_2Cl_2 (3 x 20 mL). The organic layers were collected, dried ($MgSO_4$), filtered and evaporated. Purification by column chromatography column chromatography on silica gel gave **S30** as an oil (2.6 g, 99%). ^{1}H NMR (400 MHz, $CDCl_3$) δ 5.86 (1H, ddt, J = 16.7, 10.2, 6.5 Hz), 5.09 (1H, dd, J = 17.1, 1.6 Hz), 4.99 (1H, dd, J = 10.3, 1.4 Hz), 3.68 (3H, s), 3.18 (3H, s), 2.53 (2H, t, J = 7.4 Hz), 2.41 (2H, q, J = 7.4 Hz). Data in accordance with literature.[22]

A dry Schlenk tube equipped with a stirring bar was charged with 2-bromopyridine (0.60 mL, 6.3 mmol, 1 equiv.) and dry THF (6.2 mL). The solution was cooled to 0 °C and *i*-PrMgCl•LiCl (4.9 mL, 6.3 mmol, 1 equiv., 1.3 M in THF) was added dropwise. The mixture was stirred at 0 °C for 4 h and then **S30** (0.9 g, 6.3 mmol, 1 equiv.) was added. The mixture was allowed to warm to room temperature overnight and then H_2O (10 mL) and Et_2O (20 mL) were added. The layers were separated and the aqueous layer was extracted with Et_2O (3 x 20 mL). The combined organic layers were dried ($MgSO_4$), filtered and evaporated. Purification by column chromatography on silica gel, gave **S31** as an oil. ^{1}H NMR (400 MHz, $CDCl_3$) δ 9.18 (1H, d, J = 1.5 Hz), 8.78 (1H, dd, J = 4.8, 1.6 Hz), 8.24 (1H, dt, J = 8.0, 1.9 Hz), 7.43 (1H, dd, J = 7.9, 4.8 Hz), 5.90 (1H, ddt, J = 16.8, 10.2, 6.5 Hz), 5.10 (1H, dd, J = 17.1, 1.5 Hz), 5.03 (1H, dd, J = 10.2, 1.2 Hz), 3.10 (2H, t, J = 7.3 Hz), 2.52 (2H, q, J = 6.9 Hz); ^{13}C NMR (101 MHz, $CDCl_3$) δ 198.4, 153.6, 149.7, 136.9, 135.5, 132.2, 123.8, 115.9, 38.2, 27.9. Data in accordance with the literature.[23]

General procedure for the synthesis of ketones S34 and S35 – GP6

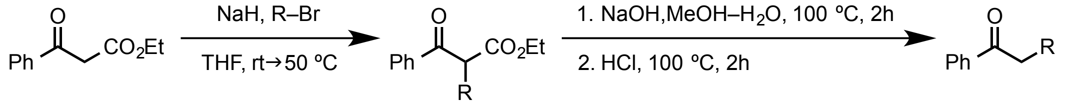

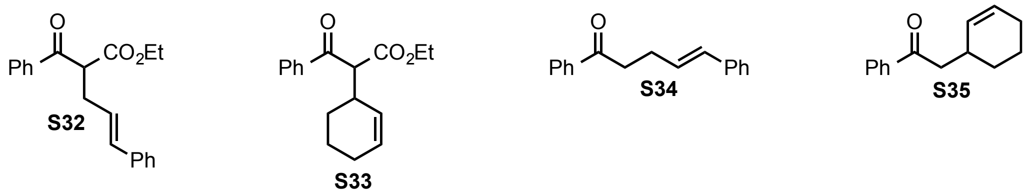

GP6.1

A solution of ethyl 3-oxo-3-phenylpropanoate (1.0 equiv.) in THF (0.17M) was treated with NaH (1.0 equiv., 60% in mineral oil), stirred for 1h and treated with the allylic bromide (1.1 equiv.). The mixture was warmed to 50 ºC and stirred overnight. The mixture was cooled to room temperature and MeOH was added. The crude product was absorbed on silica and purified by column chromatography on silica gel eluting with petrol–Et_2O (95:5) to give the product.

GP6.2

A solution of the β-ketoester (1.0 equiv.) in MeOH–H_2O (0.01M, 2:1) was treated with NaOH (4.0 equiv.) and heated under reflux for 2h (at this point TLC analysis revealed complete hydrolysis of the ester). The mixture was cooled to room temperature, slowly acidified with HCl to pH=1 and heated under reflux for 2h (at this point TLC analysis revealed complete decarboxylation of the β-ketoacid). The mixture was cooled to room temperature, diluted with H_2O and Et_2O. The layers were separated and the organic layer was dried ($MgSO_4$), filtered and evaporated. Purification by column chromatography on silica gel, eluting with petrol-Et_2O 99:1, gave the ketone.

Ethyl (*E*)-2-benzoyl-5-phenylpent-4-enoate (S32)

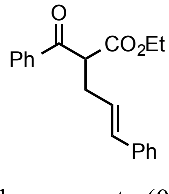

Following **GP6.1**, ethyl 3-oxo-3-phenylpropanoate (0.45 mL, 2.60 mmol) gave **S32** (690 mg, 86%) as an oil. ^{1}H NMR (400 MHz, $CDCl_3$) δ 7.96 (2H, d, J = 12 Hz), 7.12–7.56 (8H, m), 6.42 (1H, d, J = 15.5 Hz), 6.18 (1H, ddd, J = 15.5, 8.0, 8.0 Hz), 4.41 (1H, t, J = 8.0 Hz), 4.11 (2H, q, J = 7.5 Hz), 2.86 (2H, m), 1.12 (3H, t, J = 7.5 Hz); ^{13}C NMR (101 MHz, $CDCl_3$) δ 194.1, 169. 1, 136.8, 136.0, 133.3, 132.4, 128.5, 128.4, 128.4, 128.3, 128.0, 127.1, 125.9, 125.9, 61.33, 54.2, 32.3, 14.0. Data in accordance with the literature.[24]

(*E*)-1,5-Diphenylpent-4-en-1-one (S34)

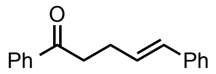

Following **GP6.2**, **S32** (690 mg, 2.24 mmol) gave **S34** (500 mg, 94%) as an oil. ^{1}H NMR (400 MHz, $CDCl_3$) δ 7.98 (2H, dt, *J* = 7.8, 1.3 Hz), 7.57 (1H, tt, *J* = 7.8, 1.5 Hz), 7.47 (2H, t, *J* = 7.9 Hz), 7.34 (2H, d, *J* = 7.1 Hz), 7.29 (2H, t, *J* = 7.5 Hz), 7.22 (1H, tt, *J* = 7.5, 1.3 Hz), 6.50 (1H, d, *J* = 16.3 Hz), 6.33 (1H, dt, *J* = 16.3, 7.4 Hz), 3.17 (2H, t, *J* = 7.4 Hz), 2.64 (2H, qd, *J* = 7.3, 1.3 Hz); ^{13}C NMR (101 MHz, $CDCl_3$) δ 199.3, 137.5, 137.2, 133.1, 130.5, 129.2, 128.4, 128.5, 128.1, 127.3, 126.1, 38.5, 27.5. Data in accordance with the literature.[25]

2-(Cyclohex-2-en-1-yl)-1-phenylethan-1-one (S35)

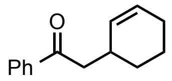

Following **GP6.2**, **S35** (442 mg, 1.61 mmol) gave **S36** (266 mg, 82%) as an oil. ^{1}H NMR (400 MHz, $CDCl_3$) δ 7.96 (2H, d, *J* = 7.2 Hz), 7.59–7.42 (3H, m), 5.75–5.70 (1H, m), 5.62–5.57 (1H, m), 2.97–2.74 (3H, m), 2.04–1.50 (5H, m), 1.37–1.24 (1H, m); ^{13}C NMR (101 MHz, $CDCl_3$) δ 191.3, 137.2, 133.0, 130.8, 128.6, 128.1, 128.0, 44.8, 31.6, 29.1, 25.1, 21.1. Data in accordance with the literature.[26]

10-Hydroxydec-1-en-5-one (S37)

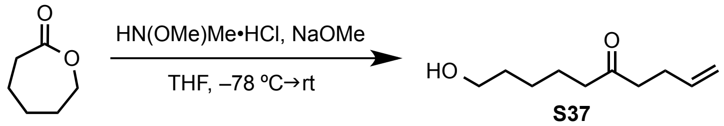

S37

A slurry of lactone e-caprolactone (250 mg, 2.19 mmol, 1.0 equiv.), HN(OMe)Me•HCl (260 mg, 2.63 mmol, 1.2 equiv.), and NaOMe (30 mg, 0.55 mmol, 25 mol%) in THF (9.0 mL) was cooled to –78 °C and treated with a freshly prepared solution of but-3-en-1-ylmagnesium bromide [Grignard preparation: a dry Schlenck tube equipped with a stirring bar was charged with Mg turnings (570 mg, 23.9 mmol, 8.1 equiv.) and then the flask was evacuated and refilled with N_2 (x 3). The minimum amount of THF was added and then 4-bromobut-1-ene (6.2 mL, 23.40 mmol, 8.1 equiv.) was added as a solution in THF (23 mL). The corresponding mixture was stirred for 1 additional hour]. The mixture was allowed to warm to room temperature overnight. NH_4Cl was added and the mixture was diluted with Et_2O. The layers were separated and the organic layer was dried ($MgSO_4$), filtered and evaporated. Purification by column chromatography on silica gel, eluting with petrol–Et_2O 95:5, gave **S37** (310 mg, 83%) as an oil.

tert-Butyl 3-Allyl-4-oxopiperidine-1-carboxylate (S38)

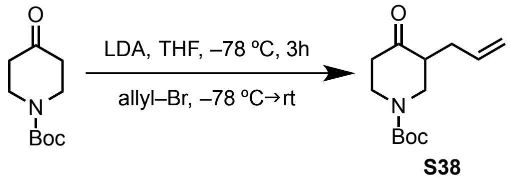

A dry flask equipped with a magnetic stirring bar was charged with *i*-Pr_2NH (0.12 mL, 0.86 mmol, 1.0 equiv.) and THF (2.0 mL) and it was cooled to –78 ºC. *n*-BuLi (0.55 mL, 0.86 mmol, 1.0 equiv., 1.6M in hexane) was added by dropwise and the mixture was stirred at the same temperature for 1 h. *tert*-Butyl 4-oxopiperidine-1-carboxylate (172 mg, 0.95 mmol, 1.1 equiv.) was added as a solution in THF (2.0 mL) at –78 ºC and the mixture was stirred at the same temperature for 3 h. Allyl bromide (80 μL, 0.948 mmol, 1.1 equiv.) was added by dropwise and the mixture was allowed to warm to room temperature overnight. The mixture was diluted with NH4Cl (5 mL) and EtOAc (10 mL). The layers were separated and the aqueous layer was extracted with EtOAc (2 x 5 mL). The combined organic layers were dried (MgSO4), filtered and evaporated. Purification by column chromatogtraphy on silica gel, eluting with petrol:EtOAc 98:2, gave **S38** (50 mg, 24%) as an oil. ^{1}H NMR (400 MHz, $CDCl_3$, rotamers) δ 5.75–5.65 (1H, m), 5.04–4.98 (2H, m), 4.16–3.98 (2H, m), 3.31–2.83 (2H, m), 2.49–2.32 (4H, m), 2.02–1.94 (1H, m), 1.42 (9H, s). Data in accordance with the literature.[27]

General procedure for the synthesis of *O*-aryl-oximes 2a–d – GP7

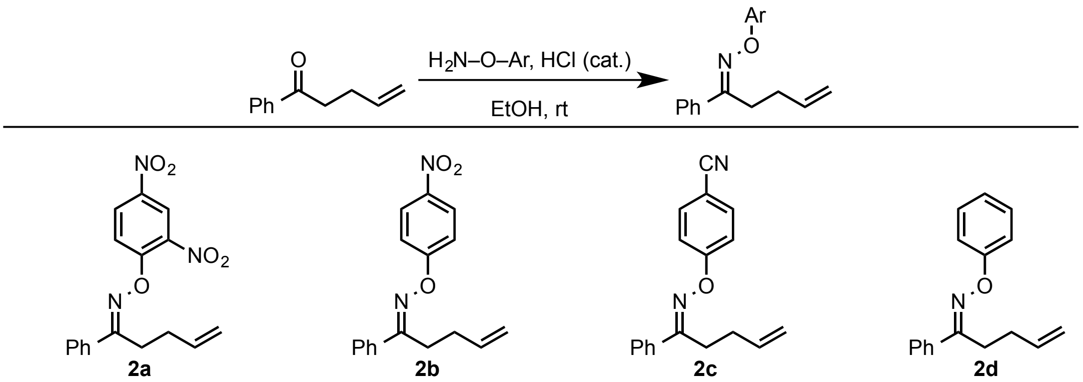

A solution of **S9** (1.0 equiv.) in EtOH (0.1M) was treated with the *O*-arylhydroxylamine (1.0 equiv.) and HCl (1 drop) and stirred at room temperature overnight. H_2O was added and the EtOH was evaporated. The mixture was diluted with EtOAc and the layers were separated. The aqueous layer was extracted with EtOAc (x 3) and the combined organic layers were dried ($MgSO_4$), filtered and evaporated. Purification by column chromatography on silica gel, eluting with petrol–EtOAc (99:1→97:3), gave the *O*-aryloxime.

1-Phenylpent-4-en-1-one *O*-(2,4-Dinitrophenyl) Oxime (2a)

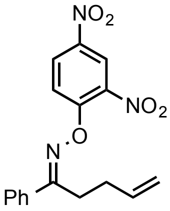

Following **GP7** but purifying **2a** by filtration (see **GP8**), **S9** (400 mg, 2.74 mmol) gave **2a** (650 mg, 70%) as an amorphous yellow solid; *E*:*Z* 10:1. R_f 0.78 [petrol–EtOAc (75:25)]; m.p. 73-76 °C; FT-IR ν_{max} (film)/cm^{-1} 3119, 1605, 1520, 1338, 1313, 1280, 1258, 1235, 1139, 1063; ^{1}H NMR (400 MHz, $CDCl_3$) δ 8.96 (1H, d, *J* = 2.7 Hz,), 8.48 (1H, dd, *J* = 9.4, 2.8 Hz), 8.10 (1H, d, *J* = 9.4 Hz), 7.78 (2H, dd, *J* = 8.2, 1.5 Hz), 7.56–7.48 (3H, m), 5.90 (1H, ddt, *J* = 16.9, 10.1, 6.8 Hz), 5.12 (1H, dq, *J* = 17.0, 1.5 Hz), 5.05 (1H, dq, *J* = 10.1, 1.5, 1.5 Hz), 3.17 (2H, t, *J* = 7.6 Hz), 2.46 (2H, q, *J* = 7.4 Hz); ^{13}C NMR (101 MHz, $CDCl_3$) δ 166.8, 136.3, 133.2, 131.1, 129.5, 129.0, 128.8, 127.2, 122.2, 117.4, 116.3, 30.9, 28.4; HRMS *m/z* (APCI): Found MH$^+$ 342.1078 $C_{17}H_{16}O_5N_3$ requires 342.1090. X-ray quality crystals of **2a** were by slow infusion of *n*-hexane into a solution of **2a** in Et_2O.

1-Phenylpent-4-en-1-one *O*-(4-Nitrophenyl) Oxime (2b)

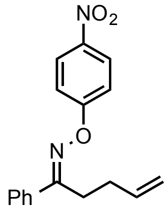

Following **GP7**, **S9** (100 mg, 0.65 mmol) gave **2b** (190 mg, 97%) as an amorphous orange oil; *E:Z* 3.5:1. R_f 0.54 [petrol–EtOAc (90:10)]; FT-IR ν_{max} (film)/cm^{-1} 3078, 2937, 1589, 1514, 1487, 1334, 1290, 1235, 1159, 1110; ^{1}H NMR (400 MHz, $CDCl_3$) δ 8.28 (2H, d, *J* = 9.3 Hz), 7.78 (2H, dd, *J* = 7.6, 1.8 Hz), 7.49 (3H, m), 7.42 (2H, d, *J* = 9.3 Hz), 5.95–5.82 (1H, m), 5.09–5.02 (2H, m), 3.09 (2H, t, *J* = 7.9 Hz), 2.42 (2H, q, *J* = 7.3 Hz); ^{13}C NMR (101 MHz, $CDCl_3$, *E:Z* isomers) δ 164.3^M & 164.3^m, 163.6^M & 163.5$_m$, 142.5^M & 142.3^m, 136.8$^{M\,\&\,m}$, 134.2^M & 132.9^m, 130.6^M & 129.7^m, 128.9^M & 128.5^m, 127.7^m & 127.1^M, 125.9^M & 125.8^m, 115.9^M & 115.8^m, 114.7^M & 114.5^m, 35.1^m & 30.9^M, 30.4^m & 27.2^M; HRMS (APCI): Found MH$^+$ 297.1239 $C_{17}H_{17}O_3N_2$: requires 297.1239.

1-Phenylpent-4-en-1-one *O*-(4-Cyanophenyl) Oxime (2c)

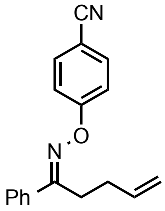

Following **GP7**, **S9** (120 mg, 0.75 mmol) gave **2c** (200 mg, 95%) as an pale yellow oil; *E*:*Z* 7:1. R_f 0.49 [petrol–EtOAc (90:10)]; FT-IR ν_{max} (film)/cm^{-1} 3057, 2224, 1600, 1499, 1232, 1161; ^{1}H NMR (400 MHz, $CDCl_3$) δ 7.74 (2H, dd, *J* = 7.7, 1.9 Hz), 7.63 (2H, dt, *J* = 9.0, 2.2 Hz), 7.48–7.43 (3H, m), 7.37 (2H, dt, *J* = 9.0, 2.2 Hz), 5.89 (1H, ddt, *J* = 16.8, 10.1, 6.6 Hz), 5.10 (1H, dq, *J* = 17.0, 1.5 Hz, 1H), 5.04 (dq, *J* = 10.1, 1.3 Hz, 1H), 3.05 (2H, t, *J* = 7.8 Hz), 2.43–2.35 (2H, m); ^{13}C NMR (101 MHz, $CDCl_3$) δ 162.9, 162.4, 136.6, 134.1, 133.6, 130.2, 128.6, 126.8, 119.1, 115.6, 115.2, 105.4, 30.6, 26.9; HRMS (APCI): Found MH^+ 277.1341 $C_{18}H_{17}ON_2$: requires 277.1337.

1-Phenylpent-4-en-1-one *O*-Phenyl Oxime (2d)

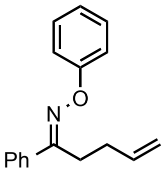

Following **GP7**, **S9** (73 mg, 0.46 mmol) gave **2d** (140 mg, 89%) as an amorphous solid; *E*:*Z* 9:1. R_f 0.74 [petrol–EtOAc (90:10)]; FT-IR ν_{max} (film)/cm^{-1} 3062, 1592, 1488, 1444 1213, 1159, 1023; ^{1}H NMR (500 MHz, $CDCl_3$) δ 7.77–7.74 (2H, m), 7.44–7.40 (3H, m), 7.36–7.31 (2H, m), 7.31–7.27 (2H, m), 7.04 (1H, tt, *J* = 7.3, 1.2 Hz), 5.89 (1H, ddt, *J* = 16.8, 10.2, 6.6 Hz), 5.08 (1H, dq, *J* = 17.1, 1.6 Hz), 5.01 (1H, dq, *J* = 10.2, 1.4 Hz), 3.09–2.99 (2H, m), 2.43–2.38 (2H, m); ^{13}C NMR (101 MHz, $CDCl_3$) δ 161.2, 159.7, 137.3, 135.2, 129.8, 129.4, 128.7, 126.9, 122.3, 115.5, 115.0, 30.9, 26.8; HRMS (APCI): Found MH^+ 252.1388 $C_{14}H_{25}O_2$: requires 252.1396. Data in accordance with the literature.[28]

General procedure for the synthesis of *O*-aryl-oximes S29–45 – GP8

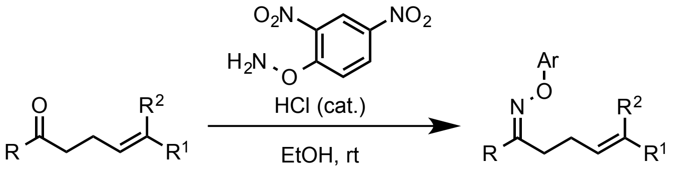

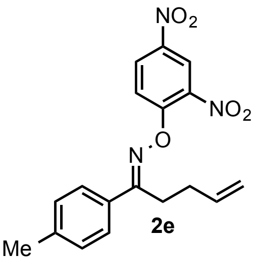
2e

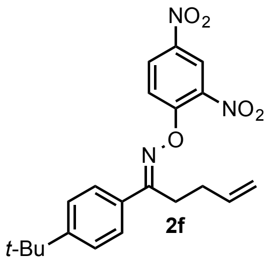
2f

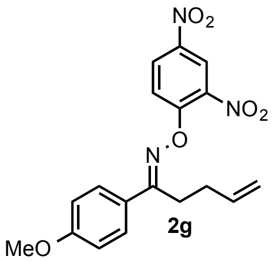
2g

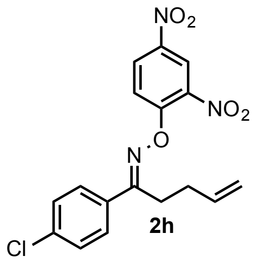
2h

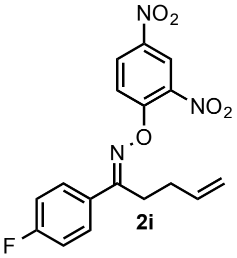
2i

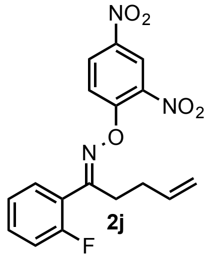
2j

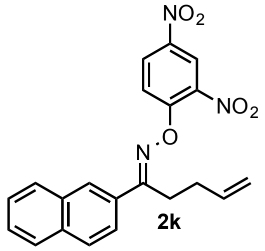
2k

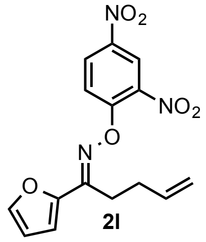
2l

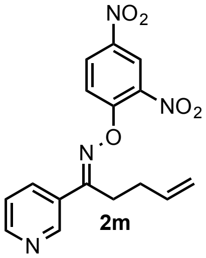
2m

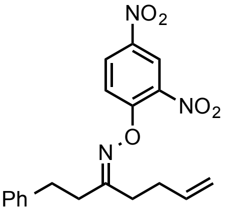
2n

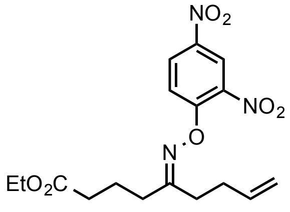
2o

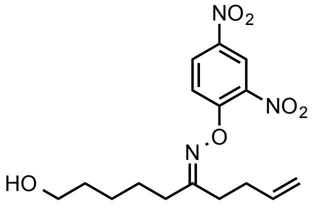
2p

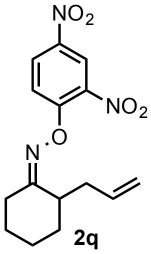
2q

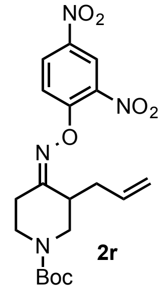
2r

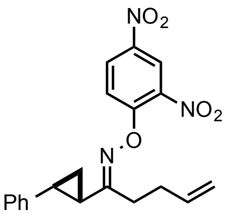
2s

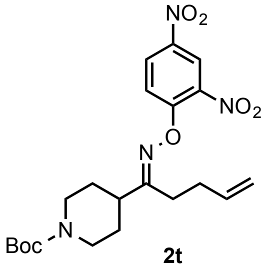
2t

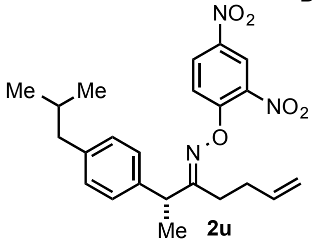
2u

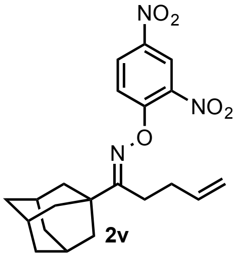
2v

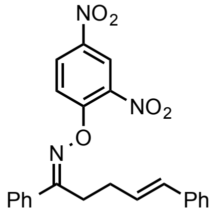
2w

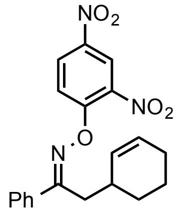
2y

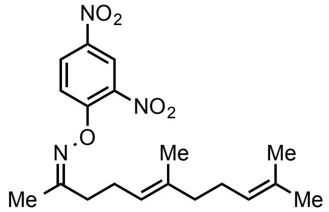
2z

A solution of the ketone (1.0 equiv.) in EtOH (0.1M) was treated with the *O*-(2,4-dinitrophenyl)hydroxylamine (1.0 equiv.) and HCl (1-3 drops). Upon addition of HCl the mixture became a yellow solution and over time the product crystallized out (see picture for the reaction leading to **2a**). Once the reaction was judged complete by TLC analysis the pure product was filtered washing with cold *n*-hexane and dried under high vacuum for 2h.

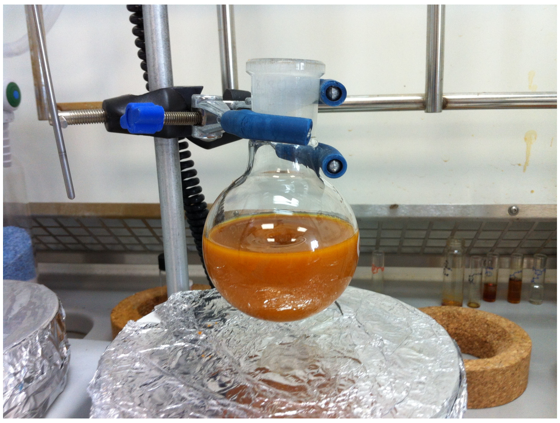

1-(4-Methylphenyl)pent-4-en-1-one *O*-(2,4-Dinitrophenyl) Oxime (2e)

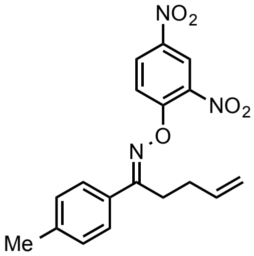

Following **GP8**, **S10** (155 mg, 0.89 mmol) gave **2e** (220 mg, 70%) as an amorphous solid; *E*:*Z* 10:1. R_f 0.81 [petrol–EtOAc (75:25)]; m.p. 88-91 °C; FT-IR ν_{max} (film)/cm^{-1} 1603, 1525, 1469, 1339, 1310, 1259; ^{1}H NMR (400 MHz, $CDCl_3$) δ 8.86 (1H, d, *J* = 2.7 Hz), 8.38 (1H, dd, *J* = 9.4, 2.8 Hz), 8.00 (1H, d, *J* = 9.4 Hz), 7.58 (2H, d, *J* = 8.0 Hz), 7.21 (2H, d, *J* = 7.9 Hz), 5.81 (1H, ddt, *J* = 16.9, 10.1, 6.8 Hz), 5.01 (1H, dq, *J* = 17.0, 1.5 Hz), 4.94 (1H, dd, *J* = 10.1, 1.6 Hz), 3.08–3.00 (2H, m), 2.35–2.31 (5H, m); ^{13}C NMR (101 MHz, $CDCl_3$) δ 166.6, 157.6, 141.6, 140.7, 136.4, 130.2, 129.7, 129.5, 128.2, 127.1, 122.2, 117.4, 116.2, 30.9, 28.2, 21.5; HRMS (APCI): Found MH^+ 356.1239 $C_{18}H_{17}O_5N_3$ requires 356.1246.

1-(4-*tert*-Butylphenyl)pent-4-en-1-one *O*-(2,4-Dinitrophenyl) Oxime (2f)

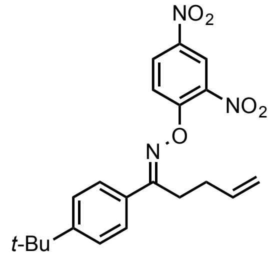

Following **GP8**, **S11** (300 mg, 1.39 mmol) gave **2f** (460 mg, 83%) as an amorphous solid; *E*:*Z* 10:1. R_f 0.86 [petrol–EtOAc (75:25)]; m.p. 83-86 °C; FT-IR ν_{max} (film)/cm^{-1} 1603, 1530, 1471, 1338, 1314, 1258, 1140, 1065; ^{1}H NMR (400 MHz, $CDCl_3$) δ 8.87 (1H, d, *J* = 2.7 Hz), 8.38 (1H, dd, *J* = 9.4, 2.8 Hz), 8.00 (1H, d, *J* = 9.4 Hz), 7.63 (2H, d, *J* = 8.7 Hz), 7.43 (2H, d, *J* = 8.7 Hz), 5.82 (1H, ddt, *J* = 16.9, 10.1, 6.8 Hz), 5.03 (1H, dq, *J* = 17.1, 1.5 Hz), 4.96 (1H, dd, *J* = 10.1, 1.2 Hz), 3.05 (2H, t, *J* = 8.0 Hz), 2.36 (2H, q, *J* = 7.2 Hz), 1.29 (9H, s); ^{13}C NMR (101 MHz, $CDCl_3$) δ 166.58, 157.59, 154.68, 140.72, 136.49, 130.20, 129.46, 128.22, 126.97, 125.94, 122.19, 117.35, 116.14, 53.47, 34.95, 31.17 , 28.31.; HRMS (APCI): Found MH^+ 397.1634 $C_{21}H_{24}O_5N_3$ requires 397.1632.

1-(4-Methoxyphenyl)pent-4-en-1-one *O*-(2,4-Dinitrophenyl) Oxime (2g)

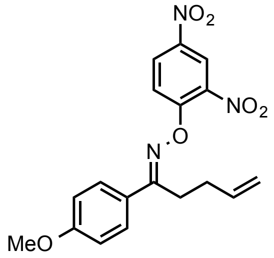

Following **GP8**, **S23** (150 mg, 0.79 mmol) gave **2g** (280 mg, 96%) as an amorphous solid; *E*:*Z* 9:1. R_f 0.73 [petrol–EtOAc (75:25)]; m.p. 93-95 °C; FT-IR ν_{max} (film)/cm^{-1} 1601, 1512, 1469, 1337, 1313, 1254, 1175, 1139, 1024; ^{1}H NMR (400 MHz, $CDCl_3$) δ 8.96 (1H, d, *J* = 2.7 Hz), 8.47 (1H, dd, *J* = 9.4, 2.7 Hz), 8.09 (1H, d, *J* = 9.4 Hz), 7.75 (2H, d, *J* = 9.0 Hz), 7.01 (2H, d, *J* = 9.0 Hz), 5.91 (1H, ddt, *J* = 16.9, 10.2, 6.8 Hz), 5.11 (1H, dq, *J* = 17.1, 1.5 Hz), 5.04 (1H, dd, *J* = 10.2, 1.6 Hz), 3.90 (3H, s), 3.08 (2H, t, *J* = 7.8 Hz), 2.45 (2H, q, *J* = 7.2 Hz); ^{13}C NMR (101 MHz, $CDCl_3$) δ 166.1, 162.0, 157.6, 140.7, 136.5, 129.4, 128.8, 125.3, 122.2, 117.3, 116.13, 114.3, 113.7, 55.5, 31.0, 28.1; HRMS *m/z* (APCI): Found MH^+ 372.1180 $C_{18}H_{17}O_6N_3$ requires 372.1196.

1-(4-Chlorophenyl)pent-4-en-1-one *O*-(2,4-Dinitrophenyl) Oxime (2h)

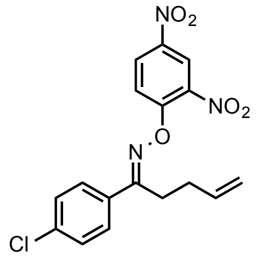

Following **GP8**, **S24** (400 mg, 2.74 mmol) gave **2h** (220 mg, 77%) as an amorphous solid; *E*:*Z* 7:1. R_f 0.81 [petrol–EtOAc (75:25)]; m.p. 82-85 °C; FT-IR ν_{max} (film)/cm^{-1} 1603, 1515, 1493, 1469, 1341, 1313, 1252, 1237, 1139, 1093, 1061, 1012; ^{1}H NMR (400 MHz, $CDCl_3$) δ 8.97 (1H, d, *J* = 2.7 Hz), 8.48 (1H, dd, *J* = 9.4, 2.7 Hz), 8.06 (1H, d, *J* = 9.4 Hz), 7.72 (2H, d, *J* = 8.6 Hz), 7.53–7.45 2H, d, *J* = 8.6 Hz), 5.88 (1H, ddt, *J* = 16.8, 10.1, 6.7 Hz), 5.10 (1H, dd, *J* = 17.1, 1.4 Hz), 5.05 (1H, d, *J* = 10.1 Hz), 3.14 (2H, t, *J* = 7.8 Hz), 2.44 (2H, q, *J* = 7.5 Hz); ^{13}C NMR (101 MHz, $CDCl_3$) δ 165.7, 157.3, 141.0, 137.4, 136.1, 131.6, 129.5, 129.3, 128.8, 128.50, 122.2, 117.3, 116.4, 30.8, 28.2; HRMS *m/z* (APCI): Found: MH$^+$ 376.0698 $C_{17}H_{15}ClO_5N_3$ requires 376. 0700.

1-(4-Fluorophenyl)pent-4-en-1-one O-(2,4-dinitrophenyl) Oxime (2i)

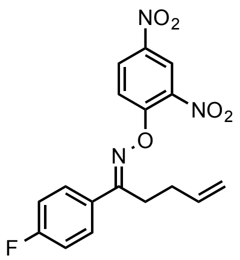

Following **GP8**, **S12** (150 mg, 0.84 mmol) gave **2i** (263 mg, 87%) as an amorphous solid; *E:Z* 4:1. R_f 0.78 [petrol–EtOAc (75:25)]; m.p. 50-90 °C (1st isomer start point and 2nd isomer end point); FT-IR ν_{max} (film)/cm^{-1} 1601, 1524, 1510, 1469, 1338, 1314, 1256, 1236, 1160, 1138, 1065; ^{1}H NMR (400 MHz, $CDCl_3$) δ 8.93 (1H, d, *J* = 2.7 Hz), 8.45 (1H, dd, *J* = 9.4, 2.7 Hz), 8.04 (1H, d, *J* = 9.4 Hz), 7.76 (2H, dd, *J* = 9.0, 5.3 Hz), 7.17 (2H, t, *J* = 8.5 Hz), 5.86 (1H, ddt, *J* = 16.9, 10.1, 6.8 Hz), 5.08 (1H, dq, *J* = 17.0, 1.5 Hz), 5.02 (1H, dq, *J* = 10.1, 1.2 Hz, 1H), 3.12 (t, *J* = 7.8 Hz, 2H), 2.42 (q, *J* = 7.7 Hz, 2H); ^{13}C NMR (101 MHz, $CDCl_3$, *E:Z* isomers) δ 165.7^M, 164.8^m, 164.5^M (d, *J* = 254.4 Hz), 163.6^m (d, *J* = 254.4 Hz), 157.3^M, 157.2^m, 140.9^M, 140.7^m, 136.2^M, 136.0^m, 130.5^m (d, J = 8.9 Hz), 129.5, 129.3^M (d, *J* = 8.3 Hz), 129.25^M (d, *J* = 3.5 Hz), 129.2^m, 122.2^M, 121.9^m, 127.4^m (d, *J* = 3.5 Hz), 117.4^M, 117.3^m, 116.3^M, 116.2^m, 116.1^M (d, *J* = 21.9 Hz), 115.6^m (d, *J* = 21.9 Hz), 34.7^m, 30.8^M, 30.3^m, 28.3^M; ^{19}F NMR (376 MHz, $CDCl_3$, decoupled) δ –108.6; HRMS (APCI): Found MH$^+$ 360.0984 $C_{17}H_{15}FO_5N_3$ requires 360.0984.

1-(2-Fluorophenyl)pent-4-en-1-one O-(2,4-dinitrophenyl) Oxime (2j)

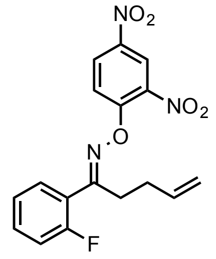

Following **GP8**, **S13** (150 mg, 0.84 mmol) gave **2j** (0.20 g, 66 %) as an amorphous solid; *E:Z* 3:1. R_f 0.73 [petrol–EtOAc (75:25)]; m.p. 77-79 °C; FT-IR ν_{max} (film)/cm^{-1} 1602, 1524, 1443, 1339, 1312, 1263, 1248, 1235, 1209, 1133, 925, 916, 843, 708, 642, 609, 540 ^{1}H NMR (400 MHz, $CDCl_3$) δ 8.87 (0.75H, d, *J* = 2.7 Hz), 8.74 (0.25H, d, *J* = 2.7 Hz), 8.36 (1H, dd, *J* = 9.4, 2.8 Hz), 7.93 (1H, d, *J* = 9.4 Hz), 7.45–7.40 (2H, m), 7.24–7.12 (2H, m), 5.83–5.70 (1H, m), 5.05–4.91 (2H, m), 3.08 (1.5H, t, *J* = 7.3 Hz), 2.80 (0.5H, t, *J* = 6.9 Hz), 2.34–2.29 (m, 2H); ^{19}F NMR (376 MHz, $CDCl_3$, decoupled) δ –111.7^M, –113.3^m; HRMS *m/z* (APCI): Found MH^+ 360.1073 $C_{17}H_{15}O_5N_3F$ requires 360.0996.

1-(Naphthalen-2-yl)pent-4-en-1-one *O*-(2,4-Dinitrophenyl) Oxime (2k)

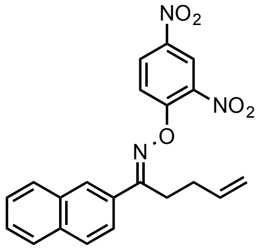

Following **GP8**, **S25** (100 mg, 0.48 mmol) gave **2k** (130 mg, 60%) as an amorphous solid; *E*:*Z* 2:1. R_f 0.31 [petrol–EtOAc (90:10)]; m.p. 134-137 °C; ^{1}H NMR (400 MHz, $CDCl_3$) δ 8.96 (0.66H, d, *J* = 2.4 Hz)^M & 8.82 (0.33H, d, *J* = 2.4 Hz)^m, 8.48 (0.66H, dd, *J* = 9.5, 2.6 Hz)^M & 8.45 (0.33H, dd, *J* = 9.9, 2.5 Hz)^m, 8.17 (0.66H, d, *J* = 21.5 Hz)^M & 8.14 (0.33H, d, *J* = 9.3 Hz)^m, 8.01–7.88 (4H, m), 7.65–7.57 (2H, m), 5.93 (1H, ddt, *J* = 13.2, 9.8, 6.7 Hz), 5.15 (1H, d, *J* = 17.5 Hz), 5.07 (1H, d, *J* = 10.5 Hz), 3.28 (1.33H, t, *J* = 7.8 Hz)^M & 3.03 (0.66H, t, *J* = 7.4 Hz)^m, 2.53 (1.33H, q, *J* = 7.2 Hz)^M and 2.42 (0.66H, q, *J* = 7.0 Hz)^m; ^{13}C NMR (101 MHz, $CDCl_3$, *E:Z* isomers) δ 166.4^M & 165.8^m, 157.5^M & 157.5^m , 140.8^M & 140.7^m , 136.5^M & 136.4^m, 136.1^m & 136.0^M, 134.5^M & 133.9^m, 132.9^M & 132.6^m, 130.5$^{M\ \&\ m}$, 129.6^M & 129.3^m, 128.9^m & 128.9^M, 128.8^M & 128.7^m, 128.1^m & 127.9^M, 127.8^M (2C) & 127.7^m (2C), 127.0^M & 126.9^m, 125.0^m & 123.6^M, 122.3^M & 122.0^m, 117.5^m & 117.4^M, 116.3$^{M\ \&\ m}$, 34.8^m & 31.1^M , 30.5^m & 28.1^M; HRMS (APCI): Found MH^+ 392.1455 $C_{21}H_{24}O_5N_3$: requires 392.1446.

1-(Furan-2-yl)pent-4-en-1-one *O*-(2,4-Dinitrophenyl) Oxime (2l)

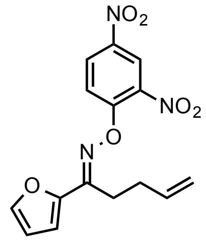

Following **GP8**, **S26** (100 mg, 0.67 mmol) gave **2l** (130 mg, 60%) as an amorphous solid. *Z:E 3:2* R_f 0.31 [petrol–EtOAc (90:10)]; m.p. 75-78 °C; FT-IR ν_{max} (film)/cm^{-1} 3105, 1604, 1536, 1521, 1471, 1338, 1267, 1140, 1064, 1026; ^{1}H NMR (400 MHz, $CDCl_3$) δ 8.92 (1H, d, *J* = 2.5 Hz), 8.47 (1H, dd, *J* = 9.4, 2.5 Hz), 8.09 (1H, d, *J* = 9.4 Hz), 7.61 (1H, d, *J* = 0.7 Hz), 6.99 (1H, d, *J* = 3.3 Hz), 6.61–6.56 (1H, m), 5.89 (1H, ddt, *J* = 16.9, 10.1, 6.8 Hz), 5.09 (1H, dd, *J* = 17.0, 0.9 Hz), 5.02 (1H, d, *J* = 10.1 Hz), 3.02 (2H, t, *J* = 7.8 Hz), 2.48 (2H, q, *J* = 7.3 Hz); ^{13}C NMR (101 MHz, $CDCl_3$) δ 157.8, 157.3, 147.2, 145.7, 140.8, 136.3, 135.9, 129.6, 122.2, 117.4, 116.3, 114.5, 112.2, 31.2, 27.8; HRMS (APCI): Found MH$^+$ 332.0864 $C_{21}H_{24}O_5N_3$: requires 332.0883.

1-(Pyridin-3-yl)pent-4-en-1-one O-(2,4-dinitrophenyl) Oxime (2m)

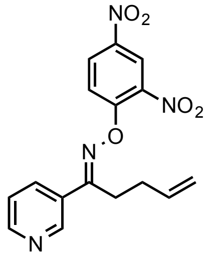

Following **GP8**, **S31** (150 mg, 0.93 mmol) gave **2m** (110 mg, 35%) as an amorphous solid. R_f 0.16 [petrol–EtOAc (90:10)]; m.p. 99-101 °C; FT-IR ν_{max} (film)/cm^{-1} 3111, 2984, 1597, 1516, 1470, 1411, 1338, 1261, 1135, 1066, 1026; ^{1}H NMR (500 MHz, $CDCl_3$) δ 8.99 (1H, d, *J* = 1.7 Hz), 8.95 (1H, d, *J* = 2.7 Hz), 8.75 (1H, dd, *J* = 4.8, 1.6 Hz), 8.47 (1H, dd, *J* = 9.4, 2.7 Hz), 8.08–8.03 (2H, m), 7.44 (1H, ddd, *J* = 8.0, 4.8, 0.8 Hz), 5.85 (1H, ddt, *J* = 16.9, 10.1, 6.8 Hz), 5.08 (1H, dq, *J* = 17.0, 1.5 Hz), 5.03 (1H, dq, *J* = 10.2, 1.0, 1.0 Hz), 3.16 (2H, t, *J* = 7.7 Hz), 2.47–2.42 (2H, m); ^{13}C NMR (101 MHz, $CDCl_3$) δ 164.7, 157.1, 151.8, 148.3, 135.9, 134.5, 129.5, 123.7, 122.2, 117.3, 116.7, 30.6, 28.1; HRMS (APCI): Found MH$^+$ 343.1033 $C_{16}H_{15}O_5N_4$: requires 343.1042

1-Phenylhept-6-en-3-one *O*-(2,4-Dinitrophenyl) Oxime (2n)

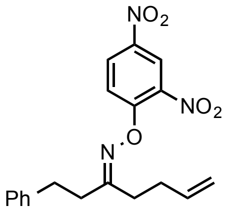

Following **GP8**, **S27** (166 mg, 0.882 mmol) gave **2n** (290 mg, 89%) as an amorphous solid; *E*:*Z* 3:2. m.p. 48-64 °C (1st isomer start point and 2nd isomer end point); ^{1}H NMR (400 MHz, $CDCl_3$, *E:Z* isomers) δ 8.9 (0.6H, d, *J* = 2.5 Hz), 8.9 (0.4H, d, *J* = 2.6 Hz), 8.40 (0.6H, dd, *J* = 9.4, 2.8 Hz), 8.37 (0.4H, dd, *J* = 9.4, 2.8 Hz), 7.89 (0.6H, d, *J* = 9.4 Hz), 7.74 (0.4H, d, *J* = 9.4 Hz), 7.35–7.29 (1H, m), 7.28–7.14 (4H, m), 5.84 (1H, ddt, *J* = 12.7, 10.4, 6.4 Hz), 5.13 (1H, dq, *J* = 17.4, 1.6, 1.6 Hz), 5.08 (1H, dq, *J* = 10.2, 1.2 Hz), 2.82 (3H, m), 2.77–2.65 (2H, m), 2.47–2.35 (3H, m); ^{13}C NMR (101 MHz, $CDCl_3$) δ 169.1^m, 169.0^M, 157.1^M,157.5^m, 140.5^m, 140.5^M, 140.4^m, 140.1^M, 136.6^M, 136.4^m, 135.8^m, 135.7^M, 129.4^M, 129.4^m, 128.7^m, 128.6^M, 128.4^M & 128.3^m, 126.5^{M,} 126.5^m, 122.1^M; 122.1^m, 117.3^m, 117.2^M, 116.3^m, 116.1^M, 33.9^M, 32.6^m, 32.1^M, 31.9^m, 30.0^M, 29.9^m, 35.9^m, 29.7^M; HRMS (APCI): Found MH^+ 370 .1466 $C_{19}H_{18}O_5N_3$ requires 370.1403. Data in accordance with the literature.[29]

Ethyl 5-((2,4-Dinitrophenoxy)imino)non-8-enoate (2o)

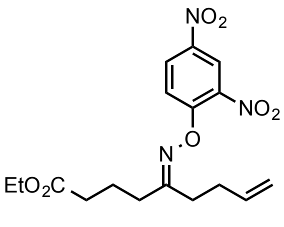

Following **GP8** but purifying the product by column chromatography, **S14** (200 mg, 1.01 mmol) gave **2o** (299 mg, 78%) as an amorphous solid; *E*:*Z* 1:1. R$_f$ 0.81 [petrol–EtOAc (75:25)]; ^{1}H NMR (400 MHz, $CDCl_3$, *E:Z* isomers) δ 8.93 (0.5H, t, *J* = 2.8 Hz), 8.92 (0.5H, t, *J* = 2.8 Hz), 8.45 (0.5H, dd, *J* = 2.8, 1.1 Hz), 8.43 (0.5H, dd, *J* = 2.8, 1.1 Hz), 7.97 (0.5H, d, *J* = 2.7 Hz), 7.95 (0.5H, d, *J* = 2.7 Hz), 5.91–5.80 (1H, m), 5.17–5.03 (2H, m), 4.17 (1H, q, *J* = 7.1 Hz), 4.16 (1H, q, *J* = 7.1 Hz), 2.71–2.62 (2H, m), 2.57–2.33 (6H, m), 2.05–1.90 (m, 2H), 1.29 (1.5H, t, *J* = 7.1 Hz), 1.27 (1.5H, t, *J* = 7.1 Hz); ^{13}C NMR (101 MHz, $CDCl_3$) δ 172.9 & 172.85, 169.15 & 169.1, 157.55 & 157.49, 140.5, 136.5 & 136.3, 129.5 & 129.45, 122.25 & 122.2, 117.25 & 117.2, 116.3 & 116.1, 60.65 & 60.6, 33.8 & 33.6, 33.45 & 33.4, 30.1, 30.0 & 29.5, 21.2 & 20.9, 14.3 & 14.25; HRMS (APCI): Found MH^+ 380.1456 $C_{17}H_{22}N_3O_7$ requires 380.1458.

2-Allylcyclohexan-1-one O-(2,4-dinitrophenyl) oxime (2q)

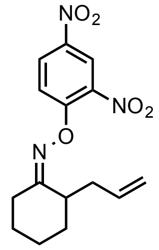

Following **GP8**, 2-allylcyclohexan-1-one (200 mg, 1.49 mmol) gave **2q** (377 mg, 82%) as an amorphous solid. R_f 0.51 [petrol–EtOAc (90:10)]; m.p. 89-104 °C (1st isomer start point and 2nd isomer end point); FT-IR ν_{max} (film)/cm^{-1} 3091, 2945, 1600, 1521, 1471, 1346, 1260, 1141, 1064, 908, 846, 825, 742, 719; ^{1}H NMR (400 MHz, $CDCl_3$, *E:Z* isomers) δ 8.91 (1H, d, *J* = 2.8 Hz), 8.44 (1H, dd, *J* = 9.4, 2.8 Hz), 7.98 (1H, d, *J* = 9.4 Hz), 5.91–5.81 (1H, m), 5.16–5.08 (2H, m), 3.16–3.10 (1H, m), 2.63 (1H, dtt, *J* = 14.1, 6.4, 1.4 Hz), 2.56–2.49 (1H, m), 2.45–2.38 (1H, m), 2.32–2.23 (1H, m), 2.06–2.00 (1H, m), 1.91–1.83 (2H, m), 1.73–1.64 (1H, m), 1.58–1.50 (2H, m); ^{13}C NMR (101 MHz, $CDCl_3$, *E:Z* isomers) δ 171.4^m, 170.8^M, 157.9^M, 157.7^m, 140.3^m, 136.0^M, 135.8^m, 135.2^M, 129.4^M, 129.3^m, 122.1, 117.3^M, 117.2^m, 117.0^m, 116.8^M, 42.4, 35.4^m, 35.2^M, 34.9^m, 32.8^M, 29.3^m, 28.3^m, 26.6^m, 26.5^M, 26.4^M, 23.7^M, 20.1^m; HRMS (APCI): Found MH^+ 320.1251 $C_{15}H_{18}O_5N_3$ requires 321.1246.

***tert*-Butyl 3-Allyl-4-((2,4-dinitrophenoxy)imino)piperidine-1-carboxylate (2r)**

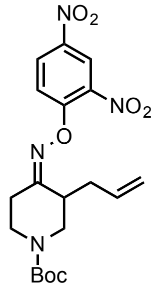

Following **GP8** but purifying the product by column chromatography, **S31** (50 mg, 0.21 mmol) gave **2r** (70 mg, 80%) as an amorphous solid; *E*:*Z* 3:1. R_f 0.64 [petrol–EtOAc (90:10)]; ^{1}H NMR (400 MHz, $CDCl_3$, *E* and *Z* isomer, rotamers) δ 8.82 (1H, t, *J* = 3.0 Hz), 8.35 (1H, dt, *J* = 9.4, 2.7 Hz), 7.88 (1H, dd, *J* = 9.4, 4.0 Hz, 1H), 5.81–5.68 (1H, m), 5.11–4.98 (2H, m), 4.39–4.10 (0.5H, m), 3.91–3.68 (1H, m), 3.64–3.37 (2H, m), 3.01–2.64 (2H, m), 2.61–2.58 (1H, m), 2.53–2.36 (1H, m), 2.31–2.22 (1H, m), 1.42 (9H, s); ^{13}C NMR (101 MHz, $CDCl_3$, *E* and *Z* isomer, rotamers) δ 167.5, 157.3, 154.5, 140.7, 135.9^m, 134.6^M, 134.2^m, 129.4^M, 122.1, 118.1^m, 117.9^M, 117.2^M, 117.1^m, 80.5, 47.5 (br), 41.3, 33.6 (br), 28.4.

1-(2-Phenylcyclopropyl)pent-4-en-1-one O-(2,4-dinitrophenyl) Oxime (2s)

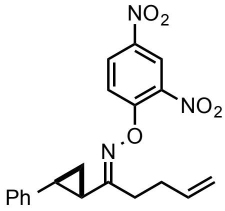

Following **GP8** but purifying the product by column chromatography, **S28** (264 mg, 1.32 mmol) gave **2s** (347 mg, 69%) as an amorphous solid; *E*:*Z* 1:1. R_f 0.64 [petrol–EtOAc (90:10)]; FT-IR ν_{max} (film)/cm^{-1} 3089, 1641, 1602, 1519, 1499, 1471, 1338, 1313, 1274, 1260, 1141, 1094, 1063; ^{1}H NMR (400 MHz, CDCl$_3$) δ 8.94 (0.5H, d, *J* = 2.8 Hz), 8.92 (0.5H, d, *J* = 2.8 Hz), 8.33 (0.5H, dd, *J* = 9.4, 2.6 Hz), 8.30 (0.5H, dd, *J* = 9.4, 2.6 Hz), 7.87 (0.5H, d, *J* = 9.4 Hz), 7.84 (0.5H, d, *J* = 9.4 Hz), 7.37–7.33 (2H, m), 7.27–7.16 (3H, m), 5.97–5.83 (1H, m, 1H), 5.19–5.04 (2H, m), 3.00 (0.5H, ddd, *J* = 8.9, 6.0, 4.9 Hz), 2.81–2.65 (1.5H, m), 2.52–2.40 (3H, m), 2.29–2.25 (1H, m), 1.92 (0.5H, ddd, *J* = 8.5, 5.8, 4.5 Hz), 1.72 (0.5H, ddd, *J* = 9.0, 5.7, 5.2 Hz), 1.58-1.43 (m, 2H); ^{13}C NMR (101 MHz, CDCl$_3$, *E* and *Z* isomer) δ 170.0 & 167.9, 157.8 & 157.5, 140.5 & 140.4, 140.3 & 139.7, 136.7 & 136.5, 135.8 & 135.7, 129.5 & 129.4, 128.7 & 128.65, 126.8 & 126.6, 126.2 & 126.0, 122.2 & 122.15, 117.2 & 117.1, 116.3 & 116.0, 30.4 & 30.2, 30.0 & 29.0, 26.6 & 25.7, 24.6 & 21.4, 16.2 & 15.9; HRMS (APCI): Found MH$^+$ 382.14.05 $C_{20}H_{20}N_3O_5$ requires 382.1403.

***tert*-Butyl 4-(1-((2,4-Dinitrophenoxy)imino)pent-4-en-1-yl)piperidine-1-carboxylate (2t)**

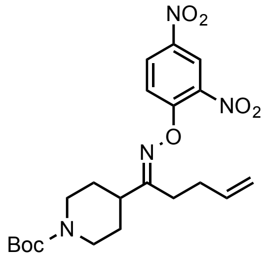

Following **GP8** but purifying the product by column chromatography, *tert*-butyl 4-(pent-4-enoyl)piperidine-1-carboxylate (150 mg, 0.56 mmol) gave **2t** (244 mg, 97%) as an amorphous solid; *E*:*Z* 3:1. R_f 0.64 [petrol–EtOAc (90:10)]; ^{1}H NMR (400 MHz, CDCl$_3$, *E* and *Z* isomer) δ 8.83 (0.3H, d, *J* = 2.8 Hz), 8.81 (0.7H, d, *J* = 2.7 Hz), 8.36 (0.3H, dd, 9.4, 2.8 Hz), 8.34 (0.7H, dd, *J* = 9.4, 2.7 Hz), 7.87 (0.3H, d, *J* = 9.4 Hz), 7.84 (0.7H, d, *J* = 9.4 Hz), 5.85–5.69 (1H, m), 5.07–4.88 (2H, m), 4.16 (2H, br s), 2.73 (2H, br t, *J* = 11.6 Hz), 2.56 (1H, dd, *J* = 9.1, 6.7 Hz), 2.50–2.23 (4H, m), 1.82 (1H, br d, *J* = 14.4 Hz), 1.72 (1H, br d, *J* = 13.2 Hz), 1.61–1.48 (2H, m), 1.40 (9H, s).

2-(4-Isopropylphenyl)hept-6-en-3-one O-(2,4-Dinitrophenyl) Oxime (2u)

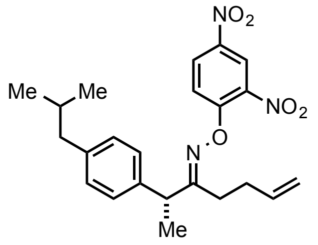

Following **GP8** but purifying the product by column chromatography, **S29** (127 mg, 0.52 mmol) gave **2u** (179 mg, 81%) as an amorphous solid; *E*:*Z* 1.5:1. R_f 0.59 [petrol–EtOAc (90:10)]; FT-IR ν_{max} (film)/cm^{-1} 2954, 1603, 1527, 1471, 1339, 1314, 1274, 1142, 1064; ^{1}H NMR (400 MHz, $CDCl_3$, *E* and *Z* isomer) δ 8.84 (0.6H, d, *J* = 2.6 Hz), 8.83 (0.4H, d, *J* = 2.7 Hz), 8.38 (0.4H, dd, *J* = 9.4, 2.7 Hz), 8.36 (0.6H, d, *J* = 9.4, 2.7 Hz), 7.95 (0.4H, d, *J* = 9.4 Hz), 7.89 (0.6H, d, *J* = 9.4 Hz), 7.14–7.08 (2H, m), 7.06–7.02 (2H, m), 5.74–5.61 (1H, m), 4.94–4.85 (2H, m), 2.59 (0.4H, ddd, *J* = 12.4, 9.0, 6.8 Hz), 2.38 (2H, t, *J* = 7.7 Hz), 2.32–2.30 (0.6H, m), 2.26–2.14 (2H, m), 2.11–2.04 (1H, m), 1.77 (1H, sept, *J* = 6.5 Hz), 1.48 (1.2H, d, *J* = 7.0 Hz), 1.47 (1.8H, d, *J* = 7.3 Hz), 0.82 (2.4H, d, *J* = 6.5 Hz), 0.81 (3.6H, d, *J* = 6.6 Hz); ^{13}C NMR (101 MHz, $CDCl_3$, diastereomers) δ 171.6, 171.1, 157.7, 157.6, 141.1, 140.8, 140.6, 137.6, 137.1, 136.8, 136.7, 136.0, 129.7, 129.6, 129.5, 129.4, 127.5, 127.4, 122.15, 122.1, 117.4, 117.3, 115.8, 115.5, 45.2, 45.0, 38.4, 30.6, 30.25, 30.2, 30.1, 29.6, 29.5, 22.4, 18.4, 16.0; HRMS (APCI): Found MH$^+$ 426.2021 $C_{23}H_{28}N_3O_5$ requires 426.2029.

Adamantan-1-yl)pent-4-en-1-one *O*-(2,4-Dinitrophenyl) Oxime (2v)

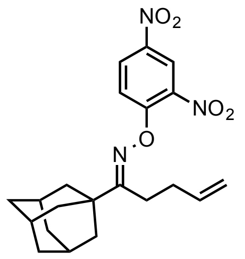

Following **GP8** but leaving the reaction for 3 days, **S15** (150 mg, 0.69 mmol) gave **2v** (70 mg, 37%) as an amorphous solid. R_f 0.62 [petrol–EtOAc (90:10)]; m.p. 113-117 °C; FT-IR ν_{max} (film)/cm^{-1} 2904, 2852, 1600, 1520, 1468, 1451, 1339, 1311, 1274, 1232, 1134, 1064; ^{1}H NMR (400 MHz, $CDCl_3$) δ 8.82 (1H, d, *J* = 2.6 Hz), 8.34 (1H, dd, *J* = 9.4, 2.7 Hz), 7.88 (1H, d, *J* = 9.4 Hz), 5.85 (1H, ddt, *J* = 16.8, 10.2, 6.6 Hz), 5.06 (1H, d, *J* = 17.1 Hz), 4.97 (1H, d, *J* = 10.1 Hz), 2.48 (2H, m), 2.27 (2H, q, *J* = 8.4 Hz), 2.10 (3H, br s), 1.89 (3H, s), 1.88 (3H, s), 1.81 (1H, br s), 1.78 (2H, br s), 1.74 (2H, br s), 1.71 (1H, br s); ^{13}C NMR (101 MHz, $CDCl_3$) δ 175.0, 158.0, 140.3, 137.2, 135.8, 129.4, 122.2, 117.3, 115.6, 40.8, 39.2, 36.5, 31.0, 27.9, 26.4; HRMS (APCI): Found MH$^+$ 320.1251 $C_{15}H_{13}ON_2$: requires 320.1246.

(4*E*)-1,5-Diphenylpent-4-en-1-one *O*-(2,4-Dinitrophenyl) Oxime (2w)

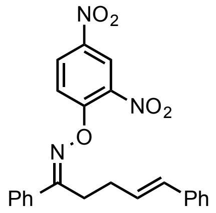

Following **GP8** but purifying the product by column chromatography, **S34** (126 mg, 1.49 mmol) gave **2w** (164 mg, 74%) was obtained as a yellow resin; *E:Z* 10:1. R_f 0.64 [petrol–EtOAc (90:10)]; m.p. 105-114 °C (start point of 1st isomer and end point of 2nd isomer); FT-IR ν_{max} (film)/cm^{-1} 3123, 3024, 2927, 1602, 1521, 1493, 1468, 1455, 1444, 1338, 1313, 1260, 1235, 1133, 1062; ^{1}H NMR (400 MHz, $CDCl_3$) δ 8.75 (1H, d, *J* = 2.6 Hz), 8.24 (1H, dd, *J* = 9.4, 2.8 Hz), 7.88 (1H, d, *J* = 9.4 Hz), 7.68 (2H, dd, *J* = 8.2, 1.5 Hz), 7.44–7.37 (m, 3H), 7.13–7.04 (m, 5H), 6.28 (1H, dt, *J* = 15.8, 1.1 Hz), 6.14 (1H, dt, *J* = 15.8, 7.0 Hz), 3.12 (2H, br t, *J* = 7.6 Hz, 2H), 2.48 (2H, q, *J* = 7.1 Hz); ^{13}C NMR (101 MHz, $CDCl_3$) δ 166.7, 157.5, 140.7, 137.1, 133.2, 131.5, 131.2, 129.4, 129.0, 128.5, 128.1, 127.27, 127.21, 126.0, 122.2, 117.3, 30.4, 28.8; HRMS (APCI): Found MH^+ 418.1403 $C_{23}H_{20}N_3O_5$: requires 418.1403.

(*E*)-2-(Cyclohex-2-en-1-yl)-1-phenylethan-1-one *O*-(2,4-Dinitrophenyl) Oxime (2y)

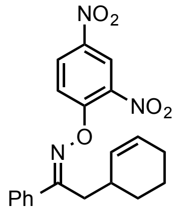

Following **GP8** but purifying the product by column chromatography, **S35** (150 mg, 0.75 mmol) gave **2y** (224 mg, 78%) as a yellow resin. R_f 0.44 [petrol–EtOAc (90:10)]; m.p. 101-108 °C; FT-IR ν_{max} (film)/cm^{-1} 2922, 1602, 1525, 1471, 1335, 1313, 1258, 1140, 1132, 1064; ^{1}H NMR (400 MHz, $CDCl_3$) δ 8.92 (1H, d, *J* = 2.7 Hz), 8.46 (1H, dd, *J* = 9.4, 2.6 Hz), 8.10 (1H, d, *J* = 9.4 Hz), 7.81–7.78 (2H, m), 7.58–7.48 (3H, m), 5.72 (1H, br dd, *J* = 9.9, 3.0 Hz), 5.55 (1H, br dd, *J* = 10.1, 2.0 Hz), 3.19–3.05 (2H, m), 2.59 (1H, br s), 1.99 (2H, br s), 1.85–1.70 (2H, m), 1.58–1.44 (1H, m), 1.43–1.33 (1H, m); ^{13}C NMR (101 MHz, $CDCl_3$) δ 166.6, 157.5, 140.8, 136.0, 133.7, 131.0, 129.7, 129.5, 128.9, 128.6, 127.4, 122.2, 117.4, 34.7, 33.6, 28.9, 25.0, 20.9; HRMS (APCI): Found MH^+ 382.1387 $C_{20}H_{19}O_5N_3$ requires 382.1403.

(2Z,5E)-6,10-dimethylundeca-5,9-dien-2-one O-(2,4-dinitrophenyl) oxime (2z)

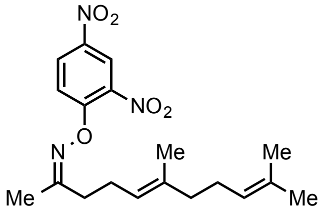

Following **GP8** but purifying the product by column chromatography, (*E*)-6,10-dimethylundeca-5,9-dien-2-one (126 mg, 1.49 mmol) gave **2z** (180 mg, 99%) as an oil; *E*:*Z* 1:1. R_f 0.56 (M), 0.46 (m) [petrol–EtOAc (90:10)]; FT-IR ν_{max} (film)/cm^{-1} 2916, 1604, 1472, 1339, 1289, 1237, 1142, 1064; ^{1}H NMR (400 MHz, $CDCl_3$, *E:Z* isomers) δ 8.87 (0.5H, d, *J* = 2.9 Hz), 8.86 (0.5H, d, *J* = 3.1 Hz), 8.41 (0.5H, dd, *J* = 9.2 2.7 Hz), 8.39 (0.5H, dd, *J* = 9.1, 2.7 Hz) 7.95 (0.5H, m), 7.93 (0.5H, m), 5.15 (1H, dt, *J* = 7.0, 1.1 Hz), 5.12–5.03 (1H, m), 2.46-2.41 (2H, m), 2.37-2.31 (2H, m), 2.18 & 2.17 (3H, s), 2.11–2.07 (3H, m), 2.06-1.95 (2H, m), 1.75-1.55 (11H, m); ^{13}C NMR (101 MHz, $CDCl_3$, *E:Z* isomers) δ 167.9, 167.8, 167.1, 167.0, 157.6, 157.5, 140.4, 137.25, 137.2, 137.1, 137.0, 135.85, 131.8, 131.7, 131.55, 131.5, 129.3, 124.1, 124.05, 124.0, 122.95, 122.9, 122.1, 122.05, 122.0, 117.25, 117.2, 117.1, 39.65, 39.6, 35.9, 35.6, 32.0, 31.9, 31.3, 31.1, 26.6, 26.55, 26.5 (x 2), 25.75, 25.7, 25.6; HRMS (APCI): Found MH^+ 376.1873 $C_{19}H_{25}N_3O_5$ requires 376.1872.

3 Electrochemical studies

3.1 General Experimental Detail

Cyclic voltammetry was conducted on an Autolab PGSTAT100 (Metrohm) using a 3-electrode cell configuration. A glassy carbon working electrode was employed alongside a platinum flag counter electrode and a silver pseudo-reference electrode. Ferrocene solution was added as an internal standard to determine the precise potential scale.[30] These values were converted to a saturated calomel electrode (SCE) scale. 5 mM oxime solutions were freshly prepared in acetonitrile along with 0.1 M supporting electrolyte (tetrabutylammonium tetrafluoroborate). Nitrogen was passed through the sample between measurements to avoid the deleterious influence of oxygen reduction, either directly or through indirect reaction with the oxime-derived species. Samples were examined at 8 different scan rates 0.05 V s^{-1} – 2.00 V s^{-1}. The fragmented species reacted too rapidly to produce an oxidation peak on reversal of the potential sweep direction for all except oxime **c** in Scheme 3. Without the presence of an oxidation peak, or other kinetic information about the species formed, the formal reduction potential can only be estimated to within ~50 mV. As a result, we have used the $E_p$max (potential corresponding to the maximum reductive current in the voltammogram from the fastest scan-rate, 2 V s^{-1}).[31]

3.2 Electrochemical potentials

Oxime	$E_{1,2}^{red}$ (V) vs SCE	Oxime	$E_{1,2}^{red}$ (V) vs SCE
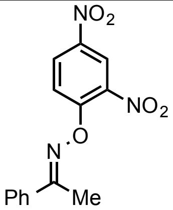 **1a**	–0.55	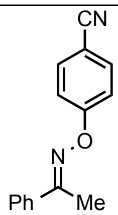 **1e**	–1.70
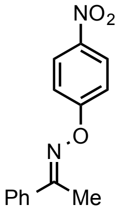 **1b**	–0.65	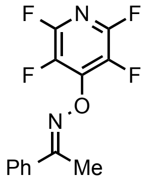 **S7**	– 1.75
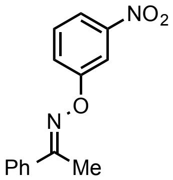 **1c**	–0.67	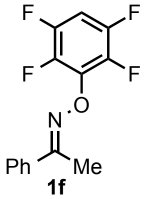 **1f**	–1.79
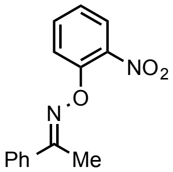 **1d**	–0.93	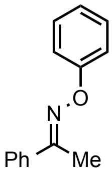 **1g**	–1.87

3.3 Cyclic voltammograms

1a

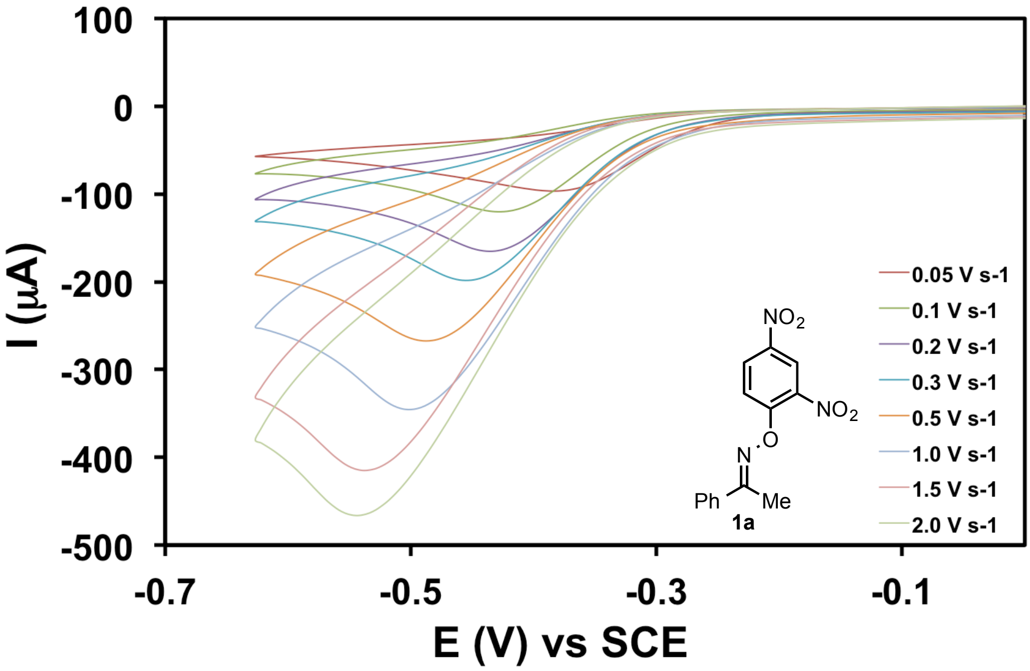

1b

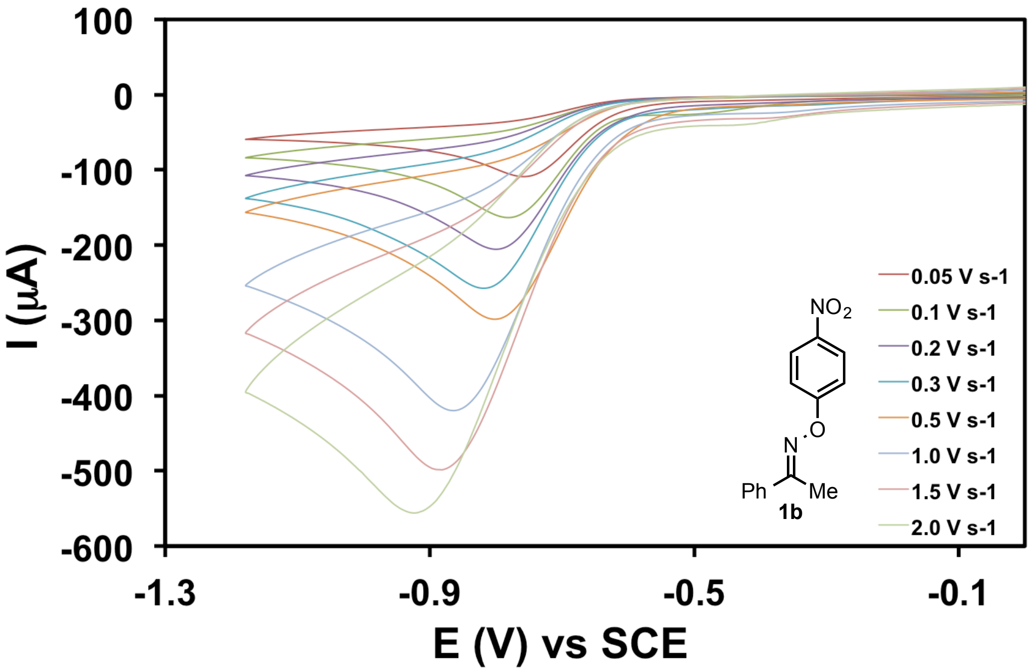

1c

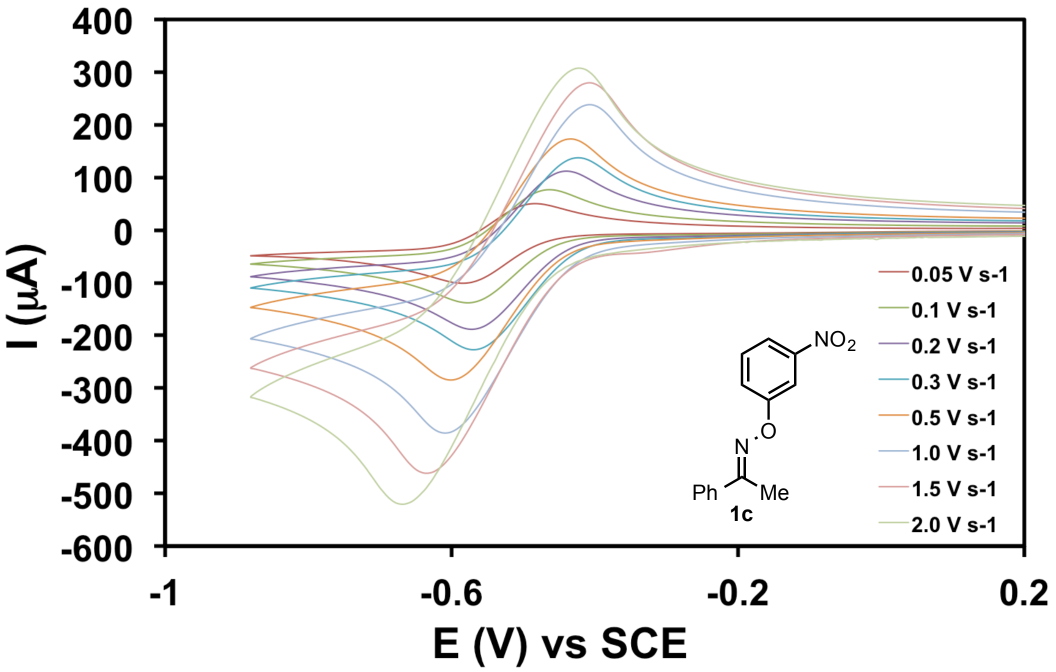
400
300
200
100
0
-100
-200
-300
-400
-500
-600
I (μA)
-1
-0.6
-0.2
0.2
E (V) vs SCE
0.05 V s-1
0.1 V s-1
0.2 V s-1
0.3 V s-1
0.5 V s-1
1.0 V s-1
1.5 V s-1
2.0 V s-1
NO2
O
N
Ph
Me
1c

1d

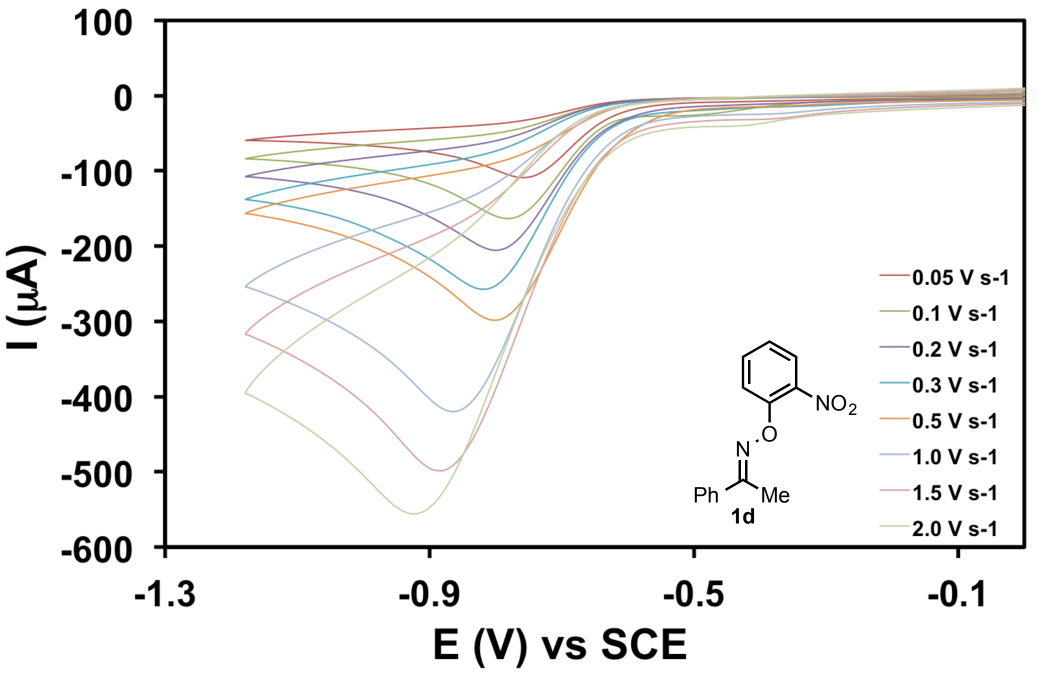
100
0
-100
-200
-300
-400
-500
-600
I (μA)
-1.3
-0.9
-0.5
-0.1
E (V) vs SCE
0.05 V s-1
0.1 V s-1
0.2 V s-1
0.3 V s-1
0.5 V s-1
1.0 V s-1
1.5 V s-1
2.0 V s-1
NO2
O
N
Ph
Me
1d

1e

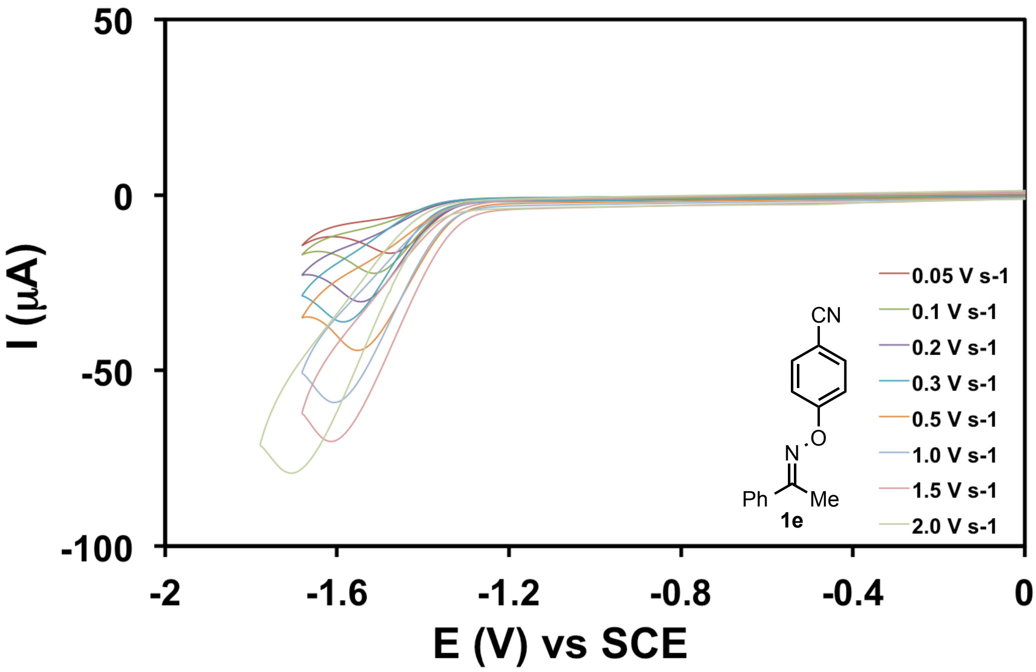

50
0
-50
-100
I (μA)
-2
-1.6
-1.2
-0.8
-0.4
0
E (V) vs SCE
0.05 V s-1
0.1 V s-1
0.2 V s-1
0.3 V s-1
0.5 V s-1
1.0 V s-1
1.5 V s-1
2.0 V s-1
CN
O
N
Ph
Me
1e

S7

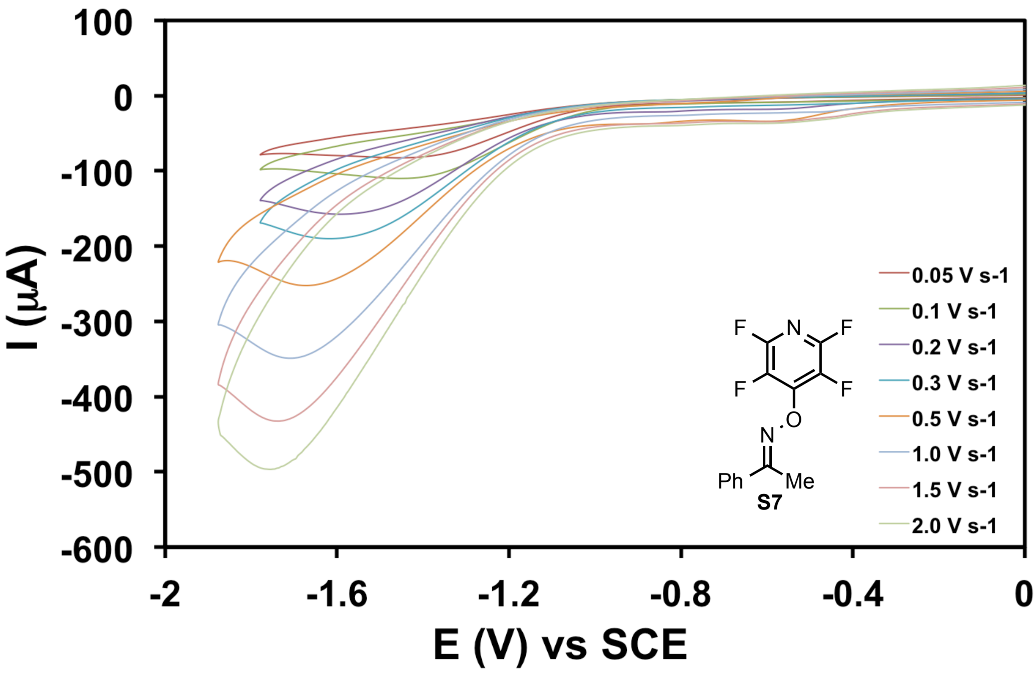

100
0
-100
-200
-300
-400
-500
-600
I (μA)
-2
-1.6
-1.2
-0.8
-0.4
0
E (V) vs SCE
0.05 V s-1
0.1 V s-1
0.2 V s-1
0.3 V s-1
0.5 V s-1
1.0 V s-1
1.5 V s-1
2.0 V s-1
F
N
F
F
F
O
N
Ph
Me
S7

1f

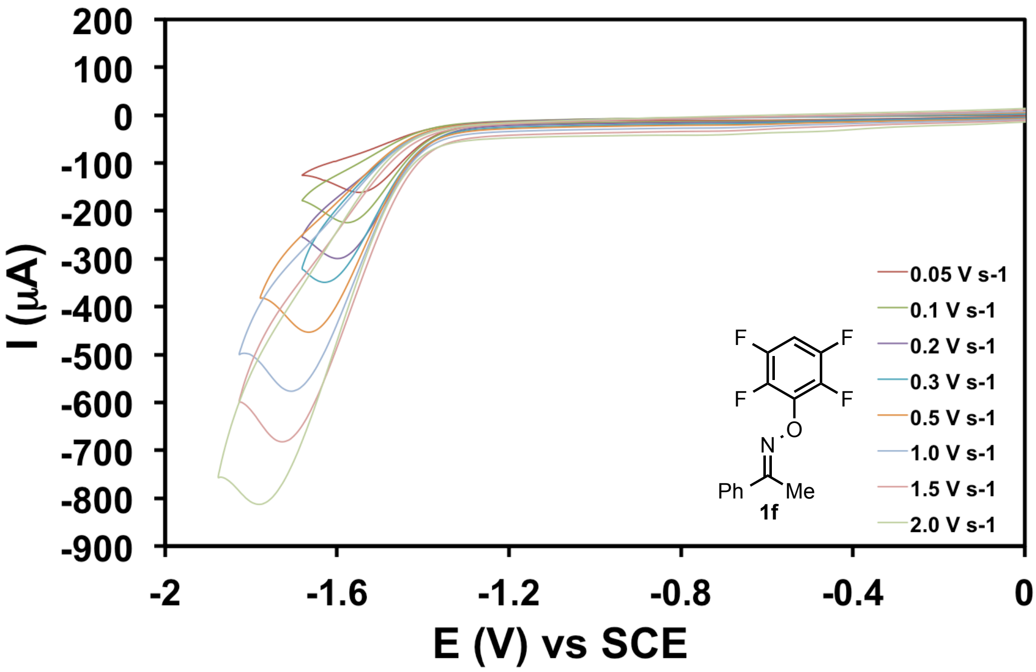
200
100
0
-100
-200
-300
-400
-500
-600
-700
-800
-900
I (μA)
-2
-1.6
-1.2
-0.8
-0.4
0
E (V) vs SCE
0.05 V s-1
0.1 V s-1
0.2 V s-1
0.3 V s-1
0.5 V s-1
1.0 V s-1
1.5 V s-1
2.0 V s-1
F
F
F
F
O
N
Ph
Me
1f

1g

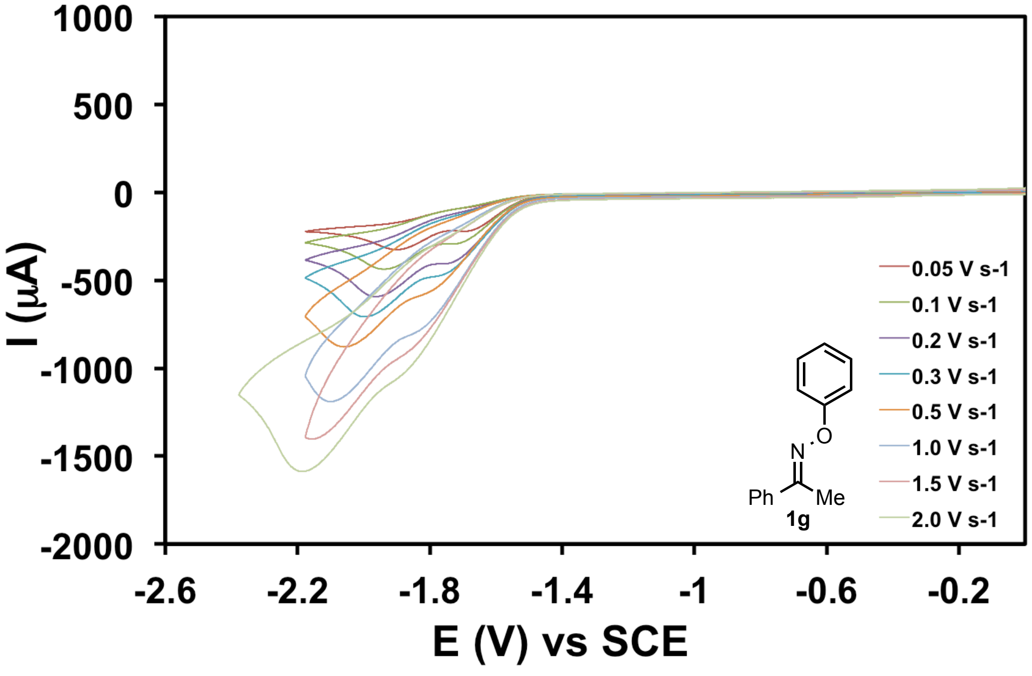
1000
500
0
-500
-1000
-1500
-2000
I (μA)
-2.6
-2.2
-1.8
-1.4
-1
-0.6
-0.2
E (V) vs SCE
0.05 V s-1
0.1 V s-1
0.2 V s-1
0.3 V s-1
0.5 V s-1
1.0 V s-1
1.5 V s-1
2.0 V s-1
O
N
Ph
Me
1g

4 Hydroimination-cyclization

4.1 Reaction optimization with oxime 2a

General Procedure for the reaction optimization using oxime 2a – GP9

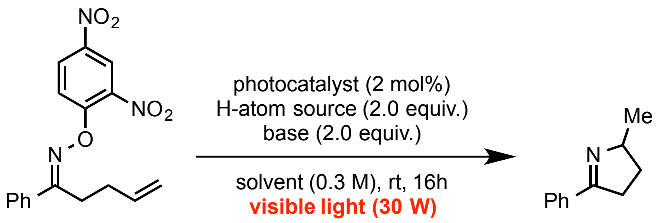

A dry tube equipped with a stirring bar was charged with **2a** (30 mg, 0.088 mmol, 1.0 equiv.), the photocatalyst (1.76 μmol, 2 mol%), the base (0.176 mmol, 2.0 equiv.), the H-source (0.176 mmol, 2.0 equiv.) and the solvent (0.3 M). The mixture was stirred at room temperature overnight in front of a 30 W fluorescent household bulb. H_2O (2 mL) and EtOAc (2 mL) were added. The layers were separated and the aqueous layer was extracted with EtOAc (2 x 2 mL). The combined organic layers were dried ($MgSO_4$), filtered and evaporated. 1,3,5-Trimethoxybenzene (4.9 mg, 0.029 mmol, 0.33 equiv.) and $CDCl_3$ (0.4 mL) were added and the mixture was analysed by ^1H NMR spectroscopy to determine the NMR yield.

Entry	Photocatalyst	H-Source	Base	Solvent	Yield (%)
**1**	Ir(ppy)$_3$	**HD1**	–	CH_3CN	20
**2**		**HD2**	–	CH_3CN	traces
**3**		CHD	–	CH_3CN	22
**4**		CHD	K_2CO_3	HFIP	61
**5**[a]		CHD	–	HFIP	traces
**6**	no photocatalyst	CHD	–	HFIP	traces
**7**		–	–	HFIP	traces
**8**	no photocatalyst	–	–	HFIP	–
**9**		CHD	$CsCO_3$	HFIP	82
**10**		CHD	CsOAc	HFIP	82
**11**		CHD	K_2CO_3	MeOH	50
**12**		CHD	K_2CO_3	acetone	78
**13**		CHD	K_2CO_3	toluene	traces
**14**		CHD	K_2CO_3	DMF	97
**15**		CHD	K_2CO_3	*i*-PrOH	53
**16**		CHD	K_2CO_3	$CHCl_3$	57
**17**	Cu(dap)$_2$Cl	CHD	K_2CO_3	HFIP	47
**18**	eosin Y	CHD	–	HFIP	64
**19**		CHD	K_2CO_3	HFIP	89
**20**	no photocatalyst	CHD	K_2CO_3	HFIP	traces
**21**		CHD	K_2CO_3	MeOH	73
**22**		CHD	K_2CO_3	acetone	84
**23**		CHD	K_2CO_3	toluene	traces
**24**		CHD	K_2CO_3	DMF	79
**25**		CHD	K_2CO_3	*i*-PrOH	83
**26**		CHD	K_2CO_3	$CHCl_3$	traces

a) the reaction was run in the dark

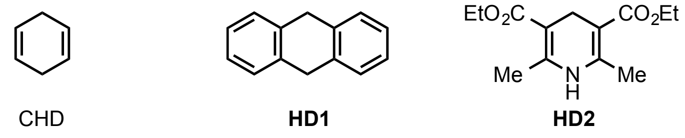

Under the optimized reaction conditions (entry 22), solvent evaporation and analysis of the crude by 1H NMR spectroscopy provided the following spectrum. The potassium 2,4-$(NO_2)_2$-phenoxide is not soluble in $CDCl_3$ and is not present in the sample.

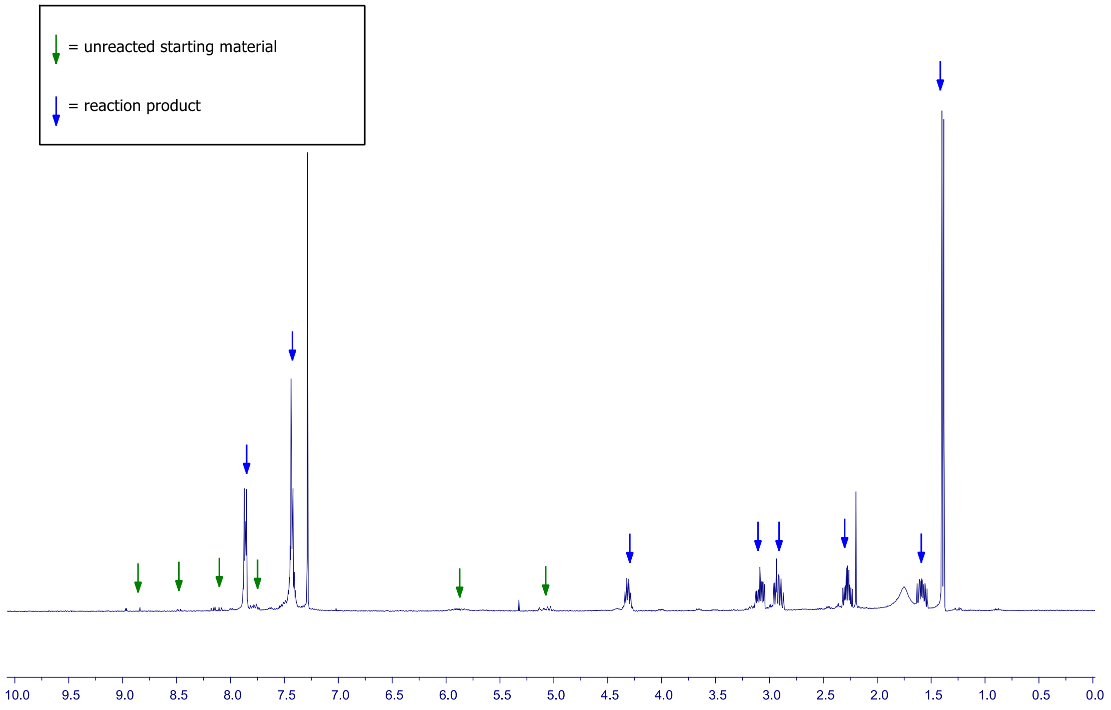

In order to obtain a cleaner product an acid-base wash was performed.

Upon completion of the reaction the acetone was evaporated and the crude was taken up with CH_2Cl_2 and H_2O. HCl was added until pH = 1. The layers were separated and the aqueous layer was washed with CH_2Cl_2 (x 2). The combined organic layers were dried ($MgSO_4$), filtered and evaporated to give the 2,4-$(NO_2)_2$-phenol and unreacted starting material. The aqueous layer was treated with NH_4OH until pH = 14 and extracted with CH_2Cl_2 (x 3). The combined organic layers were dried ($MgSO_4$), filtered and evaporated to give **2a**.

^{1}H NMR of the organic layer after acid wash

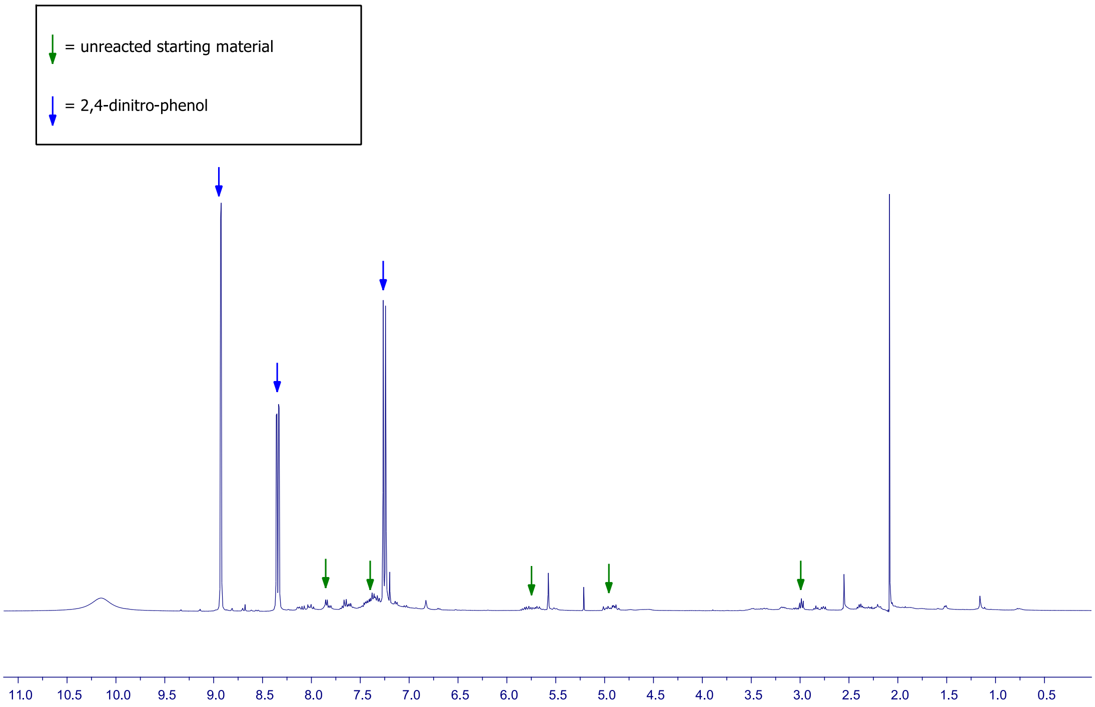

^{1}H NMR of the organic layer after base wash

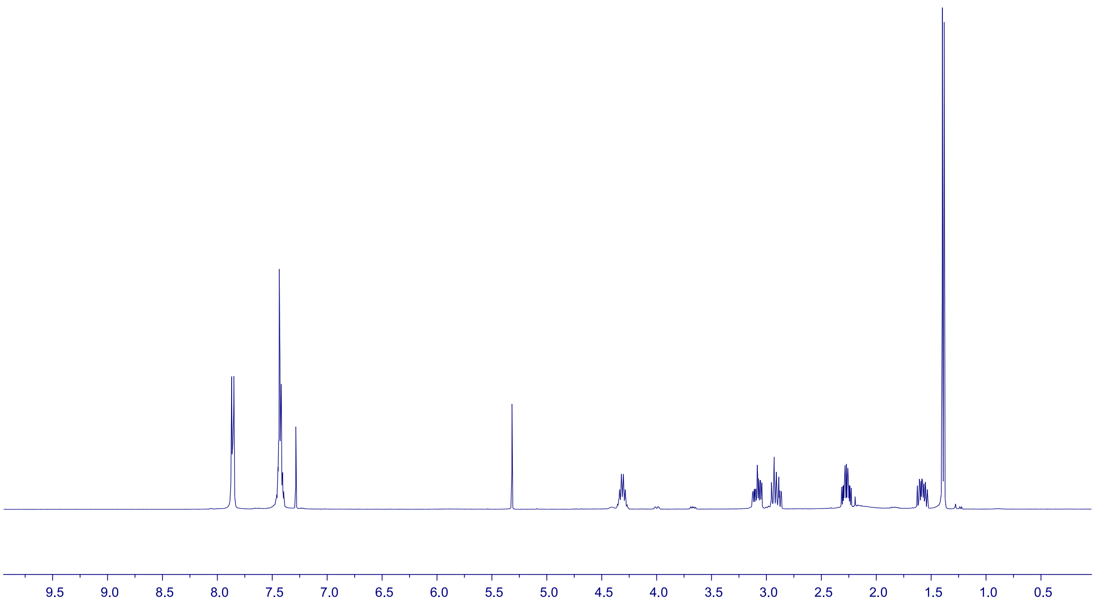

^{13}C NMR of the organic layer after base wash

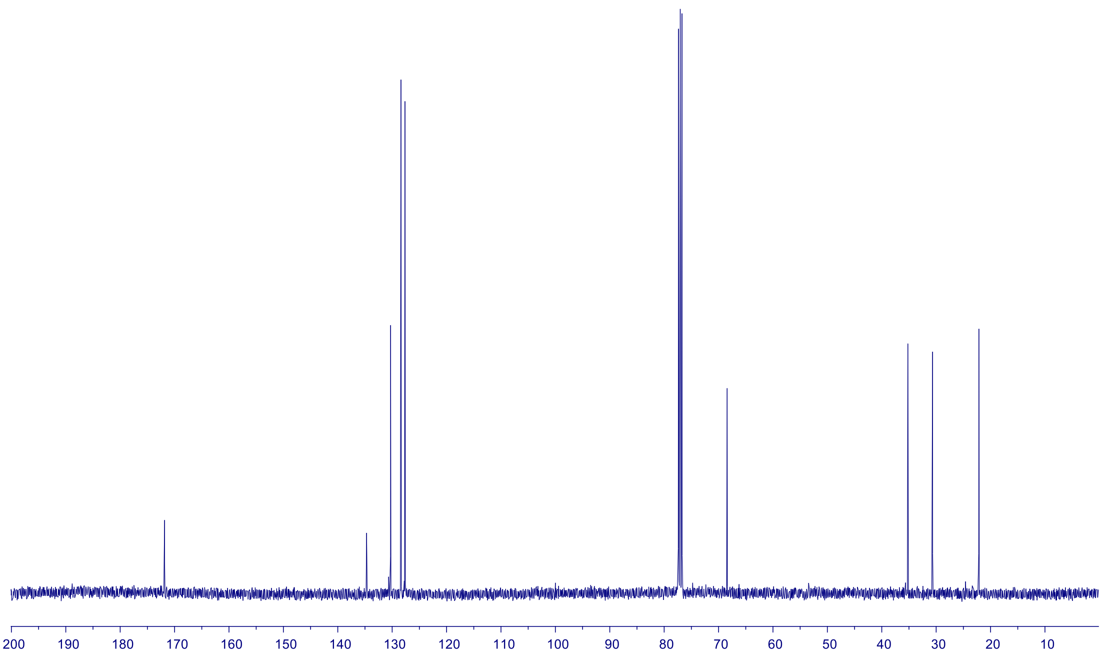

4.2 Mechanistic Studies

4.2.1 Proposed Mechanism

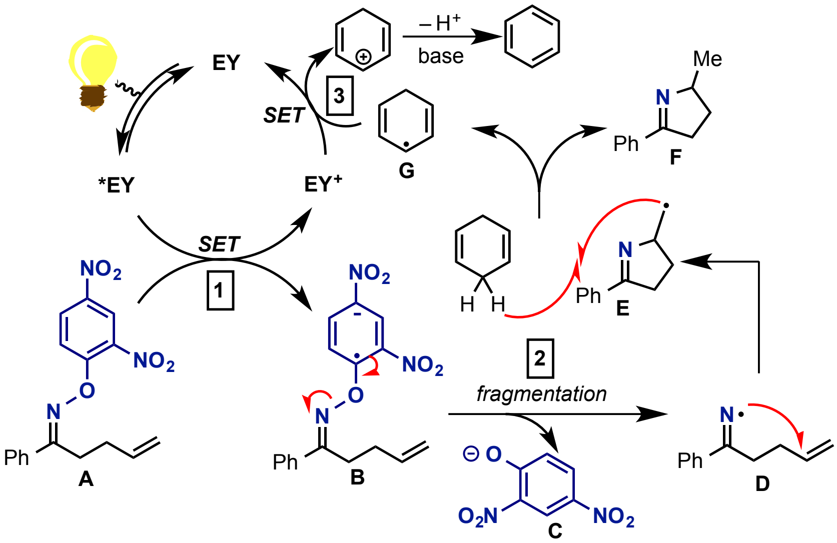

The photoredox cycle is supported by all the control experiments present in Section 4.1: entries 5, 6, 7, 8, 20 and the Light ON/OFF reaction (Section 4.2.3).

Further evidences:

STEP 1. The initial SET step between A and *EY is supported by the emission quenching experiments, see Section 4.2.2, and the results from the reactions with monochromatic LEDs, see Section 4.2.4.

STEP 2. The fragmentation step is supported by the isolation of **C** in quantitative yield.

STEP 3. The final SET step that concludes the catalytic cycle is supported by the identification by GC-MS of antracene when the reaction was run using **HD1** as the H-source. Antracene could not be detected if the reaction was run in the absence of light.

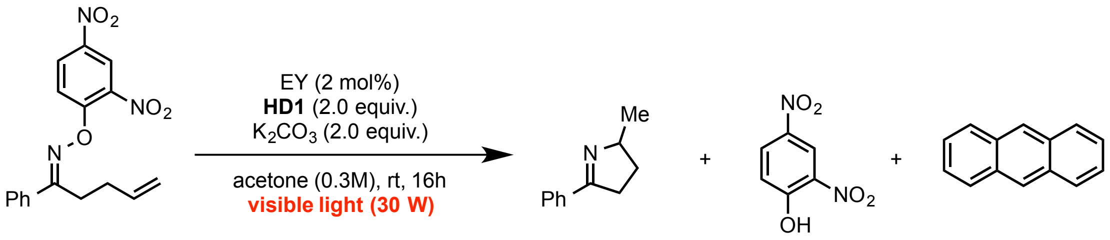

In a recent elegant account Yoon has demonstrated how Light-ON/OFF reactions are sometimes not sufficient to rule out the presence of radical chain processes.[32]

In this particular example the only possible radical chain event would involve the SET from C-radical **E** to **A** to give the radical anion **B** and the carbocation **H**. Even if this might seem plausible, the only way to access product **F** would be by hydride addition onto **H** (or by

hydride opening of the corresponding the aziridium ion). The fact the in the presence of the hydride donor **HD2** no product was obtained (Section 4.1, entry 2), rules out a radical chain process and support the proposed photoredox mechanism.

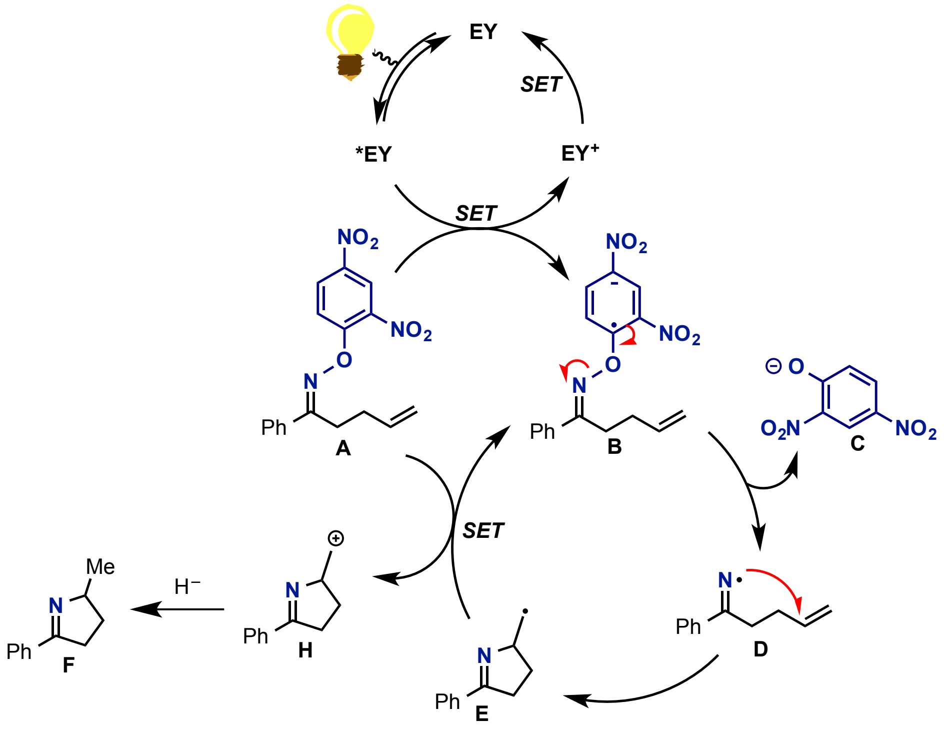

4.2.2 Emission quenching experiments (Stern-Volmer Studies)

Emission intensities were recorded using a Steady State emission spectra were recorded on an Edinburgh Instrument FP920 Phosphorescence Lifetime Spectrometer equipped with a 5 watt microsecond pulsed xenon flash lamp and a 450 watt steady state xenon lamp and a red sensitive photomultiplier in peltier (air cooled) housing, (Hamamatsu R928P). spectrophotometer. All the EY solutions were excited at 400 nm and the emission intensity was collected at 555 nm.[33]

Experimental procedure:

A screw-top quartz cuvette was charged with a 0.1 mM solution of EY in MeOH (2.0 mL) and the initial emission was collected then the appropriate amount of **2a** as a 0.2 M solution in MeOH–CH_2Cl_2 (1:1) was added. The sample was shaken for 1 min and then the emission of the sample was collected.

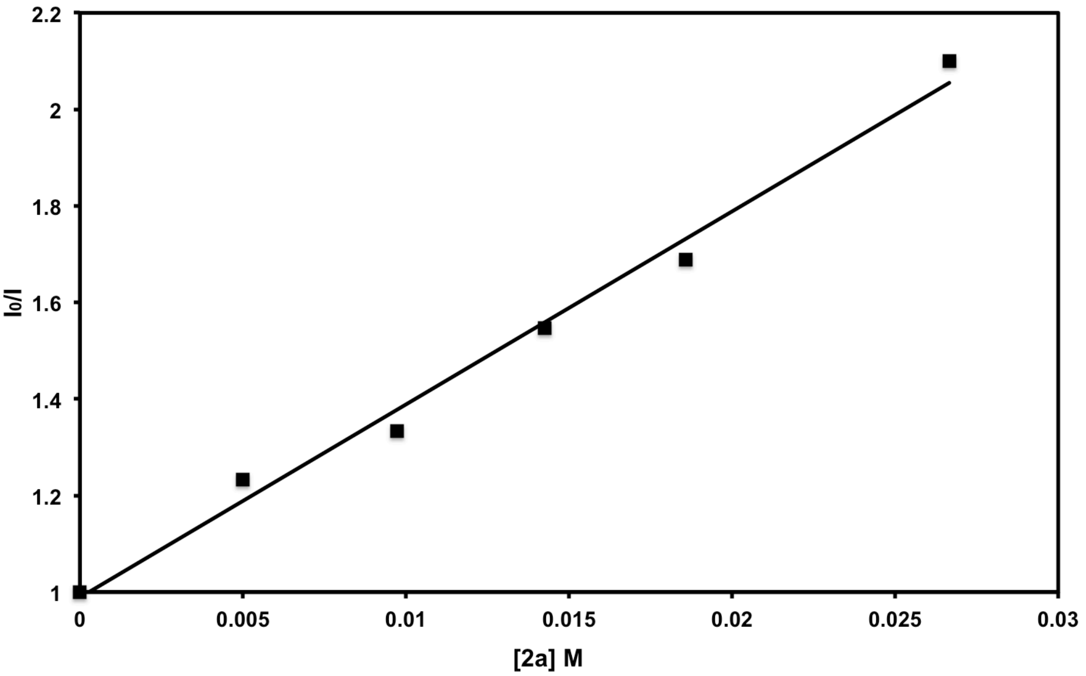

4.2.3 Light ON/OFF reaction

A dry tube equipped with a stirring bar was charged with **2a** (30 mg, 0.088 mmol, 1.0 equiv.), K_2CO_3 (24 mg, 0.176 mmol, 2.0 equiv.), eosin Y (1.2 mg, 1.76 μmol, 2 mol%), acetone-d_6 (0.27 mL) and CHD (34 μL, 0.176 mmol, 4.0 equiv.). The mixture for stirred for 15 min and then the 30 W light bulb was switched on and stirred under visible light irradiation for 6h at which point a reaction aliquot (10 μL) was taken, and diluted with $CDCl_3$ and analysed by ^1H NMR spectroscopy. The light bulb was switched off and the mixture was stirred in the dark for 12h at which point a reaction aliquot (10 μL) was taken, and diluted with $CDCl_3$ and analysed by ^1H NMR spectroscopy. The light bulb was switched on and the mixture stirred under visible light irradiation for 6h at which point a reaction aliquot (10 μL) was taken, and diluted with $CDCl_3$ and analysed by ^1H NMR spectroscopy.

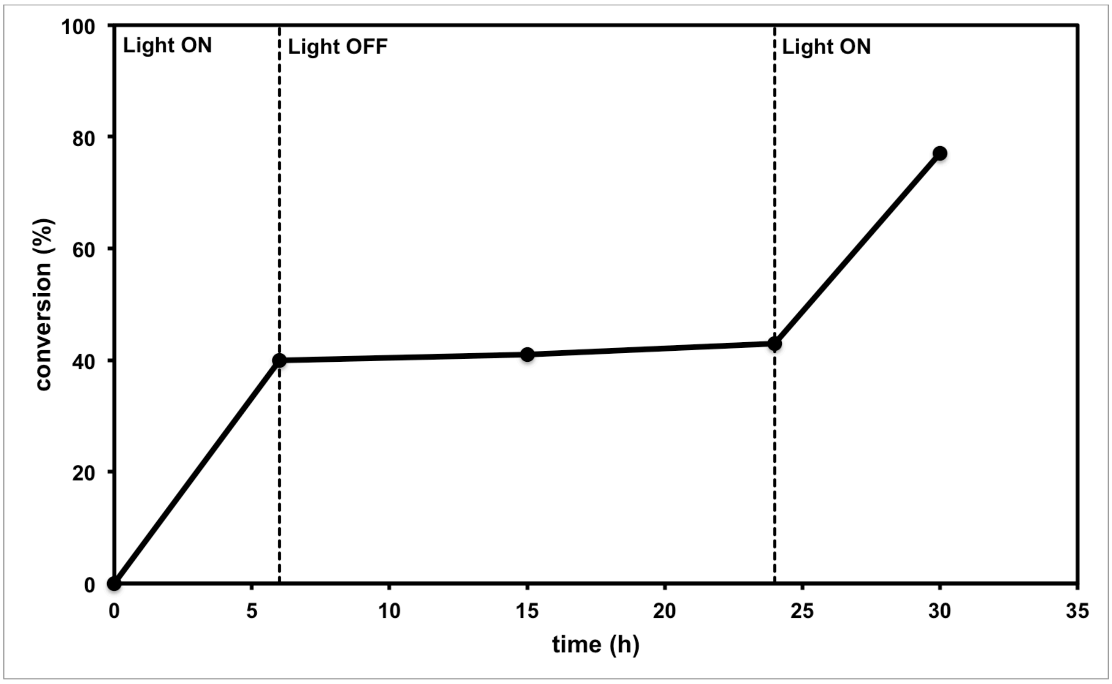

4.2.4 Reaction with monochromatic LEDs

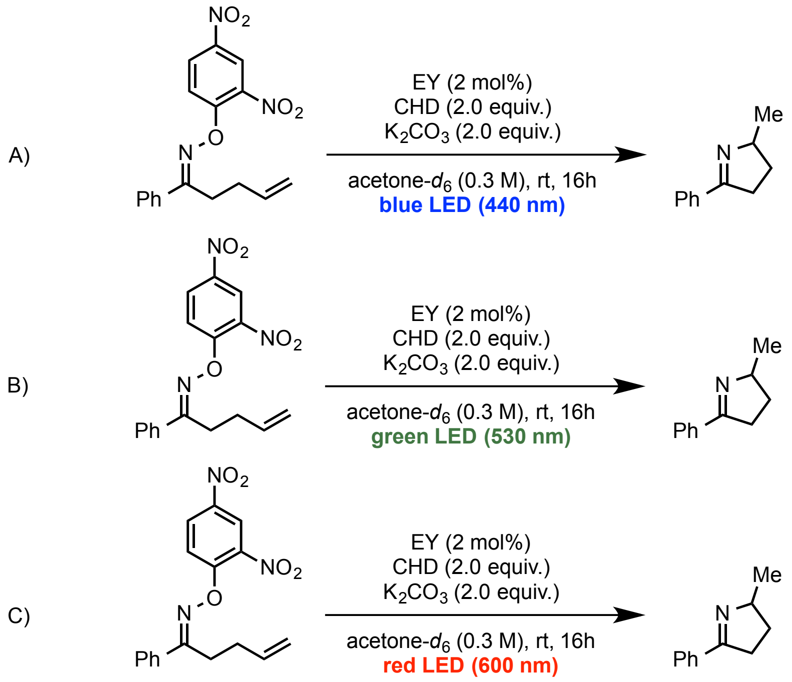

Three dry tubes equipped with a stirring bar were charged with **2a** (20 mg, 0.059 mmol, 1.0 equiv.), EY (0.8 mg, 1.76 µmol, 2 mol%), K_2CO_3 (32 mg, 0.176 mmol, 2.0 equiv.), CHD (22 µL, 0.234 mmol, 4.0 equiv.) and acetone-d_6 (0.2 mL). The mixtures were stirred at room temperature overnight in front of a blue (A), a green (B) and a red LED (C). The mixtures were diluted with $CDCl_3$ (0.5 mL) and analysed by ^{1}H NMR spectroscopy to determine the reaction conversion.

Entry	Reaction	Conversion (%)
1	**A**	60
2	**B**	95
3	**C**	31

These results support the overall mechanism as green light has the optimum wavelength to promote EY to excited state *EY. This is expected to deliver a more efficient catalytic cycle.

4.3 Substrate scope

General Procedure for the hydroimination-cyclization reaction – GP10

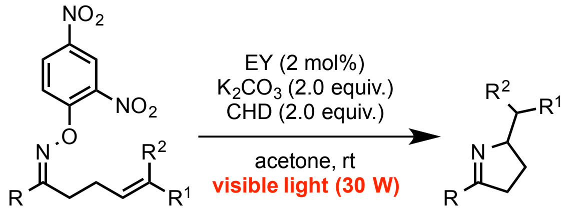

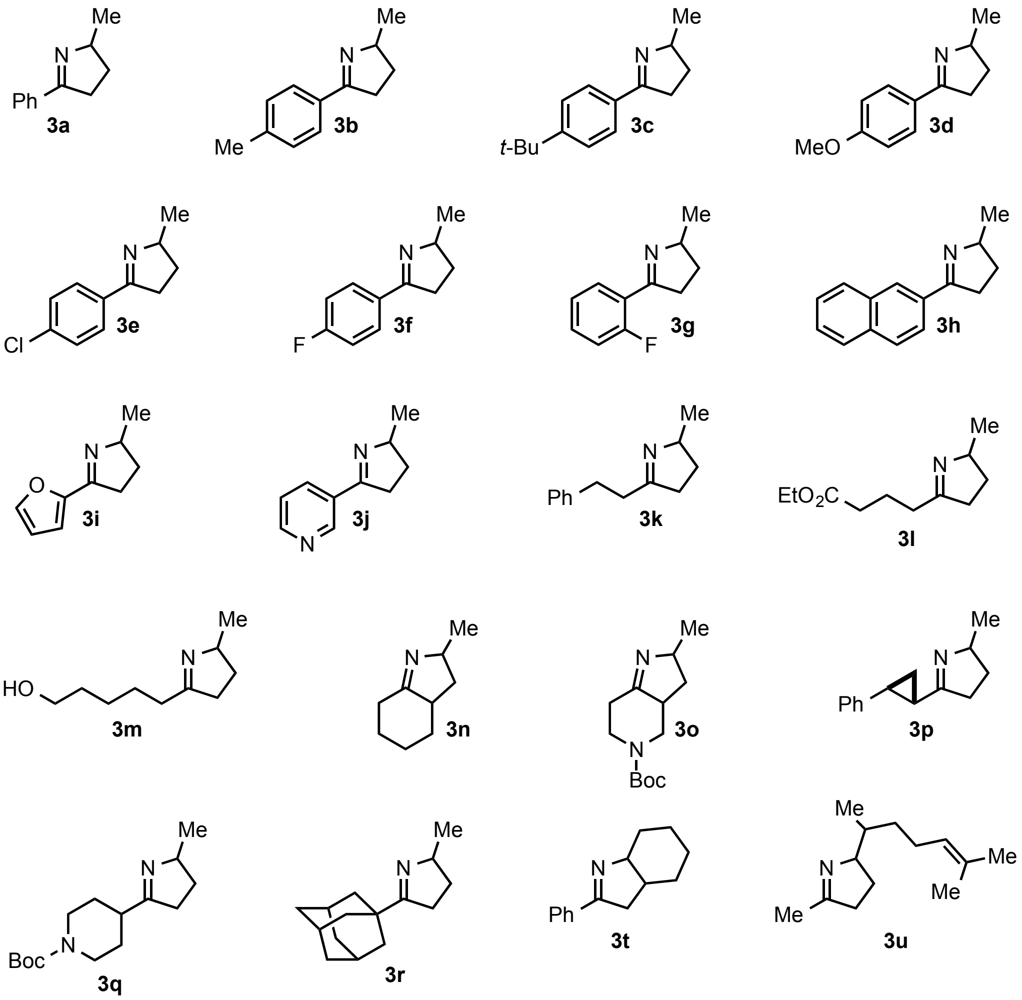

A dry tube equipped with a stirring bar was charged with the oxime (1.0 equiv.), EY (2 mol%), K_2CO_3 (2.0 equiv.), 1,4-cyclohexadiene (2.0 equiv.) and acetone (0.3 M). The mixture was stirred at room temperature overnight in front of a 30 W fluorescent household bulb until judged complete by TLC analysis. The mixture was diluted with HCl (1M) and CH_2Cl_2. The layers were separated and the aqueous layer was basified to pH=14 with NH_4OH. The aqueous layer was extracted with CH_2Cl_2 (x 3) and the combined organic layers were dried ($MgSO_4$), filtered and evaporated to give the desired product as an oil.

2-Methyl-5-phenyl-3,4-dihydro-2H-pyrrole (3a)

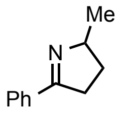

Following **GP10**, **2a** (341 mg, 1.0 mmol) gave **3a** (124 mg, 82%) as an oil. ^{1}H NMR (400 MHz, CDCl$_3$) δ 7.84 (1H, dd, *J* = 7.3, 2.0 Hz), 7.45–7.36 (2H, m), 4.29 (1H, q, *J* = 6.9 Hz), 3.06 (1H, dddd, *J* = 14.5, 9.8, 4.7, 2.1 Hz), 2.95–2.81 (1H, m), 2.30–2.19 (1H, m), 1.60–1.47 (1H, m), 1.36 (1H, d, *J* = 6.8 Hz); ^{13}C NMR (101 MHz, CDCl$_3$) δ 172.1, 134.7, 130.5, 128.5, 127.8, 68.5, 35.3, 30.7, 22.2; HRMS (EI) found M+ 159.1039 $C_{11}H_{13}N$ requires 159.1018. Data in accordance with the literature.[34]

2-Methyl-5-(*p*-tolyl)-3,4-dihydro-2*H*-pyrrole (3b)

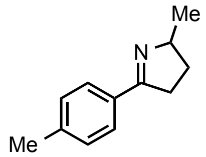

Following **GP10**, **2e** (100 mg, 0.28 mmol) gave **3b** (33 mg, 70 %) as an oil. ^{1}H NMR (400 MHz, CDCl$_3$) δ 7.73 (2H, d, *J* = 8.2 Hz), 7.20 (2H, d, *J* = 7.9 Hz), 4.27 (1H, q, *J* = 6.9 Hz), 3.04 (1H, dddd, *J* = 16.8, 9.9, 4.8, 2.1 Hz), 2.88 (1H, dddd, *J* = 17.0, 9.6, 7.7, 1.7 Hz), 2.38 (3H, s), 2.23 (1H, dddd, *J* = 12.5, 9.8, 7.6, 4.8 Hz), 1.54 (1H, dddd, *J* = 12.7, 9.9, 7.7, 6.7 Hz), 1.36 (3H, d, *J* = 6.8 Hz).; ^{13}C NMR (101 MHz, CDCl$_3$) δ 172.6, 141.4, 131.9, 129.6, 128.3, 68.4, 35.6, 31.0, 22.5, 21.9. Data in accordance with the literature.[34]

5-(4-(*tert*-Butyl)phenyl)-2-methyl-3,4-dihydro-2*H*-pyrrole (3c)

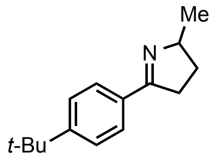

Following **GP10**, **2f** (56 mg, 0.14 mmol) gave **3c** (16.5 mg, 55%) as an oil; R$_f$ 0.76 [petrol–EtOAc (90:10)]; FT-IR ν_{max} (film)/cm^{-1} 2960, 2866, 1613, 1460, 1405, 1363, 1336, 1268, 1110, 1012; ^{1}H NMR (400 MHz, CDCl$_3$) δ 7.80 (2H, d, *J* = 8.5 Hz), 7.44 (2H, d, *J* = 8.6 Hz), 4.30 (1H, q, *J* = 6.8 Hz), 3.08 (1H, dddd, *J* = 16.8, 9.8, 4.9, 2.1 Hz), 2.89 (1H, dddd, *J* = 17.0, 9.5, 7.6, 1.7 Hz), 2.25 (1H, dddd, *J* = 12.6, 9.8, 7.6, 4.9 Hz), 1.57 (1H, dddd, *J* = 12.7, 9.9, 7.6, 6.6 Hz), 1.37 3H, d, *J* = 6.8 Hz), 1.35 (9H, s); ^{13}C NMR (101 MHz, CDCl$_3$) δ 172.0, 154.0, 131.7, 127.7, 125.5, 68.2, 35.2, 35.0, 31.2, 30.6, 22.2; HRMS (EI) found M+ 215.1662 $C_{15}H_{21}N$ requires 215.1669.

5-(4-Methoxyphenyl)-2-methyl-3,4-dihydro-2*H*-pyrrole (3d)

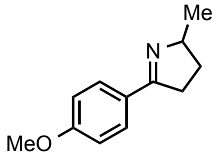

Following **GP10**, **2g** (52 mg, 0.14 mmol) gave **3d** (23 mg, 87%) as an oil; ^{1}H NMR (400 MHz, $CDCl_3$) δ 7.82 (2H, d, *J* = 8.8 Hz), 6.91 (2H, d, *J* = 8.9 Hz), 4.27 (1H, q, *J* = 6.8 Hz), 3.84 (3H, s), 3.05 (1H, dddd, *J* = 16.7, 9.8, 4.7, 2.0 Hz), 2.88 (1H, dddd, *J* = 17.2, 9.6, 7.8, 1.6 Hz), 2.24 (1H, dddd, *J* = 12.5, 9.8, 7.6, 4.8 Hz), 1.55 (1H, dddd, *J* = 12.7, 9.8, 7.7, 6.8 Hz), 1.36 (3H, d, *J* = 6.7 Hz); ^{13}C NMR (101 MHz, $CDCl_3$) δ 171.8, 161.7, 129.7, 127.0, 113.9, 108.8, 67.9, 55.5, 35.2, 30.7; Data in accordance with the literature.[35]

5-(4-Chlorophenyl)-2-methyl-3,4-dihydro-2*H*-pyrrole (3e)

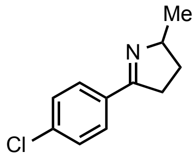

Following **GP10**, **2h** (53 mg, 0.14 mmol) gave **3e** (18 mg, 81%) as an oil; ^{1}H NMR (400 MHz, $CDCl_3$) δ 7.76 (2H, d, *J* = 8.5 Hz), 7.36 (2H, d, *J* = 8.6 Hz), 4.28 (1H, q, *J* = 6.9 Hz), 3.02 (1H, dddd, *J* = 16.7, 9.9, 4.7, 2.1 Hz), 2.85 (1H, dddd, *J* = 16.8, 9.3, 7.7, 1.6 Hz), 2.25 (1H, *J* = 12.5, 9.8, 7.7, 4.7 Hz), 1.61 – 1.49 (1H, m), 1.35 (3H, d, *J* = 6.8 Hz).; ^{13}C NMR (101 MHz, $CDCl_3$) δ 171.0, 136.5, 133.2, 129.1, 128.8, 68.6, 35.3, 30.8, 22.2; Data in accordance with the literature.[35]

5-(4-Fluorophenyl)-2-methyl-3,4-dihydro-2*H*-pyrrole (3f)

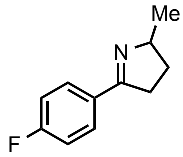

Following **GP10**, **2i** (102 mg, 0.28 mmol) gave **3f** (38.5 mg, 84%) as an oil; ^{1}H NMR (400 MHz, $CDCl_3$) δ 7.83 (1H, dd, *J* = 8.8, 5.5 Hz), 7.08 (1H, dd, *J* = 12.0, 5.4 Hz), 4.27 (1H, q, *J* = 6.9 Hz), 3.03 (1H, dddd, *J* = 16.8, 9.9, 4.7, 2.1 Hz), 2.86 (1H, dddd, *J* = 17.1, 9.7, 7.8, 1.7 Hz), 2.25 (1H, dddd, *J* = 12.5, 9.7, 7.6, 4.7 Hz), 1.55 (1H, dddd, *J* = 14.5, 9.8, 7.7, 5.1 Hz), 1.35 (1H, d, *J* = 6.8 Hz); ^{13}C NMR (101 MHz, $CDCl_3$) δ 170.9, 164.2 (d, *J* = 250.3 Hz), 131.0 (d, *J* = 3.1 Hz), 129.8 (d, *J* = 8.6 Hz), 115.5 (d, *J* = 21.6 Hz), 68.5, 35.4, 30.8, 22.2; ^{19}F NMR (376 MHz, $CDCl_3$, decoupled) δ –110.3; HRMS (EI) found MH^+ 178.1039 $C_{11}H_{13}NF$ requires 178.1032. Data in accordance with the literature.[34]

5-(2-Fluorophenyl)-2-methyl-3,4-dihydro-2*H*-pyrrole (3g)

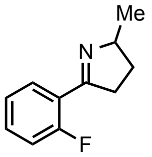

Following **GP10**, **2j** (51 mg, 0.14 mmol) gave **3g** (15 mg, 66%) as an oil; R_f 0.74 [CH_2Cl_2–MeOH (90:10)]; FT-IR ν_{max} (film)/cm^{-1} 2963, 2926, 1611, 1486, 1452, 1274, 1215, 1104; ^{1}H NMR (400 MHz, $CDCl_3$) δ 7.93 (1H, td, *J* = 7.7, 1.5 Hz), 7.38 (1H, ddd, *J* = 13.5, 7.5, 1.6 Hz), 7.16 (1H, t, *J* = 7.6 Hz), 7.08 (1H, dd, *J* = 11.3, 8.3 Hz), 4.23 (1H, q, *J* = 7.0 Hz), 3.11 (1H, dddd, *J* = 14.6, 9.7, 4.8, 2.2 Hz), 3.02–2.90 (1H, m), 2.30–2.18 (1H, m,), 1.60–1.50 (1H, m), 1.36 (3H, d, *J* = 6.8 Hz); ^{13}C NMR (101 MHz, $CDCl_3$) δ 169.5 (s), 161.6 (d, *J* = 252.3 Hz), 131.9 (d, *J* = 8.6 Hz), 130.3 (d, *J* = 3.6 Hz), 124.3 (d, *J* = 3.3 Hz), 123.0 (d, *J* = 11.9 Hz), 67.5, 38.2, 31.0, 22.1; HRMS (EI): Found M^+ 177.0945 $C_{11}H_{12}NF$ requires 177.0948.

2-Methyl-5-(naphthalen-2-yl)-3,4-dihydro-2*H*-pyrrole (3h)

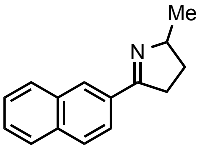

Following **GP10** but purifying the product by column chromatography, **2k** (38 mg, 0.1 mmol) gave **3h** (12 mg, 62%) as an oil; R_f 0.74 [CH_2Cl_2–MeOH (90:10)]; FT-IR ν_{max} (film)/cm^{-1} 3057, 2960, 2925, 2863, 1607, 1573, 1450, 1350, 1274, 1194, 1126, 1053;; ^{1}H NMR (400 MHz, $CDCl_3$) δ 8.10 (1H, br s), 8.01 (1H, dd, *J* = 8.6, 1.7 Hz), 7.82 (1H, dd, *J* = 6.2, 2.9 Hz), 7.79–7.77 (2H, m), 7.46–7.41 (2H, m), 4.27 (1H, q, *J* = 7.0 Hz), 3.12 (1H, dddd, *J* = 16.7, 9.9, 4.7, 2.1 Hz), 2.98–2.90 (1H, m), 2.23 (1H, dddd, *J* = 12.5, 9.8, 7.7, 4.8 Hz), 1.54 (1H, dddd, *J* = 12.6, 9.9, 7.7, 6.8 Hz), 1.34 (3H, d, *J* = 6.8 Hz); ^{13}C NMR (101 MHz, $CDCl_3$) δ 171.9, 134.3, 133.0, 132.2, 128.7, 128.13, 128.10, 127.8, 127.0, 126.4, 124.6, 68.6, 35.3, 30.8, 22.2; HRMS (EI): Found MH^+ 209.1196 $C_{15}H_{16}N$ requires 209.1199.

5-(Furan-2-yl)-2-methyl-3,4-dihydro-2*H*-pyrrole (3i)

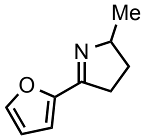

Following **GP10**, **2l** (94 mg, 0.28 mmol) gave **3i** (23 mg, 55%) as an oil; R_f 0.65 [CH_2Cl_2–MeOH (90:10)]; FT-IR ν_{max} (film)/cm^{-1} 2925, 1265, 1162, 1079, 1012; ^{1}H NMR (400 MHz, $CDCl_3$) δ 8.96 (1H, d, *J* = 1.6 Hz), 8.64 (1H, dd, *J* = 4.8, 1.6 Hz), 8.18 (1H, dt, *J* = 8.0, 2.0 Hz), 7.33 (1H, ddd, *J* = 8.0, 4.8, 0.8 Hz), 4.29 (1H, ddt, *J* = 14.4, 6.9, 2.0 Hz), 3.06 (1H, dddd,

J = 16.9, 9.9, 4.8, 2.2 Hz), 2.92 (1H, ddd, J = 17.5, 9.7, 1.9 Hz), 2.27 (1H, dddd, J = 12.6, 9.8, 7.7, 4.8 Hz), 1.58 (1H, dddd, J = 12.8, 9.9, 7.7, 6.7 Hz), 1.36 (3H, d, J = 6.8 Hz); ^{13}C NMR (101 MHz, $CDCl_3$) δ 169.7, 151.3, 149.2, 134.9, 130.4, 123.6, 68.8, 35.2, 30.6, 22.1; LRMS *m/z* (ESI): 150 (MH^+), 134, 120.

5-(Furan-2-yl)-2-methyl-3,4-dihydro-2*H*-pyrrole (3j)

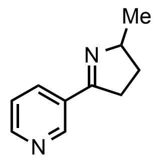

Following **GP10**, **2m** (48 mg, 0.14 mmol) gave **3j** (15 mg, 66%) as an oil. R_f 0.76 [CH_2Cl_2–MeOH (90:10)]; FT-IR ν_{max} (film)/cm^{-1} 2962, 2866, 1616, 1589, 1453, 1412, 1340, 1290, 1190, 1089, 1011; ^{1}H NMR (400 MHz, $CDCl_3$) δ 8.97 (1H, s, br), 8.64 (1H, dd, J = 4.8, 1.5 Hz), 8.18 (1H, dt, J = 8.0, 1.9, 1.9 Hz), 7.33 (1H, ddd, J = 7.9, 4.8, 0.7 Hz), 4.30 (1H, q, J = 6.9 Hz), 3.14 – 2.81 (1H, m), 2.27 (2H?, ddd, J = 12.4, 7.9, 1.6 Hz), 1.57 (1H, dd, J = 12.6, 7.1 Hz), 1.36 (3H, d, J = 6.8 Hz). ^{13}C NMR (101 MHz, $CDCl_3$) δ 169.7, 151.3, 149.2, 134.9, 130.4, 123.6, 68.8, 35.2, 30.6, 22.1. LRMS *m/z* (ESI): 160 (M^+), 145, 132.

2-Methyl-5-phenethyl-3,4-dihydro-2*H*-pyrrole (3k)

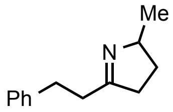

Following **GP10**, **2n** (104 mg, 0.28 mmol) gave **3k** (37 mg, 72%) as an oil; ^{1}H NMR (400 MHz, $CDCl_3$) δ 7.21 (1H, d, J = 7.3 Hz), 7.17–7.09 (1H, m), 3.97 (1H, q, J = 6.8 Hz, 1H), 2.89 – 2.80 (2H, m), 2.56 (1H, dd, J = 12.4, 4.5 Hz), 2.46–2.27 (1H, m), 2.04–1.92 (1H, m), 1.35–1.21 (1H, m), 1.18 (1H, d, J = 6.7 Hz); ^{13}C NMR (101 MHz, $CDCl_3$) δ 176.5, 141.6, 128.5, 128.4, 126.1, 67.8, 37.9, 35.6, 32.8, 30.7. Data in accordance with the literature.[36]

Ethyl 4-(2-Methyl-3,4-dihydro-2*H*-pyrrol-5-yl)butanoate (3l)

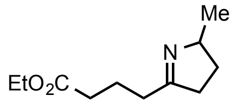

Following **GP10**, **2o** (108 mg, 0.28 mmol) gave **3l** (38 mg, 68%) as an oil; ^{1}H NMR (400 MHz, $CDCl_3$) δ 4.06 (2H, q, J = 7.1 Hz), 4.01–3.92 (1H, m), 2.48 (1H, dddd, J = 17.1, 9.9, 4.9, 2.0 Hz), 2.41–2.32 (1H, m), 2.28 (4H, app t, J = 7.4 Hz), 2.01 (1H, dddd, J = 12.6, 9.7, 7.7, 4.9 Hz), 1.90-1.82 (2H, m), 1.31 (1H, dddd, J = 12.7, 9.8, 7.9, 6.7 Hz), 1.18 (3H, t, J = 7.1 Hz), 1.17 (3H, d, J = 6.8 Hz); ^{13}C NMR (101 MHz, $CDCl_3$) δ 176.0, 173.3, 67.7, 60.3,

37.5, 33.8, 33.0, 30.6, 22.1, 21.7, 14.2; HRMS (EI): Found MH^+ 198.1497 $C_{11}H_{21}NO_2$ requires 198.1494.

5-(2-Methyl-3,4-dihydro-2*H*-pyrrol-5-yl)pentan-1-ol (3m)

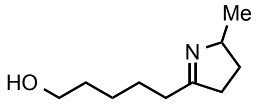

Following **GP10**, **2p** (100 mg, 0.21 mmol) gave **3m** (10 mg, 28%) as an oil; ^{1}H NMR (400 MHz, $CDCl_3$) δ 4.06–4.01 (1H, m), 3.66 (2H, t, *J* = 6.4 Hz), 2.68 (1H, br s, OH), 2.55 (1H, dddd, *J* = 17.2, 9.9, 4.9, 2.0 Hz), 2.48–2.39 (1H, m), 2.33 (2H, td, *J* = 7.4, 1.6 Hz), 2.08 (1H, dddd, *J* = 12.6, 9.7, 7.7, 4.9 Hz), 1.68–1.56 (4H, m), 1.47–1.33 (3H, m), 1.25 (3H, d, *J* = 6.8 Hz); ^{13}C NMR (101 MHz, $CDCl_3$) δ 177.3, 67.4, 62.1, 37.6, 33.5, 32.1, 30.5, 25.36, 25.30, 22.0; HRMS (EI): Found MH^+ 170.1544 $C_{10}H_{20}NO$ requires 170.1545.

2-Methyl-3,3a,4,5,6,7-hexahydro-2*H*-indole (3n)

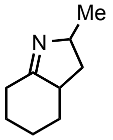

Following **GP10**, **2q** (45 mg, 0.14 mmol) gave **3m** (17 mg, 88%) as an oil; dr 3:2. ^{1}H NMR (400 MHz, $CDCl_3$) δ 4.16–4.05 (1H, m), 3.89–3.81 (1H, m), 2.68–2.59 (1H, m), 2.57–2.43 (1H, m), 2.31 (1H, dd, *J* = 12.8, 7.1 Hz), 2.29 (1H, dd, *J* = 12.7, 8.6 Hz), 2.16–2.06 (4H, m), 2.03–1.91 (3H, m), 1.83–1.75 (3H, m), 1.73–1.59 (3H, m), 1.47–1.38 (4H, m), 1.31 (3H, d, *J* = 6.8 Hz), 1.28 (1H, dd, *J* = 4.1, 2.7 Hz), 1.13 (3H, d, *J* = 6.8 Hz); ^{13}C NMR (101 MHz, $CDCl_3$, diastereomers) δ 178.2 & 177.6, 84.0 & 83.7, 64.4 & 64.1, 46.9 & 45.7, 40.5 &, 40.0, 28.7 & 28.5, 27.6 & 26.6, 23.1 &, 22.5, 21.5 & 21.4. Data in accordance with the literature.[37]

***tert*-Butyl 2-Methyl-2,3,3a,4,6,7-hexahydro-5*H*-pyrrolo[3,2-*c*]pyridine-5-carboxylate (3o)**

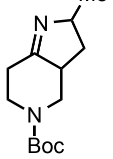

Following **GP10**, **2r** (100 mg, 0.24 mmol) gave **3o** (40 mg, 70%) as an oil; dr 1.5:1. ^{1}H NMR (400 MHz, $CDCl_3$, diastereomers and rotamers) δ 4.63–4.29 (2H, m), 4.28–4.18 (0.65H, m), 4.0–3.91 (0.45H, m), 3.76–3.69 (1H, m), 2.59–2.54 (1H, m), 2.46–2.38 (2H, m), 1.49 & 1.48 (9H, s), 1.35 (1.65H, d, *J* = 6.8 Hz), 1.15 (1.35H, d, *J* = 6.9 Hz); ^{13}C NMR (101 MHz, $CDCl_3$, diastereomers and rotamers) δ 175.0 & 174.7, 154.5, 80.5 & 80.1, 66.7 & 66.64, 50.5 (br),

48.1, 44.0 (br), 35.1, 33.2, 28.4, 22.6 & 22.0; HRMS (EI): Found MH^+ 239.1765 $C_{13}H_{23}N2O_2$ requires 239.1760.

2-Methyl-5-(2-phenylcyclopropyl)-3,4-dihydro-2*H*-pyrrole (3p)

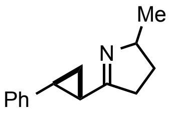

Following **GP10**, **2s** (54 mg, 0.14 mmol) gave **3p** (21 mg, 75%) as an oil; dr 1:1. R_f 0.71 [CH_2Cl_2–MeOH (90:10)]; FT-IR ν_{max} (film)/cm^{-1} 2960, 2925, 1631, 1604; ^{1}H NMR (400 MHz, $CDCl_3$, diastereomers) δ 7.31–7.27 (2H, m), 7.22–7.18 (1H, m), 7.14–7.12 (2H, m), 4.06 (1H, br s), 2.59–2.43 (2H, m), 2.38 (1H, td, *J* = 9.6, 5.2 Hz), 2.17–2.09 (1H, m), 2.08–2.00 (1H, m), 1.55 (1H, ddd, *J* = 8.9, 5.6, 4.7 Hz), 1.47–1.38 (1H, m), 1.37-1.29 (m, 1H), 1.27 (3H, d, *J* = 6.7 Hz); ^{13}C NMR (101 MHz, $CDCl_3$, diastereomers) δ 176.65 & 176.6, 141.55 & 141.5, 128.4, 126.0, 125.95 & 125.90, 67.5, 36.4 & 36.1, 30.6 & 30.55, 25.85 & 25.8, 25.6 & 25.55, 22.2 & 22.15, 17.4 & 17.3; HRMS (EI): Found M+ 199.1352 $C_{14}H_{17}N$ requires 199.1356.

***tert*-Butyl 4-(2-Methyl-3,4-dihydro-2*H*-pyrrol-5-yl)piperidine-1-carboxylate (3q)**

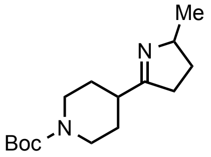

Following **GP10**, **2t** (100 mg, 0.21 mmol) gave **3m** (10 mg, 28%) as an oil; ^{1}H NMR (400 MHz, $CDCl_3$, diastereomers) δ 4.28–4.09 (2H, m), 4.05 (1H, br q, *J* = 7.3 Hz), 2.78 (2H, br t, *J* = 11.1 Hz), 2.63–2.53 (1H, m), 2.51–2.41 (2H, m), 2.10 (1H, dddd, *J* = 12.6, 9.7, 7.7, 4.9 Hz), 1.88-1.76 (2H, m), 1.61–1.58 (2H, m), 1.47 (9H, s), 1.44–1.33 (1H, m), 1.25 (3H, d, *J* = 6.7 Hz); ^{13}C NMR (101 MHz, $CDCl_3$, diastereomers) δ 179.5 179.0, 154.8, 79.7^m & 79.5^M, 67.4, 43.3 (br), 40.5^M & 39.7^m, 35.1, 30.3^M, 29.45^M, 28.5^M, 27.6, 27.5^m, 22.0^m; HRMS (EI): Found MH^+ 267.2070 $C_{15}H_{27}N_2O_2$ requires 267.2073.

5-((3*r*,5*r*,7*r*)-Adamantan-1-yl)-2-methyl-3,4-dihydro-2*H*-pyrrole (3r)

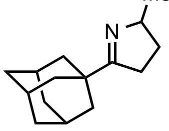

Following **GP10**, **2v** (56 mg, 0.14 mmol) gave **3r** (21 mg, 69%) as an oil. R_f 0.63 [CH_2Cl_2–MeOH (90:10)]; FT-IR ν_{max} (film)/cm^{-1} 2902, 2848, 1662, 1625, 1451, 1371, 1344, 1232, 1102; ^{1}H NMR (400 MHz, $CDCl_3$) δ 4.03 (1H, q, *J* = 7.0 Hz), 2.59 (1H, dddd, *J* = 16.4, 9.8,

5.1, 1.5 Hz), 2.51 – 2.38 (1H, m), 2.12 – 1.66 (16H, m), 1.38 – 1.27 (1H, m), 1.24 (3H, d, *J* = 6.7 Hz); ^{13}C NMR (126 MHz, $CDCl_3$) δ 180.7, 67.1, 40.2, 39.3, 37.7, 36.8, 36.5, 32.5, 30.3, 28.2, 28.1, 22.1. HRMS (EI): Found M+ 217.1821 $C_{15}H_{23}N$ requires 217.1825.

2-Phenyl-3a,4,5,6,7,7a-hexahydro-3*H*-indole (3s)

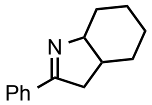

Following **GP10**, **2y** (75 mg, 0.19 mmol) gave **3s** (26 mg, 70%) as an oil. R_f 0.74 [CH_2Cl_2–MeOH (90:10)]; dr 3:1. FT-IR ν_{max} (film)/cm^{-1} 2926, 2852, 1602, 1573, 1447, 1052, 1017; ^{1}H NMR (400 MHz, $CDCl_3$) δ 7.89 – 7.80 (2H, m), 7.47 – 7.34 (3H, m), δ 4.11 (0.29H, s, br), 3.99 (0.71H, q, *J* = 5.6 Hz), 2.97 (0.29H, ddd, *J* = 16.0, 6.4, 2.8 Hz), 2.90 (0.71, ddd, *J* = 16.1, 7.2, 1.9 Hz), 2.82 (0.29H, d, *J* = 16.1 Hz), 2.72 (0.71H, ddd, *J* = 16.1, 4.5, 0.9 Hz), 2.47 (0.29H, td, *J* = 12.0, 6.4 Hz), 2.38 (0.71H, td, *J* = 13.1, 6.6 Hz), 1.90 (2H, dd, *J* = 11.9, 5.8 Hz), 1.65 – 1.55 (2H, m), 1.50 – 1.41 (2H, m), 1.35 – 1.20 (2H, m); ^{13}C NMR (101 MHz, $CDCl_3$) Major isomer: δ 174.5^m & 173.7^M, 135.3^M & 134.8^m 130.6^m & 130.5^M, 128.6^m & 128.5^M, 127.7^m & 127.6^M, 74.6^m, 70.0^M, 43.6^m, 41.6^M, 37.6^m, 36.9^M, 31.3^m, 29.3^M, 27.5^m, 27.5^M, 23.1^M, 23.1^m, 22.2^M, 21.6^m. HRMS (EI): Found M+ 199.1357 $C_{14}H_{17}N$ requires 199.1357.

5-Methyl-2-(6-methylhept-5-en-2-yl)-3,4-dihydro-2*H*-pyrrole (3t)

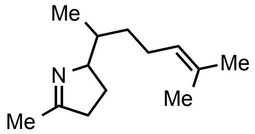

Following **GP10**, **2z** (100 mg, 0.26 mmol) gave **3t** (19 mg, 37%) as an oil, dr 1:1. ^{1}H NMR (400 MHz, $CDCl_3$) δ 5.10–5.00 (1H, m), 3.88–3.82 (0.5H, m), 3.81–3.77 (0.5H, m), 2.45–2.31 (2H, m), 2.01–1.97 (2H, m), 1.99 (1.5H, s), 1.96 (1.5H, s), 1.91–1.74 (2H, m), 1.61 (1.5H, s), 1.60 (1.5H, s), 1.57–1.55 (1H, m), 1.53 (3H, s), 1.39–1.331 (1H, m), 1.15–1.00 (1H, m), 0.90 (1.5H, d, J = 6.7 Hz), 0.71 (1.5H, d, J = 6.7 Hz); ^{13}C NMR (101 MHz, $CDCl_3$) δ 174.0, 131.4, 124.9 & 124.8, 77.9 & 77.7, 39.2 & 39.1, 37.8 & 37.1, 34.3 & 32.9, 26.2 & 25.8, 25.7, 24.5, 19.7, 17.7, 16.3 & 14.6; LRMS 194 (MH^+). Data in accordance with the literature.[38]

5 Iminohydroxilation-cyclization

5.1 Electron-Donor-Acceptor Complex (EDAC) Formation

5.1.1 UV/Vis studies

5.1.1.1 EDAC formation using Et_3N – Oximes evaluations

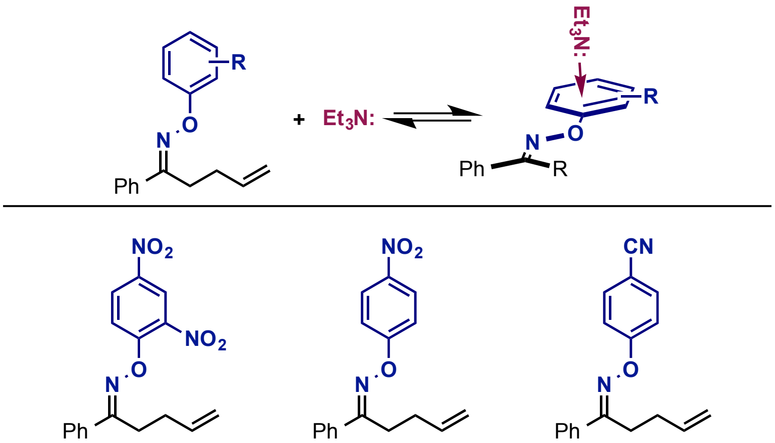

2a **2b** **2e**

A 0.3M solution of the oxime (0.06 mmol, 1.0 equiv.) in CH_3CN (0.2 mL) was treated with Et_3N (16 μL, 0.12 mmol, 2.0 equiv.), stirred at room temperature for 15 min and then analysed by UV-vis spectroscopy using a 1 mm quartz cuvette.

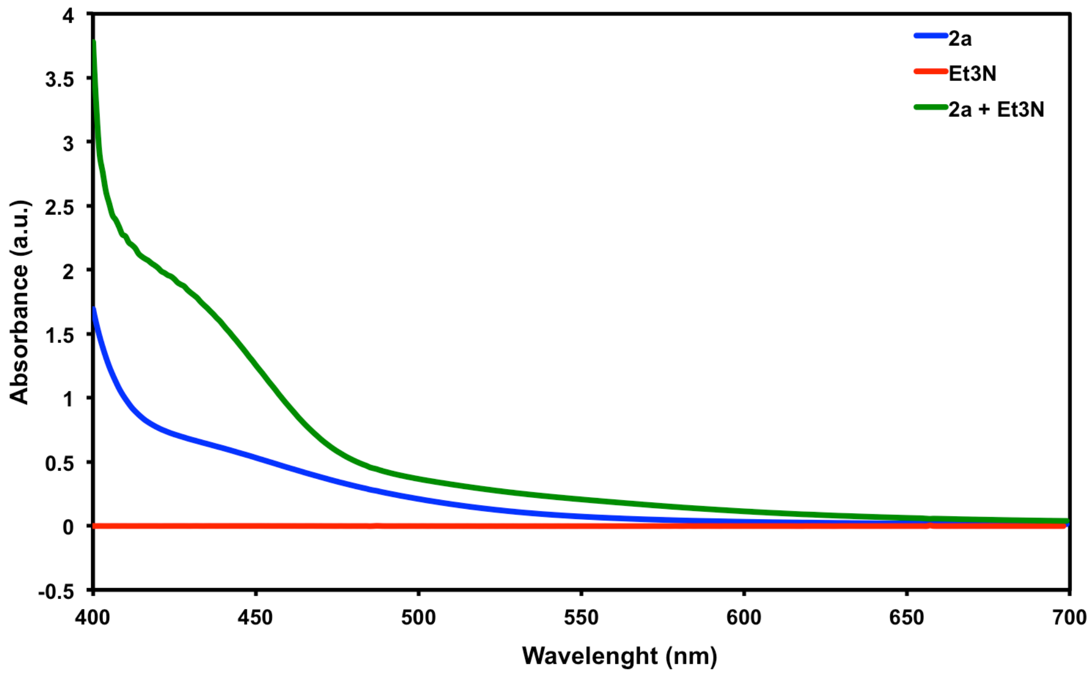

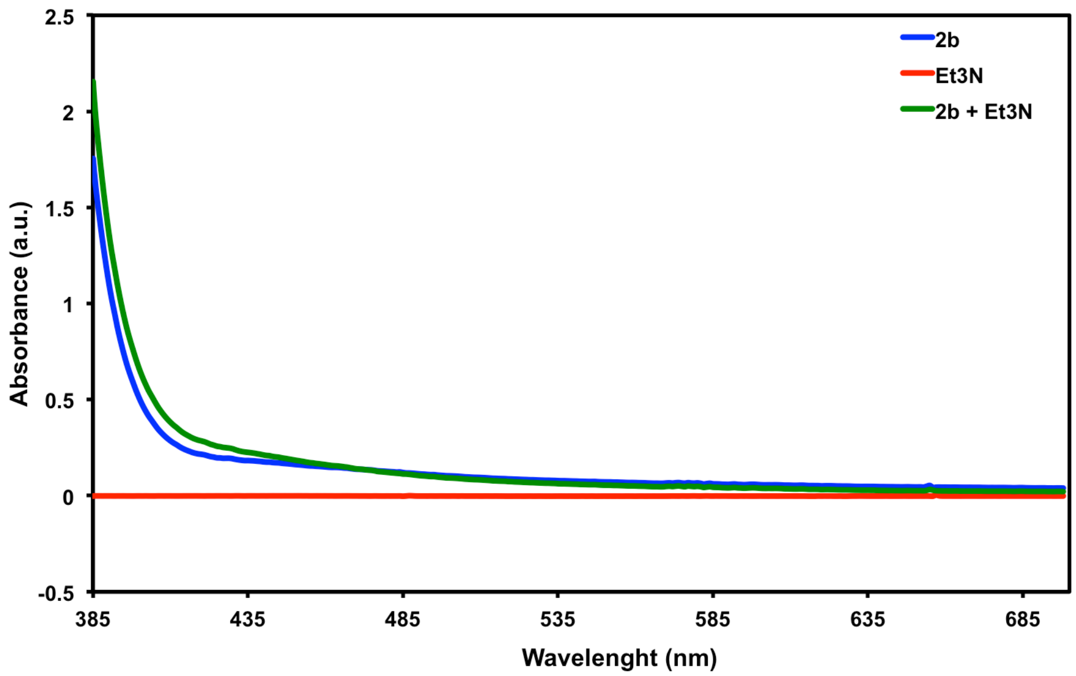

2.5
2
1.5
1
0.5
0
-0.5
Absorbance (a.u.)
385
435
485
535
585
635
685
Wavelenght (nm)
2b
Et3N
2b + Et3N

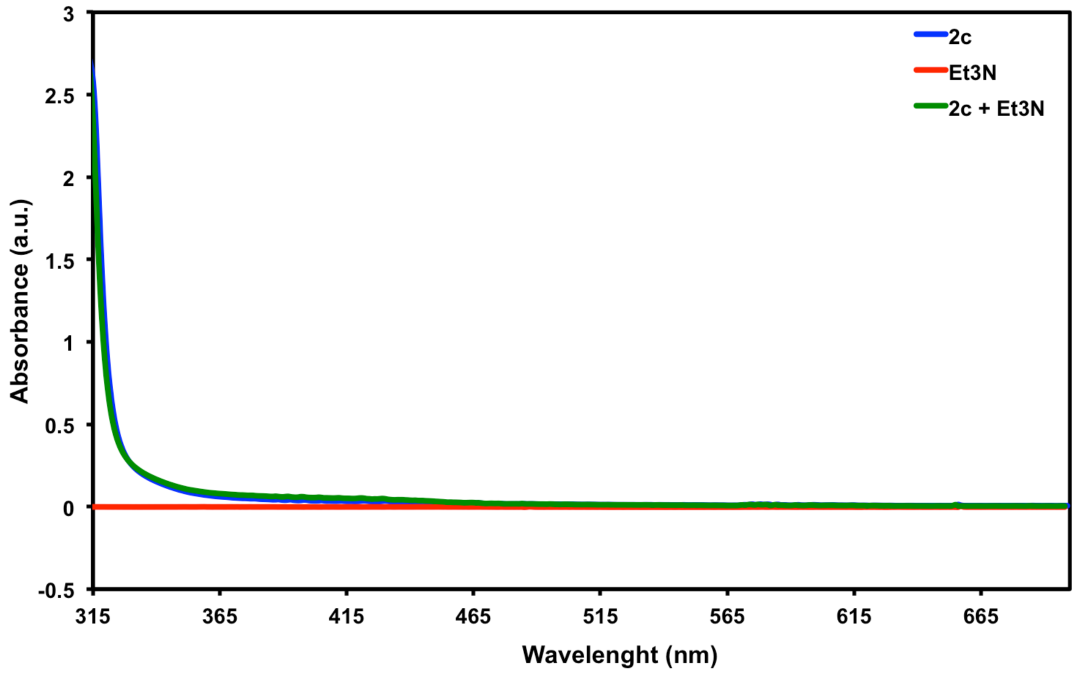

3
2.5
2
1.5
1
0.5
0
-0.5
Absorbance (a.u.)
315
365
415
465
515
565
615
665
Wavelenght (nm)
2c
Et3N
2c + Et3N

5.1.1.2 Amine screening using oxime 2a

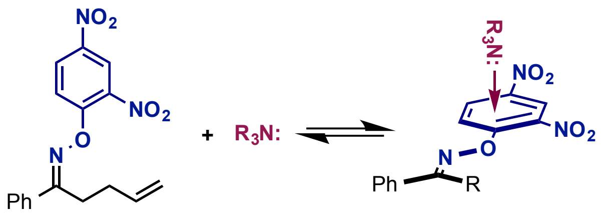

A 0.07M solution of **2a** (5 mg, 0.015 mmol, 1.0 equiv.) in CH_3CN (0.2 mL) was treated with the corresponding amine (0.029 mmol, 2.0 equiv.) and the mixture was stirred for 15 min and then analysed by UV-vis spectroscopy using a 1 mm quartz cuvette.

The amines did not absorb in the visible region.

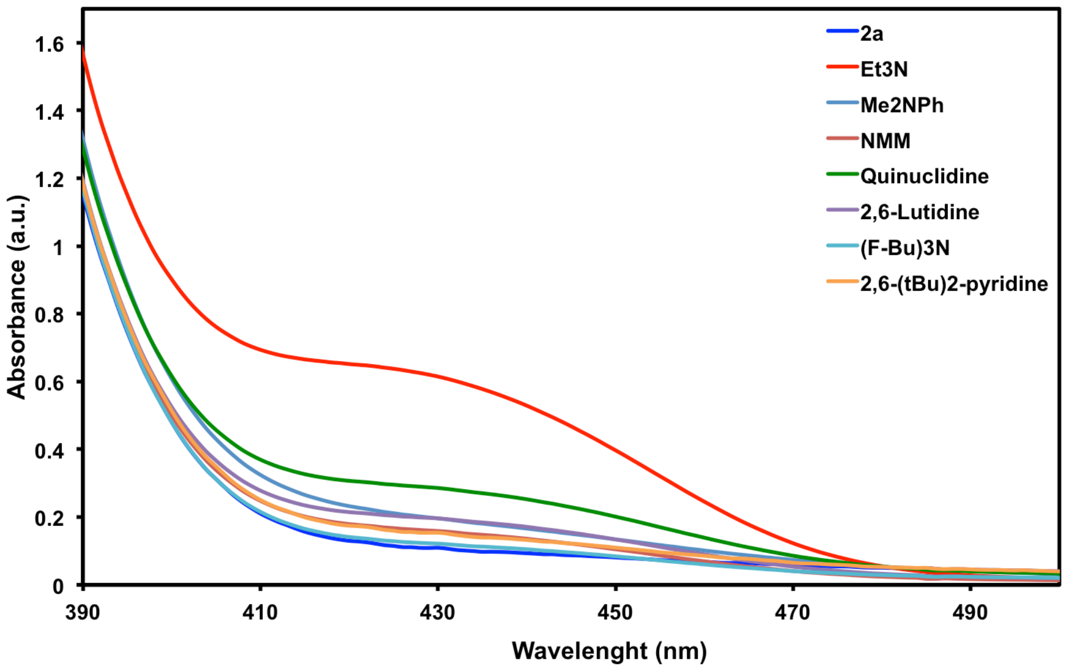

5.1.1.3 Solvents screening using oxime 2a and Et_3N

A 0.3M solution of **2a** (20 mg, 0.06 mmol, 1.0 equiv.) in the appropriate solvent (0.2 mL) was treated with Et_3N (16 μL, 0.12 mmol, 2.0 equiv.), stirred for 15 min and then analysed by UV-vis spectroscopy using a 1 mm quartz cuvette.

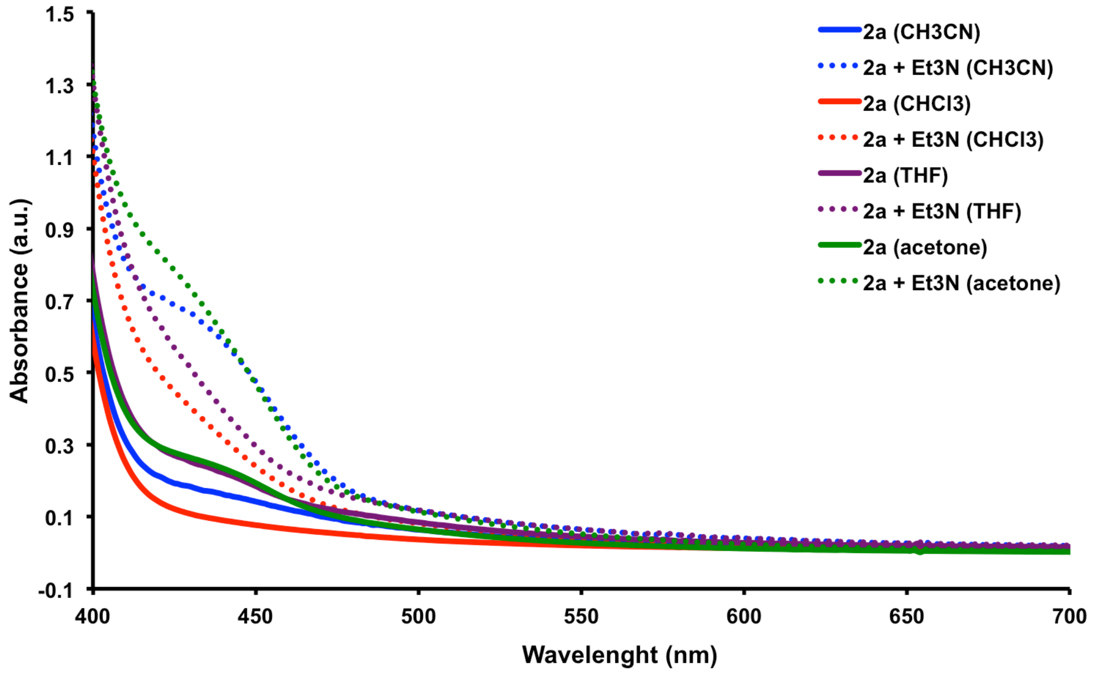

The variation in absorbance followed an apparent trend with the solvent polarity.

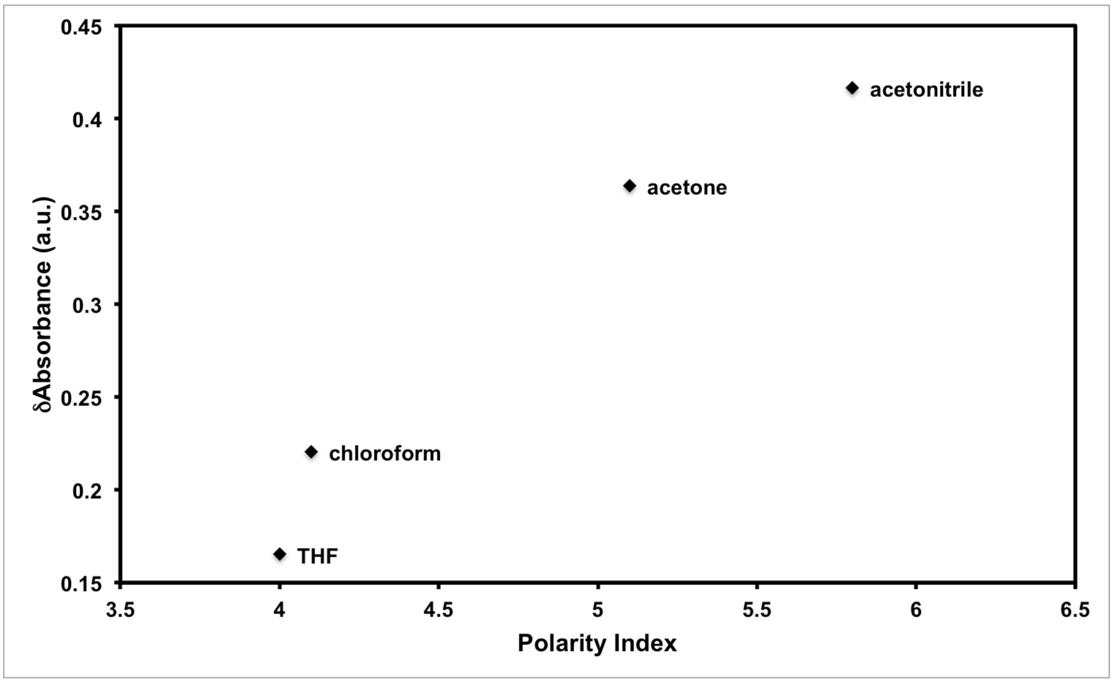

5.1.2 Binding studies

5.1.2.1 Determination of the extinction coefficient for 2a

The extinction coefficient for **2a** in CH_3CN was calculated via a calibration curve at 470 nm. The calculated extinction coefficients are:

ε_{410} = 29.5 ± 0.2 L mol^{-1} cm^{-1}

ε_{430} = 16.1 ± 0.2 L mol^{-1} cm^{-1}

ε_{450} = 11.8 ± 0.1 L mol^{-1} cm^{-1}

ε_{470} = 7.3 ± 0.3 L mol^{-1} cm^{-1}

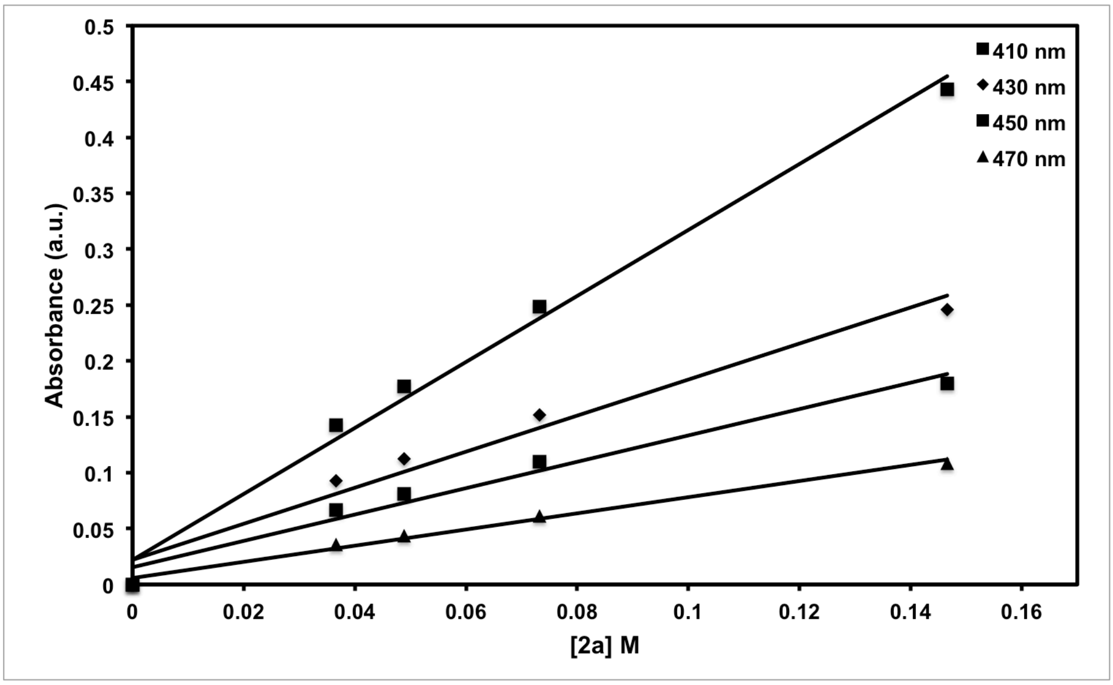

5.1.2.2 Determination of complexes stoichiometry using the Method of Continuous Variations – Job Plots for the binding of 2a with Et_3N

Two equimolar (0.3M) solution of **2a** and Et_3N in CH_3CN were prepared. Then 8 samples were prepared with defined volumes of the two stock solutions to give a total volume of 100 μL and were analysed by UV/vis spectroscopy. The observed absorbances were normalized on the concentration of **2a**.

V_{2a} (μL)	V_{Et3N} (μL)	Xi_{2a}	Xi_{Et3N}	δA^{450}_{2a}	$\Delta(\delta A^{450}_{2a})*Xi_{2a}$
100	0	1	0	0	0
80	20	0,8	0,2	1,718900649	1,375120519
60	40	0,6	0,4	3,335431226	2,001258736
50	50	0,5	0,5	4,399372037	2,199686019
40	60	0,3	0,7	4,505476951	1,351643085
20	80	0,2	0,8	5,469061533	1,093812307
10	90	0,1	0,9	6,061825752	0,606182575
0	100	0	1	0	0

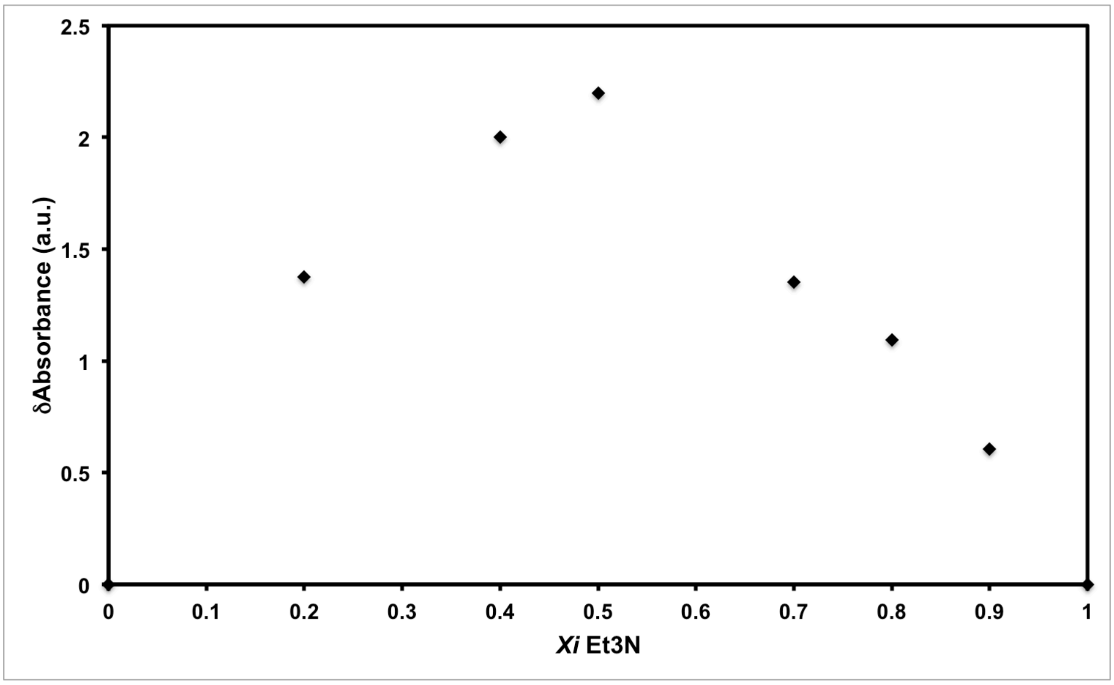

5.1.2.3 Determination of the association constant for the binding of 2a and Et_3N

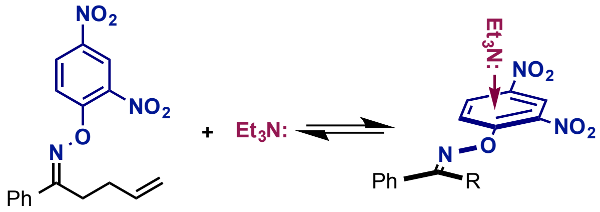

The experiments were performed via titration of the absorbance of the UV-vis trace of **2a** upon addition of Et_3N in the mixture. The binding isotherm was plotted in Origin 8.5 and a non-linear regression approach was followed to evaluate the equilibrium constant. The Rose-Drago method for UV-vis spectroscopy was used.[39] The binding studies were repeated twice.

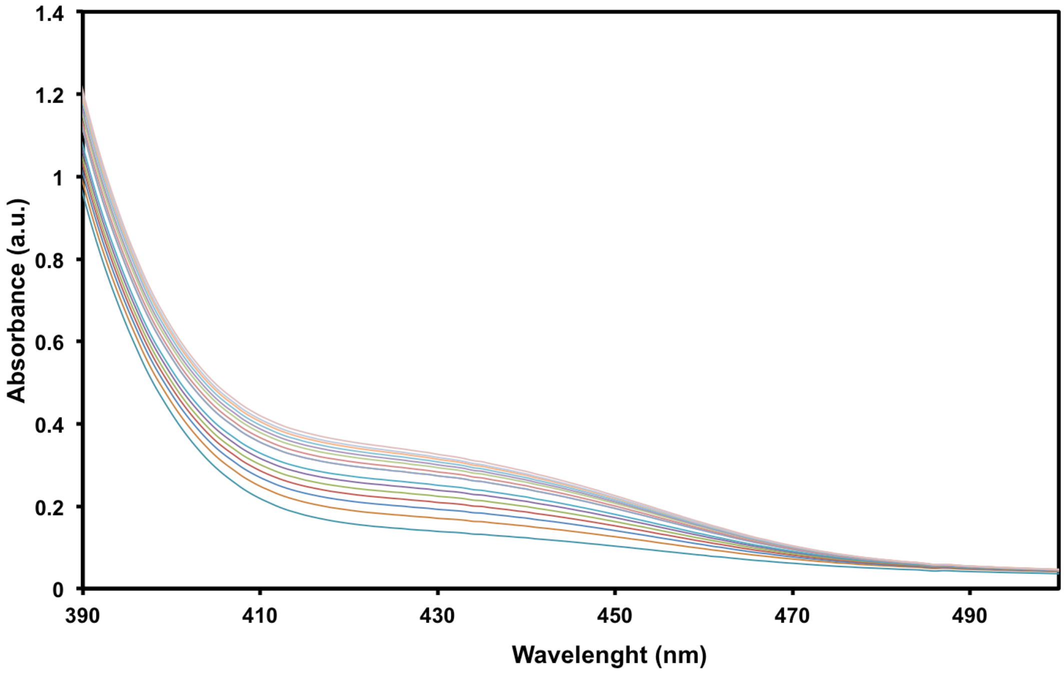

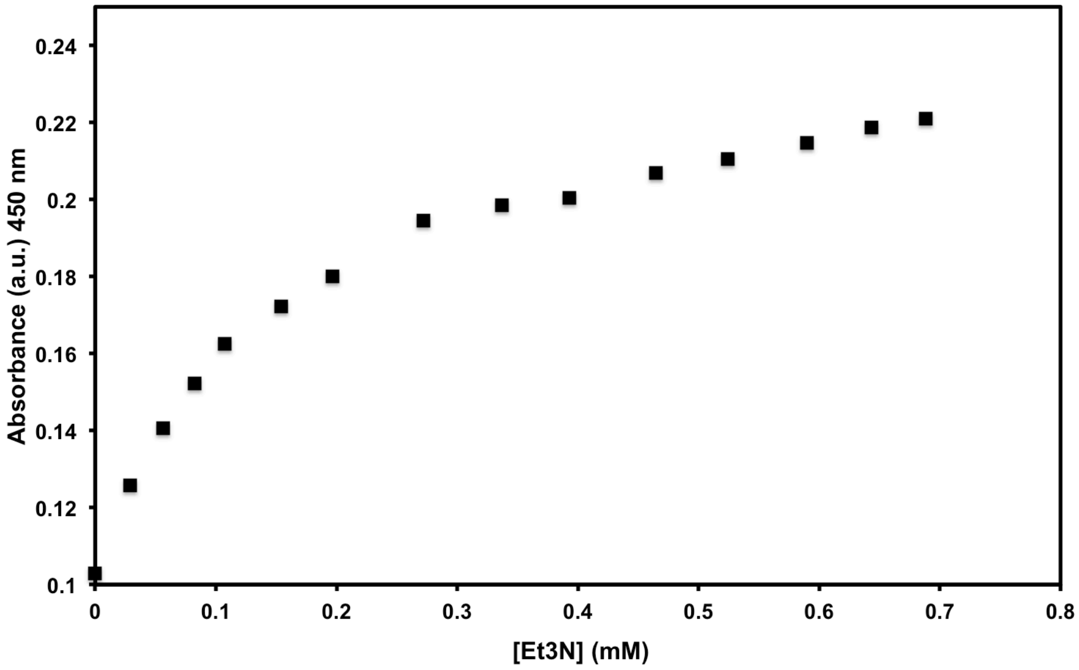

Origin report:

Nonlinear Curve Fit (Leonori2 (User)) (18/05/2015 20:22:

Input Data

		Dep/Indep	Data	Range	Weight Type
B	G0	Indep	[Book1]Sheet1!A	[1:15]	
	A	Dep	[Book1]Sheet1!B	[1:15]	No Weighting

Parameters

		Value	Standard Error
B	Eh	11.8	0
	H0	0.058	0
	Ec	38.47892	1.31652
	K	22.70356	6.64772

Reduced Chi-sqr = 1.46290040095E-4
COD(R^2) = 0.89750285515602
Iterations Performed = 1
Total Iterations in Session = 39
Fit converged. Chi-Sqr tolerance value of 1E-9 was reached.
Some parameter values were fixed.

Statistics

	B
Number of Points	15
Degrees of Freedom	13
Reduced Chi-Sqr	1.4629E-4
Residual Sum of Squares	0.0019
Adj. R-Square	0.88962
Fit Status	Succeeded(100)

Fit Status Code :
100 : Fit converged. Chi-Sqr tolerance value of 1E-9 was reached.

Summary

	Eh		H0		Ec		K		Statistics	
	Value	Standard Error	Value	Standard Error	Value	Standard Error	Value	Standard Error	Reduced Chi-Sqr	Adj. R-Square
B	11.8	0	0.058	0	38.47892	1.31652	22.70356	6.64772	1.4629E-4	0.88962

ANOVA

		DF	Sum of Squares	Mean Square	F Value	Prob>F
B	Regression	2	0.50314	0.25157	1719.6493	3.33067E-15
	Residual	13	0.0019	1.4629E-4		
	Uncorrected Total	15	0.50504			
	Corrected Total	14	0.01855			

Fitted Curves Plot

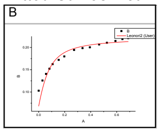

Residual vs. Independent Plot

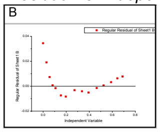

5.2 Reaction optimization

General Procedure for the reaction optimization using oxime 2a – GP11

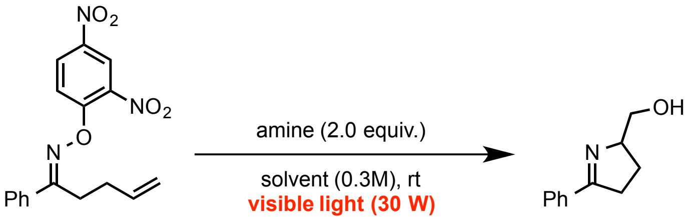

A dry tube equipped with a stirring bar was charged with **2a** (30 mg, 0.088 mmol, 1.0 equiv.), the amine (0.176 mmol, 2.0 equiv.) and the solvent (0.4 M). The mixture was stirred at room temperature overnight in front of a 30 W fluorescent household bulb. The solvent was evaporated and 1,3,5-Trimethoxybenzene (4.9 mg, 0.029 mmol, 0.33 equiv.) and $CDCl_3$ (0.4 mL) were added and the mixture was analysed by ^{1}H NMR spectroscopy to determine the NMR yield.

Entry	Amine	Solvent	Yield (%)
**1**	Et_3N	CH_3CN	95
**2**	–	CH_3CN	–
**3** [a]	Et_3N	CH_3CN	–
**4**		MeOH	43
**5**		DMF	53
**6**		Toluene	36
**7**		HFIP	25
**8**		$CHCl_3$	18
**9**	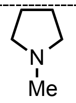	CH_3CN	25
**10**	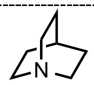	CH_3CN	32
**11**	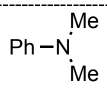	CH_3CN	12
**12**	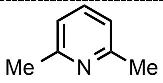	CH_3CN	–
**13**	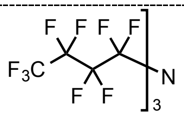	CH_3CN	–

a) the reaction was run in the dark

Under the optimized reaction conditions (entry 22), analysis of the crude after simple solvent evaporation provided the following ^{1}H NMR spectrum.

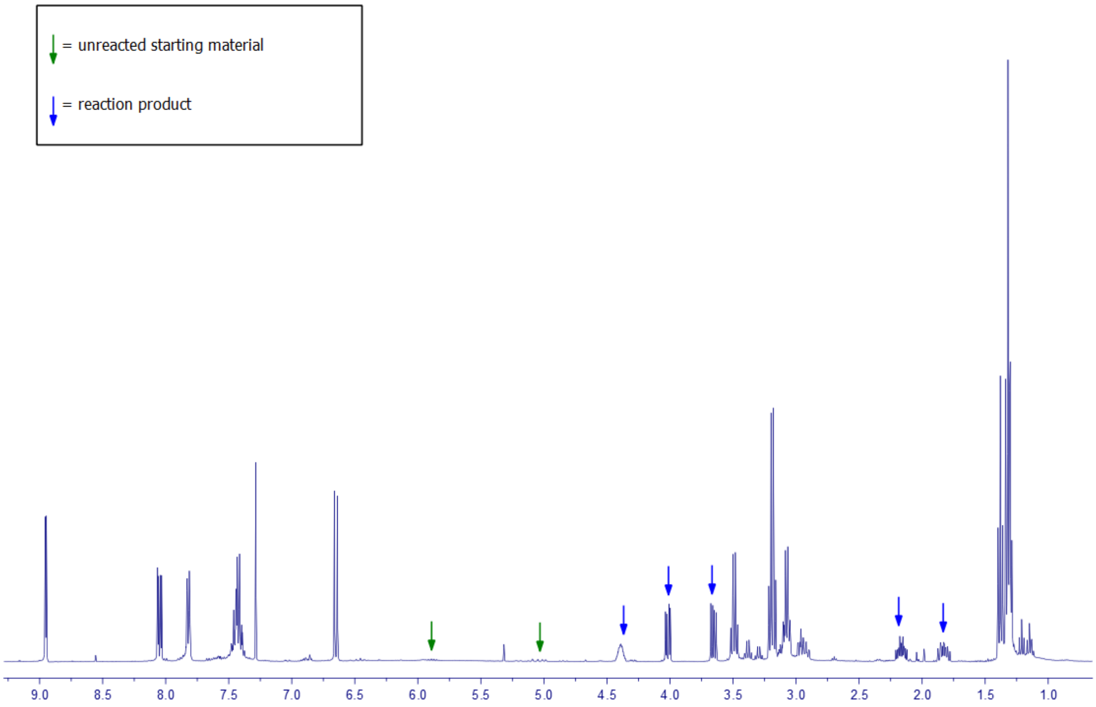

5.3 Substrate scope

General Procedure for the iminohydroxylation-cyclization reaction – GP12

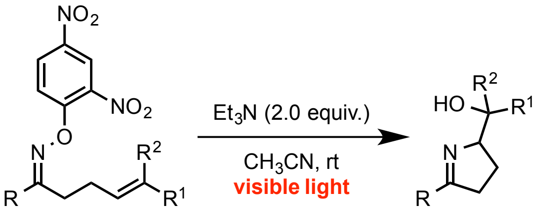

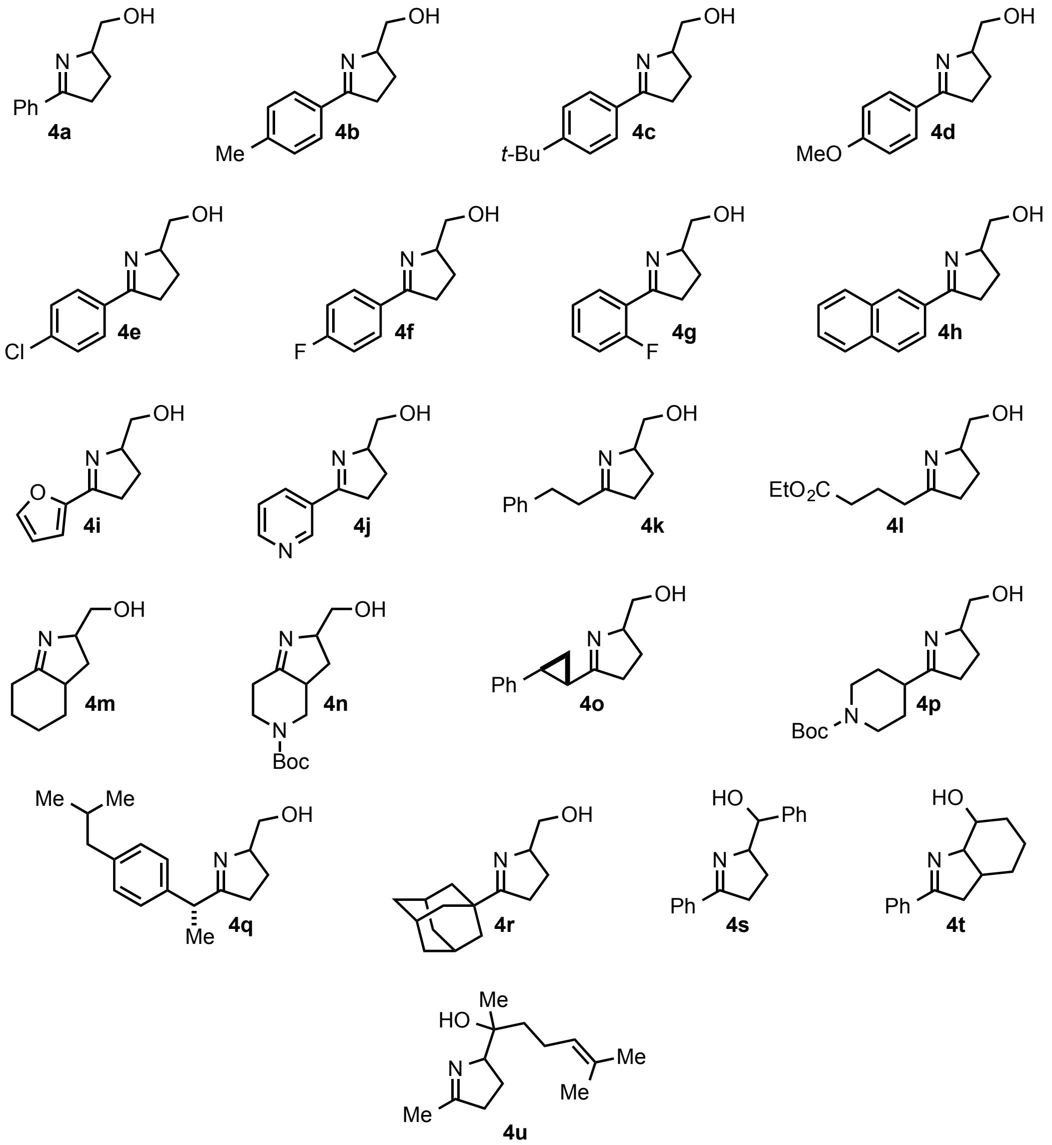

A solution of the oxime (1.0 equiv.) in CH_3CN (0.3 M) was treated with Et_3N (2 equiv.) and stirred at room temperature overnight in front of a 30 W fluorescent household bulb overnight. At the end of the reaction the volatiles were evaporated and the crude was purified by column chromatography on silica gel eluting with CH_2Cl_2:MeOH:NH_4OH (95:5:0.1) to give the product.

(5-Phenyl-3,4-dihydro-2H-pyrrol-2-yl)methanol (4a)

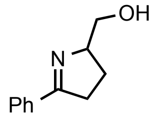

Following **GP12**, **2a** (50 mg, 0.15 mmol) gave **4a** (21 mg, 85%) as an oil. ^{1}H NMR (400 MHz, $CDCl_3$) δ 7.82 (2H, d, *J* = 6.8 Hz), 7.42–7.34 (3H, m), 4.40–4.31 (1H, m), 3.94 (1H, dd, *J* = 11.2, 4.0 Hz), 3.58 (1H, dd, *J* = 11.2, 6.1 Hz), 3.08 (1H, dddd, *J* = 14.7, 10.2, 4.5, 2.3 Hz), 2.93 (1H, ddd, *J* = 19.1, 9.7, 1.7 Hz), 2.19–2.06 (1H, m), 1.83 (1H, s, OH), 1.82–1.71 (1H, m); ^{13}C NMR (101 MHz, $CDCl_3$) δ 175.3, 131.3, 128.6, 128.1, 74.3, 66.0, 35.6, 24.4; HRMS (APCI): Found MH^+ 176.1073. $C_{11}H_{13}NO$ requires 176.1075. This compound has been described in the literature,[40] but no spectroscopic data was reported.

This reaction was scaled up to 500 mg of **2a** (1.50 mmol) and after visible light irradiation (3 days), 4a was obtained in 78% yield (204 mg).

(5-(*p*-Tolyl)-3,4-dihydro-2*H*-pyrrol-2-yl)methanol (4b)

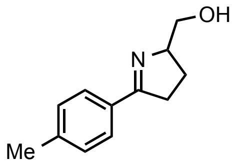

Following **GP12**, **2e** (100 mg, 0.28 mmol) gave **4b** (40 mg, 76%) as an oil. R_f 0.76 [CH_2Cl_2–MeOH (90:10)]; FT-IR ν_{max} (film)/cm^{-1} 3221, 2919, 2867, 1609, 1567, 1513, 1454, 1374, 1337, 1298, 1181, 1076; ^{1}H NMR (400 MHz, $CDCl_3$) δ 7.70 (2H, d, *J* = 8.2 Hz), 7.20 (2H, d, *J* = 7.9 Hz), 4.40–4.31 (1H, m), 3.99 (1H, dd, *J* = 11.1, 4.0 Hz), 3.62 (1H, dd, *J* = 11.1, 6.0 Hz), 3.04 (1H, dddd, *J* = 17.1, 10.3, 4.5, 2.3 Hz), 2.95–2.83 (1H, m), 2.38 (3H, s), 2.19–2.07 (1H, m), 1.86–1.72 (1H, m); ^{13}C NMR (101 MHz, $CDCl_3$) δ 174.5, 141.1, 131.6, 129.3, 127.9, 74.8, 66.3, 35.7, 24.6, 21.6; HRMS (APCI) Found MH^+ 190.1226. $C_{12}H_{16}NO$ requires 190.1232.

(5-(4-(*tert*-Butyl)phenyl)-3,4-dihydro-2*H*-pyrrol-2-yl)methanol (4c)

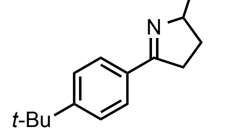

Following **GP12**, **2f** (75 mg, 0.19 mmol) gave **4c** (32 mg, 73%) as an oil. R_f 0.68 [CH_2Cl_2–MeOH (90:10)]; FT-IR ν_{max} (film)/cm^{-1} 3219, 2960, 2906, 2867, 1612, 1562, 1475, 1405, 1337, 1268, 1110, 1045; ^{1}H NMR (400 MHz, $CDCl_3$) δ 7.71 (2H, d, *J* = 8.6 Hz), 7.40 (2H, d, *J* = 8.6 Hz), 4.40–4.30 (1H, m), 4.02 (1H, dd, *J* = 11.2, 3.9 Hz), 3.62 (1H, dd, *J* = 11.2, 5.7 Hz), 3.03 (1H, dddd, *J* = 17.1, 10.3, 4.6, 2.3 Hz), 2.94–2.82 (1H, m), 2.60 (1H, s, OH), 2.11

(1H, dddd, J = 12.8, 10.0, 8.2, 4.6 Hz), 1.88–1.74 (1H, m); ^{13}C NMR (101 MHz, CDCl$_3$) δ 174.4, 154.2, 131.5, 127.7, 125.4, 74.8, 66.2, 35.7, 35.0, 31.3, 24.5; HRMS (APCI): Found MH$^+$ 232.169 $C_{15}H_{21}NO$ requires 232.1701.

(5-(4-Methoxyphenyl)-3,4-dihydro-2*H*-pyrrol-2-yl)methanol (4d)

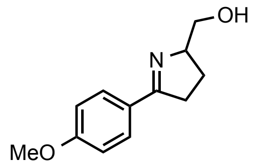

Following **GP12**, **2g** (50 mg, 0.14 mmol) gave **4d** (17 mg, 61%) as an oil. R$_f$ 0.48 [CH_2Cl_2–MeOH (90:10)]; FT-IR ν_{max} (film)/cm^{-1} 3227, 3059, 2922, 1632, 1559, 1335, 1136, 1061; ^{1}H NMR (400 MHz, CDCl$_3$) δ 7.75 (2H, d, J = 8.9 Hz), 6.90 (2H, d, J = 8.9 Hz), 4.39–4.29 (1H, m), 4.03 (1H, dd, J = 11.2, 3.9 Hz), 3.86 (3H, s), 3.64 (1H, dd, J = 11.2, 5.7 Hz), 3.01 (1H, dddd, J = 17.0, 10.3, 4.5, 2.2 Hz), 2.94–2.79 (1H, m), 2.19–2.06 (1H, m), 1.90–1.76 (1H, m); ^{13}C NMR (101 MHz, CDCl$_3$) δ 173.82, 161.64, 129.6, 127.1, 113.8, 74.7, 66.0, 55.5, 35.7, 24.5; HRMS (APCI): Found MH$^+$ 206.1181 $C_{12}H_{16}O_2N$ requires 206.1181.

(5-(4-Chlorophenyl)-3,4-dihydro-2*H*-pyrrol-2-yl)methanol (4e)

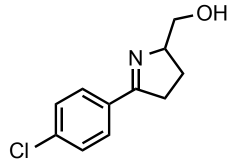

Following **GP12**, **2h** (75 mg, 0.20 mmol) gave **4e** (33 mg, 78%) as an oil. R$_f$ 0.59 [CH_2Cl_2–MeOH (90:10)]; FT-IR ν_{max} (film)/cm^{-1} 3175, 2924, 2886, 1623, 1593, 1488, 1454, 1425, 1401, 1364, 1337, 1301, 1091, 1080; ^{1}H NMR (400 MHz, CDCl$_3$) δ 7.63 (2H, d, J = 8.6 Hz), 7.27 (2H, d, J = 8.7 Hz), 4.28 (1H, br s), 3.96 (1H, dd, J = 11.3, 3.9 Hz), 3.56 (1H, dd, J = 11.3, 5.7 Hz), 2.91 (1H, ddd, J = 10.3, 4.5, 2.3 Hz), 2.81 (1H, ddd, J = 9.8, 7.8, 2.0 Hz), 2.13–2.00 (1H, m), 1.83–1.69 (1H, m); ^{13}C NMR (101 MHz, CDCl$_3$) δ 173.3, 136.7, 132.5, 129.1, 128.6, 74.9, 65.8, 35.6, 24.4; HRMS (EI): Found MH$^+$ 210.0682 $C_{11}H_{13}ClON$ requires 210.0686.

(5-(4-Fluorophenyl)-3,4-dihydro-2*H*-pyrrol-2-yl)methanol (4f)

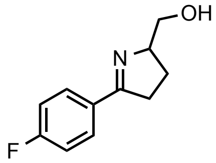

Following **GP12**, **2i** (100 mg, 0.28 mmol) gave **4f** (41 mg, 75%) as an oil. R$_f$ 0.59 [CH_2Cl_2–MeOH (90:10)]; FT-IR ν_{max} (film)/cm^{-1} 3221, 2926, 2869, 1617, 1509, 1455, 1316, 1224,

1156, 1129, 1048; ^{1}H NMR (400 MHz, $CDCl_3$) δ 7.75 (2H, dd, *J* = 8.9, 5.5 Hz), 7.05 (2H, dd, *J* = 8.9, 8.6 Hz), 4.34 (1H, br s), 4.07 (1H, dd, *J* = 11.4, 3.7 Hz), 3.65 (1H, dd, *J* = 11.4, 5.4 Hz), 3.03 (1H, br s, OH), 2.99 (1H, dddd, *J* = 17.1, 10.3, 4.5, 2.3 Hz), 2.85 (1H, dddd, *J* = 17.3, 9.8, 7.7, 2.0 Hz), 2.13 (1H, dddd, *J* = 12.8, 10.0, 8.2, 4.5 Hz), 1.87 (1H, dddd, *J* = 12.8, 10.3, 7.8, 7.2 Hz); ^{13}C NMR (101 MHz, $CDCl_3$) δ 173.5, 164.4 (d, *J* = 251.4 Hz), 130.9 (d, *J* = 75.7 Hz), 130.0 (d, *J* = 8.6 Hz), 115.5 (d, *J* = 21.8 Hz), 74.7, 65.7, 35.7, 29.7, 24.4; ^{19}F NMR (376 MHz, $CDCl_3$, decoupled) δ –112.3; HRMS (EI): Found MH^+ 194.0987 $C_{11}H_{13}FNO$ requires 194.0981.

(5-(2-Fluorophenyl)-3,4-dihydro-2H-pyrrol-2-yl)methanol (4g)

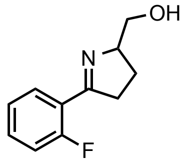

Following **GP12**, **2j** (100 mg, 0.28 mmol) gave **4g** (32 mg, 59%) as an oil. R_f 0.68 [CH_2Cl_2–MeOH (90:10)]; FT-IR ν_{max} (film)/cm^{-1} 3147, 2938, 2868, 1606, 1487, 1457, 1339, 1299, 1212, 1109, 1085; ^{1}H NMR (400 MHz, $CDCl_3$) δ 7.85 (1H, td, *J* = 7.7, 1.8 Hz), 7.32 (1H, dddd, *J* = 8.3, 7.1, 5.1, 1.8 Hz), 7.09 (1H, td, *J* = 7.8, 1.1 Hz), 7.00 (1H, ddd, *J* = 11.4, 8.3, 1.0 Hz), 4.23 (1H, br s), 3.90 (1h, dd, *J* = 11.2, 4.0 Hz), 3.56 (1H, dd, *J* = 11.2, 6.1 Hz), 3.32 (1H, br s, OH), 3.11–2.83 (2H, m), 2.12–1.98 (1H, m), 1.71 (1H, ddt, *J* = 12.8, 10.1, 7.7 Hz); ^{13}C NMR (101 MHz, $CDCl_3$) δ 171.9, 162.7 (d, *J* = 225.4 Hz), , 132.3 (d, *J* = 8.8 Hz), 130.2 (d, *J* = 3.6 Hz), 124.2 (d, *J* = 3.4 Hz), 122.5 (d, *J* = 7.8 Hz), , 116.4 (d, *J* = 22.8 Hz), 73.8, 65.9, 38.5, 29.7, 24.8; ^{19}F NMR (376 MHz, $CDCl_3$, decoupled) δ –112.7; HRMS (EI) found: 194.0978 $C_{12}H_{13}FNO$ requires 194.0980.

(5-(Naphthalen-2-yl)-3,4-dihydro-2*H*-pyrrol-2-yl)methanol (4h)

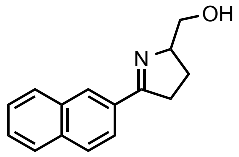

Following **GP12**, **2k** (50 mg, 0.13 mmol) gave **4h** (18 mg, 63%) as an oil. R_f 0.59 [CH_2Cl_2–MeOH (90:10)]; FT-IR ν_{max} (film)/cm^{-1} 3252, 3056, 2923, 1611, 1526, 1454, 1351, 1264, 1191, 1046; ^{1}H NMR (400 MHz, $CDCl_3$) δ 8.11–8.07 (2H, m), 7.91–7.84 (3H, m), 7.58–7.51 (2H, m), 4.46 (1H, br s), 4.10 (1H, dd, *J* = 11.2, 4.0 Hz), 3.71 (1H, dd, *J* = 11.2, 5.9 Hz), 3.25–3.17 (1H, m), 3.05 (1H, dddd, *J* = 17.2, 9.7, 7.7, 1.9 Hz), 2.27–2.18 (1H, m), 1.96–1.86 (1H, m); ^{13}C NMR (101 MHz, $CDCl_3$) δ 174.6, 134.5, 132.9, 131.4, 128.81, 128.65, 128.2,

127.8, 127.3, 126.5, 124.5, 74.8, 66.1, 35.7, 24.5; HRMS (EI): Found MH^+ 226.1243 $C_{15}H_{16}ON$ requires 226.1232.

(5-(Furan-2-yl)-3,4-dihydro-2*H*-pyrrol-2-yl)methanol (4i)

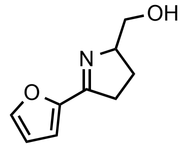

Following **GP12**, **2l** (75 mg, 0.19 mmol) gave **4i** (16 mg, 50%) as an oil. R_f 0.54 [CH_2Cl_2–MeOH (90:10)]; FT-IR ν_{max} (film)/cm^{-1} 3224, 3041, 2937, 1612, 1473, 1337, 1264, 1033; ^1H NMR (400 MHz, $CDCl_3$) δ 7.59 (1H, d, *J* = 1.5 Hz), 6.96 (1H, br d, *J* = 3.5 Hz), 6.54 (1H, dd, *J* = 3.5, 1.8 Hz), 4.41 (1H, br s), 4.04 (1H, dd, *J* = 11.3, 3.8 Hz), 3.69 (1H, dd, *J* = 11.3, 5.7 Hz), 3.11–3.02 (1H, m), 2.93 (1H, dddd, *J* = 17.4, 9.8, 7.9, 1.9 Hz), 2.22–2.13 (2H, m & OH), 1.85 (1H, dddd, *J* = 12.9, 10.3, 8.0, 7.2 Hz); ^{13}C NMR (101 MHz, $CDCl_3$) δ 161.1, 145.4, 114.96, 114.93, 112.0, 74.5, 65.7, 35.4, 24.1; HRMS (EI): Found MH^+ 166.0867 $C_9H_{12}O_2N$ requires 166.0868.

(5-Pyridin-3-yl)-3,4-dihydro-2*H*-pyrrol-2-yl)methanol (4j)

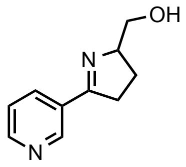

Following **GP12**, **2m** (100 mg, 0.29 mmol) gave **4j** (23 mg, 45%) as an oil. R_f 0.34 [CH_2Cl_2–MeOH (90:10)]; FT-IR ν_{max} (film)/cm^{-1} 3224, 3041, 2937, 1612, 1473, 1337, 1264, 1033; ^1H NMR (400 MHz, $CDCl_3$) δ 8.88 (1H, d, *J* = 1.6 Hz), 8.58 (1H, dd, *J* = 4.8, 1.6 Hz), 8.07 (1H, dt, *J* = 8.0, 2.0 Hz), 7.27 (1H, ddd, *J* = 8.0, 4.8, 0.8 Hz), 4.32 (1H, br s), 3.96 (1H, dd, *J* = 11.2, 3.9 Hz), 3.59 (1H, dd, *J* = 11.2, 5.9 Hz), 3.01 (1H, dddd, *J* = 17.2, 10.3, 4.5, 2.4 Hz), 2.86 (1H, dddd, *J* = 17.3, 9.8, 7.7, 2.1 Hz), 2.11 (1H, dddd, *J* = 12.9, 10.0, 8.2, 4.6 Hz), 2.07–2.06 (1H, m), 1.79 (1H, dddd, *J* = 12.9, 10.3, 7.7, 7.1 Hz); ^{13}C NMR (101 MHz, $CDCl_3$) δ 172.1, 151.4, 149.2, 134.9, 129.8, 123.4, 75.0, 65.9, 35.5, 24.4; LRMS *m/z* (ESI): 176.1 (M^+).

(5-Phenethyl-3,4-dihydro-2*H*-pyrrol-2-yl)methanol (4k)

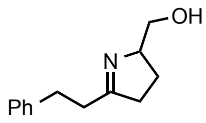

Following **GP12**, **2n** (100 mg, 0.27 mmol) gave **4k** (40 mg, 73%) as an oil. R_f 0.64 [CH_2Cl_2–MeOH (90:10)]; FT-IR ν_{max} (film)/cm^{-1} 3228, 2924, 1641, 1494, 1426, 1373, 1070, 1046; ^1H NMR (400 MHz, $CDCl_3$) δ 7.27–7.18 (3H, m), 7.18–7.09 (2H, m), 4.0.7 (1H, br s), 3.78 (1H,

dd, J = 11.2, 3.9 Hz), 3.42 (1H, dd, J = 11.1, 5.6 Hz), 2.94–2.79 (2H, m), 2.66–2.56 (2H, m), 2.54–2.31 (2H, m), 1.90 (1H, dddd, J = 13.0, 9.8, 8.1, 4.9 Hz), 1.57 (1H, dddd, J = 12.9, 10.1, 7.6, 6.8 Hz); ^{13}C NMR (101 MHz, $CDCl_3$) δ 179.5, 141.1, 128.51, 128.31, 126.2, 74.0, 65.9, 38.2, 35.3, 32.6, 24.4; HRMS (EI): Found M^+ 203.1297 $C_{13}H_{17}NO$ requires 203.1305.

Ethyl 4-(2-(Hydroxymethyl)-3,4-dihydro-2*H*-pyrrol-5-yl)butanoate (4l)

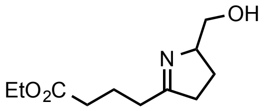

Following **GP12**, **2o** (100 mg, 0.26 mmol) gave **4l** (32 mg, 57%) as an oil. R_f 0.66 [CH_2Cl_2–MeOH (95:5)]; ^{1}H NMR (400 MHz, $CDCl_3$) δ 4.21–4.10 (1H, m), 4.14 (2H, q, J = 7.1 Hz), 3.88 (1H, dd, J = 11.2, 3.4 Hz), 3.54 (1H, dd, J = 11.2, 5.9 Hz), 2.65–2.46 (3H, m,), 2.45–2.34 (4H, m), 2.04–1.90 (3H, m), 1.65 (1H, dddd, J = 12.9, 10.2, 7.9, 7.0 Hz), 1.27 (3H, t, J = 7.1 Hz); ^{13}C NMR (101 MHz, $CDCl_3$) δ 179.2, 173.3, 74.1, 65.9, 60.4, 37.9, 33.8, 32.9, 24.5, 21.6, 14.3; HRMS (EI): Found M^+ 213.1367 $C_{11}H_{19}NO_3$ requires 213.1365.

(3,3a,4,5,6,7-Hexahydro-2*H*-indol-2-yl)methanol (4m)

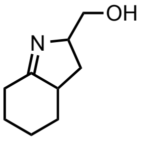

Following **GP12**, **2q** (75 mg, 0.23 mmol) gave **4m** (17 mg, 47%) as an oil; dr 1.5:1. R_f 0.55 [CH_2Cl_2–MeOH (90:10)]; FT-IR ν_{max} (film)/cm^{-1} 3251, 2932, 2859, 1650, 1447, 1265, 1197, 1049, 1007; ^{1}H NMR (400 MHz, $CDCl_3$) δ 4.22 (0.4H, br s), 4.02 (0.6H, br s), 3.92 (0.6H, dd, J = 11.2, 3.8 Hz), 3.79 (0.4H, dd, J = 11.2, 3.9 Hz), 3.54 (0.6H, ddd, J = 11.2, 7.4), 3.52 (0.4H, dd, J = 11.2, 6.0 Hz), 2.88 (1H, br s, OH), 2.75–2.60 (2H, m), 2.26–2.11 (2H, m), 2.07–1.90 (2H, m), 1.85–1.80 (1H, m), 1.65–1.52 (1H, m), 1.51–1.39 (2H, m), 1.26–1.12 (2H, m); ^{13}C NMR (101 MHz, $CDCl_3$) δ 182.2 & 181.5, 72.5 & 72.4, 66.1 & 66.0, 48.6, 35.1 & 34.7, 31.9 & 31.8, 31.7 & 31.5, 27.2 & 26.6, 25.4 & 25.1; HRMS (EI): Found MH^+ 154.1226 $C_9H_{16}ON$ requires 154.1232.

This reaction was repeated on the same scale using blue LEDs (16 h irradiation) and 4m was isolated in an improved 57% yield.

***tert*-Butyl 2-(Hydroxymethyl)-2,3,3a,4,6,7-hexahydro-5*H*-pyrrolo[3,2-*c*]pyridine-5-carboxylate (4n)**

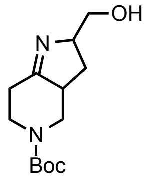

Following **GP12**, **2r** (100 mg, 0.24 mmol) gave **4n** (31 mg, 52%) as an oil, dr 3:2. R_f 0.39 [CH_2Cl_2–MeOH (95:5)]; ^{1}H NMR (400 MHz, $CDCl_3$, diastereomers and rotamers) δ 4.57–4.24 (2H, m), 4.21 (0.3H, br s), 4.01 (0.7H, br s), 3.89 (0.7H, br d, J = 11.2 Hz), 3.71 (0.3H, br d, J = 10.1 Hz), 3.48 (1H, dd, J = 11.0, 5.6 Hz), 2.84–2.60 (2H, m), 2.55–2.50 (1.3H, m), 2.42–2.23 (2H, m), 2.10–2.01 (0.7H, m), 1.85 (0.3H, br t, J = 10.6 Hz), 1.54-1.45 (0.7H, m), 1.41 (9H, s), 1.18–1.11 (m, 1H); ^{13}C NMR (101 MHz, $CDCl_3$, diastereomers and rotamers) δ 178.5 & 177.9, 154.4, 80.3 & 73.1, 65.85 & 65.8, 65.55 & 65.5, 50.3 (br), 47.8 (br) & 47.6 (br), 44.9 (br) & 43.7 (br), 31.9 (br), 28.4, 28.2; HRMS (EI): Found M^+ 254.1363 $C_{13}H_{22}N_2O_3$ requires 254.1360.

(5-(2-Phenylcyclopropyl)-3,4-dihydro-2*H*-pyrrol-2-yl)methanol (4o)

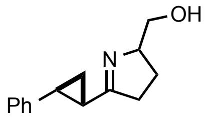

Following **GP12**, **2s** (100 mg, 0.26 mmol) gave **4o** (48 mg, 81%) as an oil; dr 1:1. R_f 0.65 [CH_2Cl_2–MeOH (90:10)]; FT-IR ν_{max} (film)/cm^{-1} 3000, 2917, 1629, 1603, 1496, 1457, 1345, 1265, 1076, 1044; ^{1}H NMR (400 MHz, $CDCl_3$, diastereomers) δ 7.21–7.17 (2H, m), 7.13–7.08 (1H, m), 7.03–7.00 (2H, m), 4.04 (1H, br s), 3.80 (0.5H, dd, J = 11.3, 3.8 Hz), 3.77 (0.5H, dd, J = 11.3, 3.9 Hz), 3.46 (0.5H, dd, J = 11.1, 5.4 Hz), 3.43 (0.5H, dd, J = 11.3, 5.7 Hz), 2.49–2.35 (2H, m), 2.33–2.25 (1H, m), 1.99–1.85 (2H, m), 1.63–1.53 (1H, m), 1.49–1.40 (1H, m), 1.25–1.20 (1H, m); ^{13}C NMR (101 MHz, $CDCl_3$, diastereomers) δ 179.75 & 179.7, 141.2 & 141.1, 128.4, 126.25 & 126.2, 126.0 & 125.9, 74.0, 65.8 & 65.6, 36.4 & 36.3, 26.1 & 25.9, 25.7 & 25.6, 24.4 & 24.3, 17.45 & 17.4; HRMS (EI): Found M^+ 215.1311 $C_{14}H_{17}NO$ requires 215.1310.

***tert*-Butyl 4-(2-(Hydroxymethyl)-3,4-dihydro-2*H*-pyrrol-5-yl)piperidine-1-carboxylate (4p)**

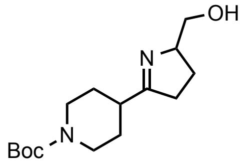

Following **GP12**, **2t** (75 mg, 0.17 mmol) gave **4o** (32 mg, 67%) as an oil; dr 1:1. R_f 0.65 [CH_2Cl_2–MeOH (90:10)]; ^{1}H NMR (400 MHz, $CDCl_3$) δ 4.16 (3H, br s), 3.86 (1H, dd, *J* = 11.1, 4.1 Hz), 3.54 (1H, dd, *J* = 11.1, 6.1 Hz), 2.79 (2H, br t, *J* = 12.2 Hz), 2.65–2.56 (1H, m), 2.55–2.44 (2H, m), 2.23–2.06 (1H, br s, OH), 2.00 (1H, dddd, *J* = 12.9, 9.7, 8.1, 4.7 Hz), 1.89–1.78 (2H, m), 1.67–1.51 (3H, m), 1.47 (9H, s); ^{13}C NMR (101 MHz, $CDCl_3$) δ 181.9, 154.8, 79.5, 73.9, 66.1, 43.9, 40.6, 35.7, 29.4, 28.5, 24.3; HRMS (EI): Found M^+ 282.1950 $C_{15}H_{26}N_2O_3$ requires 282.1943.

(5-(1-(4-Isobutylphenyl)ethyl)-3,4-dihydro-2*H*-pyrrol-2-yl)methanol (4q)

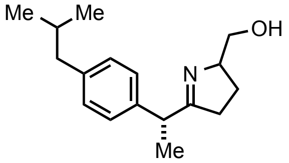

Following **GP12**, **2u** (100 mg, 0.23 mmol) gave **4q** (26 mg, 43%) as an oil; dr 1:1. R_f 0.56 [CH_2Cl_2–MeOH (90:10)]; FT-IR ν_{max} (film)/cm^{-1} 3352, 2952, 2925, 2867, 1636, 1454, 1382, 1465, 1312, 1139, 1066; ^{1}H NMR (400 MHz, $CDCl_3$, diastereomer) δ 7.32–7.26 (2H, m), 7.10–7.01 (2H, m), 4.16 (0.5H, br s), 4.10 (0.5H, br s), 3.80 (0.5H, dd, *J* = 11.3, 4.0 Hz), 3.78 (0.5H, dd, *J* = 11.3, 4.0 Hz), 3.48 (0.5H, dd, *J* = 11.1, 6.4 Hz), 3.44 (0.5H, dd, *J* = 11.1, 6.5 Hz), 2.61–2.52 (0.5H, m), 2.51–2.42 (0.5H, m), 2.39 (1H, br s), 2.37 (1H, br s), 2.36–2.27 (0.5H, m), 2.26–2.18 (0.5H, m), 2.06–1.99 (0.5H, m), 1.98–1.91 (0.5H, m), 1.86–1.74 (2H, m), 1.69 (3H, d, *J* = 5.3 Hz), 1.67–1.63 (0.5H, m), 1.58–1.50 (0.5H, m), 0.82 (6H, d, *J* = 6.6 Hz); ^{13}C NMR (101 MHz, $CDCl_3$) δ 184.1 & 184.0, 141.0, 140.9, 129.2 & 129.15, 125.35 & 125.3, 74.5, 73.2 & 73.15, 66.1 & 66.0, 45.0, 33.6, 30.25 & 30.2, 26.3 & 26.0, 25.85 & 25.81, 22.4; HRMS (EI): Found M^+ 259.1939 $C_{17}H_{25}NO$ requires 259.1936.

(5-((3*r*,5*r*,7*r*)-Adamantan-1-yl)-3,4-dihydro-2*H*-pyrrol-2-yl)methanol (4r)

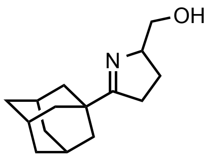

Following **GP12**, **2v** (50 mg, 0.12 mmol) gave **4r** (20 mg, 68%) as an oil. R_f 0.46 [CH_2Cl_2–MeOH (90:10)]; FT-IR ν_{max} (film)/cm^{-1} 3281, 2901, 2847, 1625, 1450, 1343, 1302, 1100,

1073; ^{1}H NMR (400 MHz, $CDCl_3$) δ 4.16 (1H, m), 3.88 (1H, dd, *J* = 11.0, 4.1 Hz), 3.52 (1H, dd, *J* = 11.0, 5.9 Hz), 2.67 (1H, dddd, *J* = 17.3, 10.2, 4.7, 2.1 Hz), 2.56–2.47 (1H, m), 2.20 (1H, s, OH), 2.08–2.03 (3H, m), 2.01–1.92 (2H, m), 1.87 (1H, br s), 1.83 (5H, m), 1.80–1.79 (1H, m), 1.78-1.76 (3H, m), 1.75–1.73 (2H, m), 1.71–1.70 (1H, mz), 1.59 (1H, dddd, *J* = 12.8, 10.1, 7.8, 6.9 Hz); ^{13}C NMR (101 MHz, $CDCl_3$) δ 187.1, 73.5, 66.2, 40.4, 38.2, 36.8, 33.2, 28.2, 24.3; HRMS (EI): Found MH^+ 233.1779 $C_{15}H_{23}NO$ requires 233.1780.

Phenyl(5-phenyl-3,4-dihydro-2*H*-pyrrol-2-yl)methanol (4s)

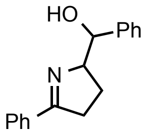

Following **GP12**, **2w** (60 mg, 0.14 mmol) gave **4s** (18 mg, 51%) as an oil; dr 1:1. R_f 0.65 [CH_2Cl_2–MeOH (90:10)]; FT-IR ν_{max} (film)/cm^{-1} 3175, 3057, 2872, 1611, 1574, 1494, 1447, 1341, 1196, 1108, 1025; ^{1}H NMR (400 MHz, $CDCl_3$, diastereomers) δ 7.91 (1H, dd, *J* = 8.0, 1.6 Hz), 7.84 (1H, dd, *J* = 8.2, 1.5 Hz), 7.53–7.35 (8H, m), 5.47 (0.5H, d, *J* = 3.1 Hz), 4.62–4.57 (0.5H, m), 4.48 (0.5H, d, *J* = 8.6 Hz), 4.44–4.38 (0.5H, m), 3.12–3.03 (0.5H, m), 3.02–2.96 (0.5H, m), 2.94–2.89 (0.5H, m), 2.88–2.81 (0.5H, m), 2.04–1.98 (0.5H, m), 1.94–1.87 (0.5H, m), 1.80–1.73 (0.5H, m), 1.70–1.64 (0.5H, m); ^{13}C NMR (101 MHz, $CDCl_3$, diastereomer) δ 175.5 & 174.1, 141.6 & 141.0, 134.1 & 134.05, 130.8 & 130.75, 128.5 & 128.45, 128.4 & 128.2, 128.0 &127.9, 127.2 & 127.1, 126.0, 79.8 & 79.1, 79.1 & 74.3, 35.7 & 35.6, 25.3 & 21.4; LRMS *m/z* (ESI): 251.1 (M^+).

2-Phenyl-3a,4,5,6,7,7a-hexahydro-3*H*-indol-7-ol (4t)

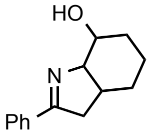

Following **GP12**, **2y** (60 mg, 0.16 mmol) gave **4t** (22 mg, 66%) as an oil; dr 3:1. R_f 0.60 [CH_2Cl_2–MeOH (90:10)]; FT-IR ν_{max} (film)/cm^{-1} 3319, 2928, 2855, 1602, 1572, 1519, 1494, 1446, 1338, 1256, 1124, 1017; ^{1}H NMR (400 MHz, $CDCl_3$, diastereomer) δ 7.89–7.83 (2H, m), 7.48–7.42 (3H, m), 4.15-4.12 (0.25H, m), 3.99–3.96 (0.75H, m), 3.96–3.92 (0.25H, m), 3.76–3.71 (0.75H, m), 3.04–2.93 (1H, m), 2.88-2.81 (0.25H, m), 2.75-2.70 (0.75H, m), 2.69-2.63 (1H, m), 2.62–2.58 (0.25H, m), 2.57–2.47 (0.75H, m), 2.37 (1H, br s, OH), 1.96-1.88 (0.25H, m), 1.88–1.80 (0.75H, m) 1.74-1.67 (1H, m), 1.66-1.55 (2H, m), 1.51-1.41 (1.5H, m), 1.36–1.28 (0.5H, m); ^{13}C NMR (101 MHz, $CDCl_3$, diastereomers) δ 174.7^{M} & 174.4^{m}, 134.8, 130.7, 128.5, 127.6, 77.5^{M} & 74.6^{m}, 72.5^{M} & 69.9^{m}, 43.5^{m} & 40.3^{M}, 37.5^{m} & 36.9^{M}, 31.2^{m} & 30.4^{M}, 27.4^{m} & 26.0^{M}, 21.4^{m} & 19.1^{M}; LRMS *m/z* (ESI): 215.1 (M^+).

6-Methyl-2-(5-methyl-3,4-dihydro-2*H*-pyrrol-2-yl)hept-5-en-2-ol (4u)

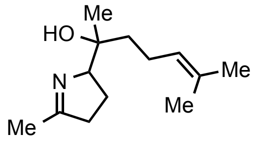

Following **GP12**, but irradiating the reaction for 3 days, **2z** (120 mg, 0.32 mmol) gave **4u** (20 mg, 29%) as an oil. R_f 0.60 [CH_2Cl_2–MeOH (90:10)]; FT-IR ν_{max} (film)/cm^{-1} 3338, 2967, 2923, 2883, 1649, 1521, 1453, 1375, 1319, 1151, 1093; ^{1}H NMR (400 MHz, $CDCl_3$, diastereomers) δ 5.18–5.10 (1H, m), 3.98–3.92 (1H, m), 2.59–2.47 (2H, m), 2.21–2.15 (1H, m), 2.08 (3H, s), 1.98–1.88 (1H, m), 1.79–1.74 (1H, m), 1.70 (3H, s), 1.65 (3H, s), 1.47–1.41 (1H, m), 1.33 (1.5H, s), 1.27 (1.5H, s), 1.27–1.22 (1H, m), 1.07 (1H, br s); ^{13}C NMR (101 MHz, $CDCl_3$, diastereomers) δ 176.5 & 176.4, 131.6 & 131.4, 124.75 & 124.7, 81.4 & 80.7, 74.1 & 74.0, 40.1 & 39.8, 39.8 & 37.5, 29.7, 25.8 & 24.5, 24.2 & 23.7, 22.3 & 21.8, 19.9 & 19.85, 17.7 & 17.7; HRMS (EI): Found MH^+ 209.1782 $C_{13}H_{23}NO$ requires 209.1780.

5.4 Mechanistic Studies

5.4.1 Proposed mechanism

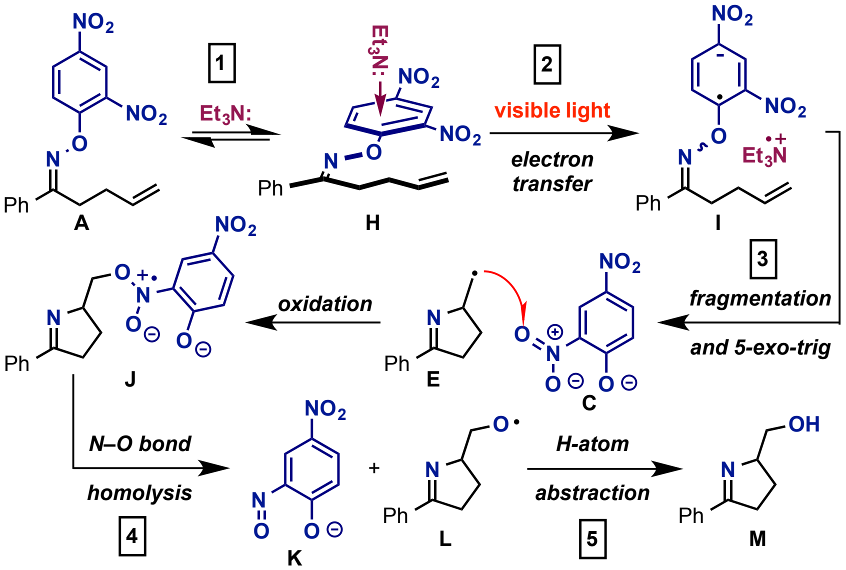

The photoredox cycle is supported by all the control experiments present in Section 5.2: entries 2 and 3 and the Light ON/OFF reaction (Section 5.4.2).

Further evidences:

STEP 1. The binding of **A** with Et_3N is supported by the UV/Vis studies, see Section 5.3.3.

STEP 2. The SET from the electron-donor-acceptor complex **H** is supported by the Glove Box reaction, see Section 5.4.4, and the reactions with monochromic LEDs, see Section 5.4.5. According to this mechanism, $Et_3N^{\bullet+}$ is formed and should decompose by several pathways like H-abstraction and deprotonation. When the reaction was run in CD_3CN, direct analysis of the mixture by ^1H NMR spectroscopy revealed peaks that are attributed to the decomposition of Et_3N (see Section 5.4.4).

STEP 3. **C** could never be isolated in the reactions.

STEP 4. Compound **K** was could be isolated in 15% yield. When the reaction was run in CD_3CN, direct analysis of the mixture by ^1H NMR spectroscopy revealed peaks that are attributed to the phenoxide **K**. This compound is extremely more polar than **C** (purification of **K** was achieved using column chromatography eluting with CH_2Cl_2:MeOH:NH_4OH 85:15:0.5) and we attribute the low mass recovery on the basis of problems in the purification as well as its possible decomposition.

STEP 5. The final O-atom abstraction might arise from Et_3N, $Et_3N^{\bullet+}$ or the CH_3CN. The decomposition product of Et_3N that can be observed in the glove-box reaction

(Section 5.4.4) seem to suggest Et_3N or $Et_3N^{\bullet+}$ as the H-atom source. In both cases very stable intermediates, an α-N-radical in the former case and an iminium ion in the latter case, would be generated.

The overall mechanism is supported by the Light ON/OFF reaction (see Section 5.4.2) and initial rate kinetic experiments (see Section 5.4.3).

5.4.2 Light ON/OFF reaction

A dry tube equipped with a stirring bar was charged with **2a** (30 mg, 0.088 mmol, 1.0 equiv.), Et_3N (23 μL, 0.176 mmol, 2.0 equiv.) and CD_3CN (0.27 mL). The mixture for stirred for 15 min and then the 30 W light bulb was switched on and stirred under visible light irradiation for 6h at which point a reaction aliquot (10 μL) was taken, and diluted with $CDCl_3$ and analysed by ^1H NMR spectroscopy. The light bulb was switched off and the mixture was stirred in the dark for 12h at which point a reaction aliquot (10 μL) was taken, and diluted with $CDCl_3$ and analysed by ^1H NMR spectroscopy. The light bulb was switched on and the mixture stirred under visible light irradiation for 6h at which point a reaction aliquot (10 μL) was taken, and diluted with $CDCl_3$ and analysed by ^1H NMR spectroscopy.

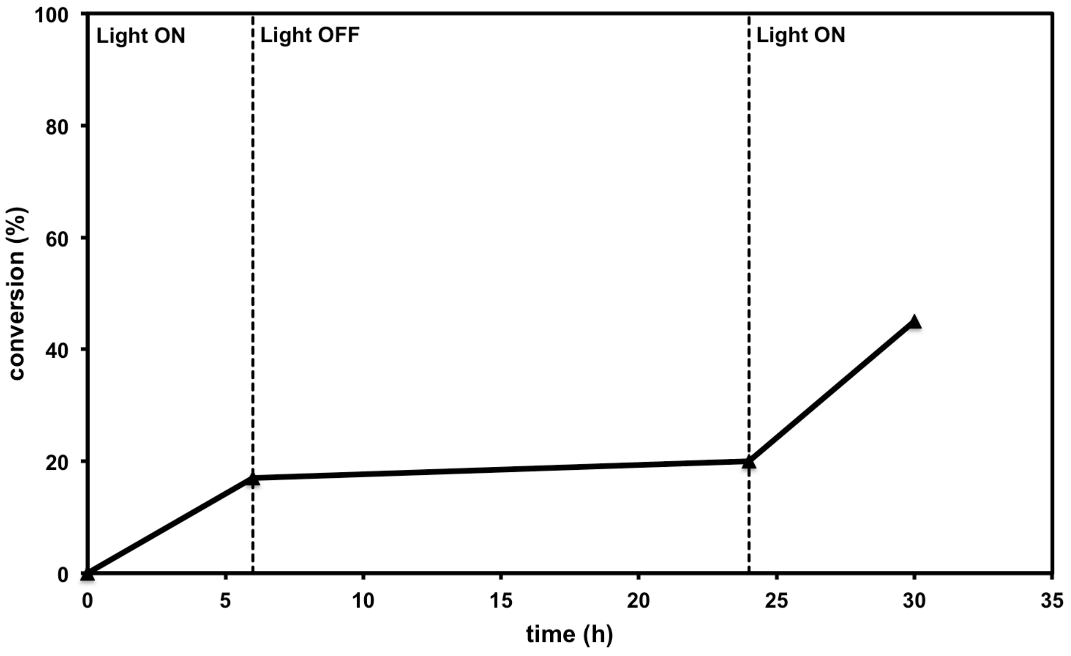

5.4.3 Kinetic studies

Six kinetic experiments were performed simultaneously. In each 5 mL Schlenk tube equipped with a stir bar, the desired amounts of **2a**, Et_3N and Et_4Si (internal standard, 0.25 equiv.) were added and brought to a final volume of 0.6 mL by addition of CD_3CN. The six reactions were allowed to stir for 10 minutes then the 30 W light-bulb was switched on. Aliquots (10-15 μL) were syringed out as the reactions progressed, placed in a dry NMR tube and diluted with $CDCl_3$. Reactions were analysed by ^1H NMR spectroscopy out to 5-15% conversion to determine the initial rates. In order to obtain better plots, the disappearance of **2a** was plotted.

[**2a**] (1.0 equiv.)	[Et_3N] (M)	Et_3N (equiv.)	$k$obs (s mol^{-1})
0,249193785	0,149516271	0.6	-0.000150592
0,249193785	0,199355028	0.8	-0.000184804
0,249193785	0,249193785	1.0	-0.000229631
0,249193785	0,996775139	4.0	-0.000239578
0,249193785	1,993550279	8.0	-0.000237335
0,49838757	0,996775139	2.0	-0.000159242

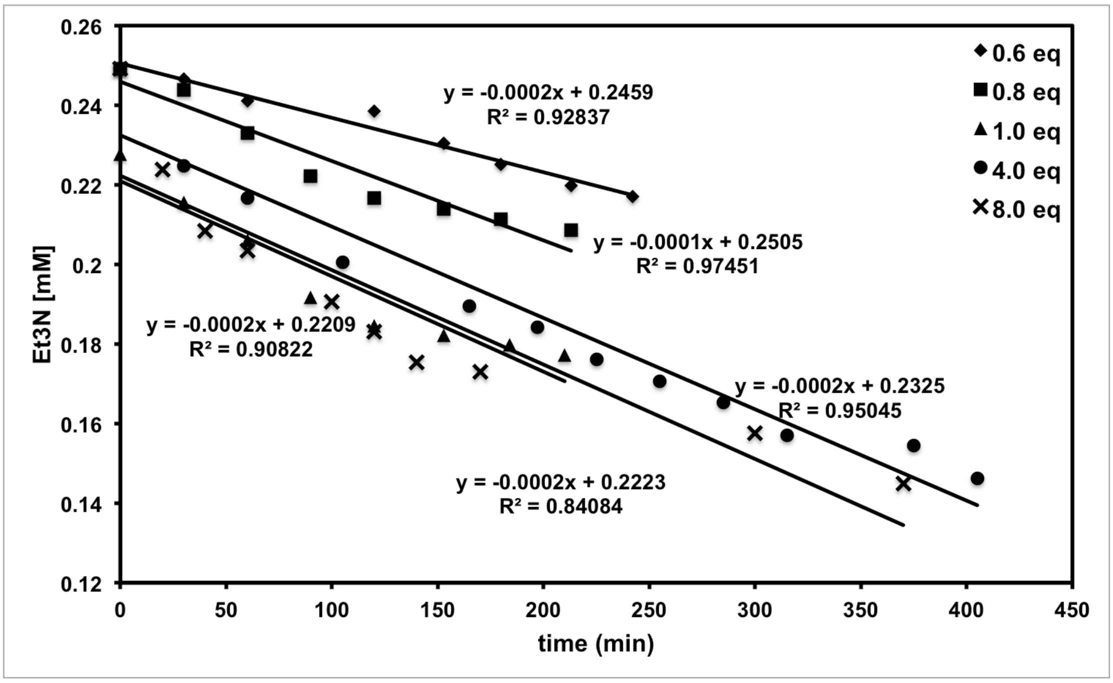

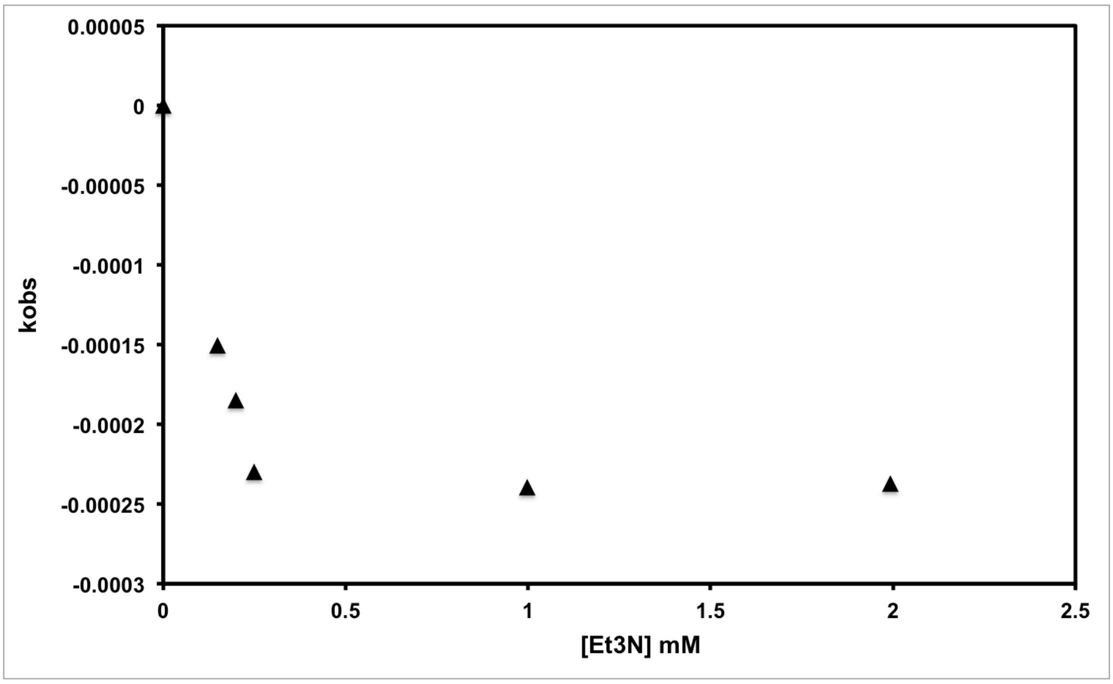

0.00005
0
-0.00005
-0.0001
-0.00015
-0.0002
-0.00025
-0.0003
kobs
0
0.5
1
1.5
2
2.5
[Et3N] mM

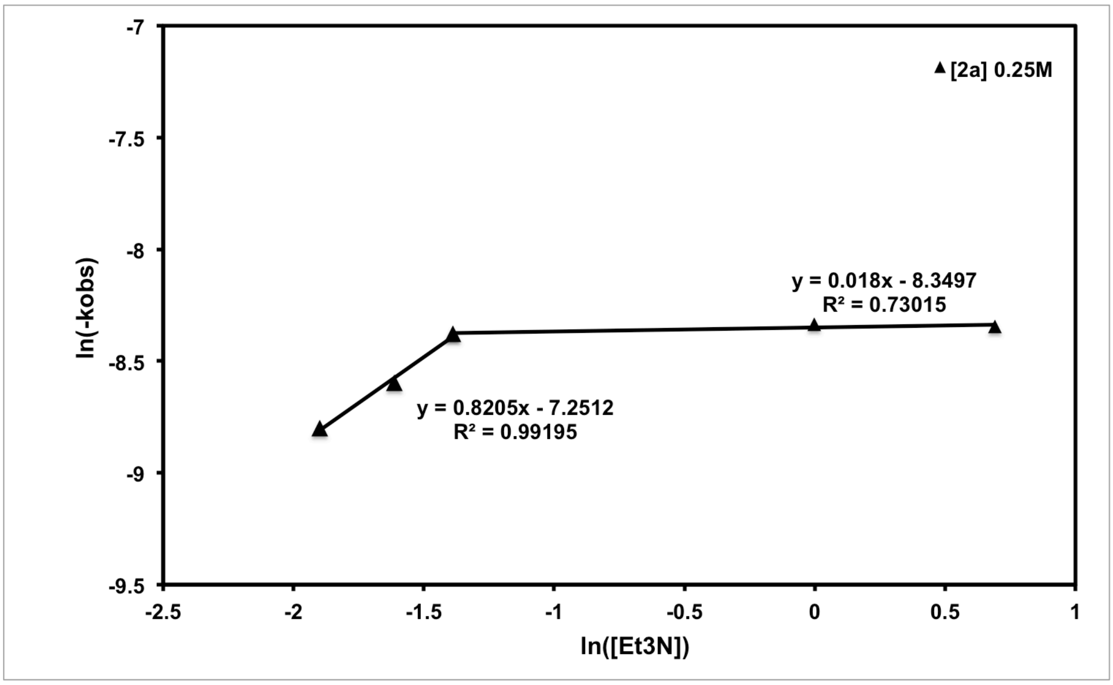

-7
-7.5
-8
-8.5
-9
-9.5
ln(-kobs)
[2a] 0.25M
y = 0.018x - 8.3497
R² = 0.73015
y = 0.8205x - 7.2512
R² = 0.99195
-2.5
-2
-1.5
-1
-0.5
0
0.5
1
ln([Et3N])

5.4.4 Glove-box reaction

A dry Schlenck tube equipped with a stirring bar was charged with **2a** (30 mg, 0.088 mmol, 1.0 equiv.), evacuated and refilled with nitrogen (x 3). A second dry Schlenck tube was charged with freshly distilled Et_3N (~2 mL) and degassed by using the freeze-pump-thaw protocol (x 3). A third dry Schlenck tube was charged with CD_3CN (~10 mL) and degassed by using the freeze-pump-thaw protocol (x 3). The three Schlenck tubes were put in the Glove Box and the oxime containing tube was charged with Et_3N (23 μL, 0.176 mmol, 2.0 equiv.) and CD_3CN (0.3 mL). The mixture was stirred for 15 min, sealed, removed from the Glove Box and stirred under visible light irradiation overnight. 0.1 mL of reaction mixture were placed in a dry Young's NMR tube and analysed by ^{1}H NMR spectroscopy.

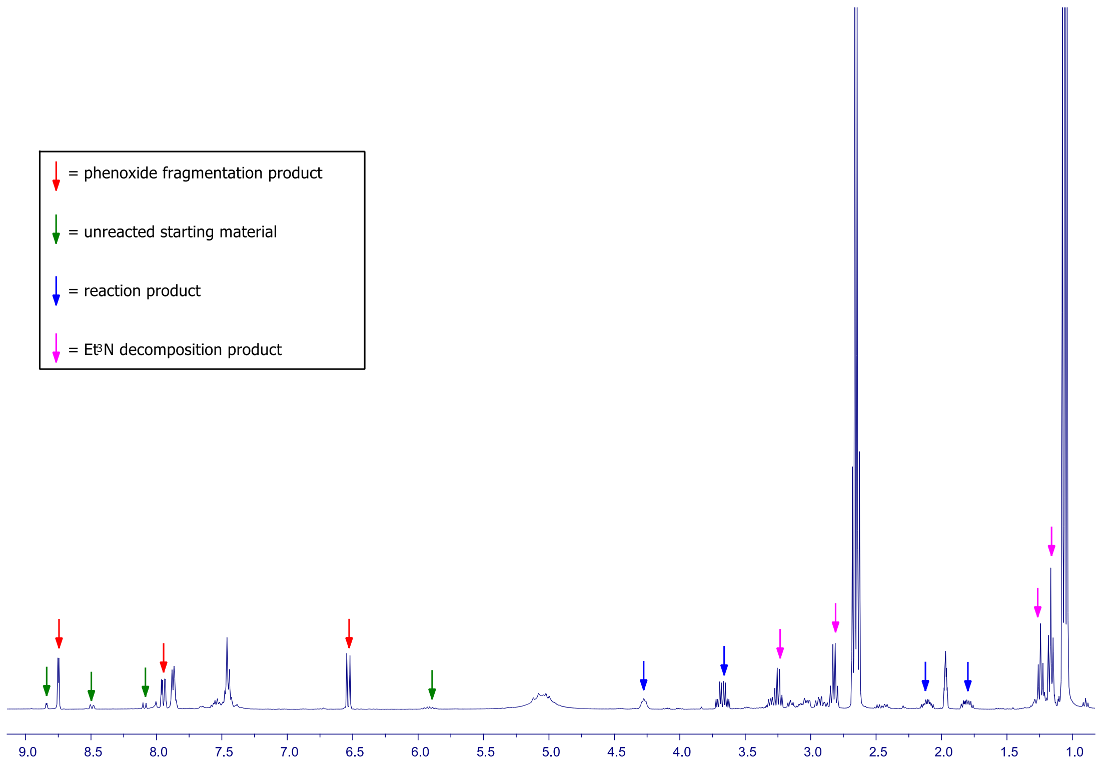

5.4.5 Reaction with monochromatic LEDs

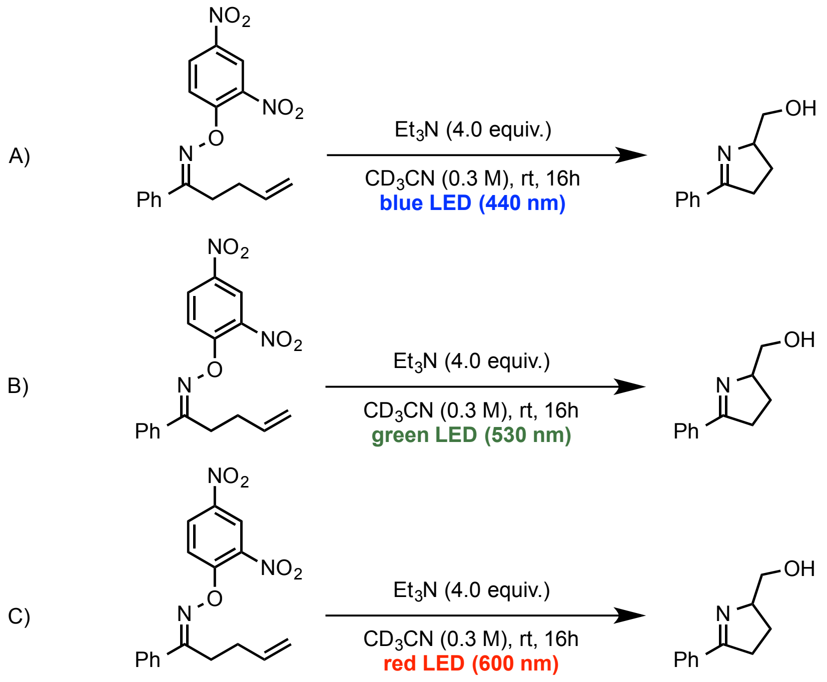

Three dry tubes equipped with a stirring bar were charged with **2a** (20 mg, 0.059 mmol, 1.0 equiv.), Et_3N (31 μL, 0.234 mmol, 4.0 equiv.) and CD_3CN (0.18 mL). The mixtures were stirred at room temperature overnight in front of a blue (A), a green (B) and a red LED (C). The mixtures were diluted with $CDCl_3$ (0.5 mL) and analysed by ^1H NMR spectroscopy to determine the reaction conversion.

Entry	Reaction	Conversion (%)
1	**A**	96
2	**B**	31
3	**C**	0

These results support the overall mechanism as blue light has the optimum wavelength to promote the excitation of the EDAC **H** and therefore facilitate the required SET process. Using red LEDs the reaction failed to provide even traces of product because at this wavelength there is no absorption.

6 Computational Studies

6.1 Determination of LUMO energies for oximes 1a–g

The structure of oximes **1a–e** and **g** were optimized using Gaussian 09[41] and the energy of the LUMO obtained using Avogadro Software.

Method: opt freq b3lyp 6-31+G(d,p) scrf=(solvent=acetonitrile)

Oxime	ΔE LUMO (eV)	$E_{1/2}^{red}$ (V)	Oxime	ΔE LUMO (eV)	$E_{1/2}^{red}$ (V)
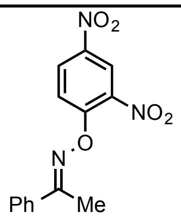<br>**1a**	0.0	–0.55	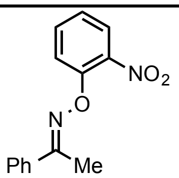<br>**1d**	–0.3	–0.93
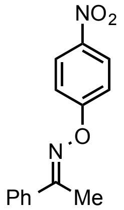<br>**1b**	–0.2	–0.65	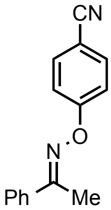<br>**1e**	–1.3	–1.7
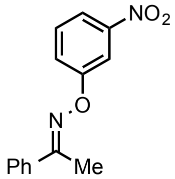<br>**1c**	–0.1	–0.67	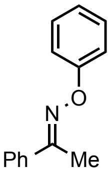<br>**1g**	–1.6	–1.87

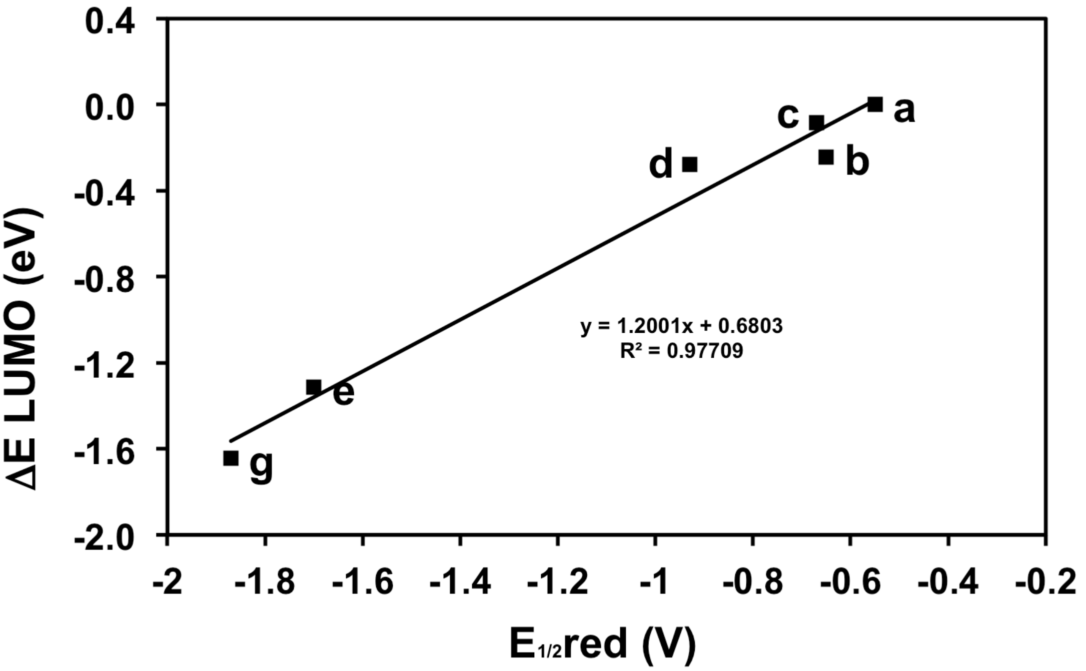
0.4
0.0
-0.4
-0.8
-1.2
-1.6
-2.0
ΔE LUMO (eV)
a
b
c
d
e
g
y = 1.2001x + 0.6803
R² = 0.97709
-2
-1.8
-1.6
-1.4
-1.2
-1
-0.8
-0.6
-0.4
-0.2
E1/2red (V)

6.2 Energy minimization for the 1a•Me_3N EDAC complex

6.2.1 Calculation details

Calculations were performed with the GAUSSIAN 09 suite of programs.[41] Density Functional Theory (DFT) was applied by the means of the B3LYP hybrid functional[42] corrected for dispersion as proposed by Grimme. Geometry optimizations were performed using the D2 version of Grimme's correction[43] (geometry optimizations using the newest D3 correction kept converging on structures with small imaginary frequencies) together with the 6-31+G(d) basis set. Electronic energies were then refined using the D3 correction with Becke-Johnson damping and the larger 6-311++G(d,p) basis set. This did not have a significant effect on the outcome of the calculations. The stationary points were characterized by full vibration frequencies calculations. Gibbs free energies were obtained from the standard enthalpies and entropies computed at 298 K in Gaussian based on the rigid rotor harmonic oscillator approach to statistical mechanics and corrected to take into account a standard state corresponding to species in solution at a concentration of 1 M: $G^{1M} = G^{1atm} + RTln(C^{1M}/C^{1atm})$ where G^{1atm} is the Gibbs free energy output by Gaussian, C^{1M} is the assumed concentration in solution and C^{1atm} is the gas phase concentration, *i.e.* 0.0409 mol.L^{-1} for an ideal gas at 298 K and 1 atm). A concentration of 2 M was assumed for the amine in order to reproduce the fact that experiments were done using 2 equivalent of NEt_3. Solvent effects (acetonitrile) were included by means of SMD single point calculations[44] on the gas-phase optimized structures. Gibbs free energies in solution were estimated as: $G^{ACN} = G^{1M} + E^{ACN} - E^{gas\,phase}$. The long-range corrected functional CAM-B3LYP[45] was used for all time-dependent DFT calculations. These calculations were performed on gas-phase optimized structures with the 6-311++G(d,p) basis set and the SMD solvation scheme.

6.2.2 Results.

6.2.2.1 Electron donor-acceptor complexes

Slightly truncated models of the experimental aryloximes **2a**, **2b** and **2c** (**1a**, **1b**, **1c**) were used in the calculations along with Me_3N.

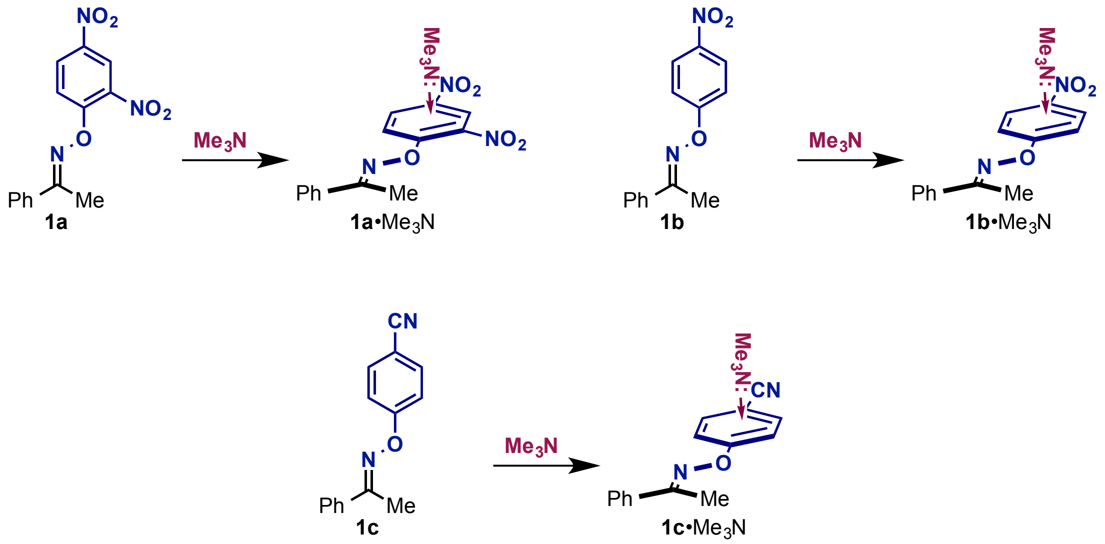

Complexation of the amine is predicted to be very weak, with computed free energies ranging between 3.1 for **1a** and 5.6 kcal.mol^{-1} for **1c**. We note that this is slightly higher than what was predicted based on experimental observations (the experimental association constant found for **2a**, *i.e.* $K \approx 22$ M^{-1}, corresponds to a theoretical free energy of –1.8 kcal.mol^{-1}) though lies within the margin of error anticipated from our model chemistry. The trend, however, should be more reliable owing to the similarity of the substrates and is indeed in good agreement with experiments.

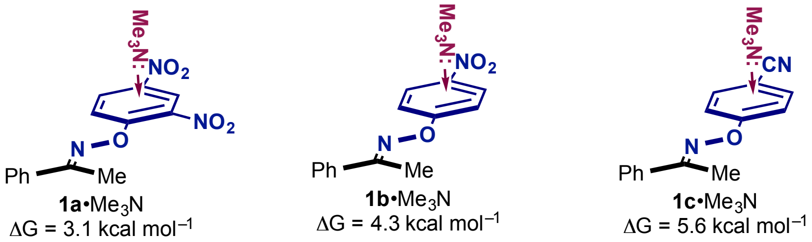

As expected, the interaction between the amine and the aryloxime is due to donation from the lone pair of the nitrogen into the π^* orbital of the aryloxime. Interestingly, the latter is found to be mainly localized on one of the electron-withdrawing groups (NO_2 or CN), with very little or no delocalization onto the aromatic ring. This results in complexes in which the amine lies above one of the aryl substituents rather than on top of the aromatic.

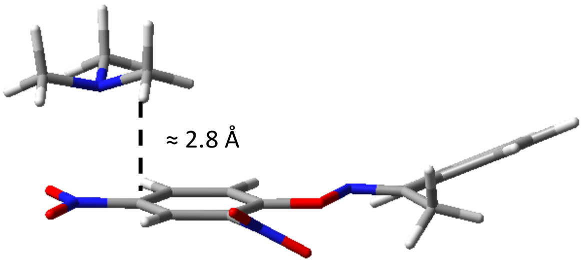

6.2.2.2 Simulated UV-Vis spectra

TD-DFT (CAM-B3LYP/SMD/6-311++G(d,p)) was used to simulate the UV-Vis spectra of **1a** and **1a**•Me_3N. The bathochromic shift observed experimentally is well reproduced and is confirmed to be due to an $n \rightarrow \pi^*_{NO2}$ transition (HOMO → LUMO+1).

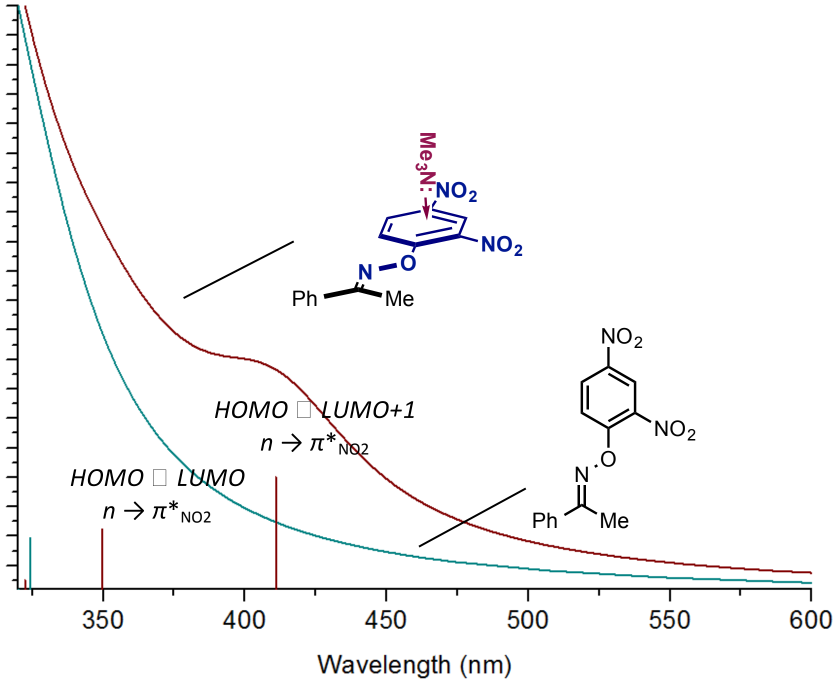

Selected Kohn-Sham orbitals of **1a**•Me_3N		
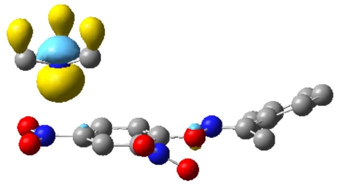	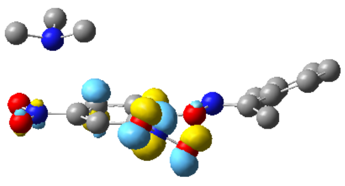	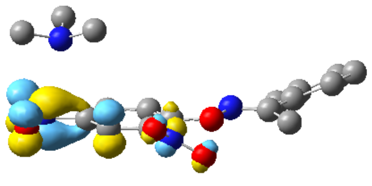
HOMO	LUMO	LUMO+1

6.2.2.3 Absolute energies and Cartesian coordinates.

Compound	$E^{gas\ phase}$ (B3LYP-D3BJ/6-311++G(d,p))	G^{1atm} (B3LYP-D2/6-31+G(d))	E^{ACN} (B3LYP-D2/6-31+G(d))
**Me_3N**	-174.5414609	-174.397415	-174.496448318
**1a**	-1080.6031062	-1080.107459	-1080.32041460
**1a**•Me_3N	-1255.1577543	-1254.501047	-1254.82596253
**1b**	-876.0460254	-875.606799	-875.819540349
**1b**•Me_3N	-1050.5986073	-1049.998523	-1050.32290703
**1c**	-763.7468548	-763.343096	-763.554171907
**1c**•Me_3N	-938.2978575	-937.732556	-938.057663119

NMe_3

N	0.100616	-0.491957	-0.069169
C	-0.085368	-0.041236	1.303094
C	1.451856	-0.204588	-0.530062
C	-0.887258	0.113378	-0.951507
H	-1.896266	-0.146732	-0.609215
H	-0.802118	1.221273	-0.978819
H	-0.754184	-0.268078	-1.971162
H	1.582628	-0.585764	-1.550124
H	1.670936	0.885107	-0.533280
H	2.178405	-0.700600	0.124932
H	-1.095174	-0.301163	1.643164
H	0.642666	-0.537445	1.956287
H	0.045724	1.057805	1.404870

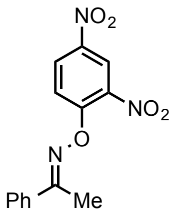

C	2.112349	0.951435	0.052223
C	0.935326	0.161436	0.034875
C	1.068322	-1.238277	-0.045724
C	2.325745	-1.824017	-0.096284
C	3.469055	-1.017197	-0.082671
C	3.373372	0.368371	-0.020863
O	-0.246384	0.805033	0.059860
N	-1.384843	-0.047619	0.000147
C	-2.458937	0.664915	0.071747
C	-2.458230	2.170781	0.172861
N	4.796387	-1.639452	-0.137688
O	4.847707	-2.870160	-0.202450
N	2.064154	2.419393	0.149286
O	2.979981	3.045878	-0.386688
C	-3.731724	-0.095545	0.032460
C	-3.790960	-1.433515	0.467858
C	-4.989646	-2.143143	0.408520
C	-6.147617	-1.531903	-0.089396
C	-6.098761	-0.203845	-0.523823
C	-4.900960	0.512499	-0.457058
O	5.780943	-0.898699	-0.115980
O	1.135360	2.932259	0.773109
H	2.432368	-2.901184	-0.151310
H	0.174355	-1.845701	-0.073770
H	4.260134	0.989733	-0.024487
H	-2.196006	2.614431	-0.797171
H	-3.436846	2.542892	0.483052
H	-1.696081	2.501300	0.885280
H	-4.874106	1.540755	-0.804824

H	-6.991920	0.277108	-0.913666
H	-7.081593	-2.086082	-0.132701
H	-5.023808	-3.171831	0.757679
H	-2.894481	-1.900964	0.862207

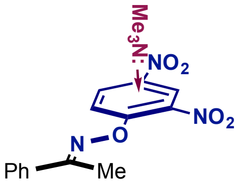

C	1.149012	1.465329	-0.381923
C	2.445883	1.042130	-0.657189
C	2.653863	-0.271179	-1.053895
C	1.589569	-1.168392	-1.170094
C	0.297183	-0.748313	-0.881126
C	0.049836	0.578047	-0.483050
N	4.018476	-0.721990	-1.339825
O	4.177744	-1.904065	-1.655248
O	-1.170014	1.053442	-0.153436
N	-2.211458	0.088478	-0.208387
C	-3.337651	0.653090	0.072812
C	-3.473726	2.114512	0.424575
O	4.926262	0.108205	-1.274933
C	-4.522935	-0.238485	0.047359
N	3.864903	-1.261122	1.386494
C	3.308566	-2.599531	1.471609
C	3.190958	-0.328069	2.272682
C	5.307793	-1.255324	1.568589
H	1.784730	-2.190152	-1.473084
H	-0.534626	-1.435811	-0.948888
H	3.275989	1.727811	-0.548555
H	-2.860988	2.723052	-0.247673
H	-4.515447	2.436826	0.365179
H	-3.098803	2.295591	1.441057
H	2.232863	-2.561830	1.256798
H	3.793025	-3.241035	0.726107
H	3.447144	-3.051958	2.475249
H	3.584307	0.683004	2.112700
H	2.117193	-0.321214	2.047444
H	3.322388	-0.590527	3.342528
H	5.769690	-1.902993	0.814272
H	5.689117	-0.237637	1.428207
H	5.604625	-1.611911	2.576284
N	0.983154	2.865717	0.033243
C	-5.646563	0.059312	0.838009
C	-6.757261	-0.788629	0.841726
C	-6.765962	-1.937736	0.045364
C	-5.654443	-2.237826	-0.752773
C	-4.541465	-1.398011	-0.751666
H	-5.649044	0.944470	1.466894
H	-7.614331	-0.549334	1.465663
H	-7.633579	-2.592398	0.041277
H	-5.659212	-3.123890	-1.382258
H	-3.682774	-1.622590	-1.376099
O	-0.063501	3.443779	-0.259529
O	1.922974	3.384938	0.640648

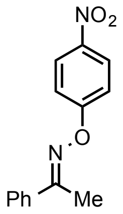

C	3.167272	-0.745034	0.029751
C	2.279491	-0.963019	1.096876
C	0.893753	-1.016194	0.882277
C	0.402663	-0.851679	-0.410856
C	1.290413	-0.636226	-1.468423
C	2.673550	-0.580487	-1.257568
O	2.881151	-1.102723	2.316565
N	1.983544	-1.390910	3.368951
C	2.609653	-1.449517	4.495013
C	4.092589	-1.202284	4.630935
N	0.764894	-0.464368	-2.823550

O	1.572776	-0.272486	-3.737973
H	4.234165	-0.708997	0.227068
C	1.774989	-1.778916	5.676523
C	0.599780	-2.542582	5.537809
C	-0.191616	-2.831543	6.649029
C	0.173834	-2.362703	7.917438
C	1.338922	-1.604159	8.065730
C	2.137324	-1.319142	6.954752
O	-0.458430	-0.520066	-2.983269
H	-0.662767	-0.886971	-0.606329
H	0.221708	-1.179962	1.713906
H	3.339411	-0.412149	-2.095679
H	4.331108	-0.159802	4.383642
H	4.444445	-1.426693	5.639681
H	4.636355	-1.830396	3.916081
H	3.032954	-0.718697	7.083610
H	1.628444	-1.232805	9.045308
H	-0.442631	-2.591459	8.782918
H	-1.090928	-3.429865	6.527684
H	0.324769	-2.911621	4.554972

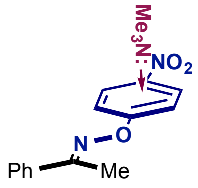

C	2.983996	-0.578128	-0.671267
C	1.074872	-0.960800	1.360568
N	1.650908	2.576705	-1.127118
C	0.671262	-0.564442	0.086942
C	1.622912	-0.373078	-0.915251
N	1.190134	0.057741	-2.243984
O	-0.018293	0.211739	-2.441225
C	3.392332	-0.974706	0.596335
C	2.439629	-1.163344	1.610125
O	2.961993	-1.543266	2.819434
N	1.989824	-1.777936	3.813674
C	2.549358	-2.090279	4.932947
C	1.623189	-2.387545	6.053350
C	2.020561	-2.167308	7.383799
C	1.141474	-2.420677	8.440333
C	-0.143225	-2.908846	8.183609
C	-0.546026	-3.138323	6.861688
C	0.327131	-2.880009	5.805534
O	2.054259	0.223494	-3.111091
C	4.046082	-2.154379	5.119155
H	4.442122	-1.141958	0.817392
H	-0.375638	-0.391293	-0.132316
H	0.350846	-1.106457	2.151043
H	3.701678	-0.419336	-1.466857
H	4.488082	-1.154821	5.016275
H	4.310478	-2.570044	6.093470
H	4.487151	-2.778432	4.333553
H	3.011047	-1.777087	7.598246
H	1.461916	-2.235891	9.462373
H	-0.824068	-3.113802	9.005748
H	-1.539932	-3.526785	6.654773
H	0.021732	-3.065011	4.780695
C	1.169797	2.874225	0.209342
C	3.080143	2.793818	-1.255760
C	0.898532	3.273716	-2.157140
H	1.017279	4.375642	-2.091719
H	1.241532	2.938433	-3.142828
H	-0.164893	3.026738	-2.061757
H	3.360353	3.859014	-1.114785
H	3.609043	2.194282	-0.504332
H	3.405775	2.473085	-2.252378
H	1.305831	3.942229	0.481809
H	0.101420	2.634332	0.275060
H	1.708912	2.256536	0.938187

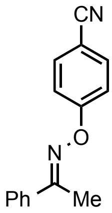

C	2.226976	-1.636380	0.705649
C	0.865618	-1.277085	0.740511
C	0.374652	-0.389730	-0.233186
C	1.224574	0.135822	-1.210230
C	2.574636	-0.226673	-1.235739
C	3.071761	-1.115790	-0.274154
C	-0.033354	-1.810618	1.793459
C	-1.514801	-1.967713	1.549617
N	0.535368	-2.121843	2.908355
O	-0.406986	-2.644413	3.818838
C	0.116825	-2.941171	5.050572
C	1.438520	-2.679868	5.436667
C	1.846960	-3.023236	6.724264
C	0.951360	-3.621634	7.628511
C	-0.374446	-3.874864	7.224048
C	-0.788883	-3.536813	5.941714
C	1.382162	-3.969742	8.949295
N	1.731281	-4.253418	10.023672
H	2.868265	-2.825154	7.035477
H	2.125233	-2.215975	4.741107
H	-1.070126	-4.336549	7.918008
H	-2.083734	-1.289523	2.198691
H	-1.771444	-1.773533	0.506388
H	-1.823006	-2.987136	1.808625
H	-1.806574	-3.727275	5.614688
H	-0.668864	-0.089401	-0.218959
H	0.830389	0.828032	-1.949719
H	3.233824	0.175597	-2.000663
H	4.118097	-1.409692	-0.293376
H	2.606345	-2.332502	1.446796

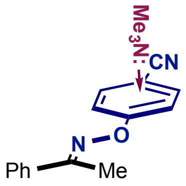

C	-4.074199	0.086721	-1.295644
C	-2.954148	0.898016	-1.562756
C	-1.664496	0.427972	-1.340429
C	-1.489212	-0.870727	-0.845303
C	-2.592424	-1.694036	-0.581693
C	-3.880172	-1.218254	-0.805584
O	-0.276236	-1.446495	-0.582767
N	0.805693	-0.559550	-0.691059
C	1.899094	-1.075458	-0.245825
C	2.035013	-2.472253	0.312003
C	-5.397446	0.586020	-1.518120
N	-6.474189	0.993649	-1.695881
C	3.095086	-0.197416	-0.312861
N	-0.746362	0.562190	1.735443
C	-2.086922	1.091125	1.931739
C	0.275233	1.523563	2.115101
C	-0.568985	-0.701010	2.428973
H	-3.103367	1.905171	-1.940560
H	-0.802255	1.054304	-1.523027
H	-4.738841	-1.849862	-0.599196
H	2.438610	-3.146904	-0.456108
H	2.731173	-2.475115	1.157337
H	1.068559	-2.863428	0.633135
H	1.267077	1.113357	1.892928
H	0.143473	2.446460	1.536408
H	0.233161	1.781829	3.194863
H	-1.313548	-1.422108	2.070728
H	0.430664	-1.097736	2.216689
H	-0.679522	-0.598218	3.529389
H	-2.202377	2.019053	1.359723
H	-2.822850	0.367276	1.563431
H	-2.302385	1.307007	2.999484

H	-2.426232	-2.696599	-0.199175
C	4.385047	-0.750978	-0.238597
C	5.517474	0.066179	-0.307175
C	5.377570	1.450022	-0.443007
C	4.095770	2.012518	-0.511141
C	2.965694	1.199342	-0.446087
H	4.509923	-1.824949	-0.138463
H	6.506872	-0.380851	-0.254604
H	6.257347	2.086569	-0.490865
H	3.978395	3.088932	-0.607928
H	1.972344	1.633056	-0.486514

7 NMR Spectra

S2: ^{1}H NMR (400 MHz, $CDCl_3$)

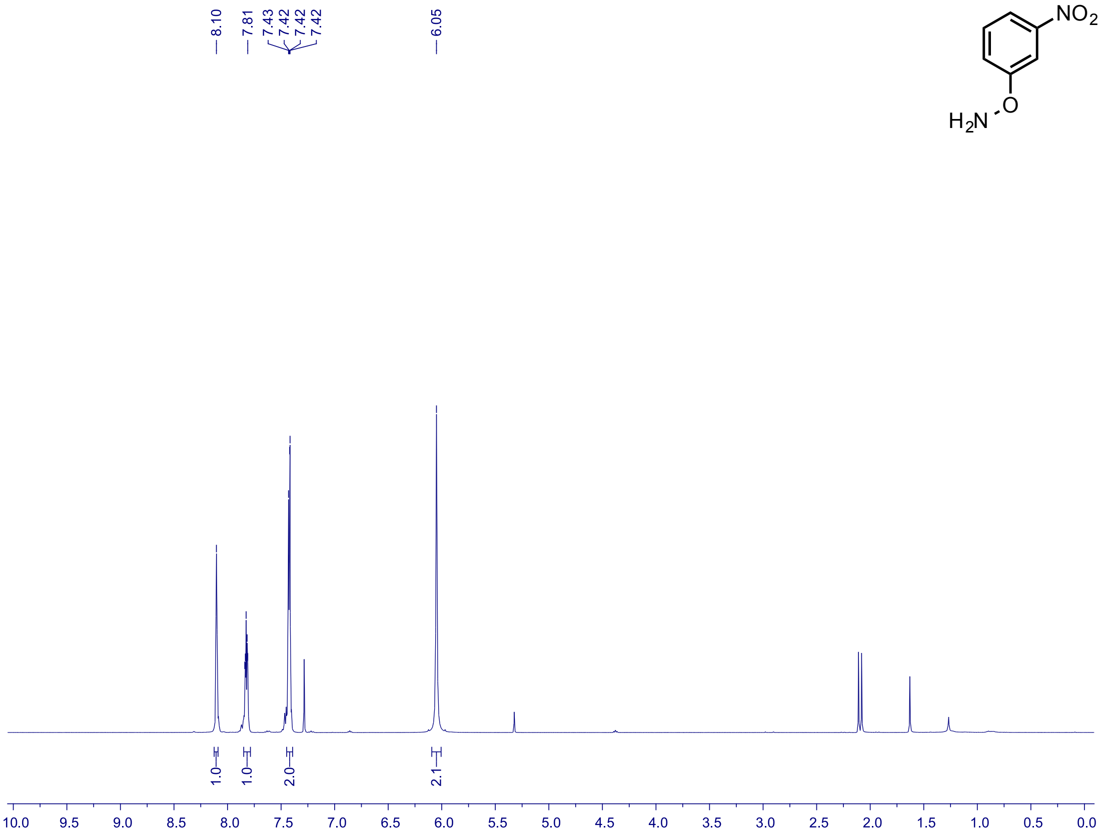

S2: ^{13}C NMR (101 MHz, $CDCl_3$)

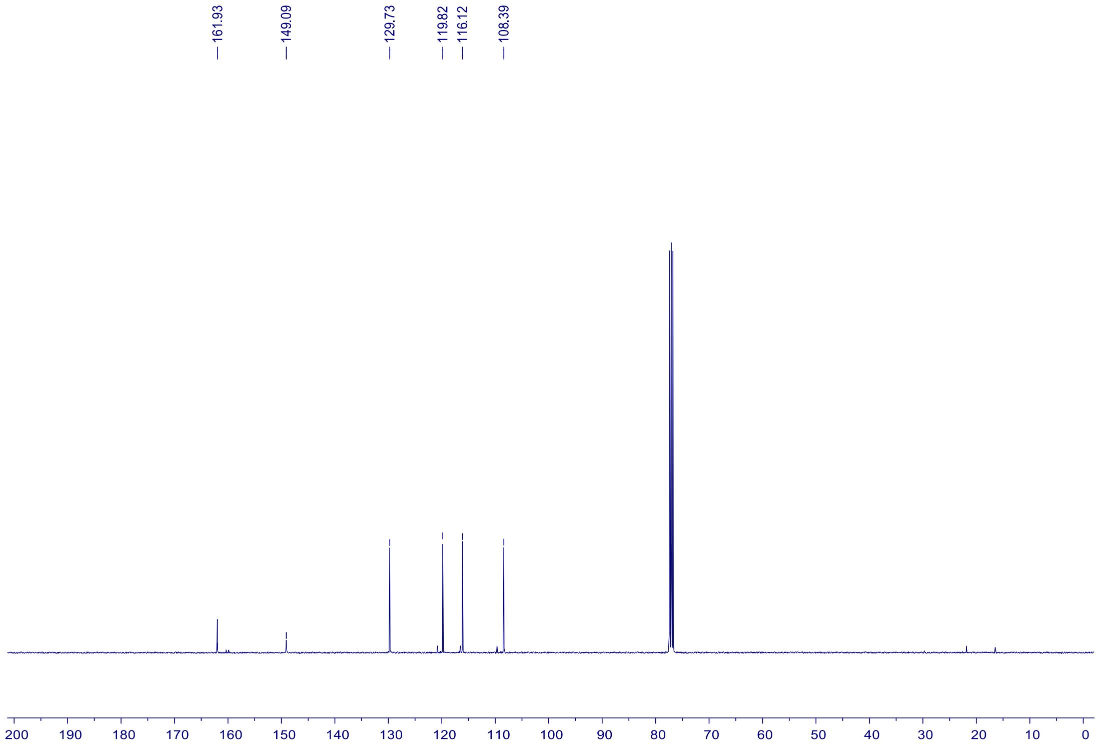

S3: ^{1}H NMR (400 MHz, $CDCl_3$)

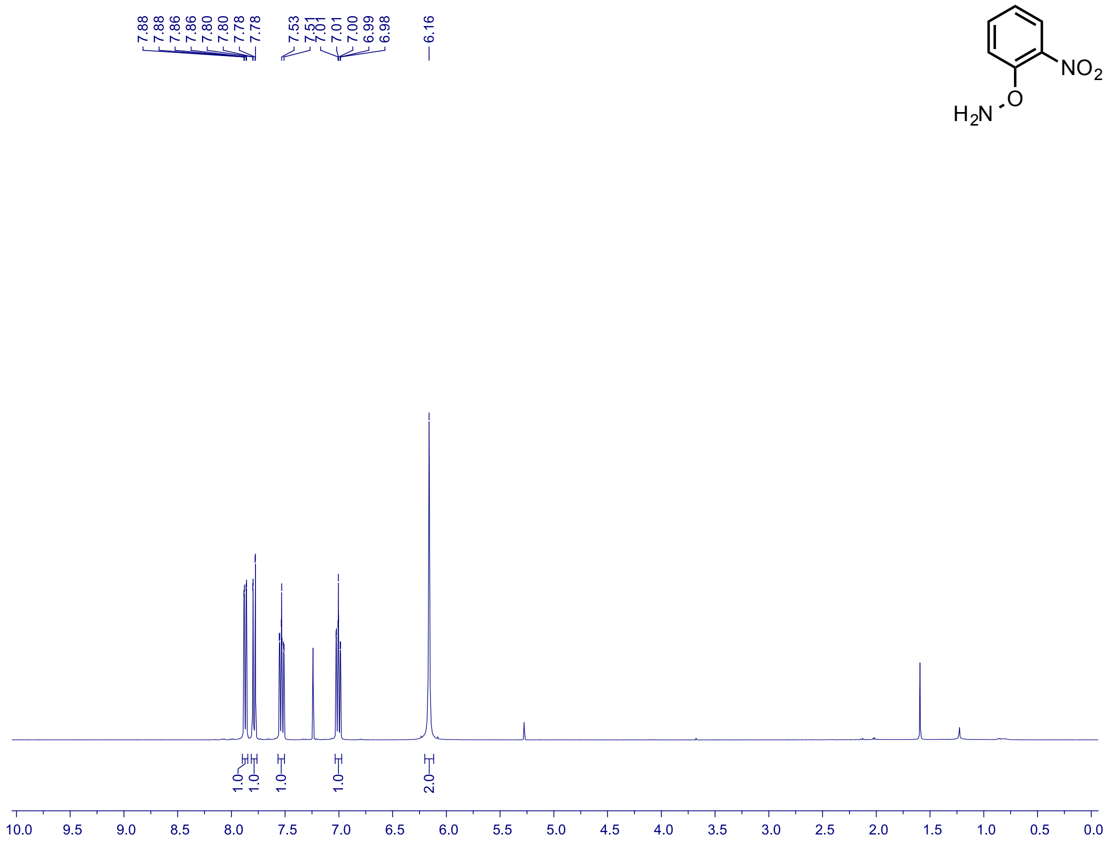

S3: ^{13}C NMR (101 MHz, $CDCl_3$)

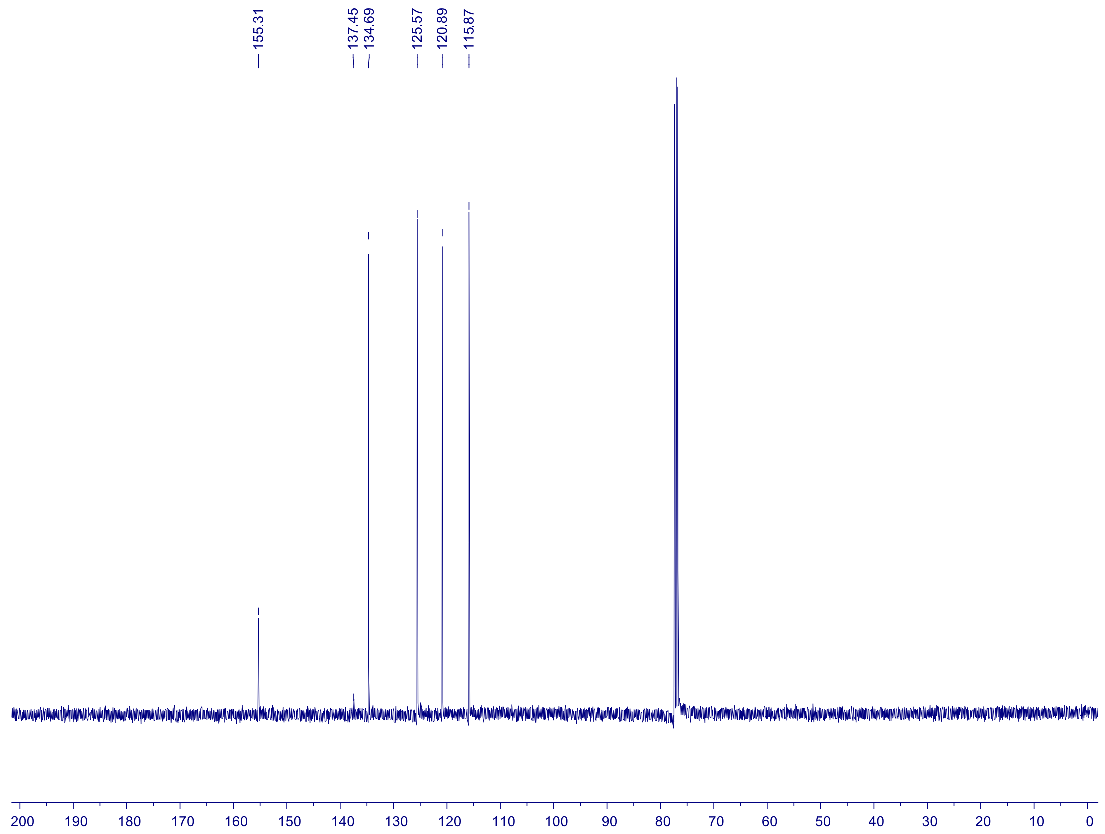

1a: ^{1}H NMR (400 MHz, $CDCl_3$)

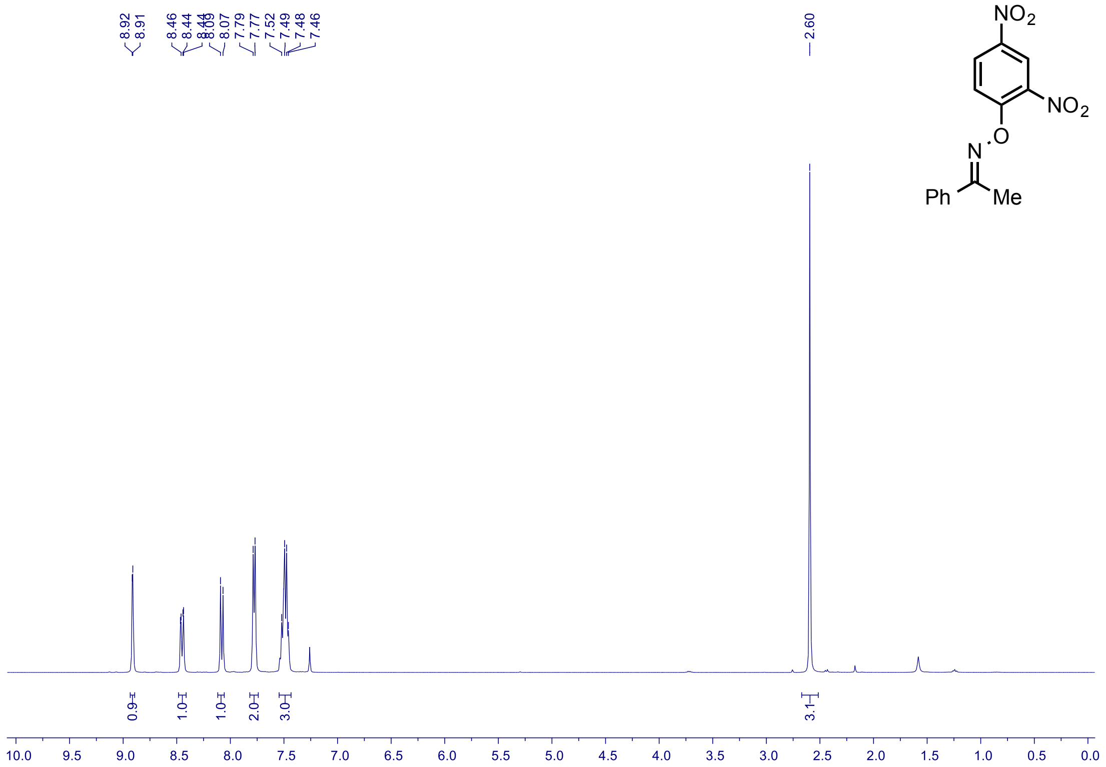

1a: ^{13}C NMR (101 MHz, $CDCl_3$)

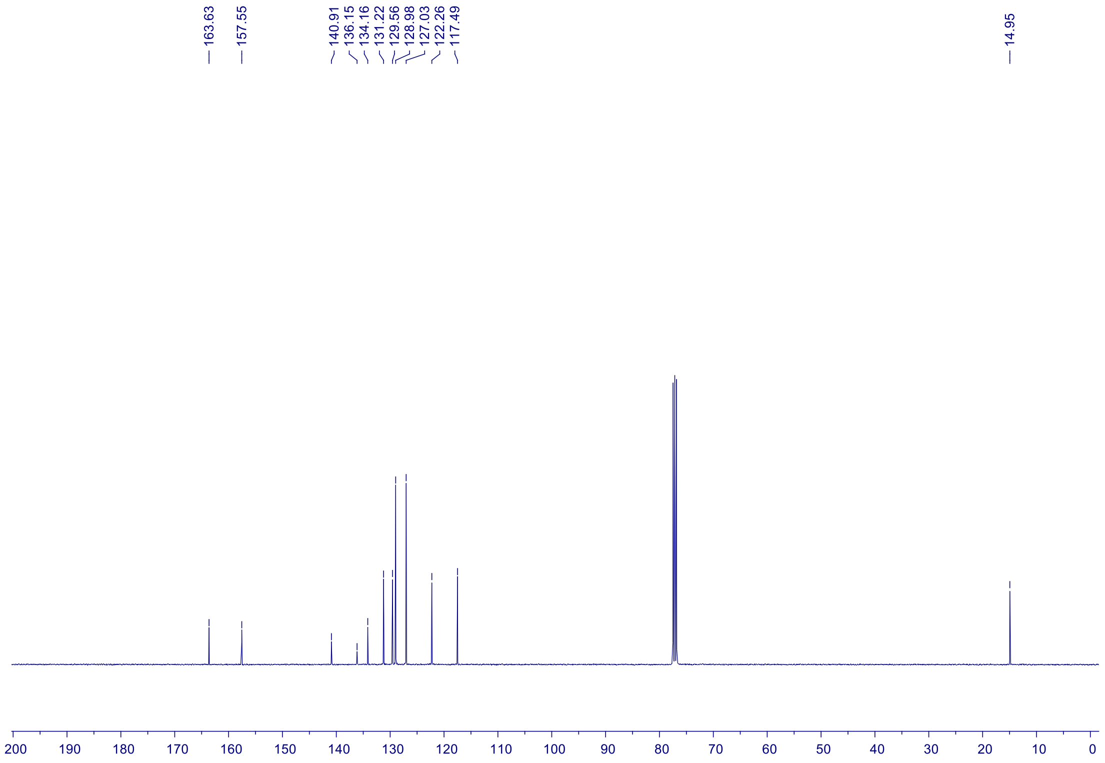

1e: ^{1}H NMR (400 MHz, $CDCl_3$)

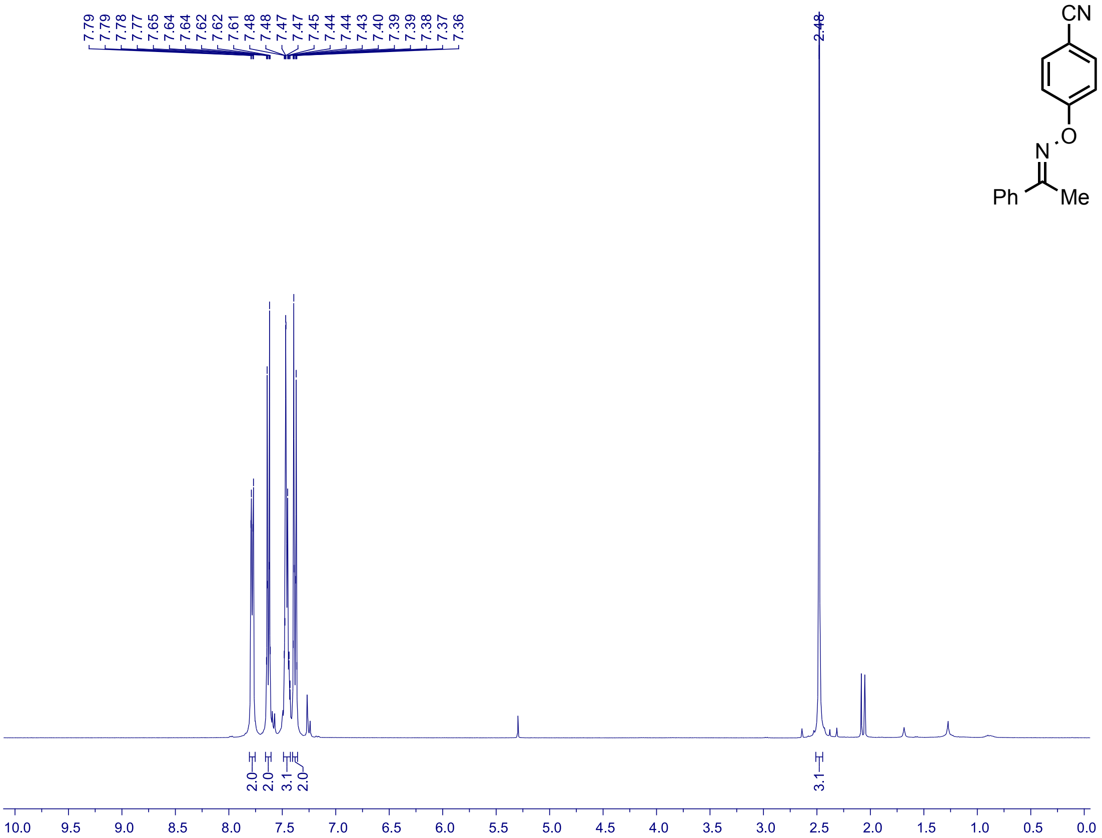

1e: ^{13}C NMR (101 MHz, $CDCl_3$)

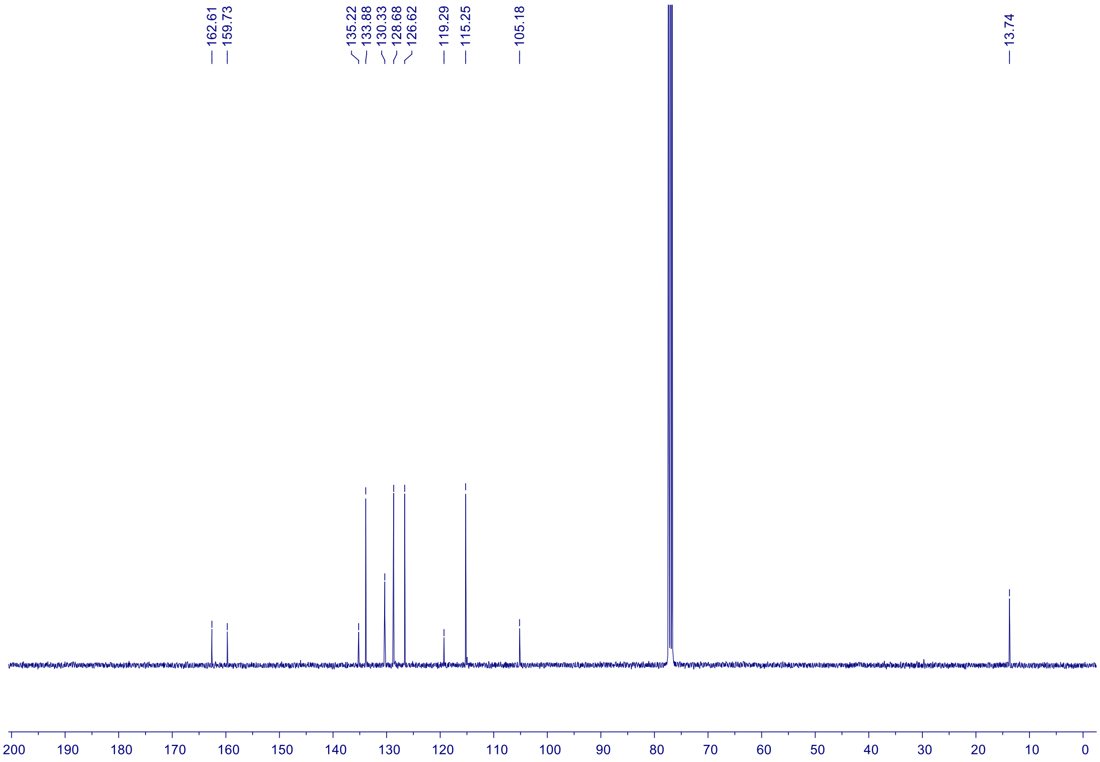

1f: ^{1}H NMR (400 MHz, $CDCl_3$)

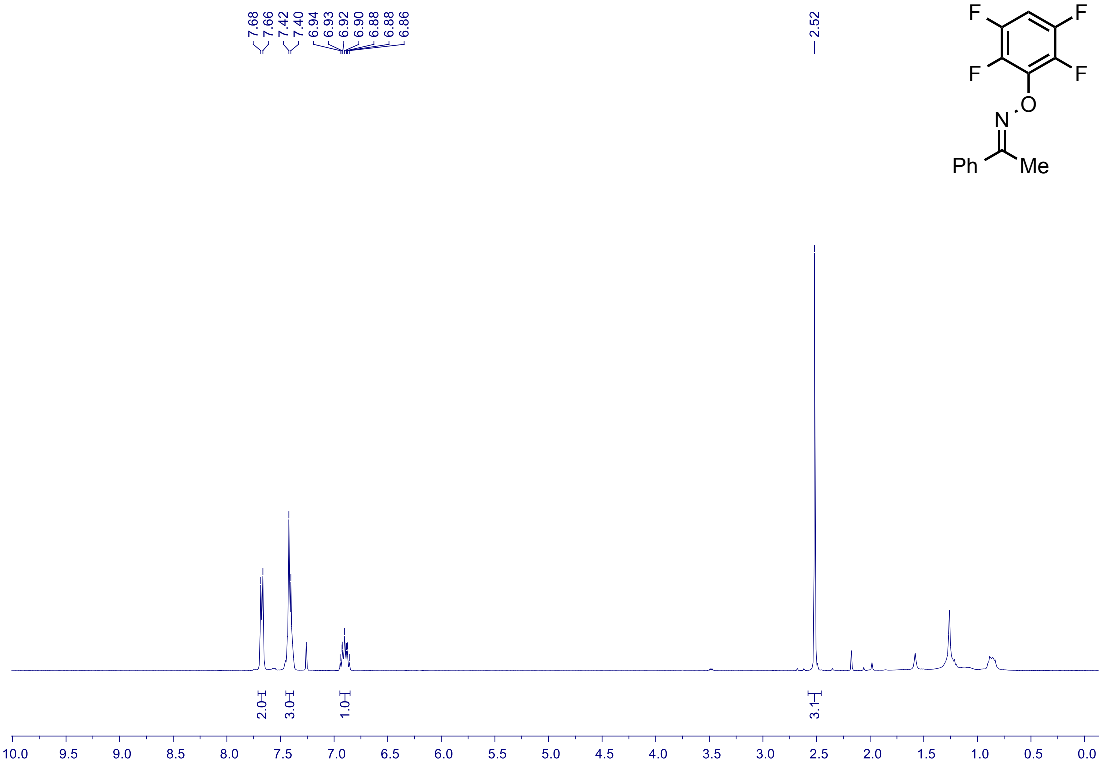

1f: ^{13}C NMR (101 MHz, $CDCl_3$)

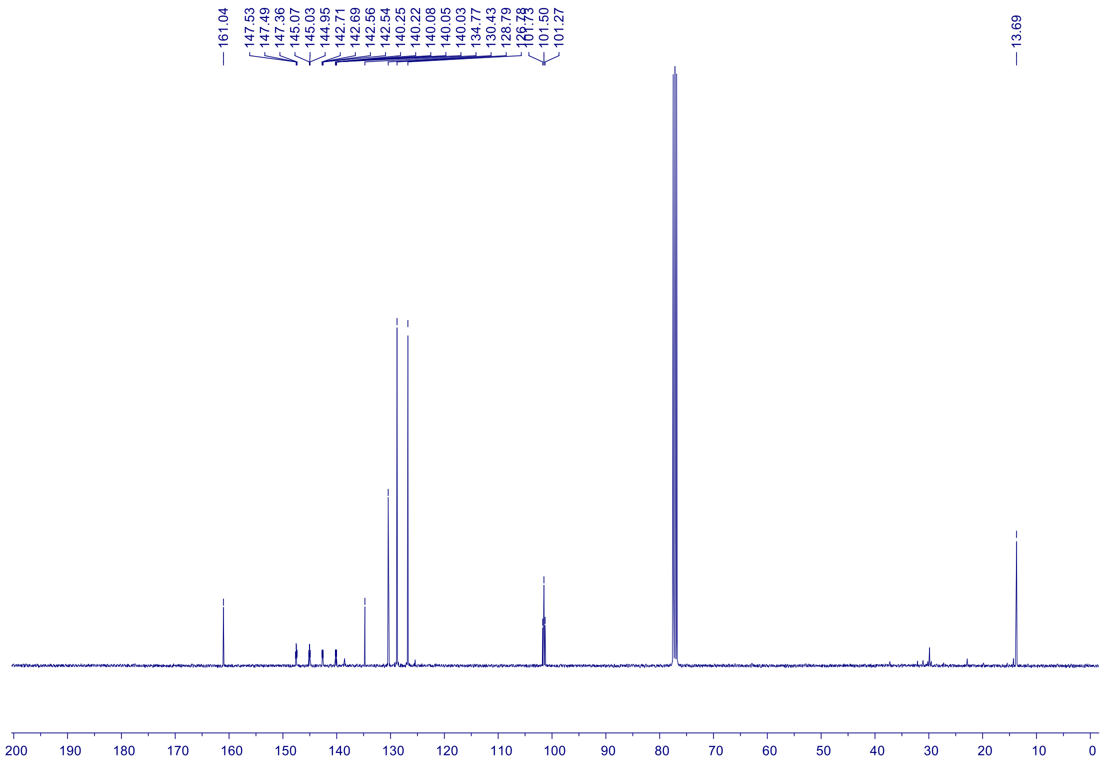

S7: ^{1}H NMR (400 MHz, CDCl$_3$)

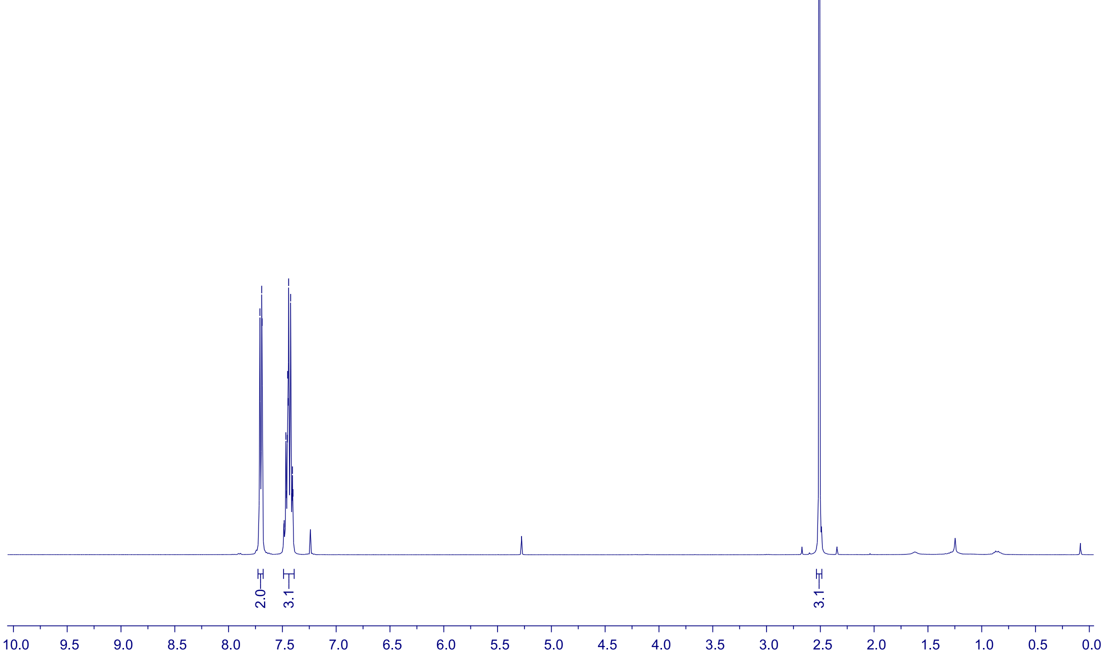

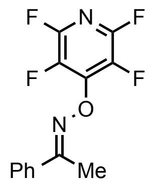

S7: ^{13}C NMR (101 MHz, CDCl$_3$)

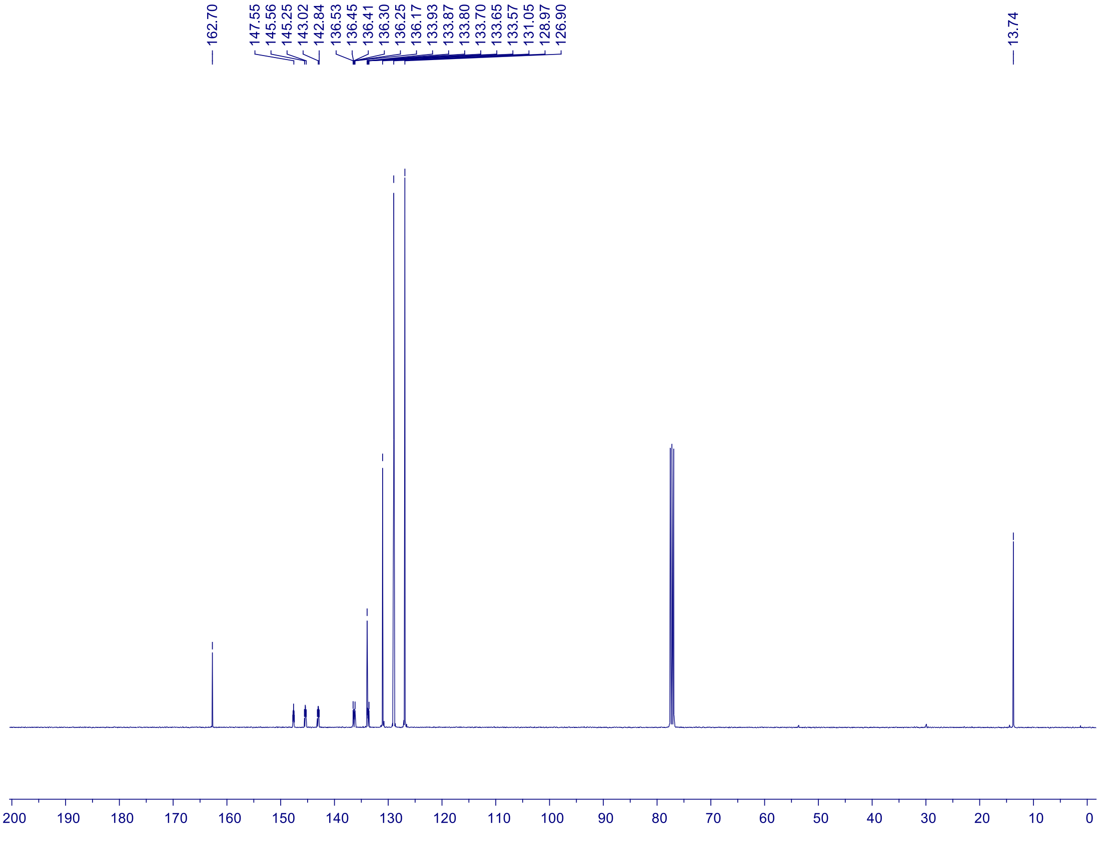

S15: ^{1}H NMR (400 MHz, $CDCl_3$)

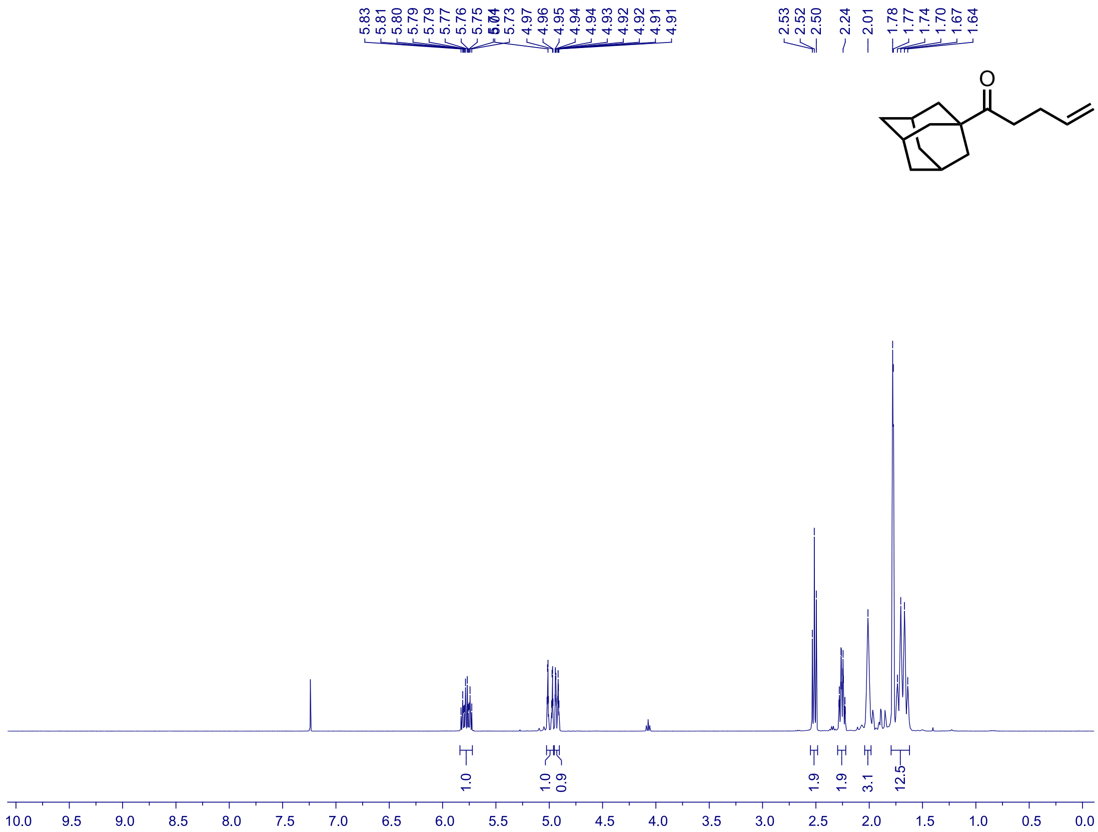

S15: ^{13}C NMR (101 MHz, $CDCl_3$)

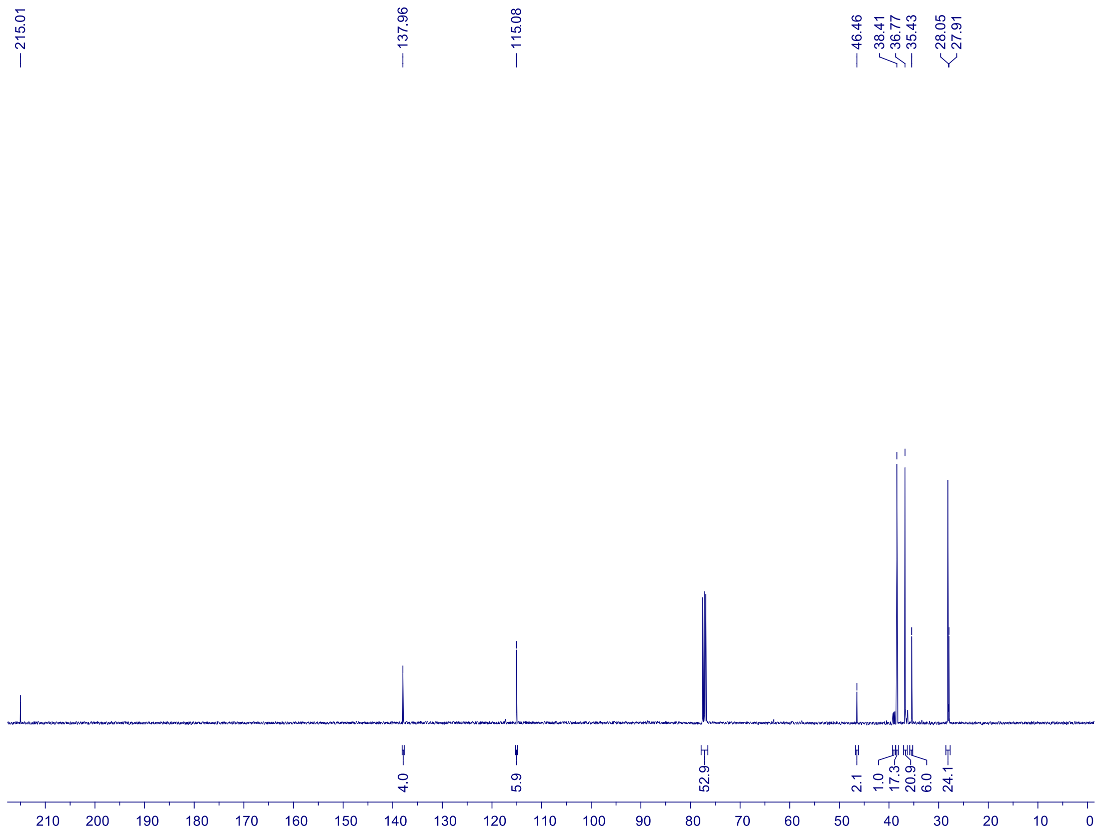

S29: ^{1}H NMR (400 MHz, $CDCl_3$)

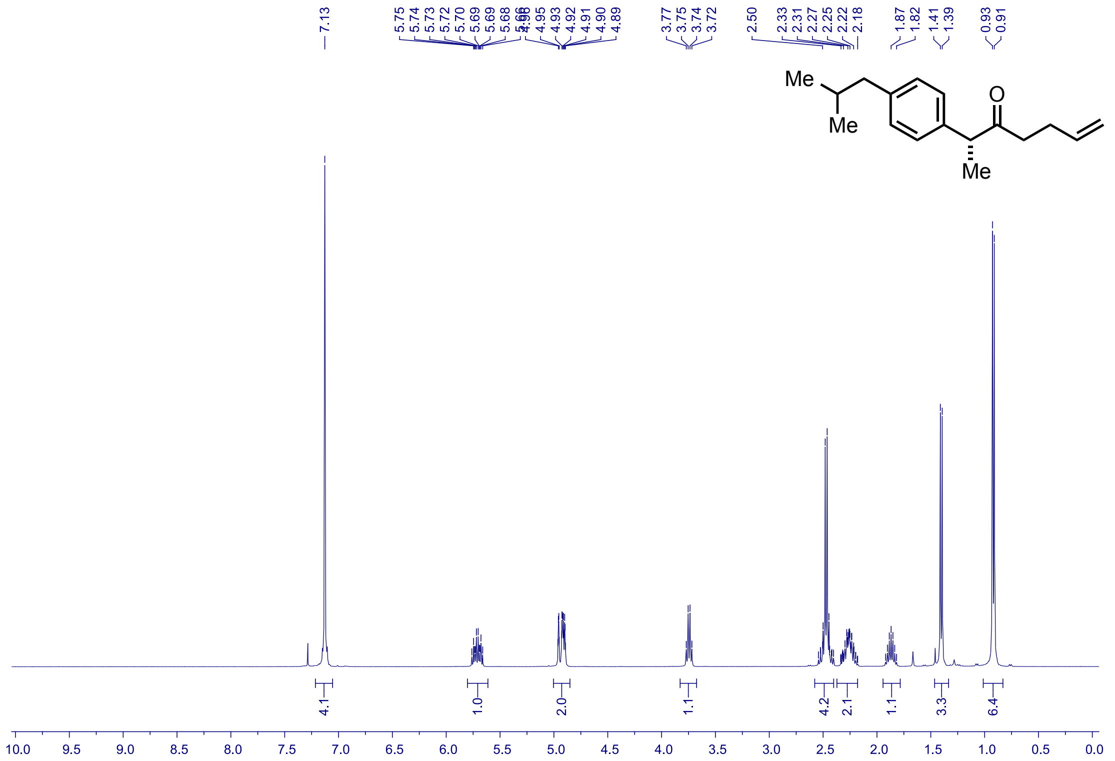

S29: ^{13}C NMR (101 MHz, $CDCl_3$)

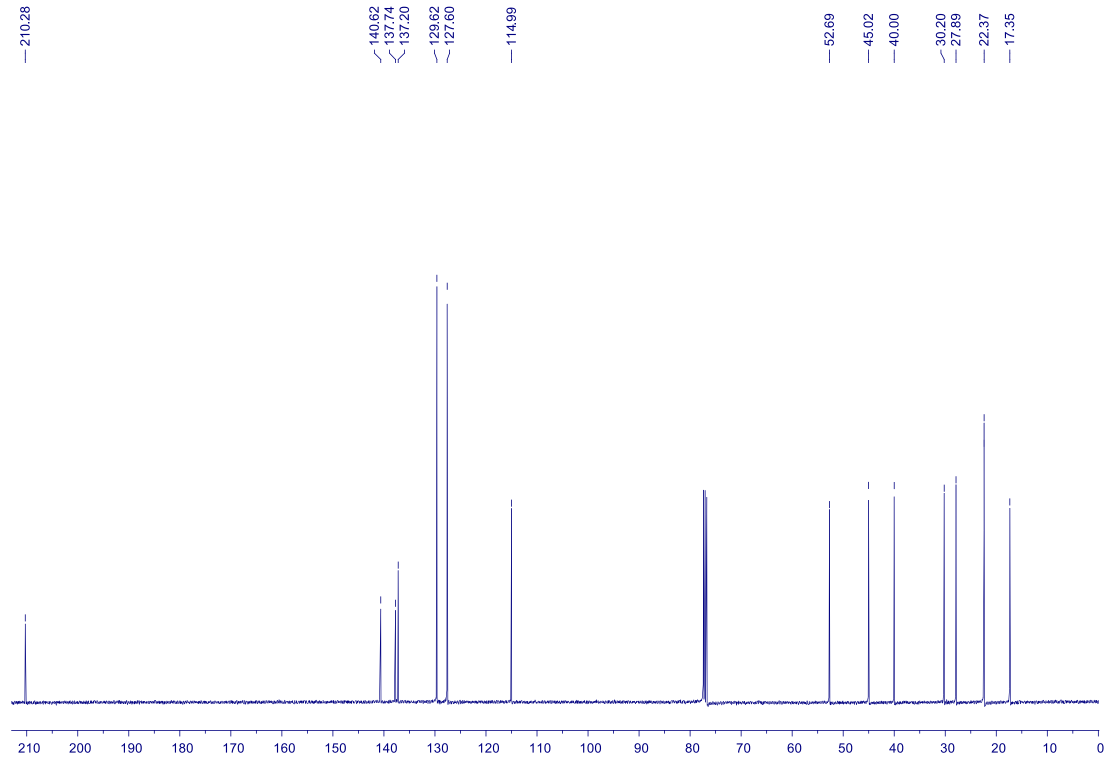

2a: ^{1}H NMR (400 MHz, $CDCl_3$)

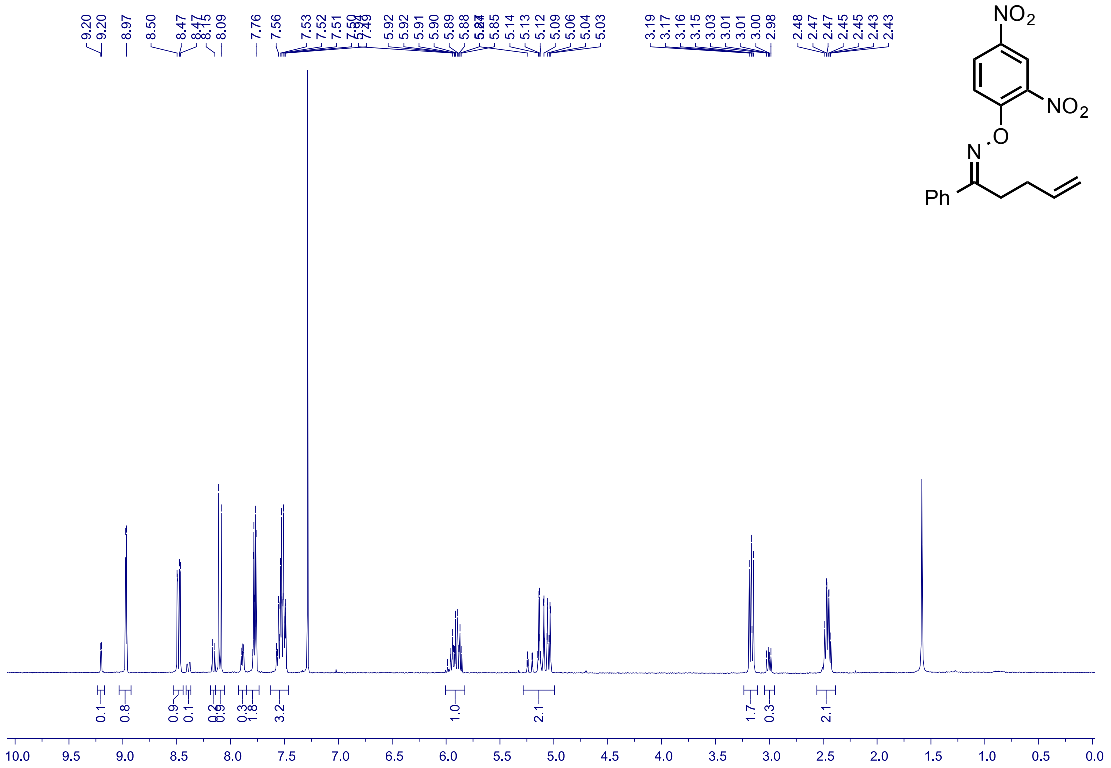

2a: ^{13}C NMR (101 MHz, $CDCl_3$)

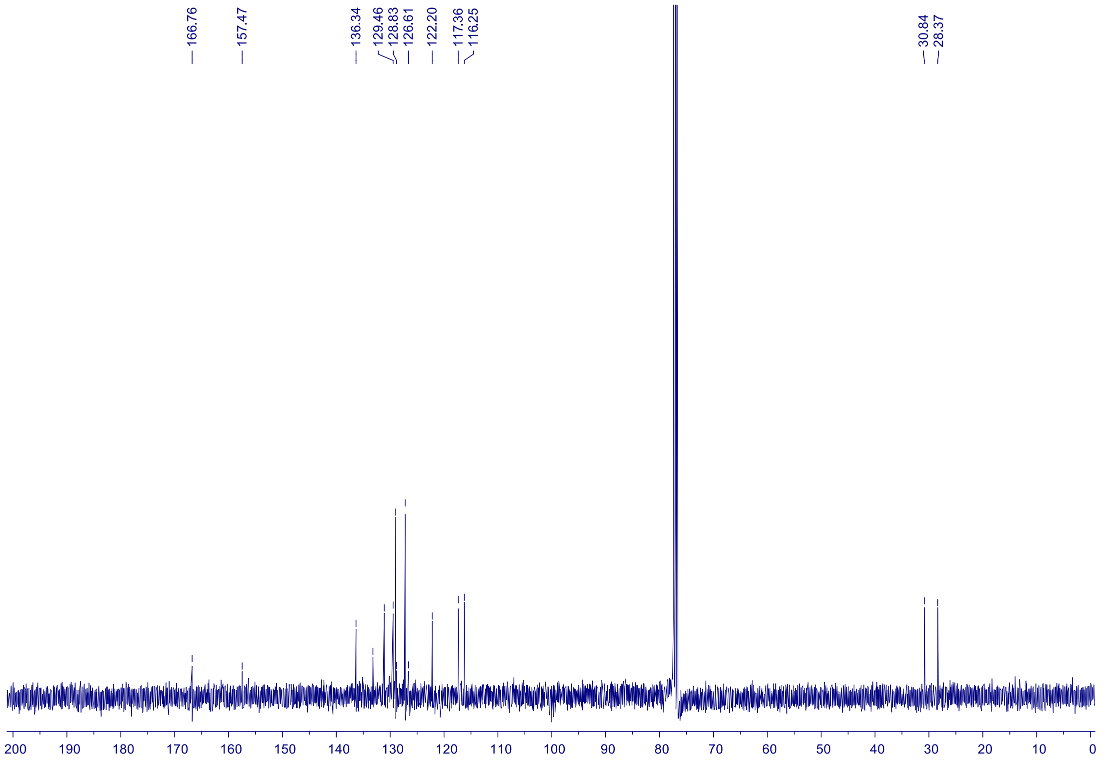

2b: ^{1}H NMR (400 MHz, $CDCl_3$)

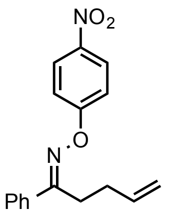

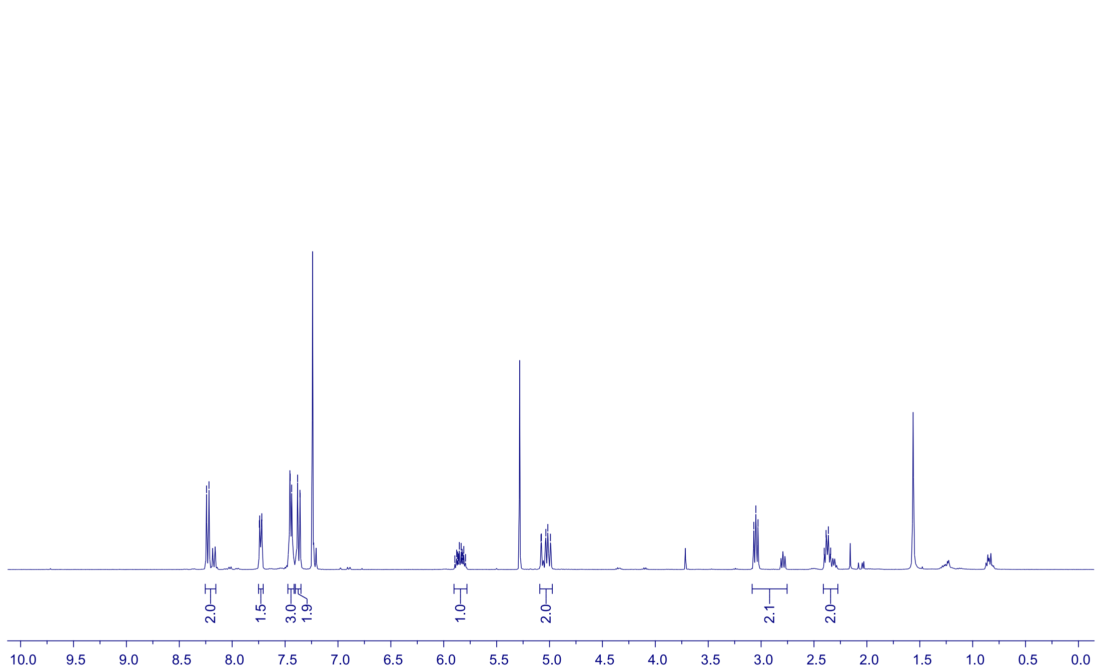

2b: ^{13}C NMR (101 MHz, $CDCl_3$)

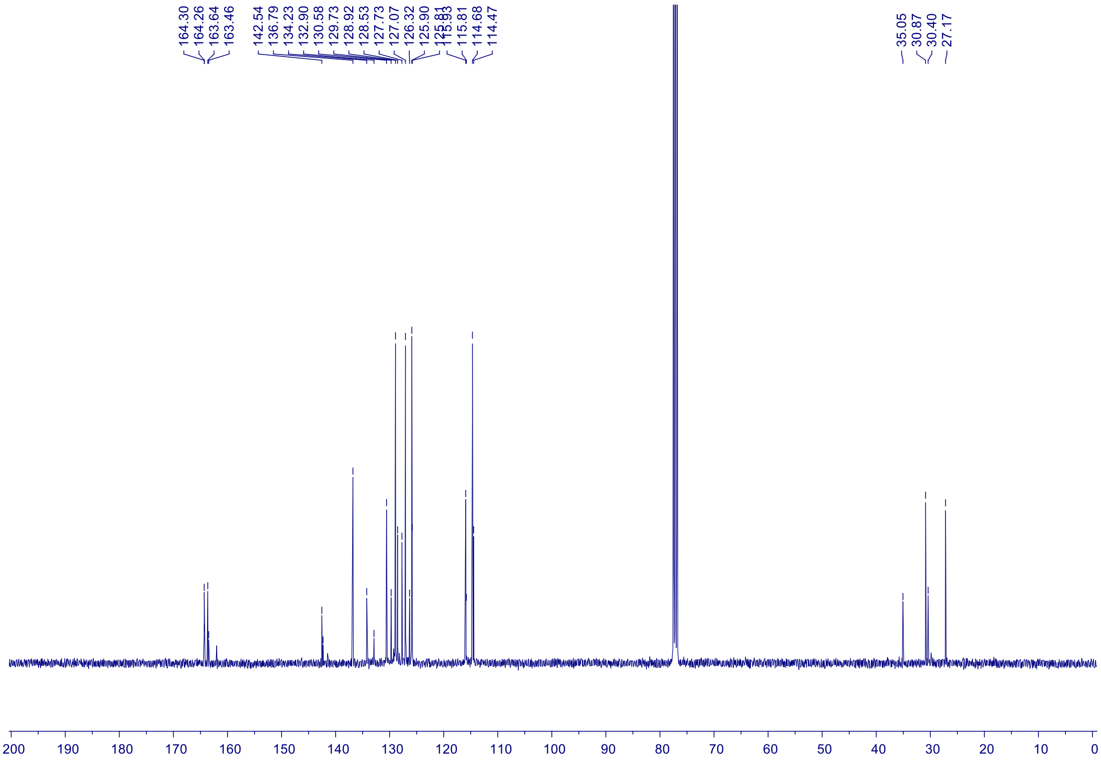

2c: ^{1}H NMR (400 MHz, $CDCl_3$)

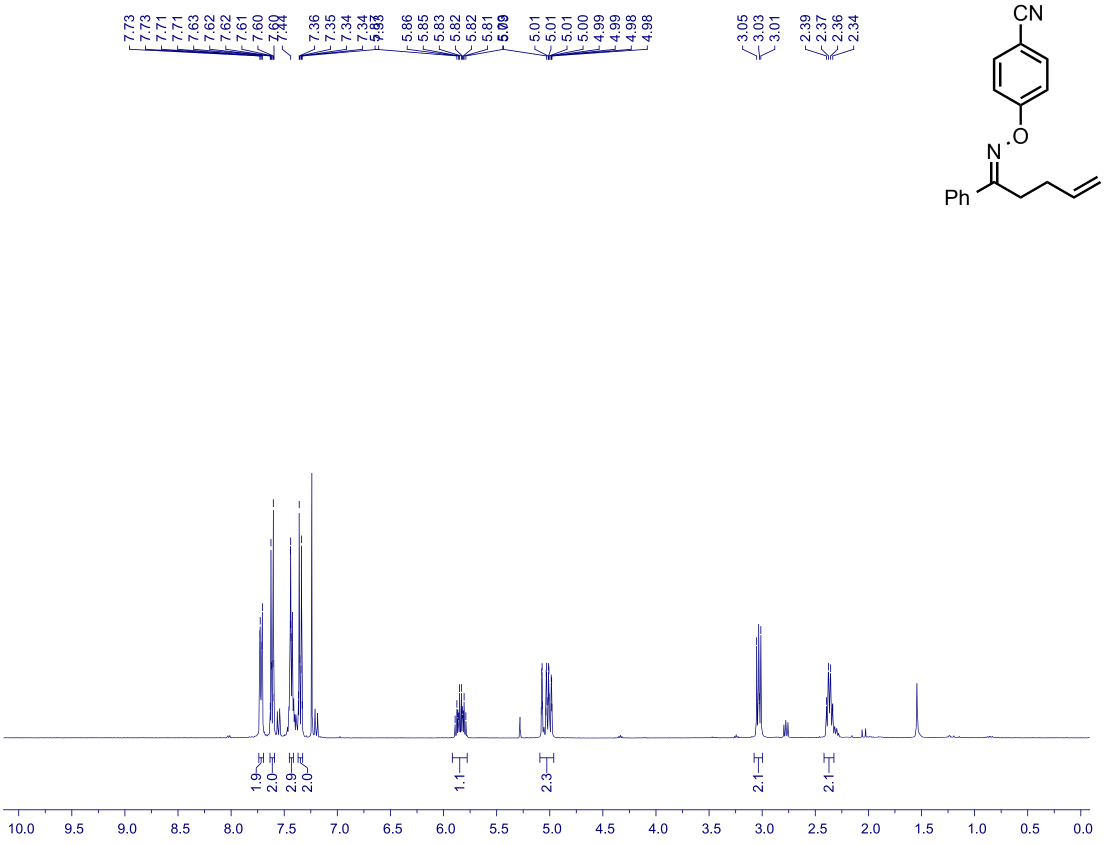

2c: ^{13}C NMR (101 MHz, $CDCl_3$)

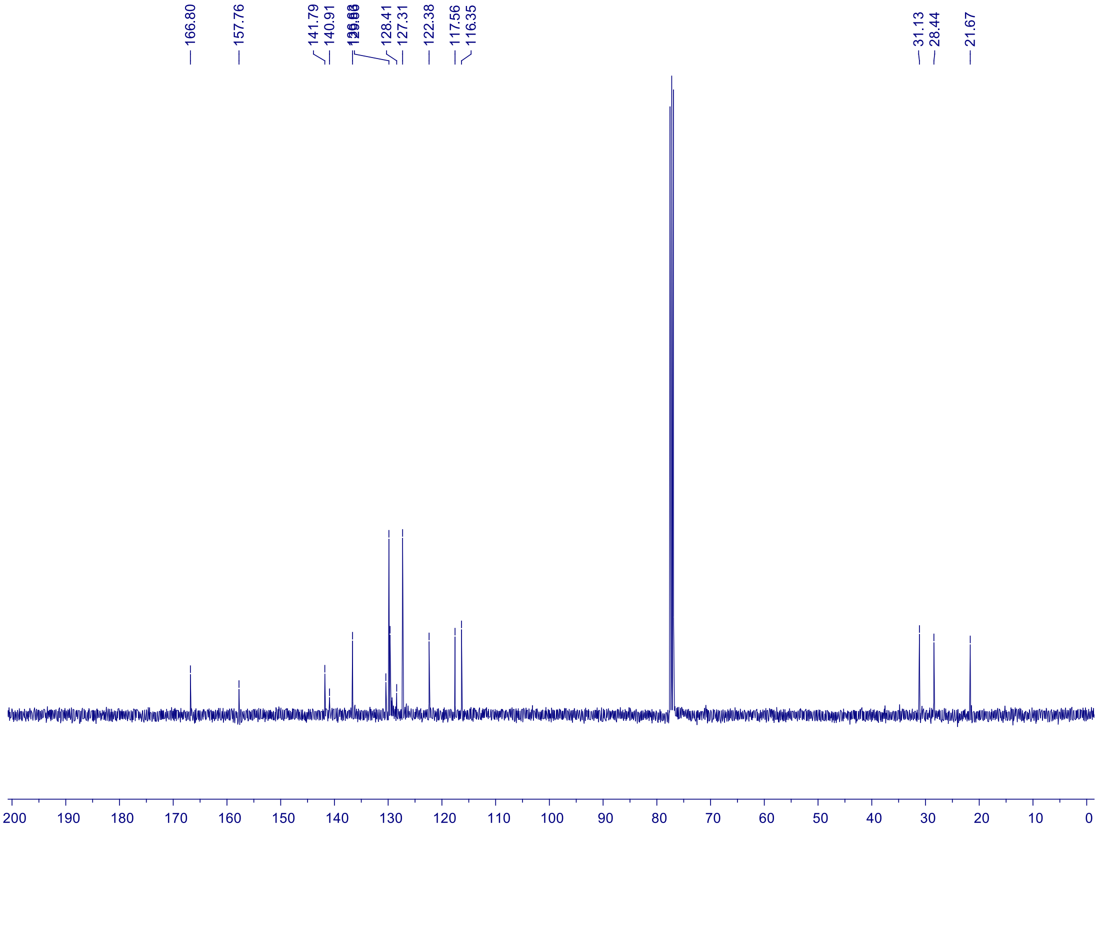

2d: ^{1}H NMR (400 MHz, $CDCl_3$)

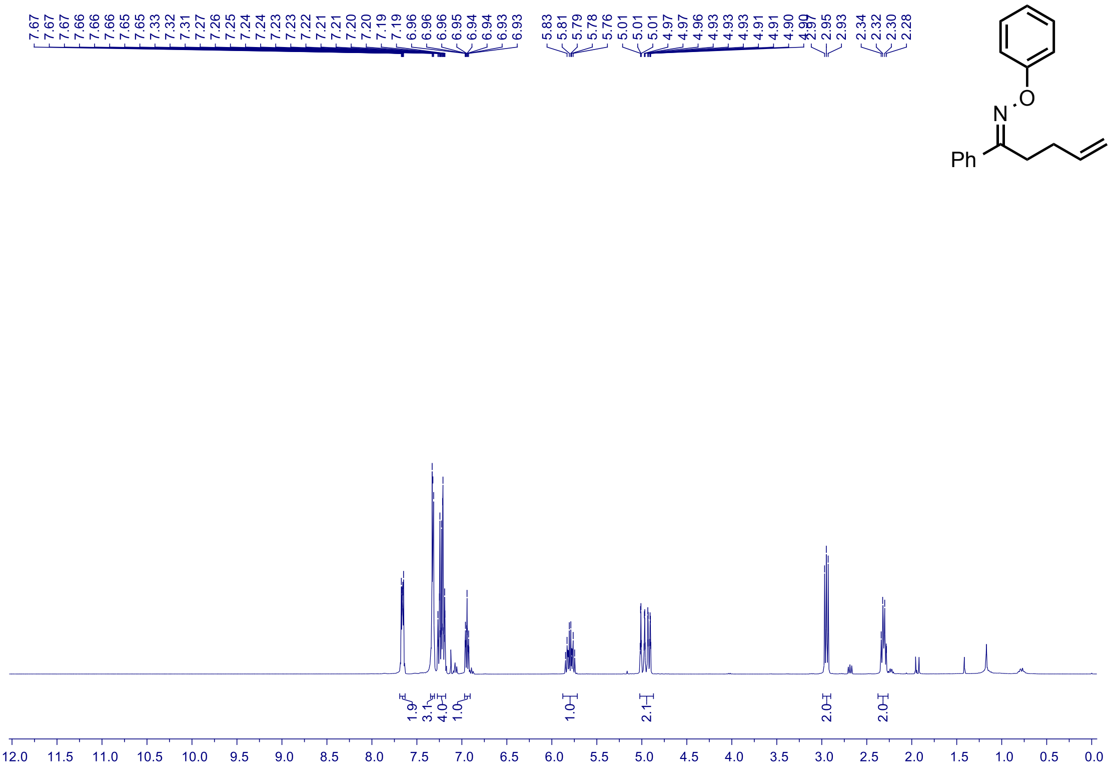

2d: ^{13}C NMR (101 MHz, $CDCl_3$)

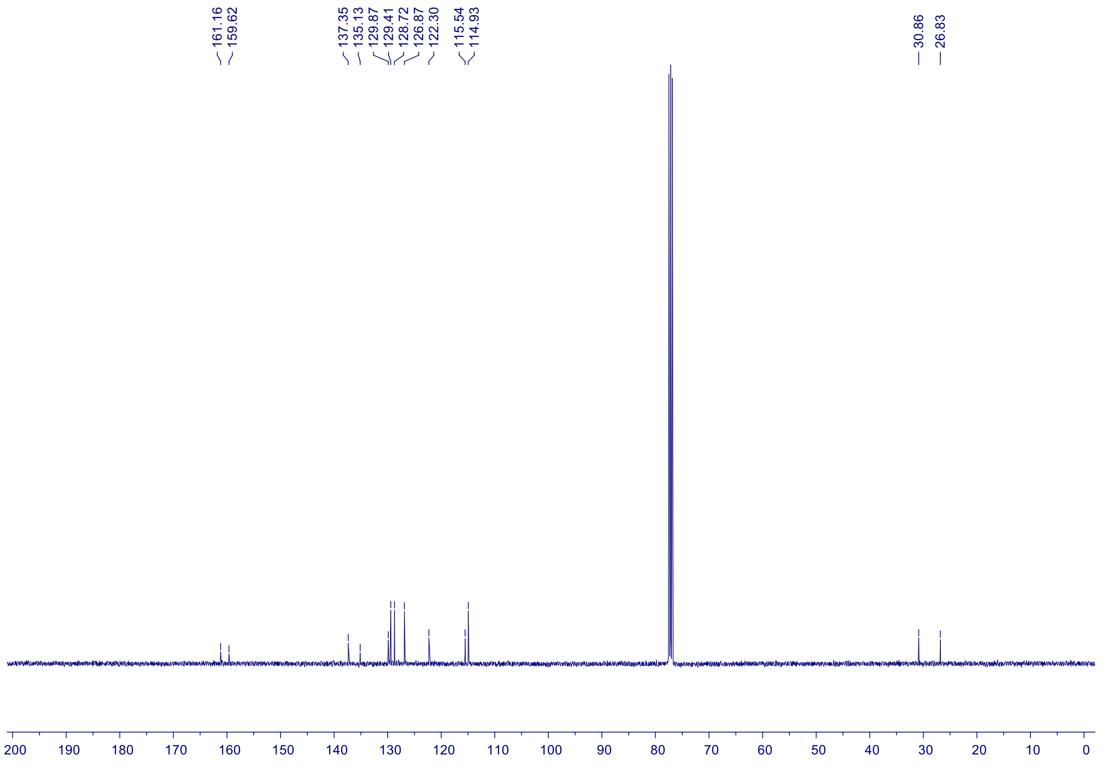

2e: ^{1}H NMR (400 MHz, $CDCl_3$)

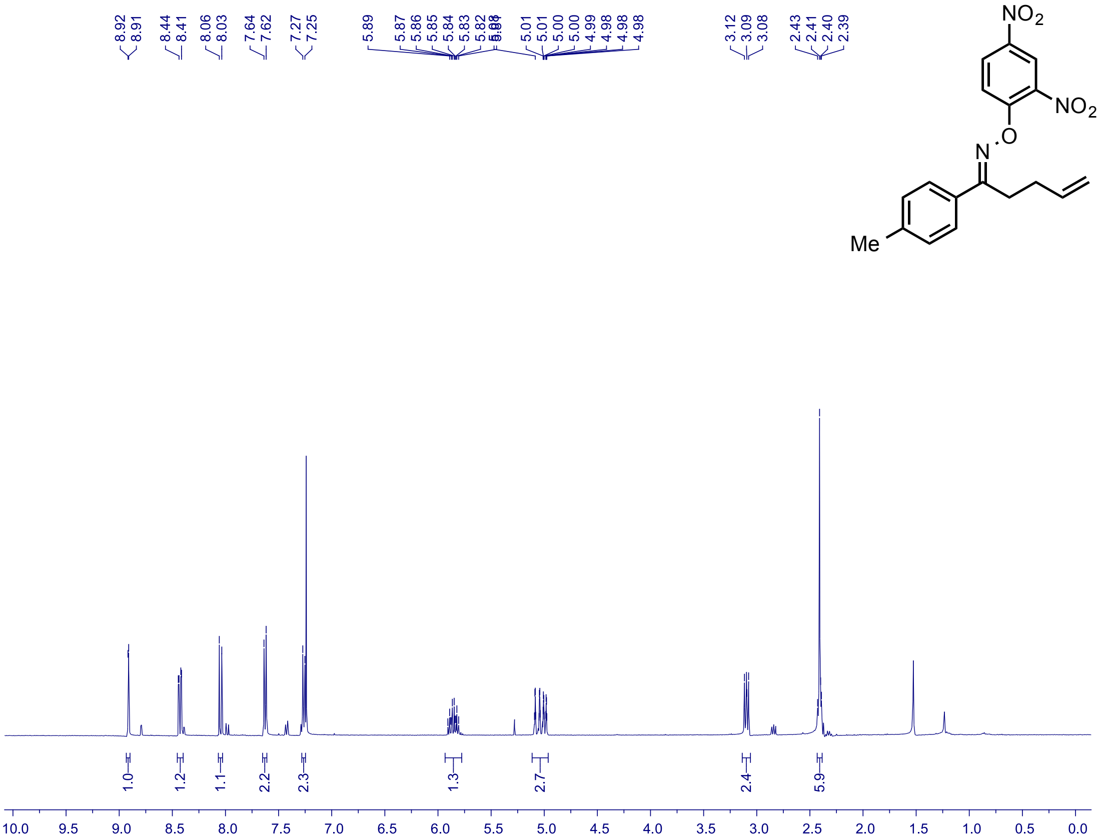

2e: ^{13}C NMR (101 MHz, $CDCl_3$)

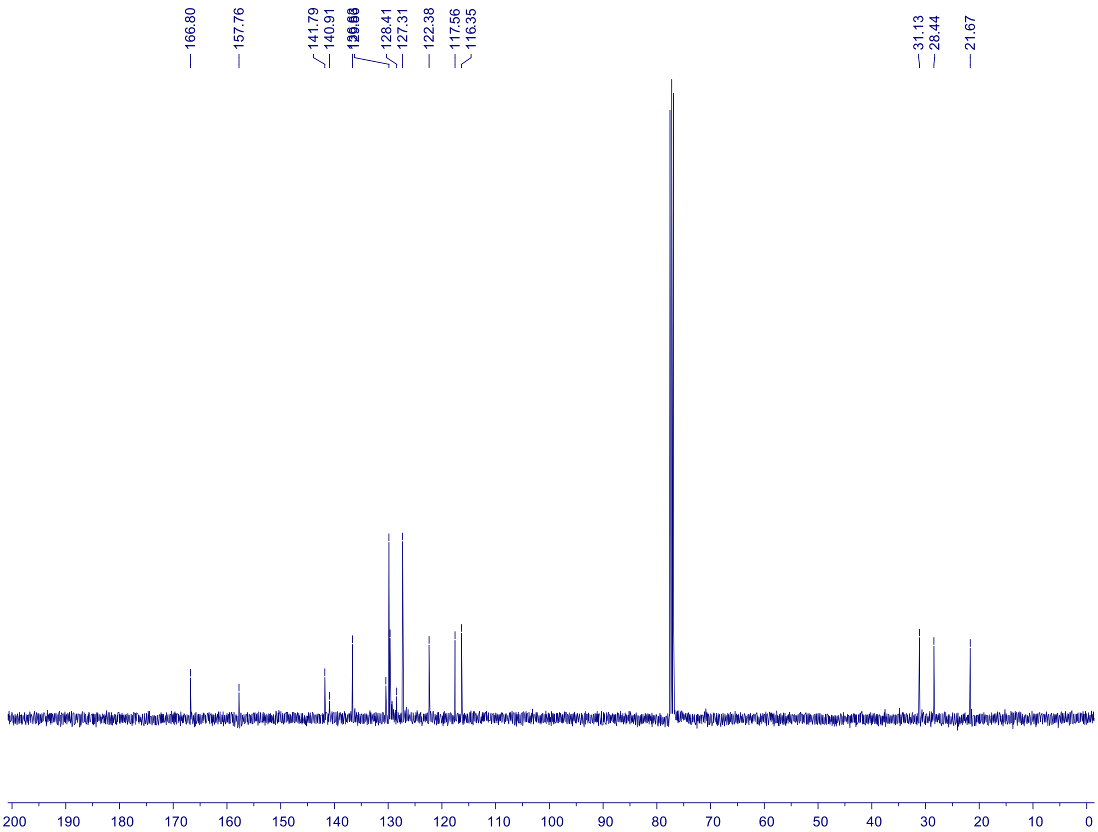

2f: ^{1}H NMR (400 MHz, $CDCl_3$)

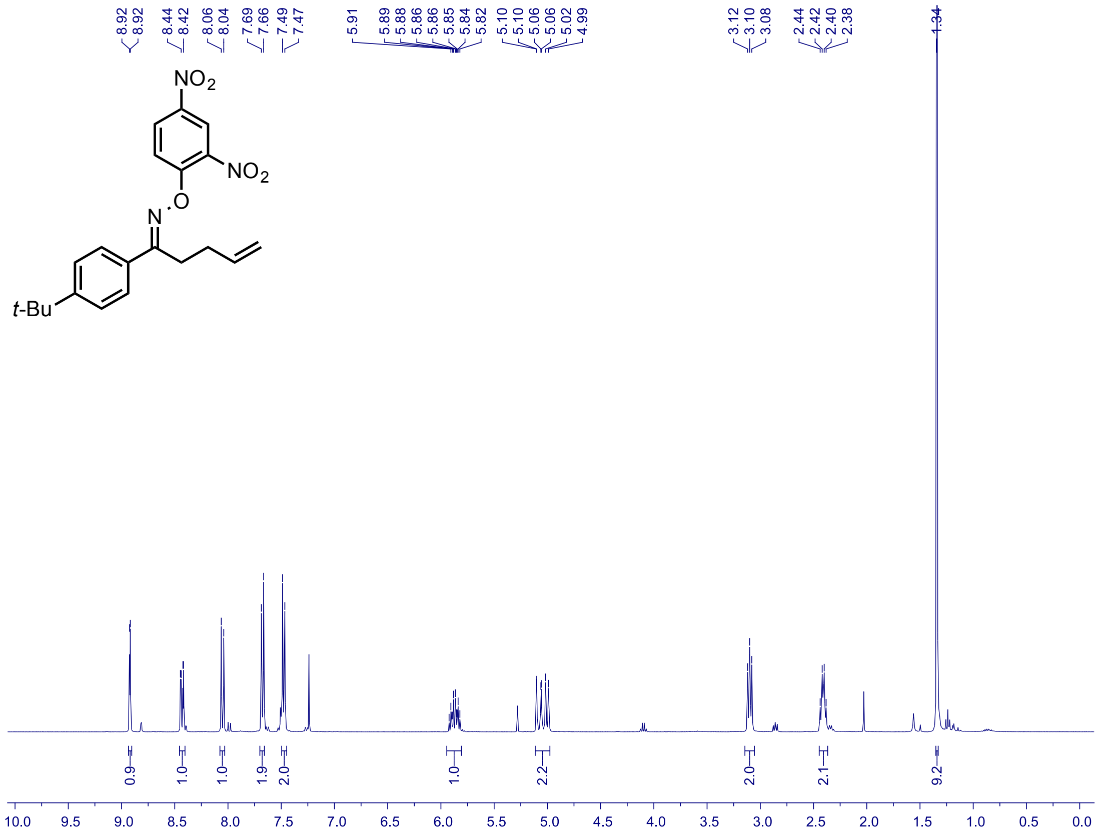

2f: ^{13}C NMR (101 MHz, $CDCl_3$)

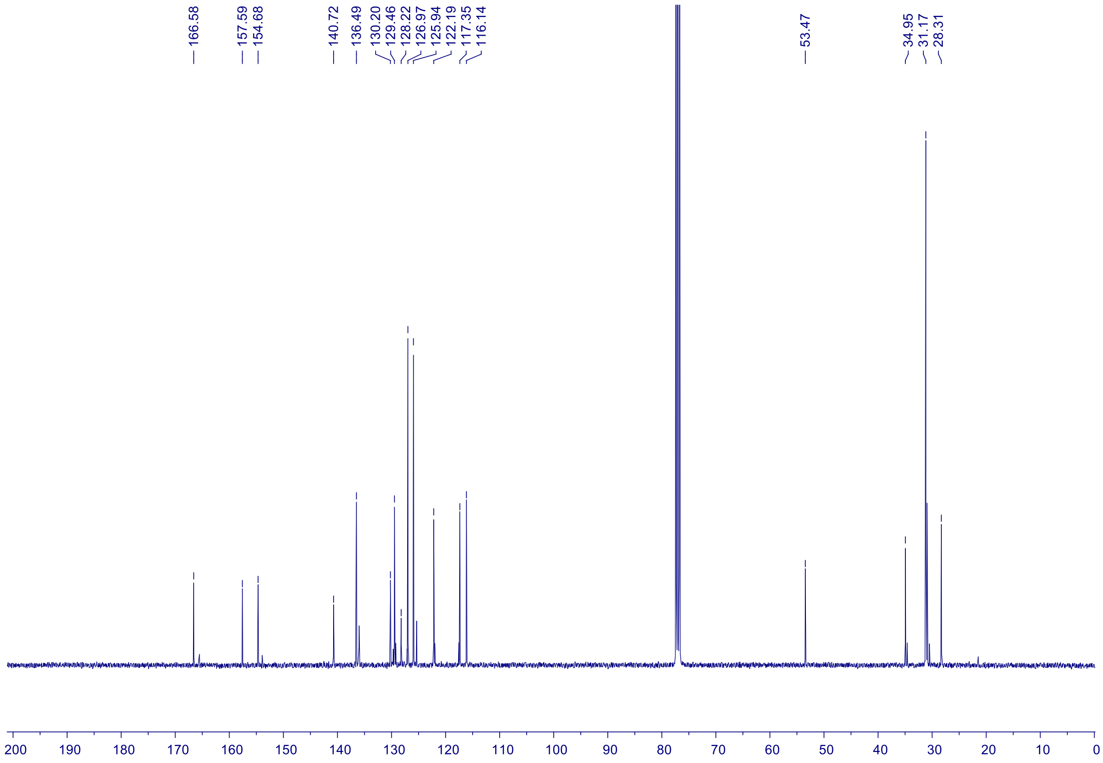

2g: ^{1}H NMR (400 MHz, $CDCl_3$)

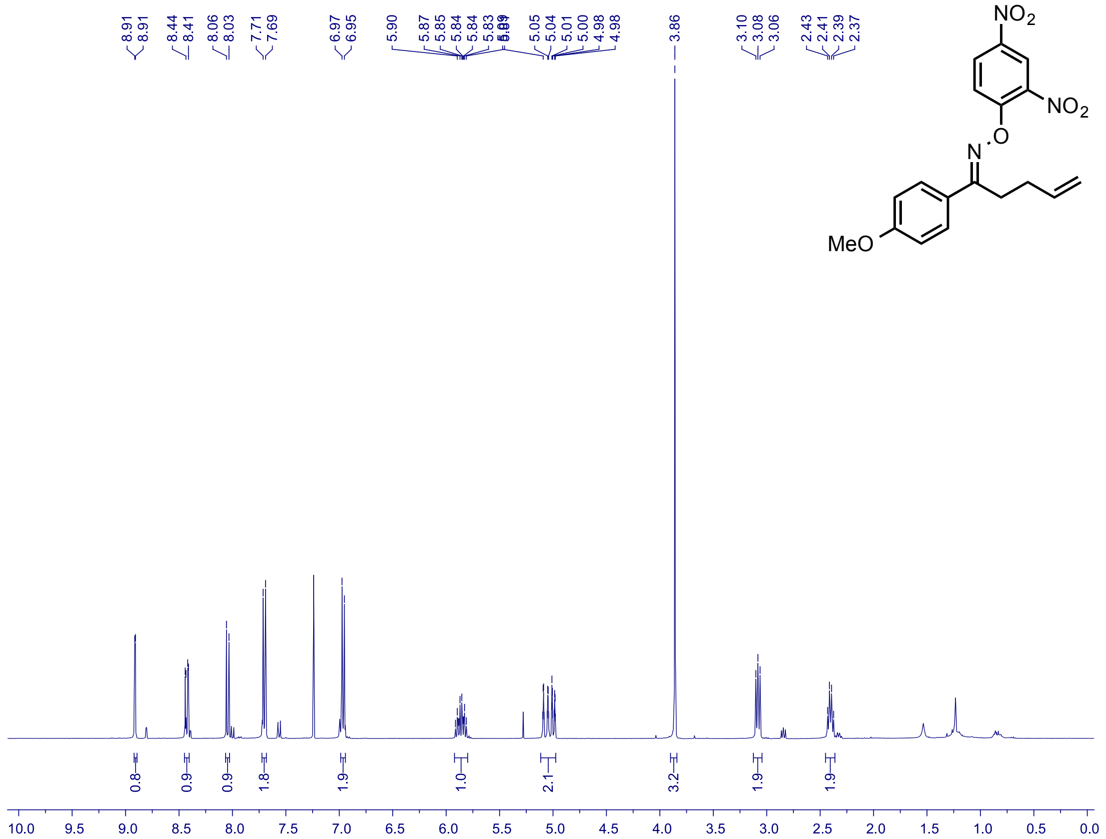

2g: ^{13}C NMR (101 MHz, $CDCl_3$)

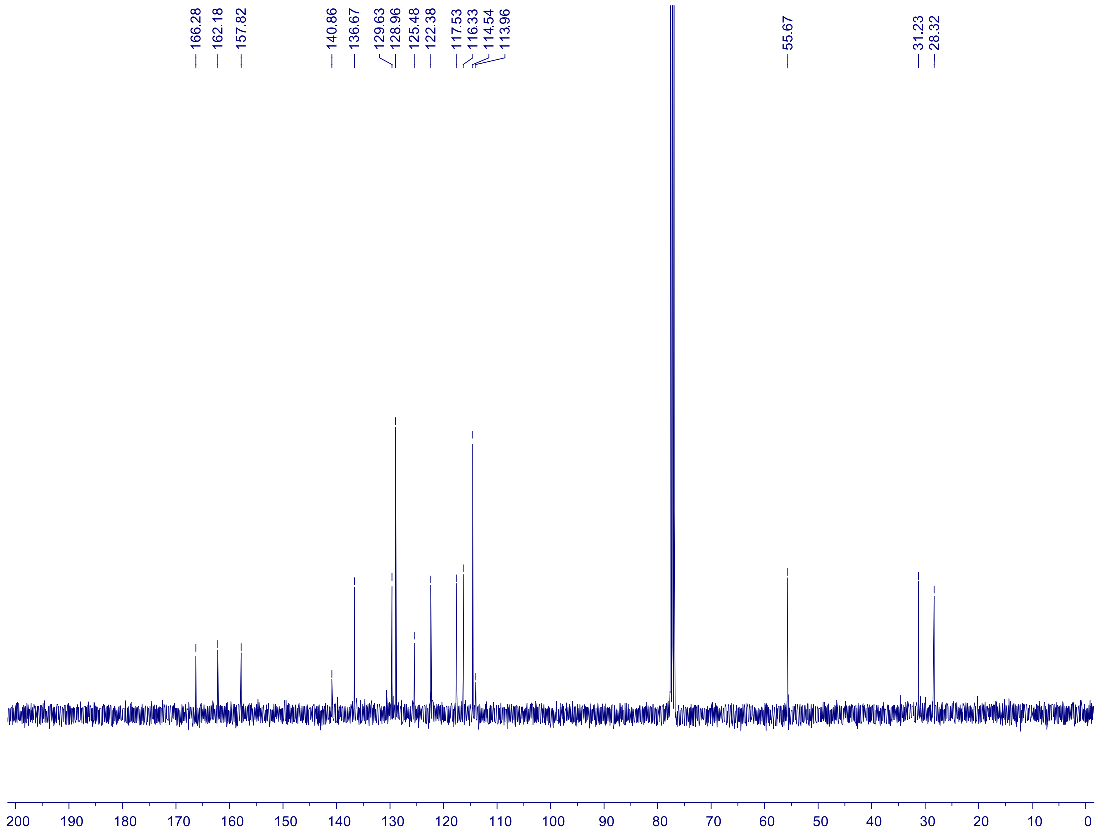

2h: ^{1}H NMR (400 MHz, $CDCl_3$)

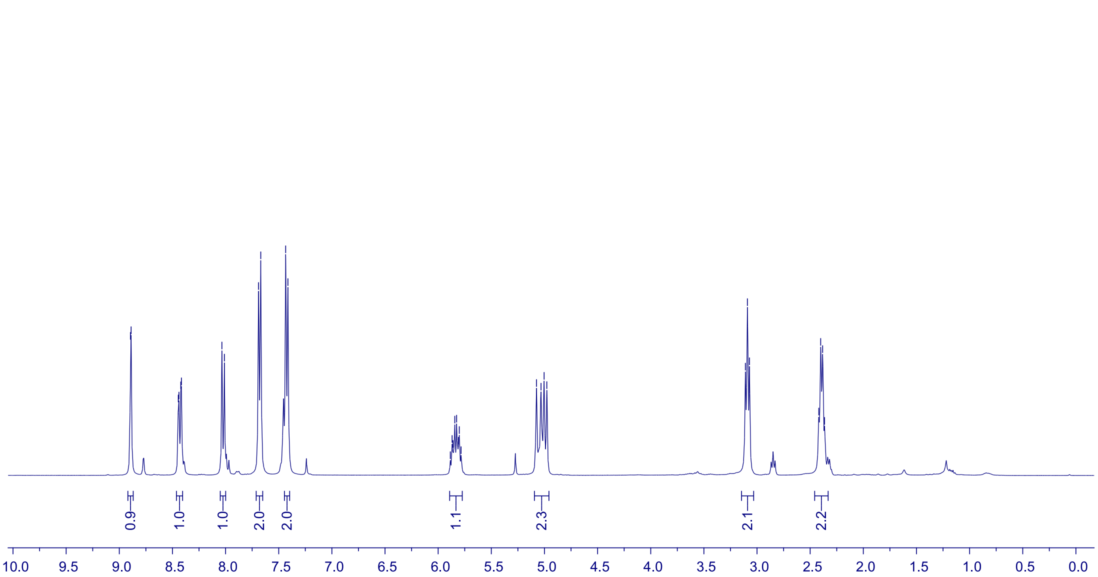

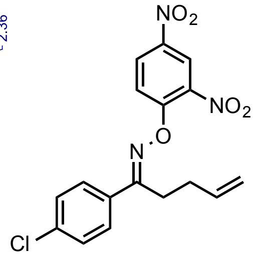

2h: ^{13}C NMR (101 MHz, $CDCl_3$)

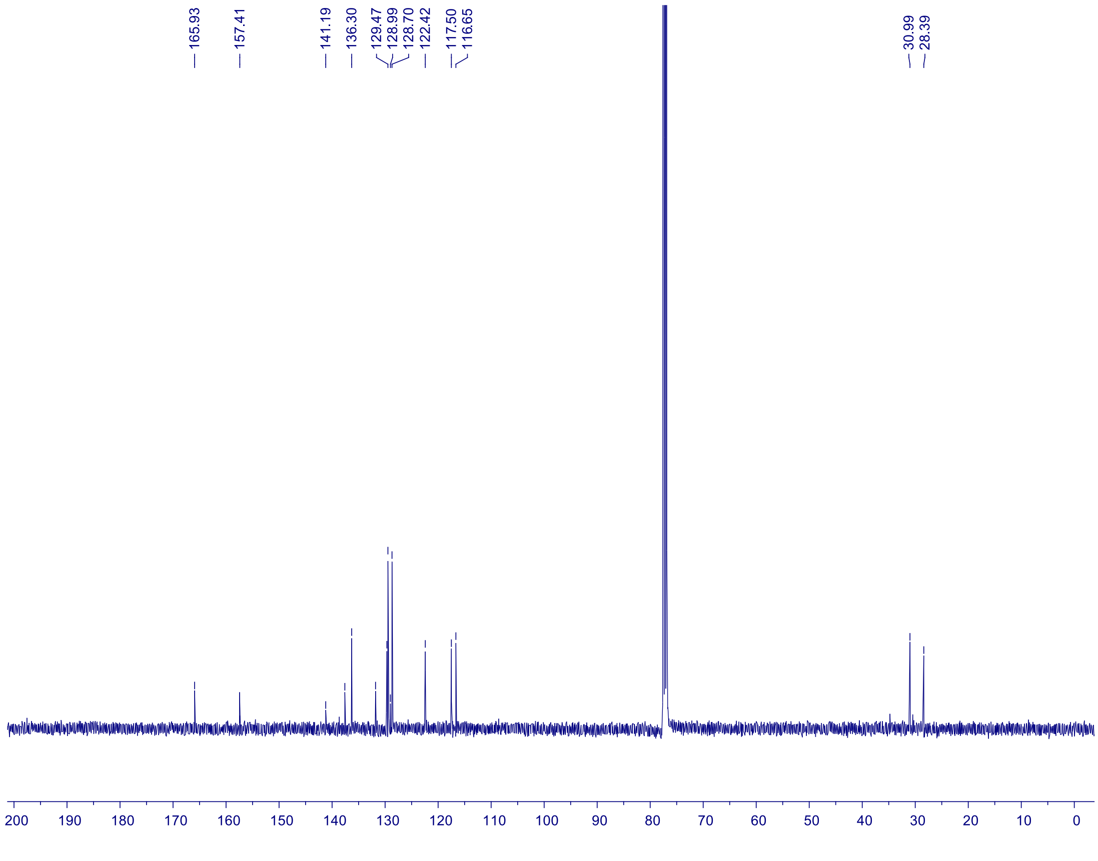

2i: ^{1}H NMR (400 MHz, $CDCl_3$)

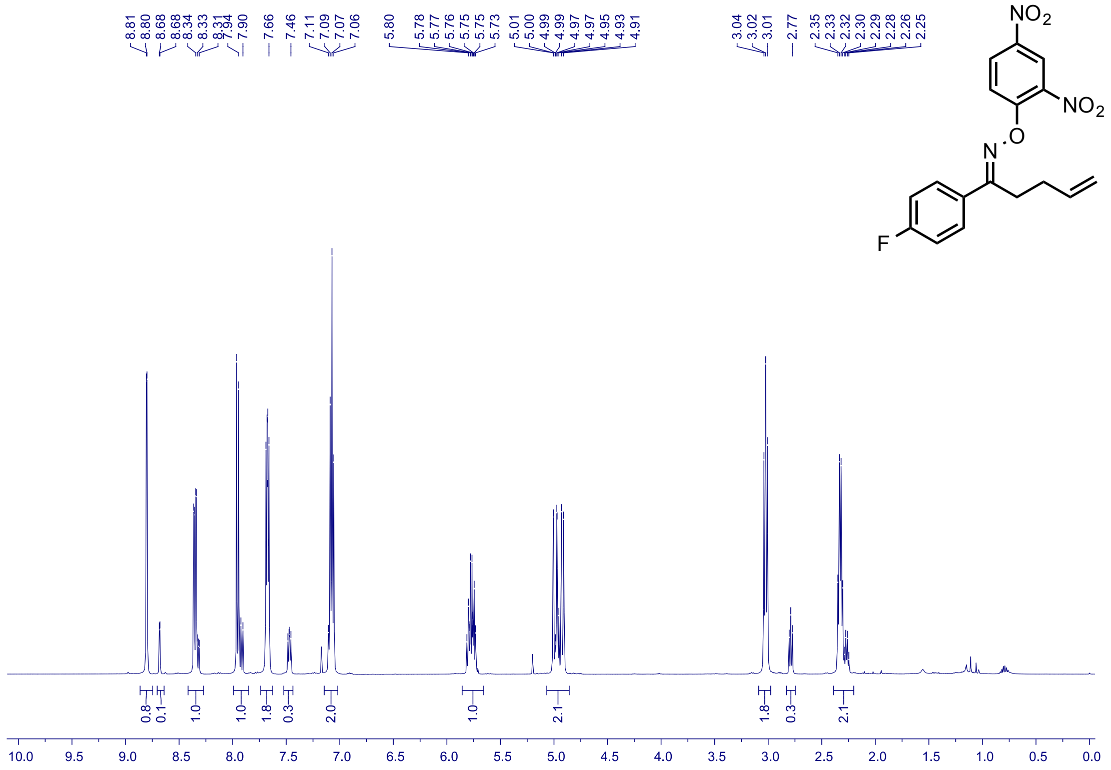

2i: ^{13}C NMR (101 MHz, $CDCl_3$)

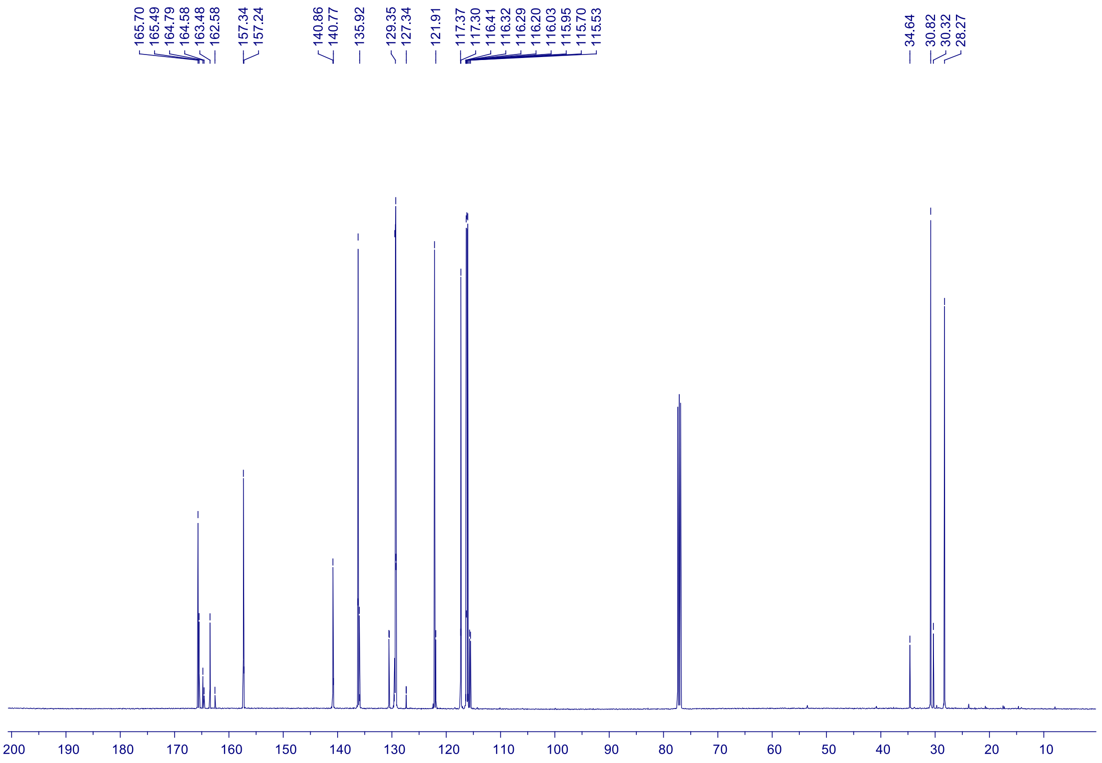

2j: ^{1}H NMR (400 MHz, $CDCl_3$)

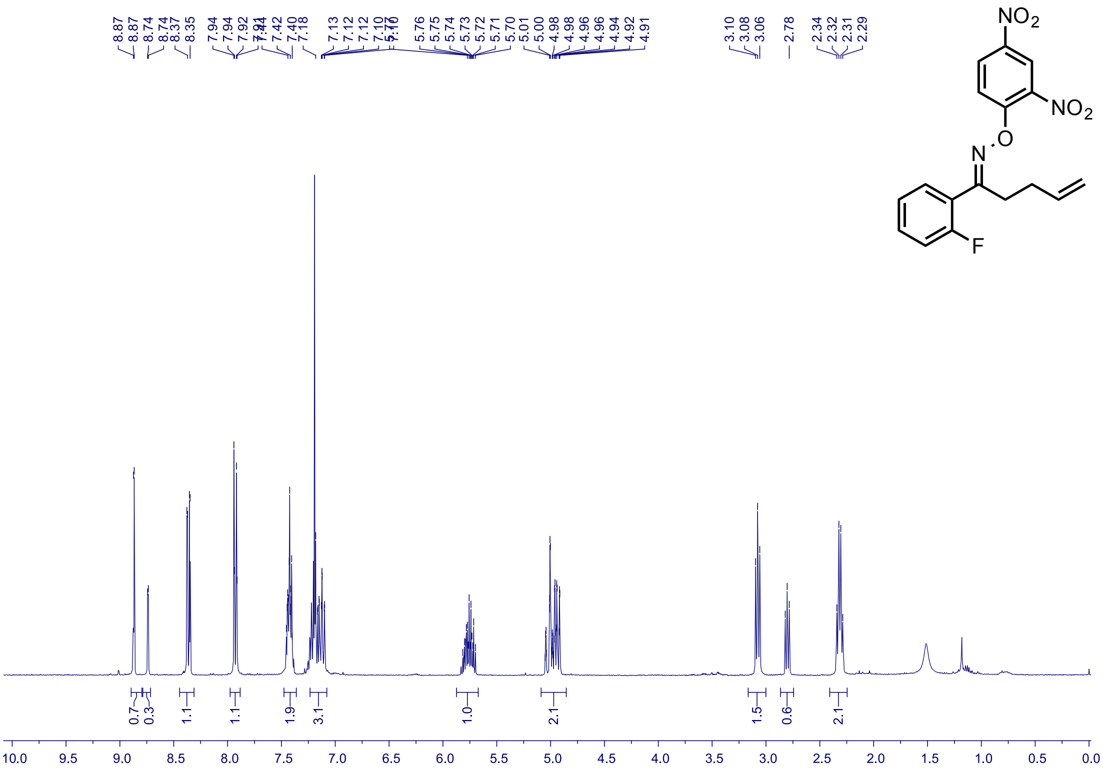

2j: ^{13}C NMR (101 MHz, $CDCl_3$)

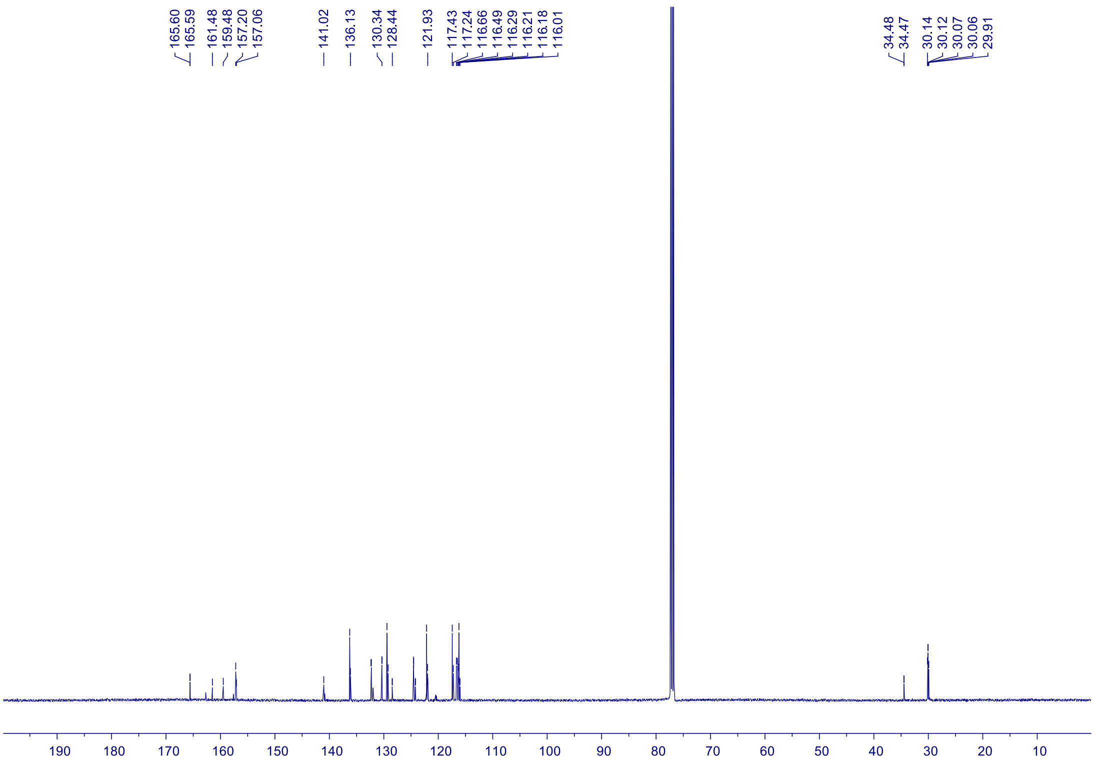

2k: ^{1}H NMR (400 MHz, $CDCl_3$)

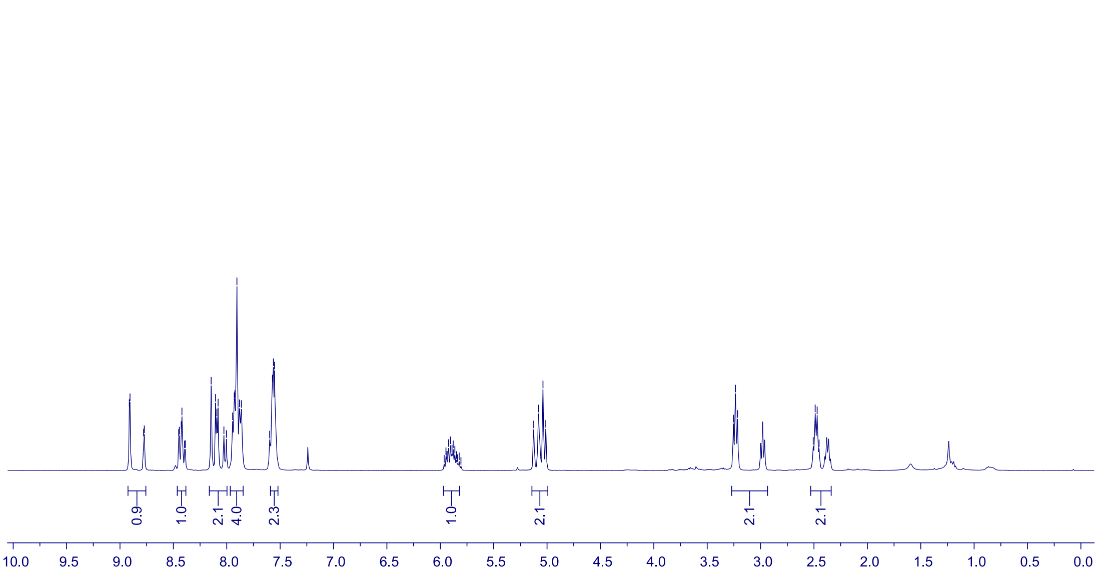

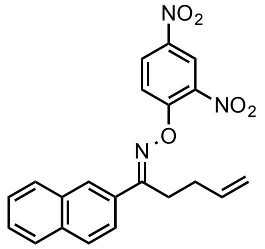

2k: ^{13}C NMR (101 MHz, $CDCl_3$)

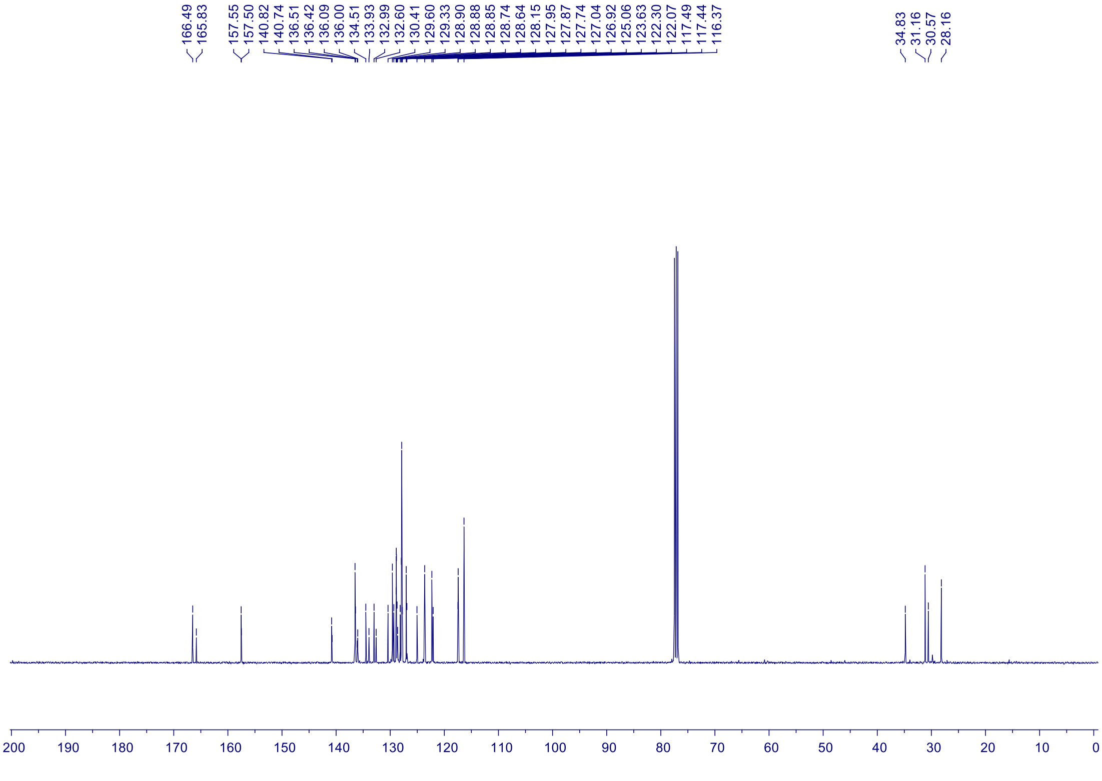

2l: ^{1}H NMR (400 MHz, $CDCl_3$)

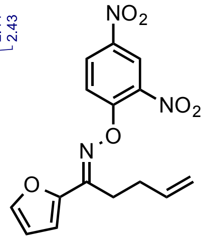

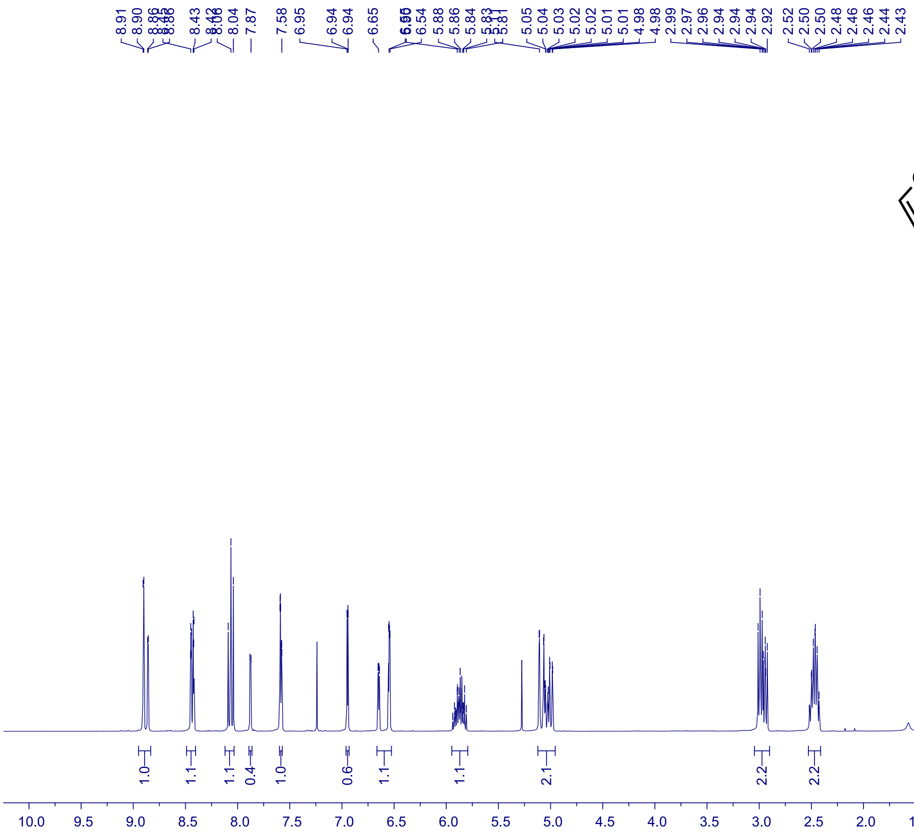

2l: ^{13}C NMR (101 MHz, $CDCl_3$)

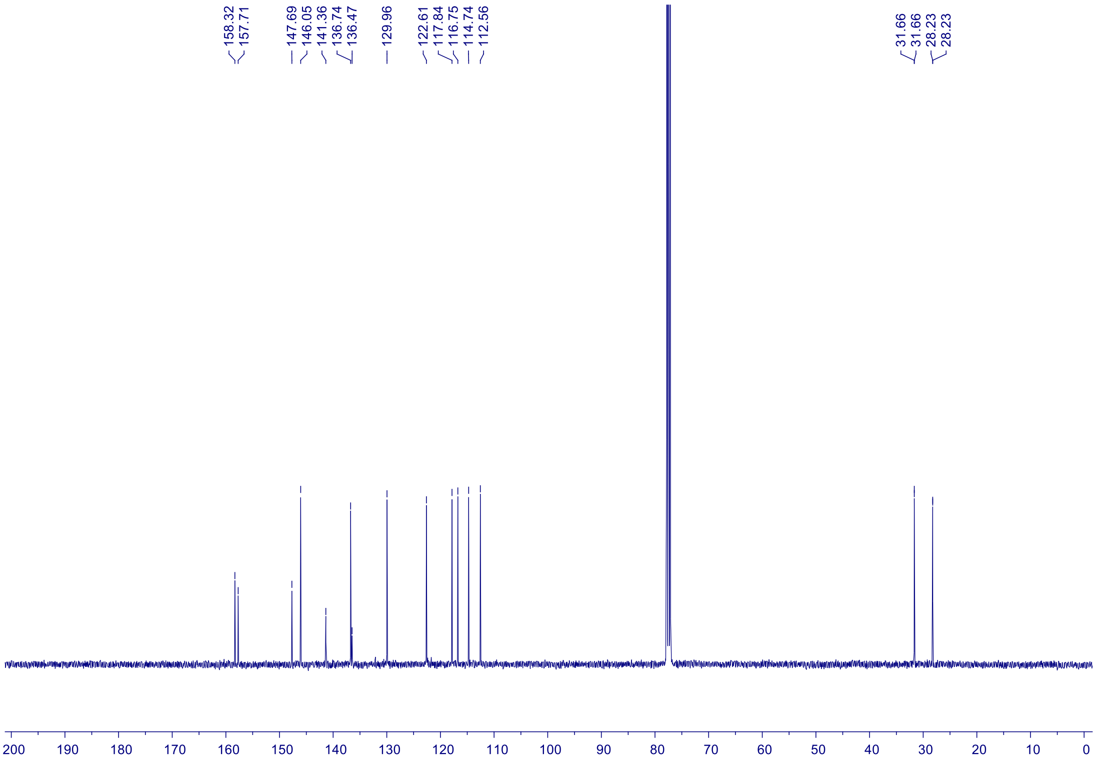

2m: ^{1}H NMR (400 MHz, $CDCl_3$)

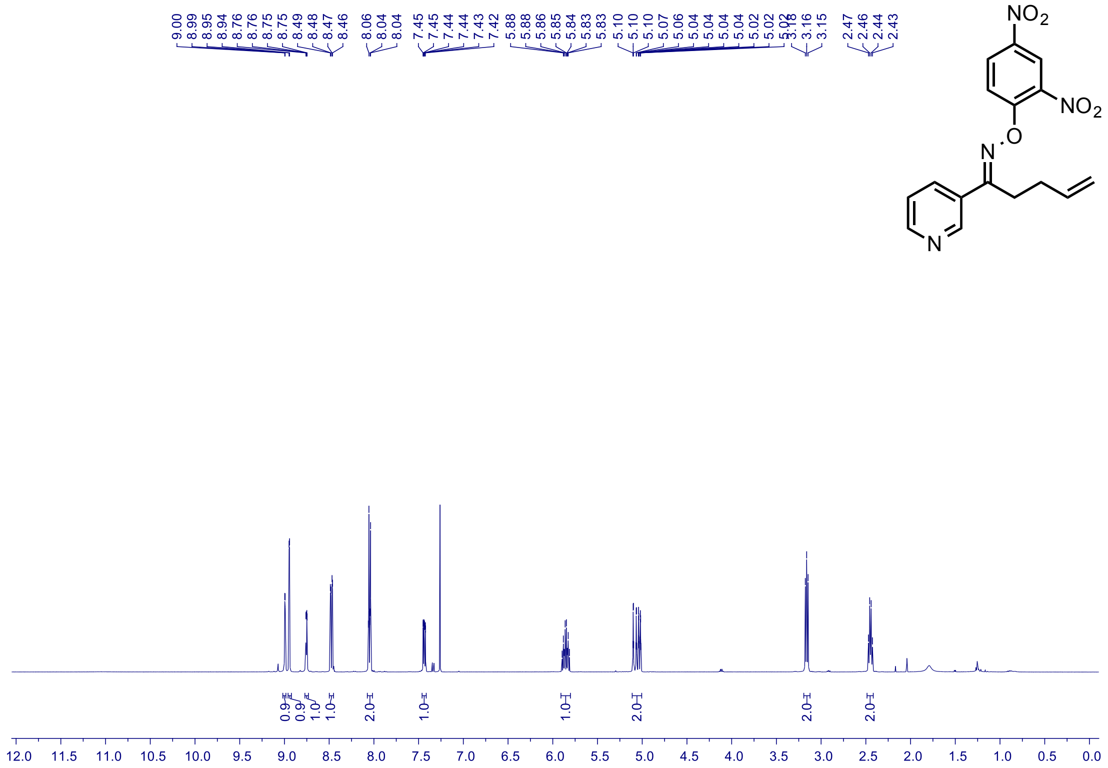

2m: ^{13}C NMR (101 MHz, $CDCl_3$)

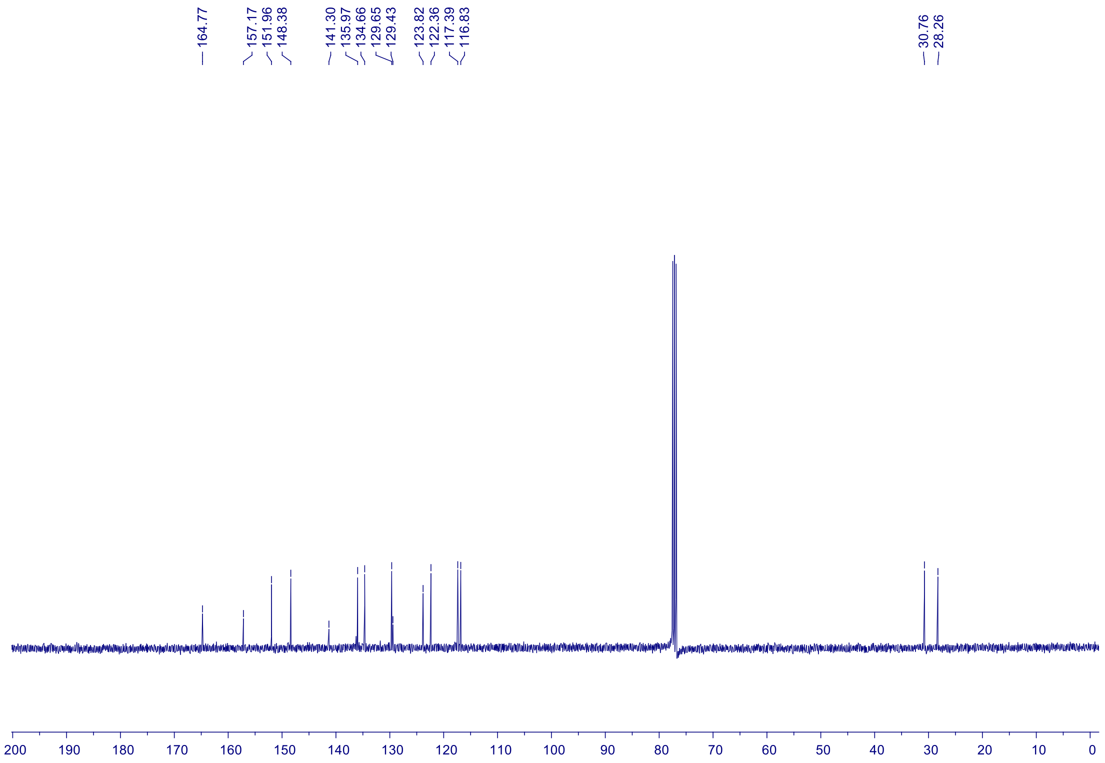

2n: ^{1}H NMR (400 MHz, $CDCl_3$)

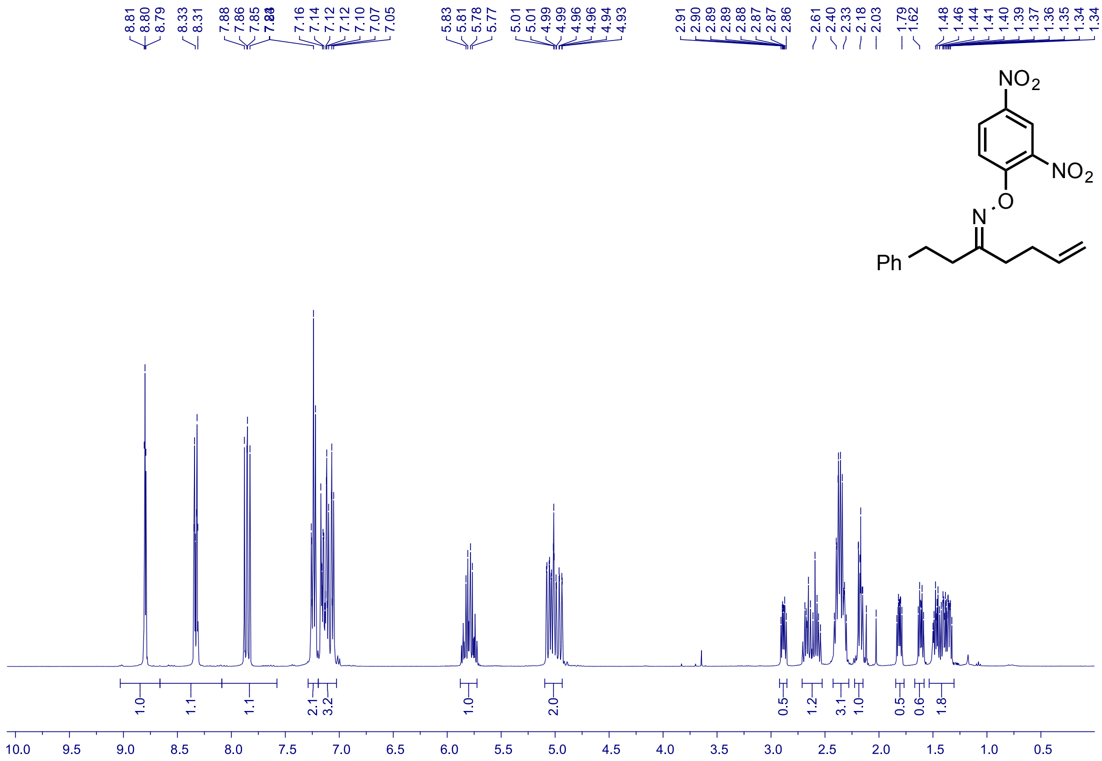

2n: ^{13}C NMR (101 MHz, $CDCl_3$)

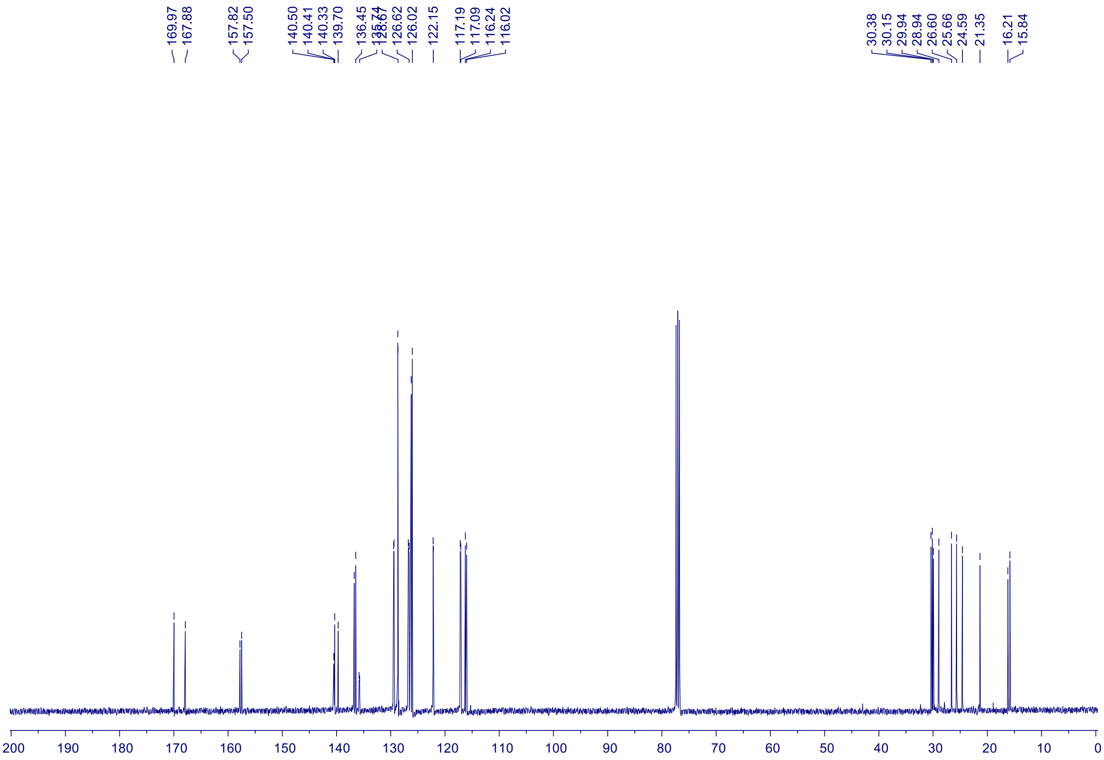

2o: ^{1}H NMR (400 MHz, $CDCl_3$)

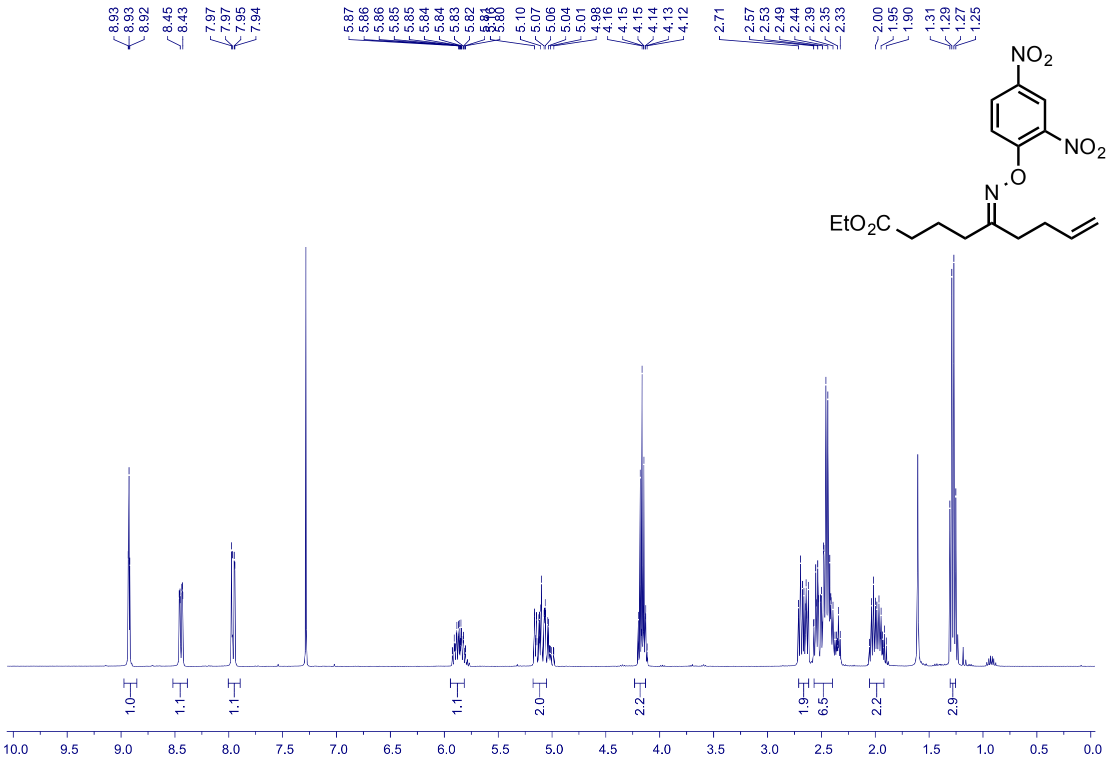

2o: ^{13}C NMR (101 MHz, $CDCl_3$)

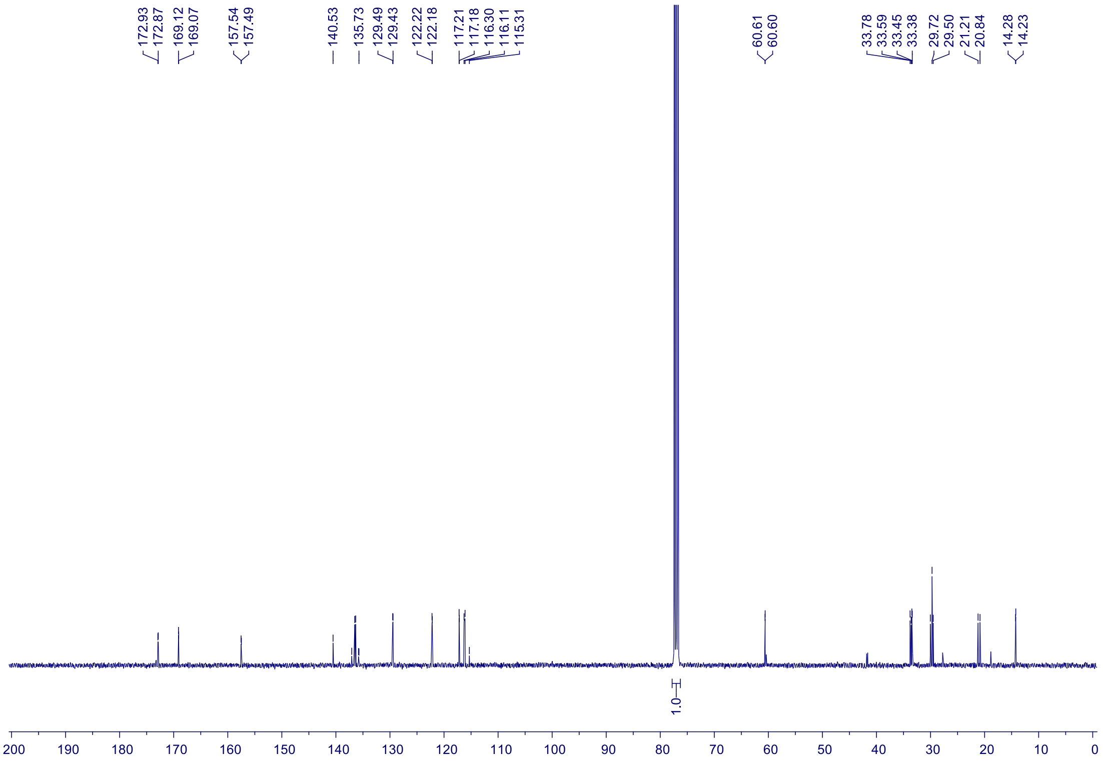

2q: ^{1}H NMR (400 MHz, $CDCl_3$)

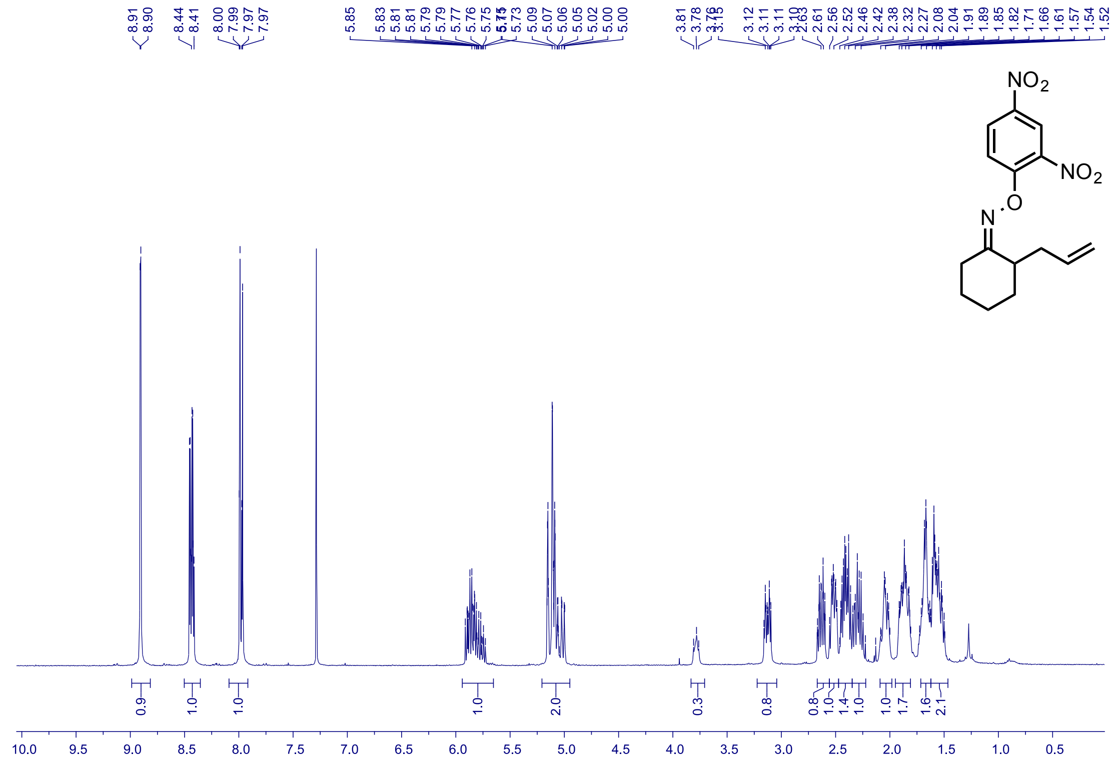

2q: ^{13}C NMR (101 MHz, $CDCl_3$)

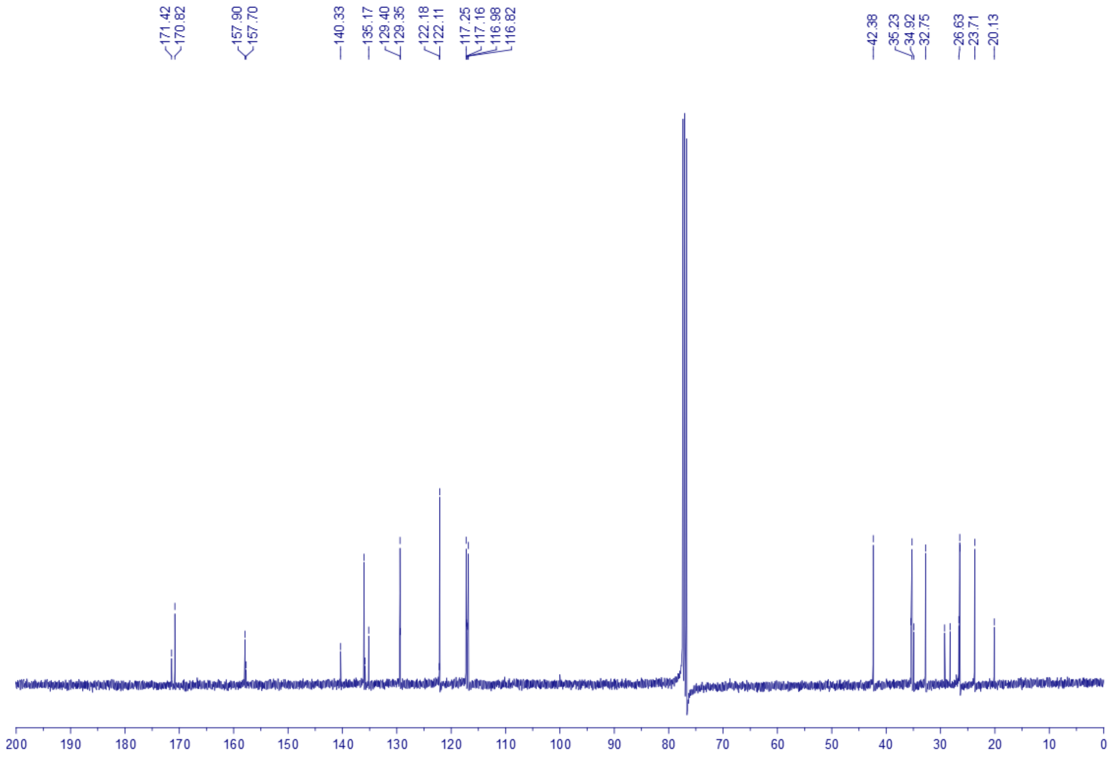

2r: ^{1}H NMR (400 MHz, $CDCl_3$)

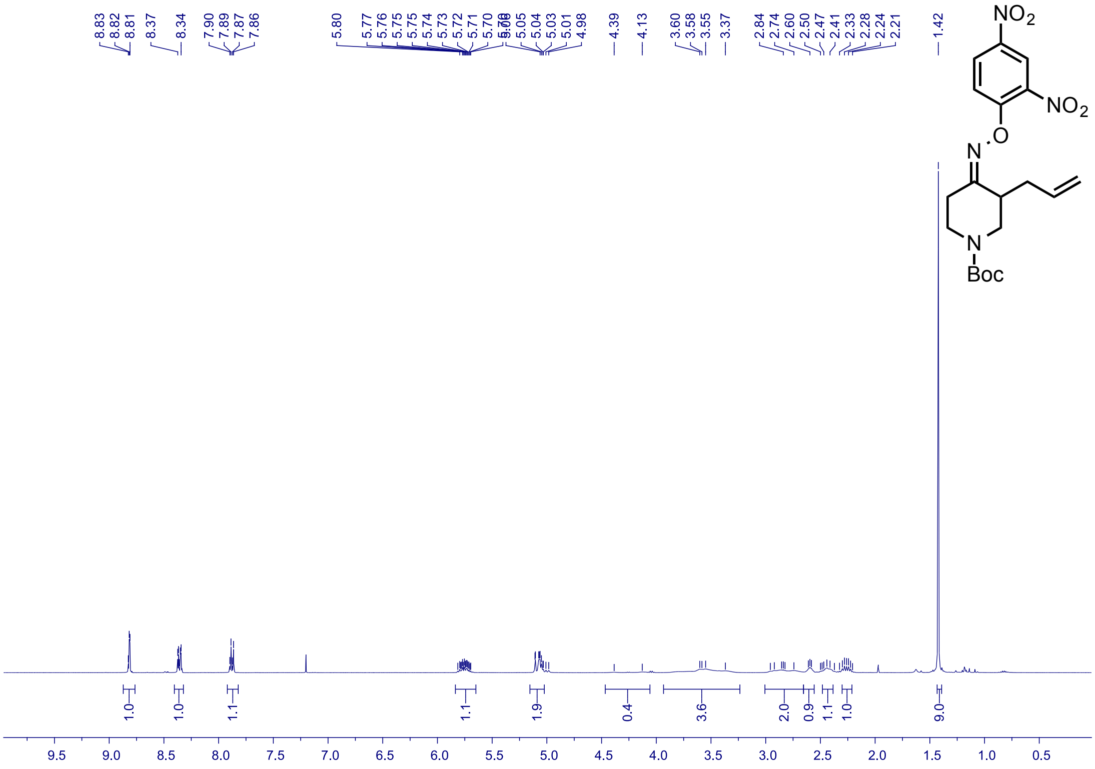

2r: ^{13}C NMR (101 MHz, $CDCl_3$)

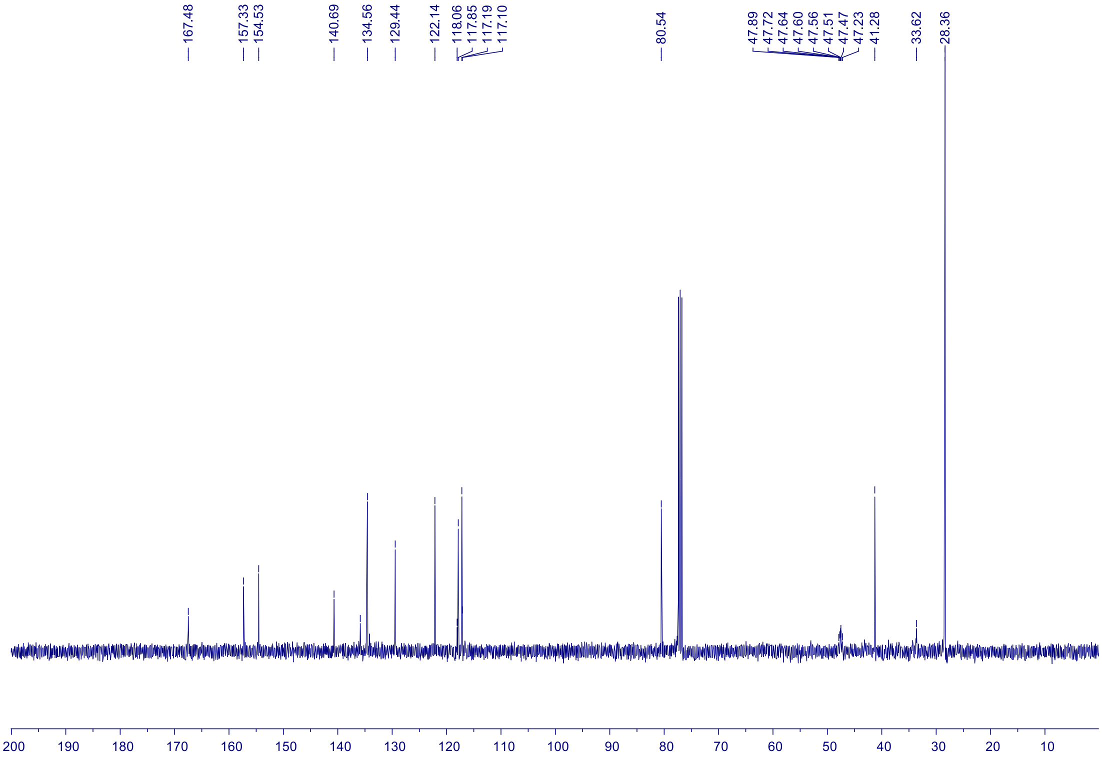

2s: ^{1}H NMR (400 MHz, $CDCl_3$)

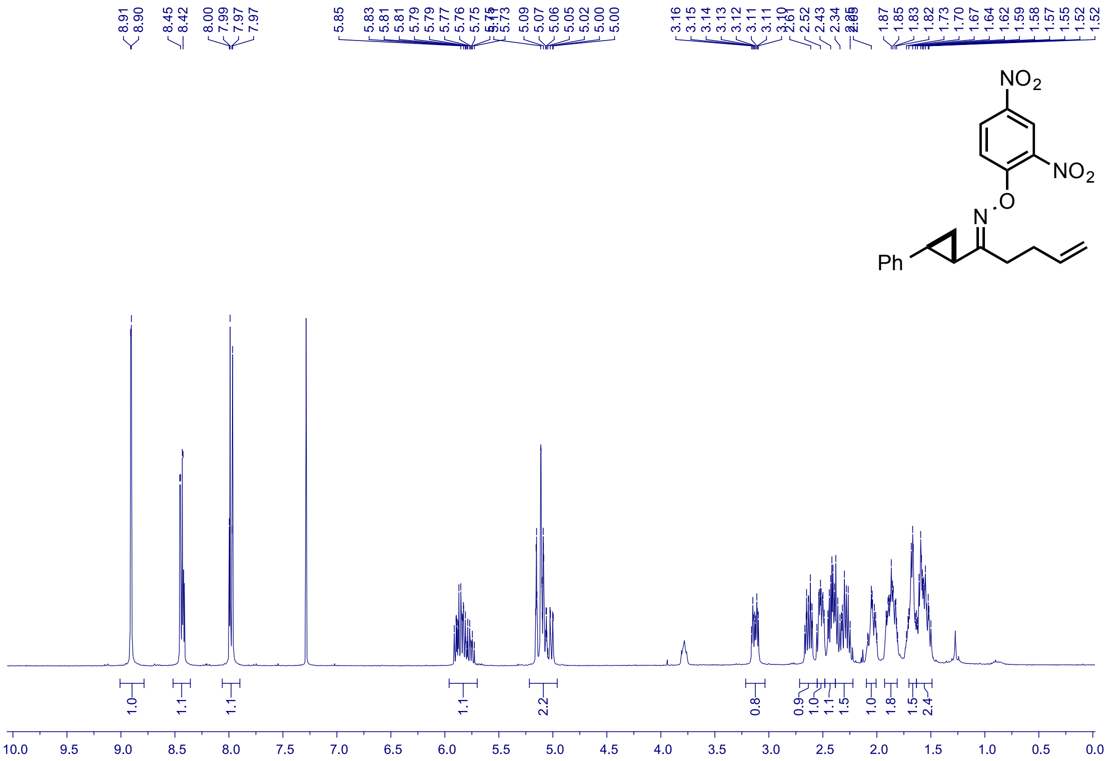

2s: ^{13}C NMR (101 MHz, $CDCl_3$)

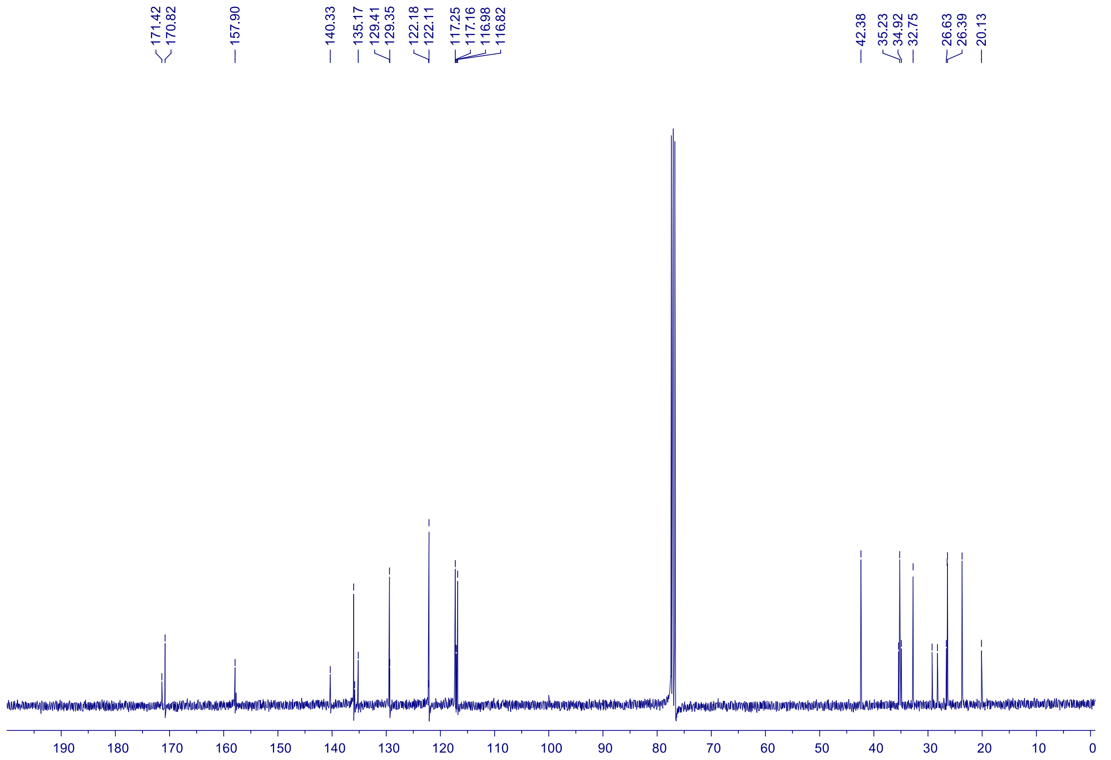

2t: ^{1}H NMR (400 MHz, CDCl$_3$)

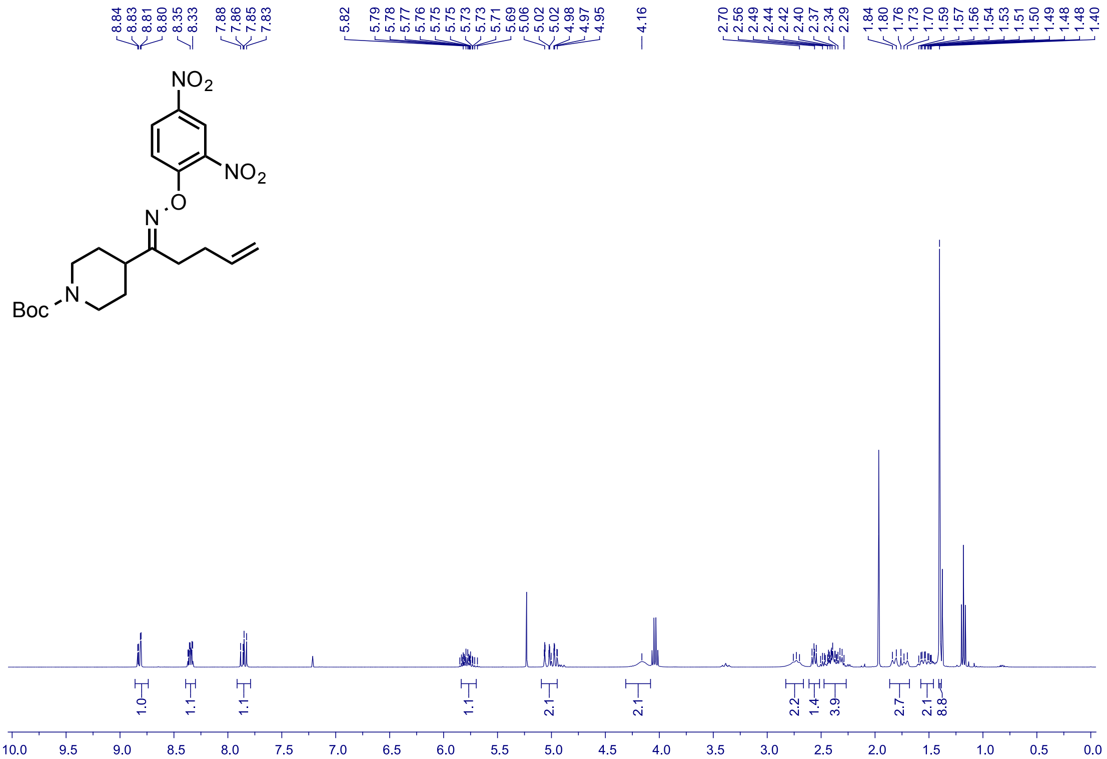

2t: ^{13}C NMR (101 MHz, CDCl$_3$)

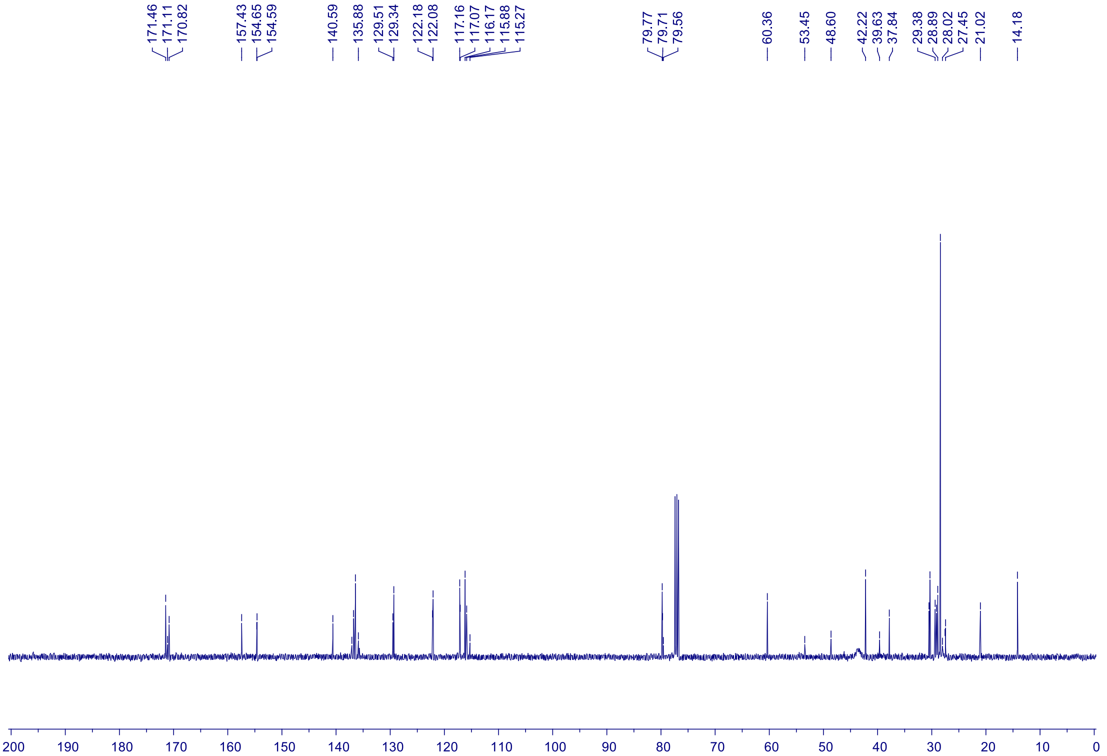

2u: ^{1}H NMR (400 MHz, CDCl$_3$)

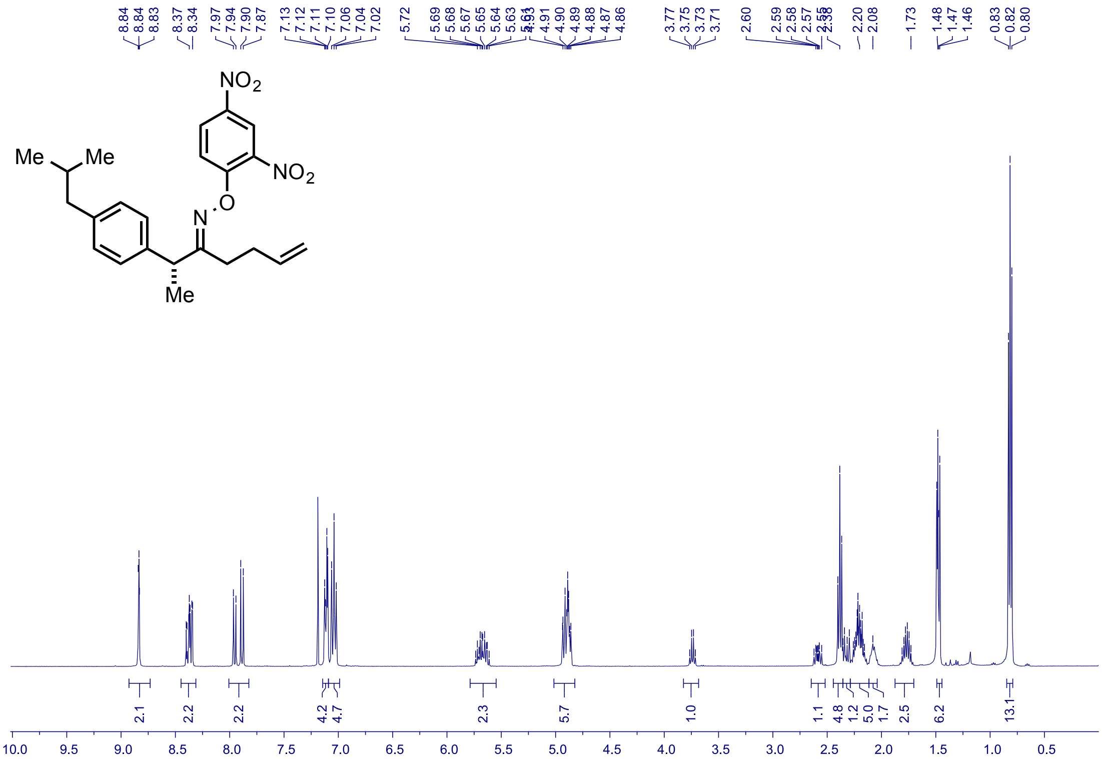

2u: ^{13}C NMR (101 MHz, CDCl$_3$)

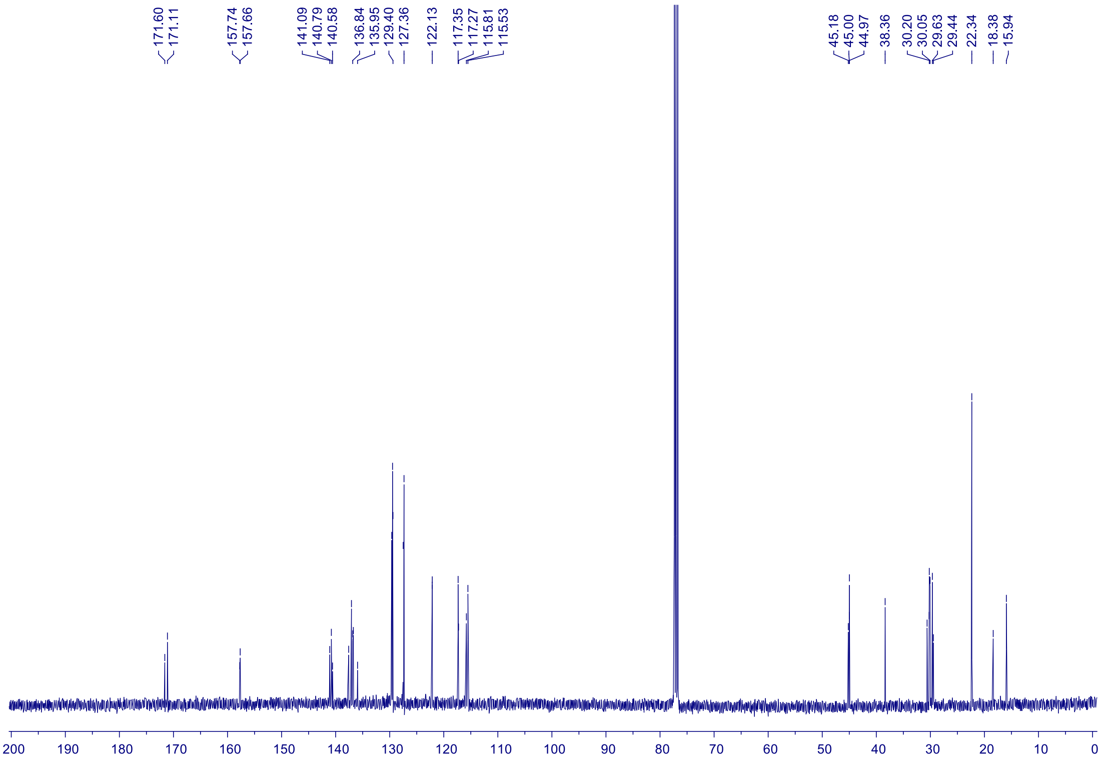

2v: ^{1}H NMR (400 MHz, CDCl$_3$)

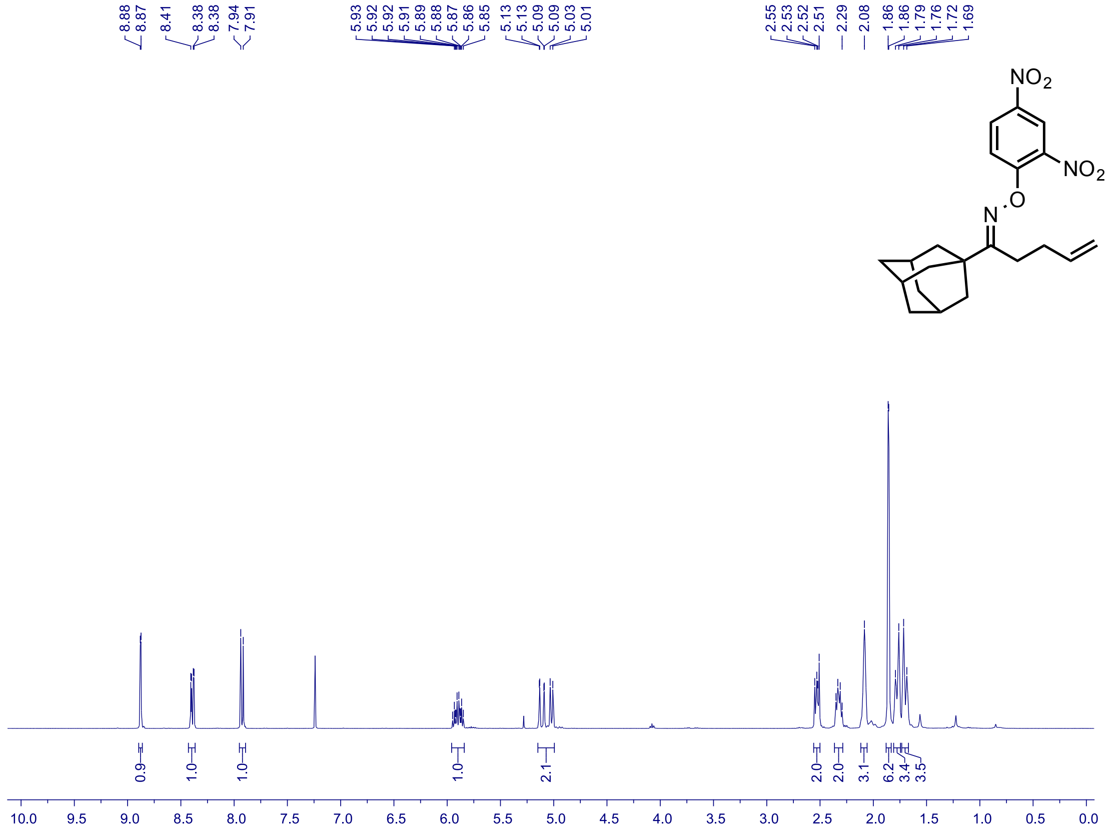

2v: ^{13}C NMR (101 MHz, CDCl$_3$)

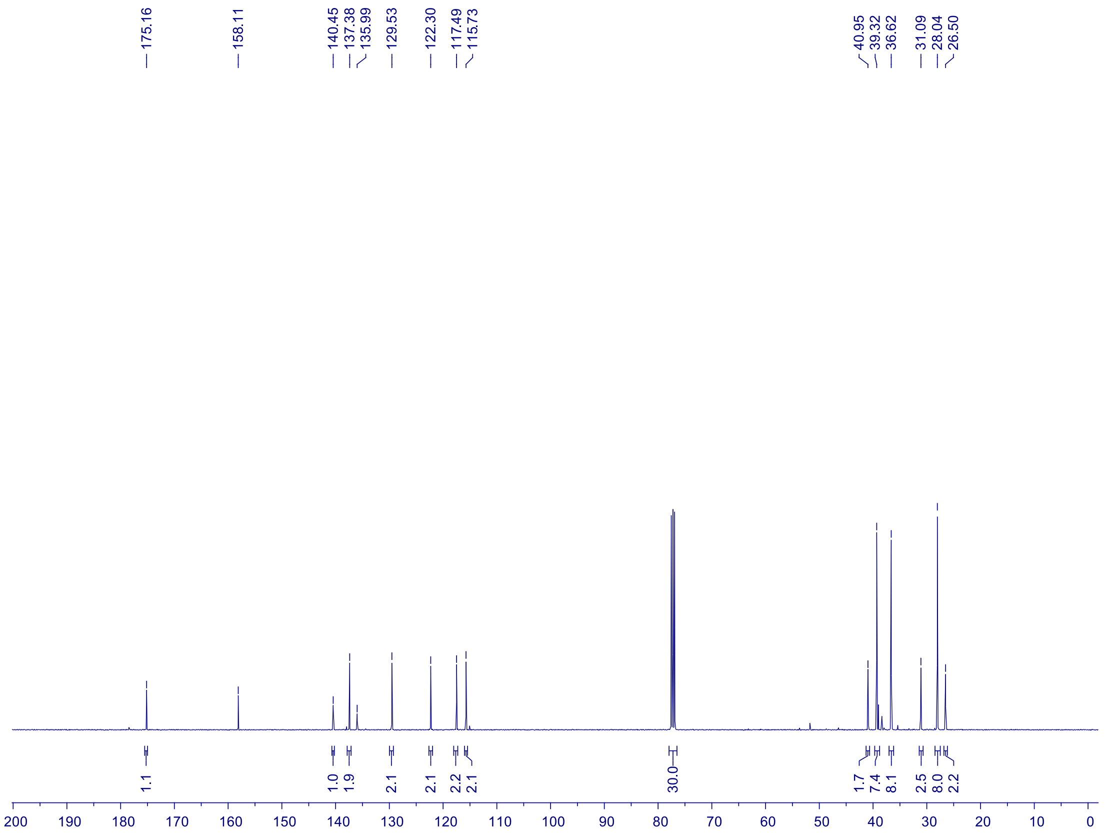

2w: ^{1}H NMR (400 MHz, $CDCl_3$)

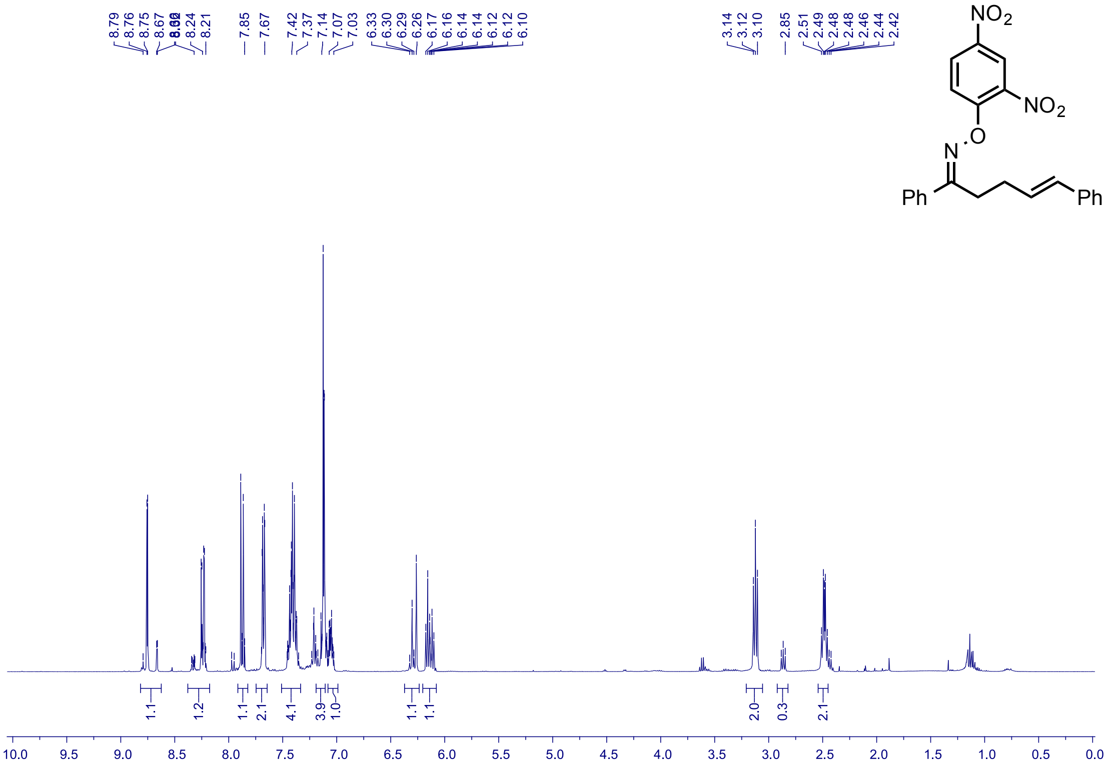

2w: ^{13}C NMR (101 MHz, $CDCl_3$)

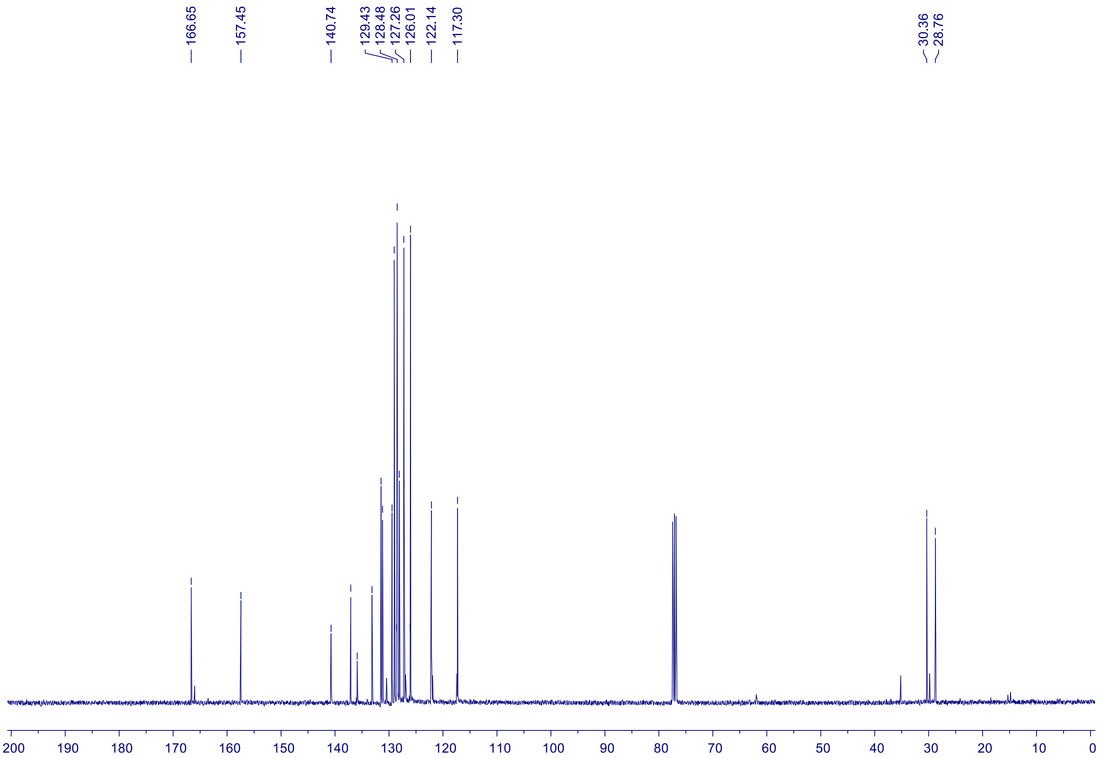

2y: ^{1}H NMR (400 MHz, $CDCl_3$)

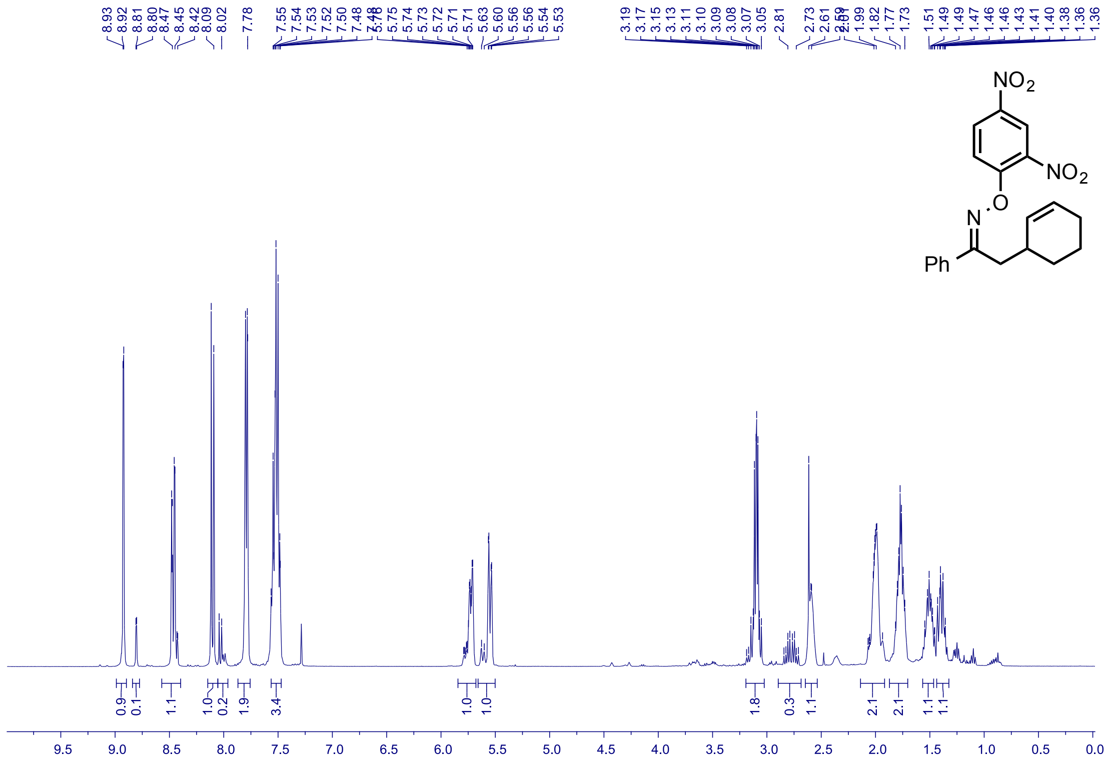

2y: ^{13}C NMR (101 MHz, $CDCl_3$)

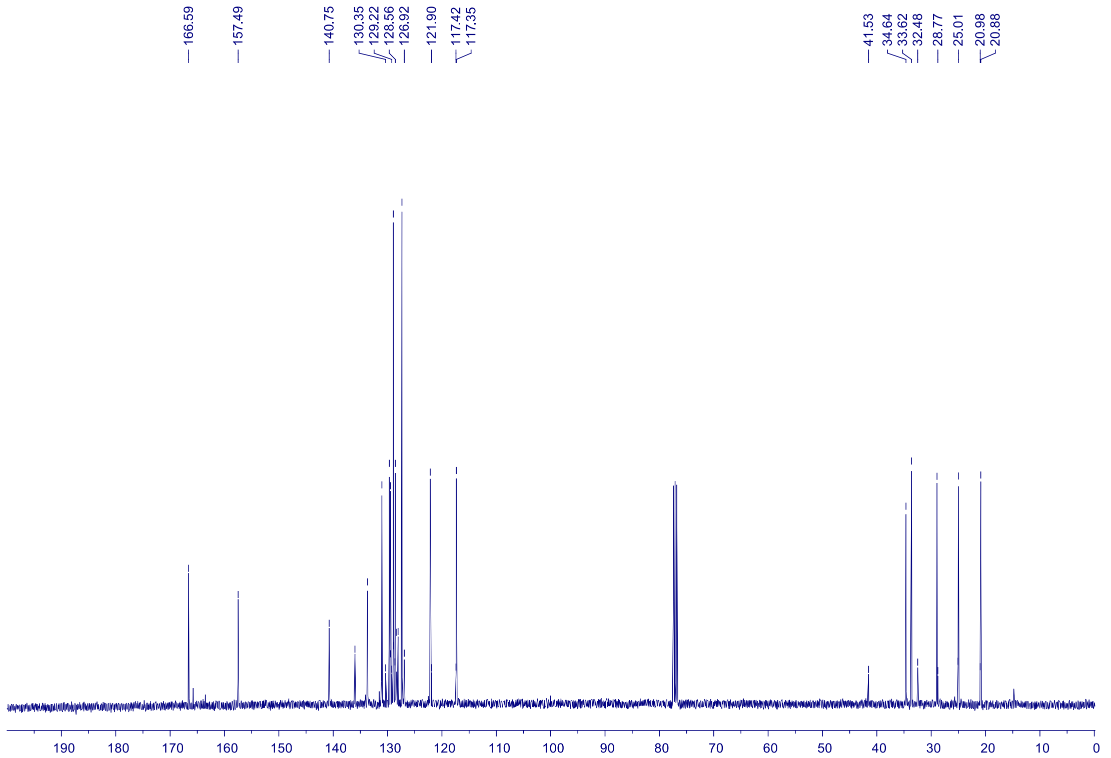

2z: ^{1}H NMR (400 MHz, $CDCl_3$)

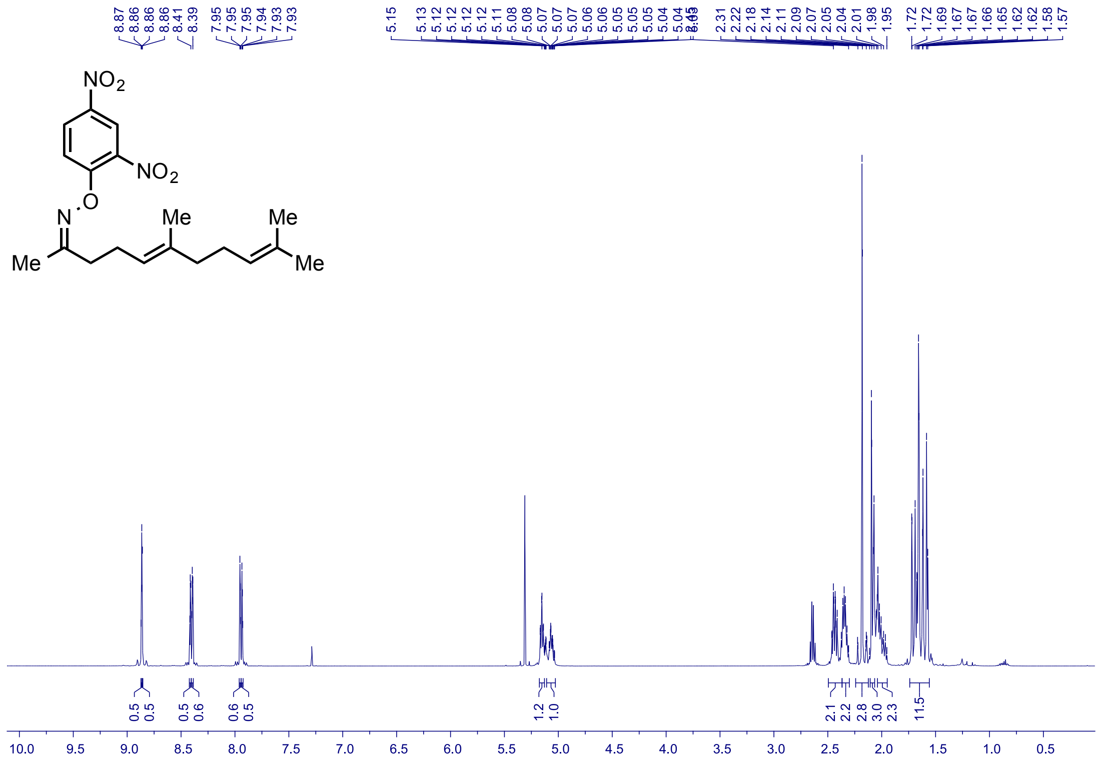

2z: ^{13}C NMR (101 MHz, $CDCl_3$)

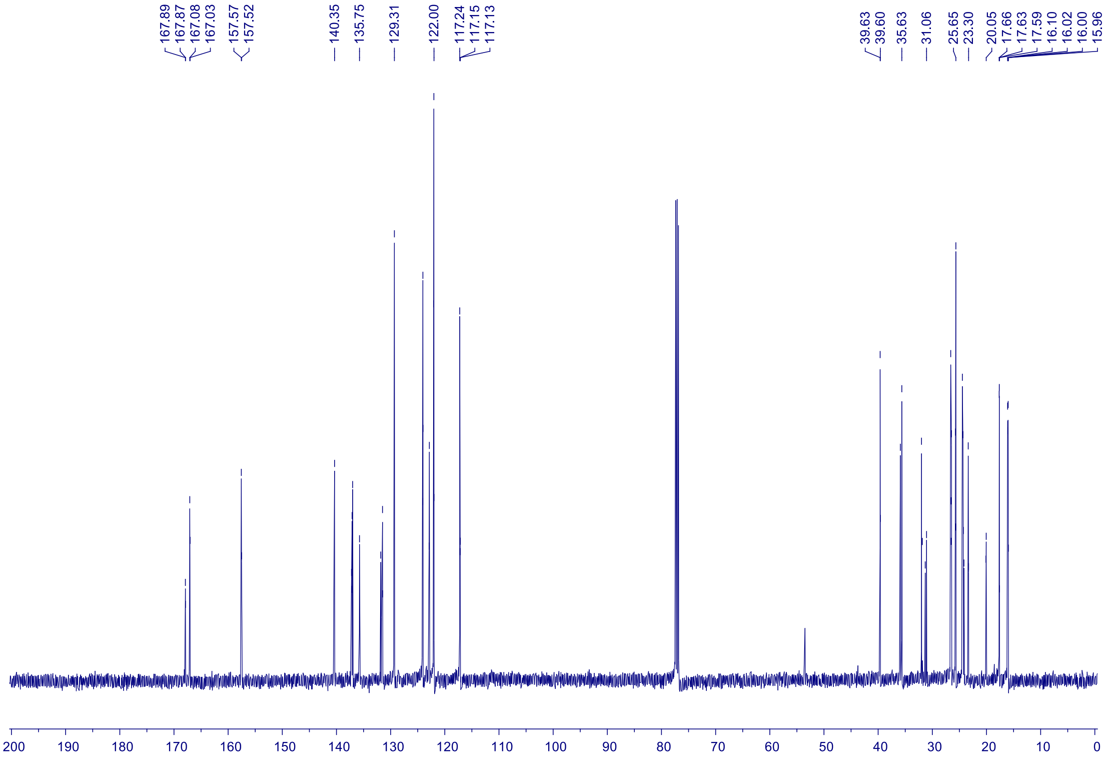

3c: ^{1}H NMR (400 MHz, $CDCl_3$)

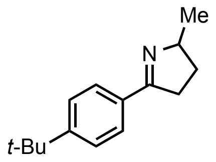

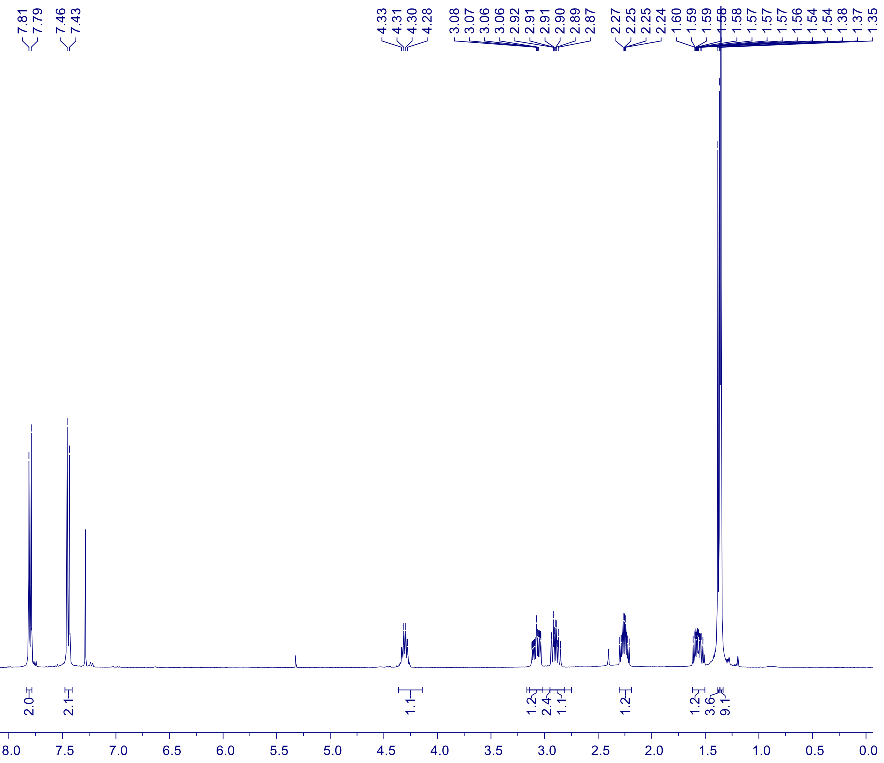

3c: ^{13}C NMR (101 MHz, $CDCl_3$)

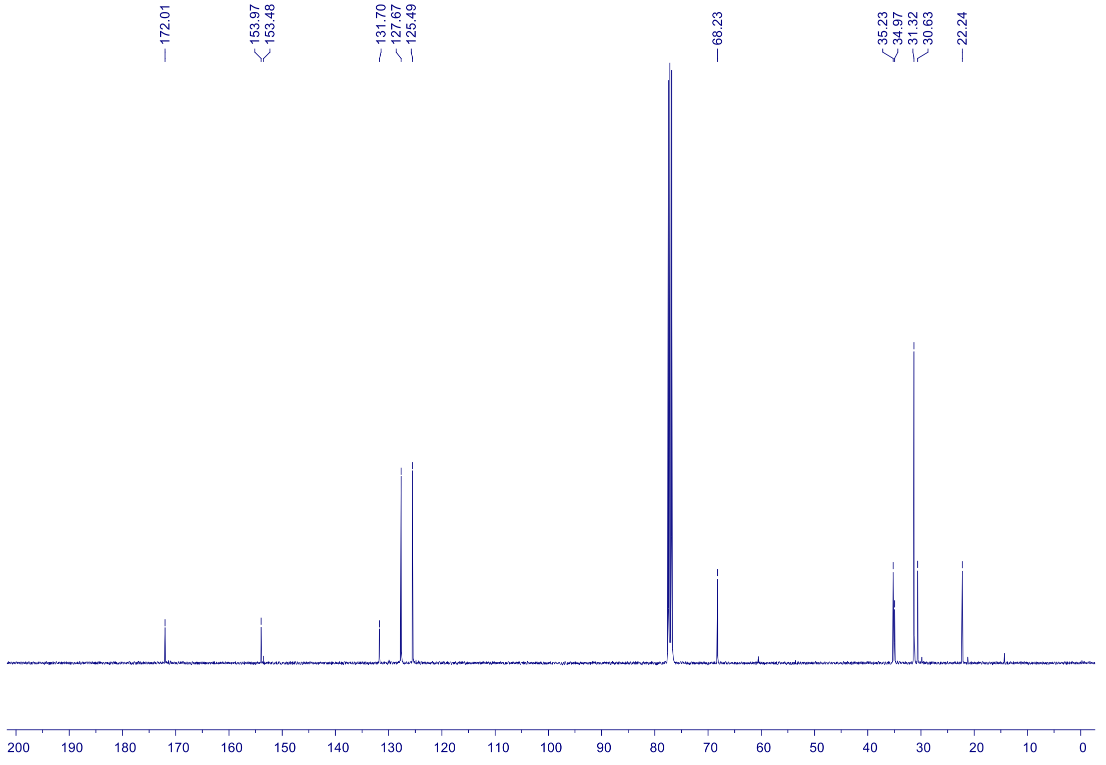

3g: ^{1}H NMR (400 MHz, $CDCl_3$)

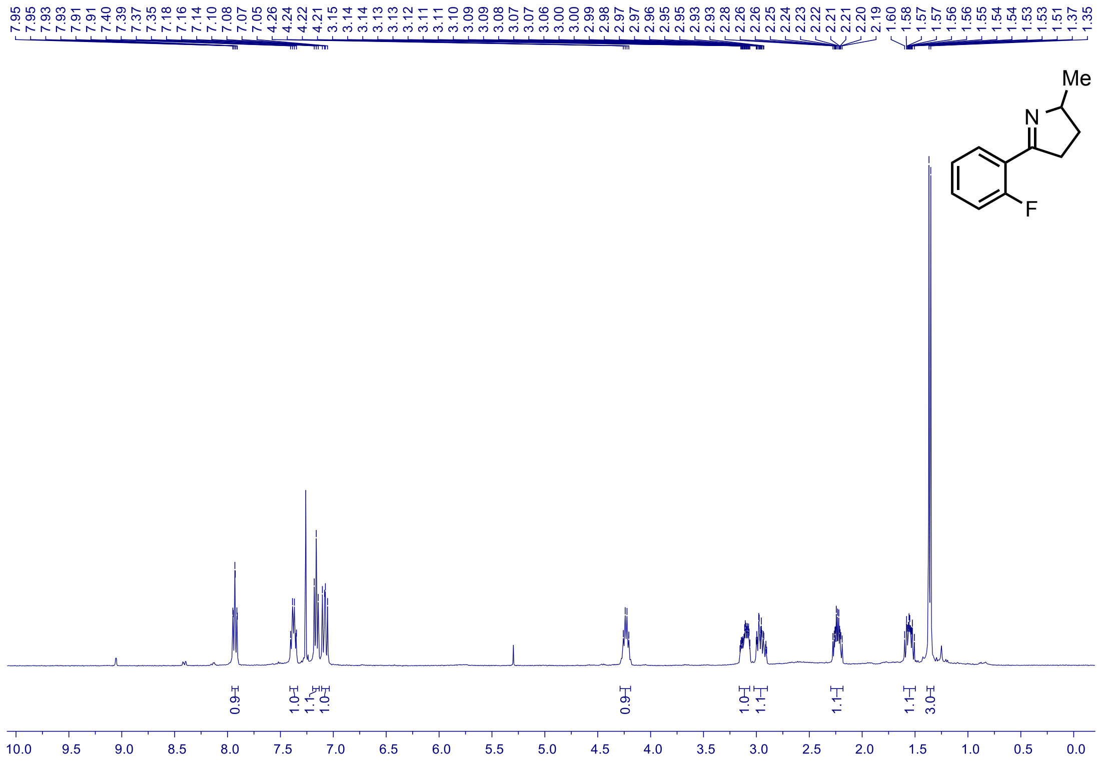

3g: ^{13}C NMR (101 MHz, $CDCl_3$)

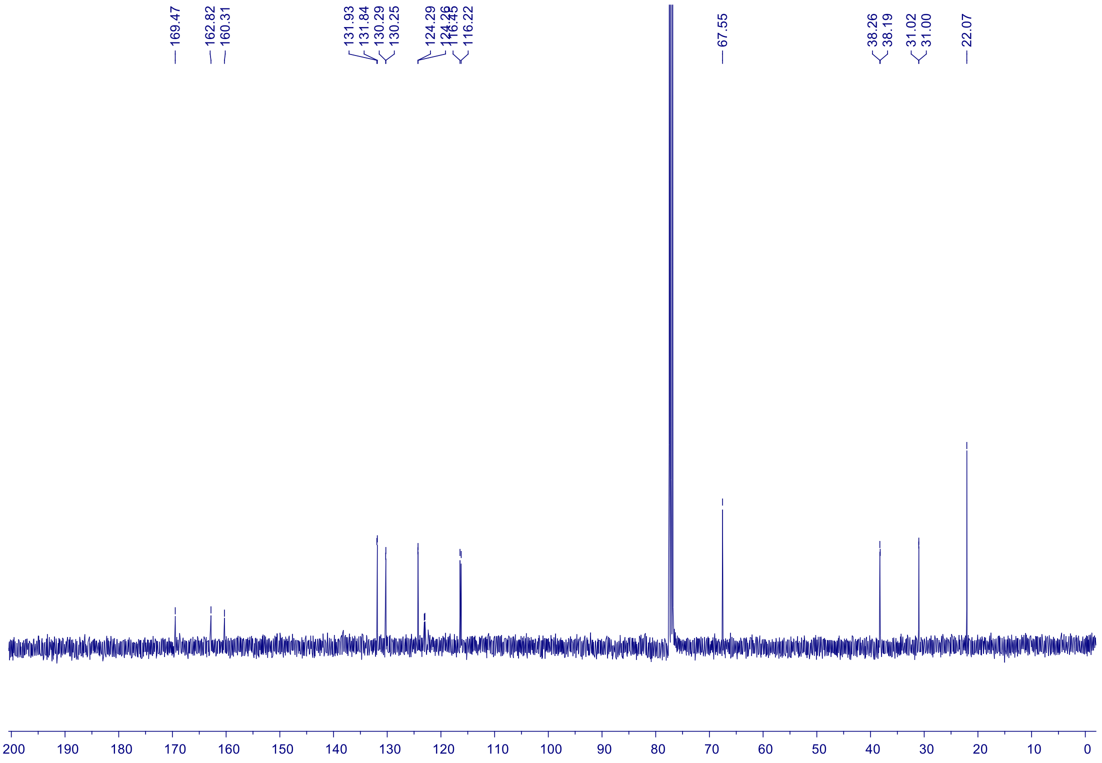

3h: ^{1}H NMR (400 MHz, $CDCl_3$)

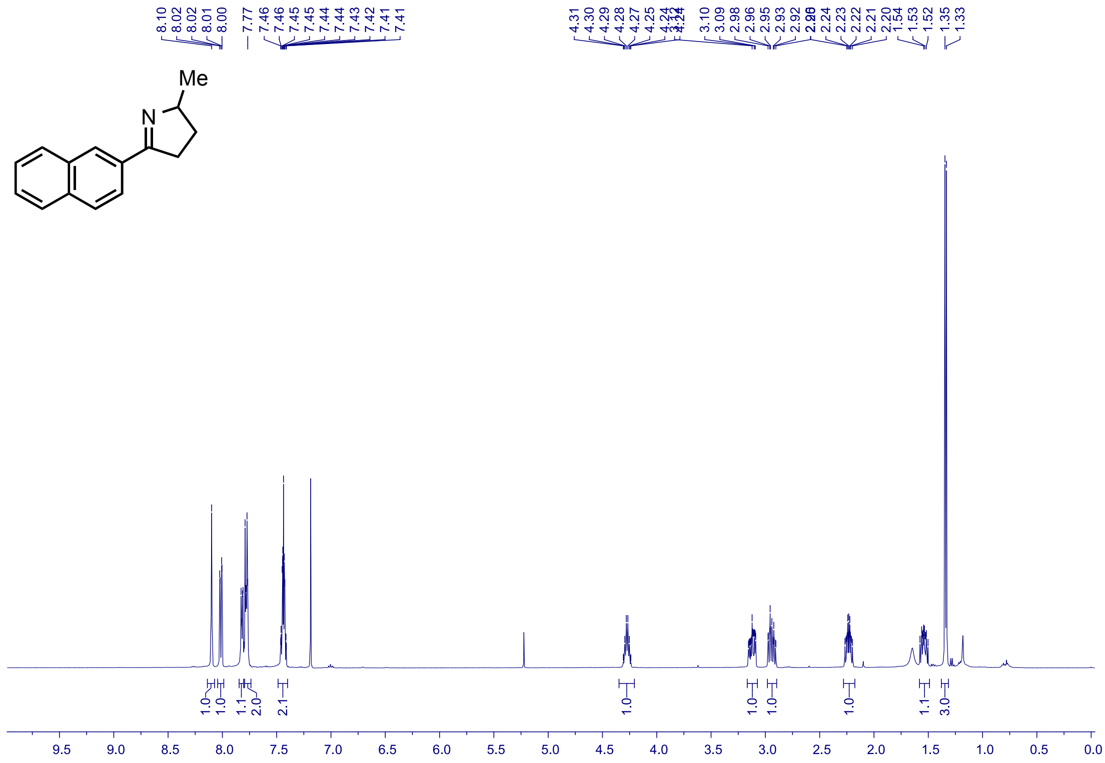

3h: ^{13}C NMR (101 MHz, $CDCl_3$)

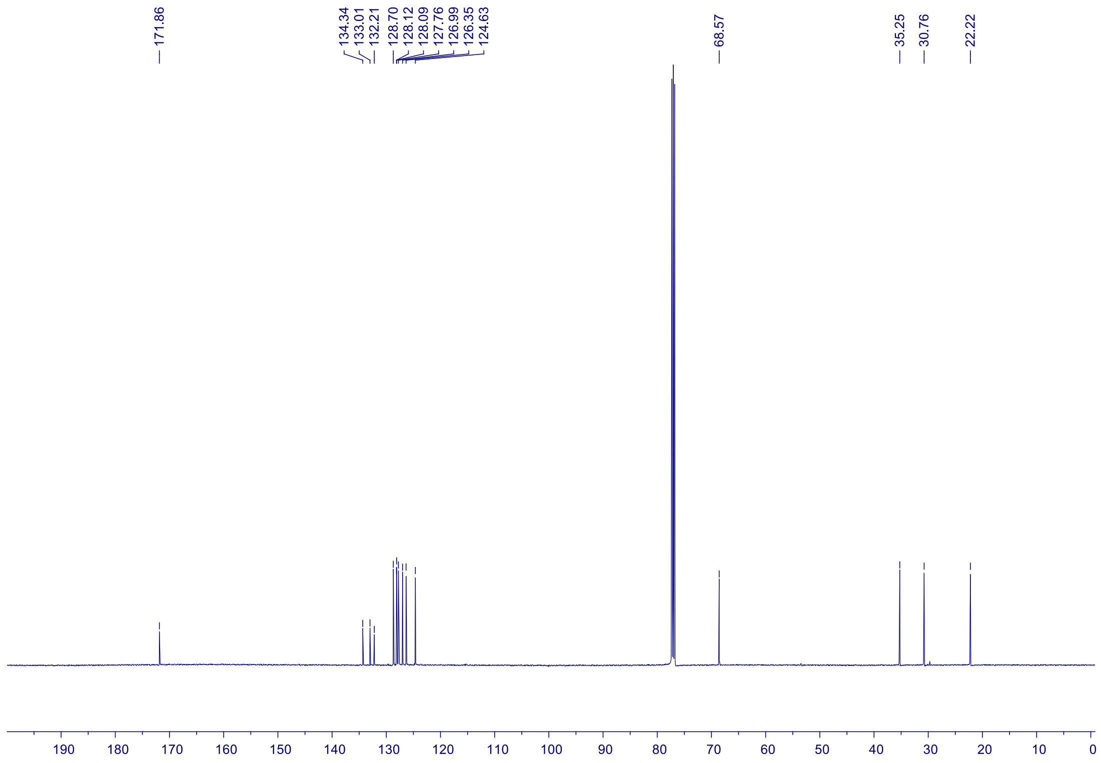

3i: ^{1}H NMR (400 MHz, $CDCl_3$)

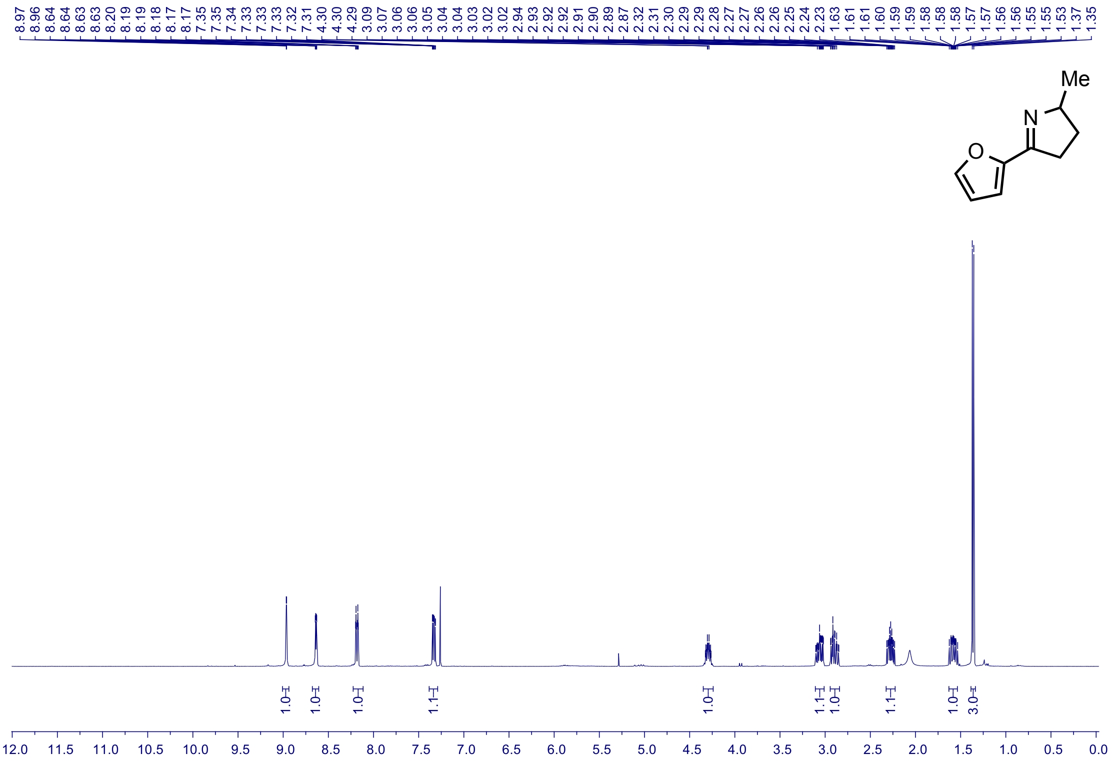

3i: ^{13}C NMR (101 MHz, $CDCl_3$)

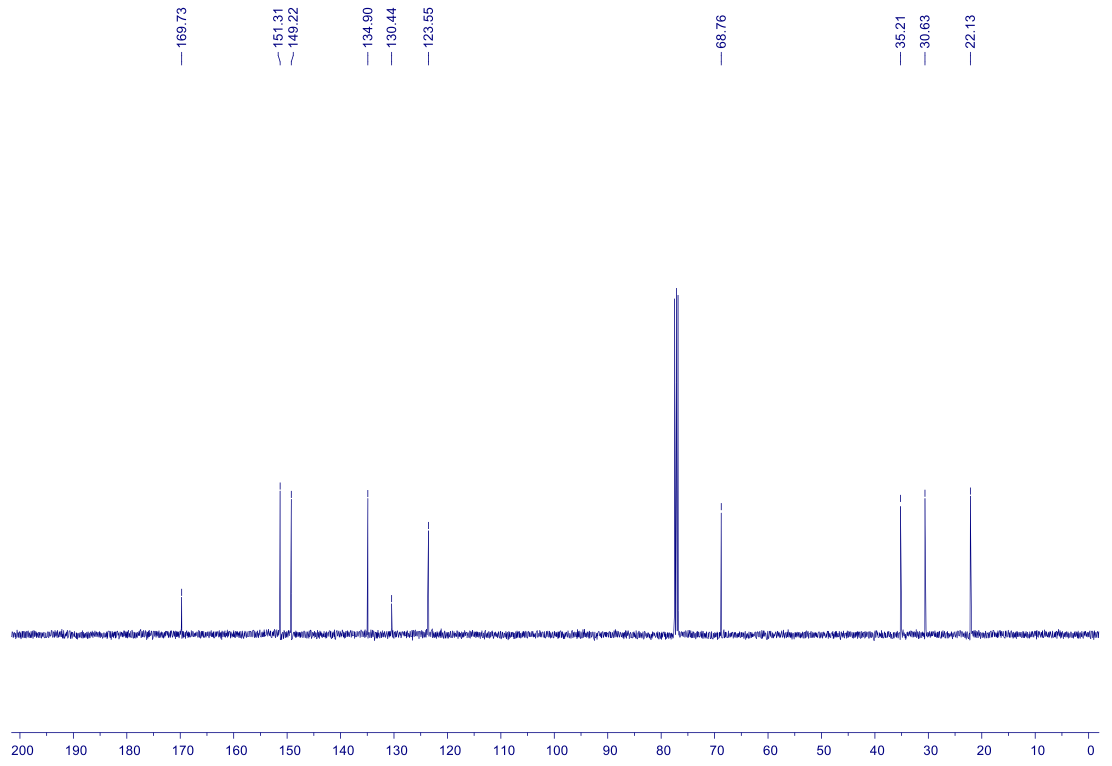

3j: ^{1}H NMR (400 MHz, $CDCl_3$)

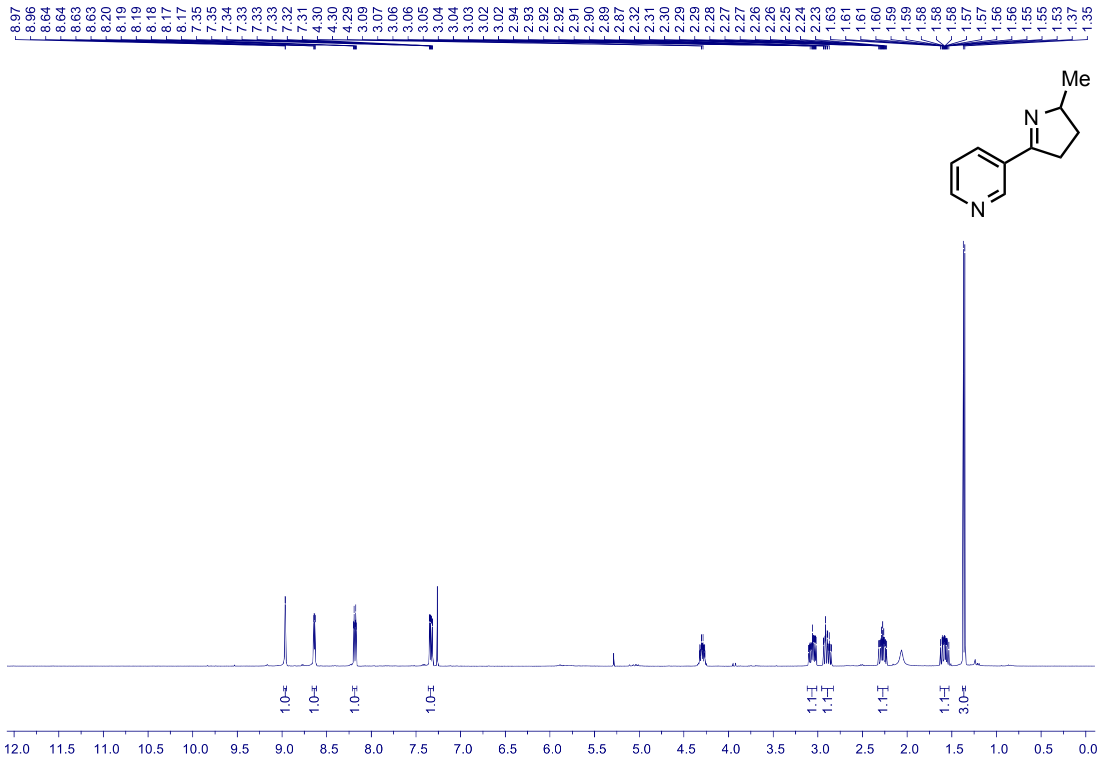

3j: ^{13}C NMR (101 MHz, $CDCl_3$)

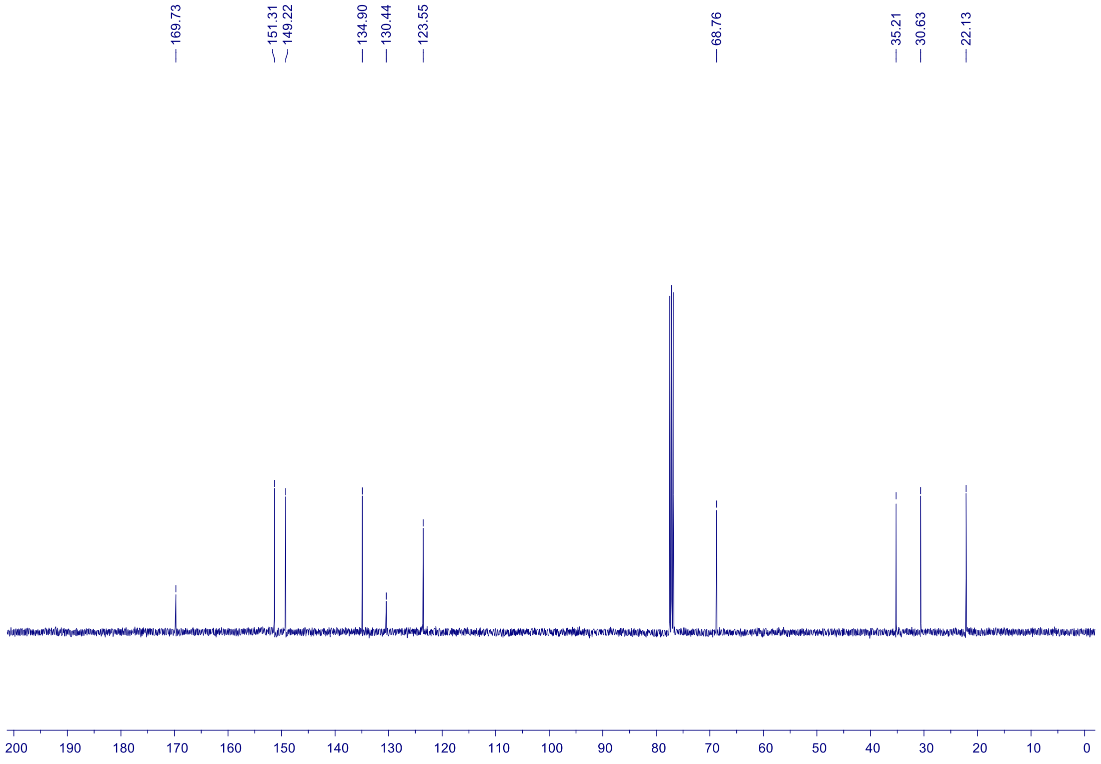

3l: ^{1}H NMR (400 MHz, $CDCl_3$)

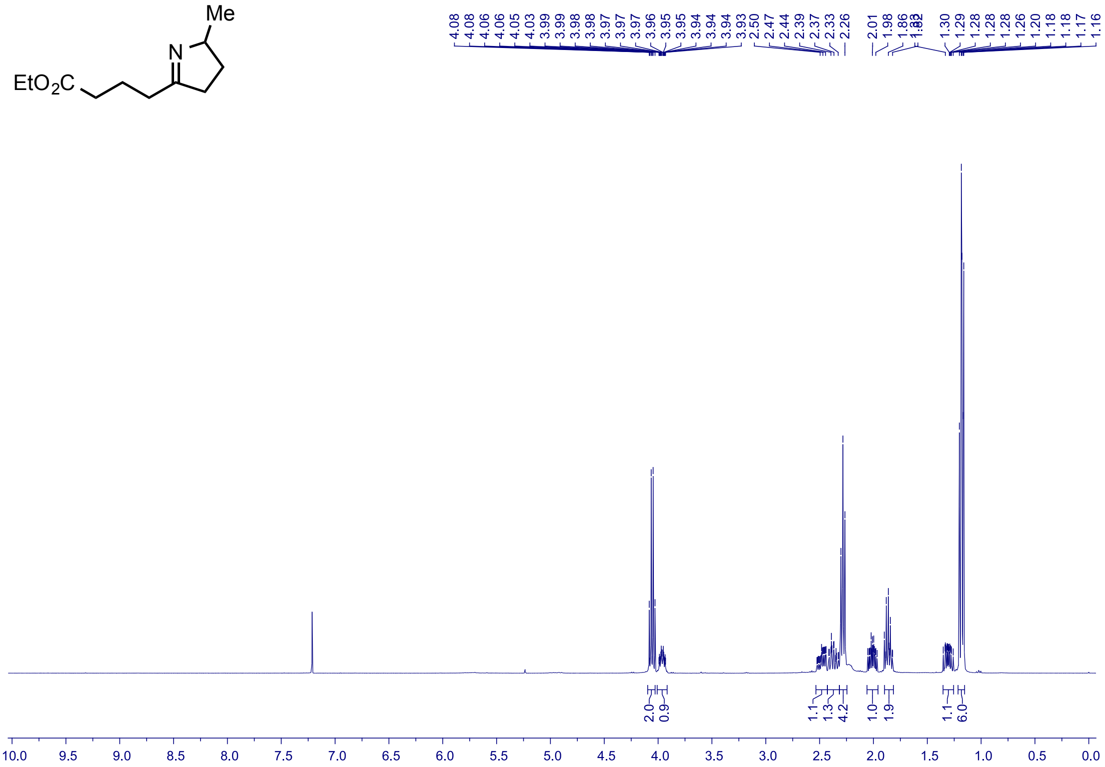

3l: ^{13}C NMR (101 MHz, $CDCl_3$)

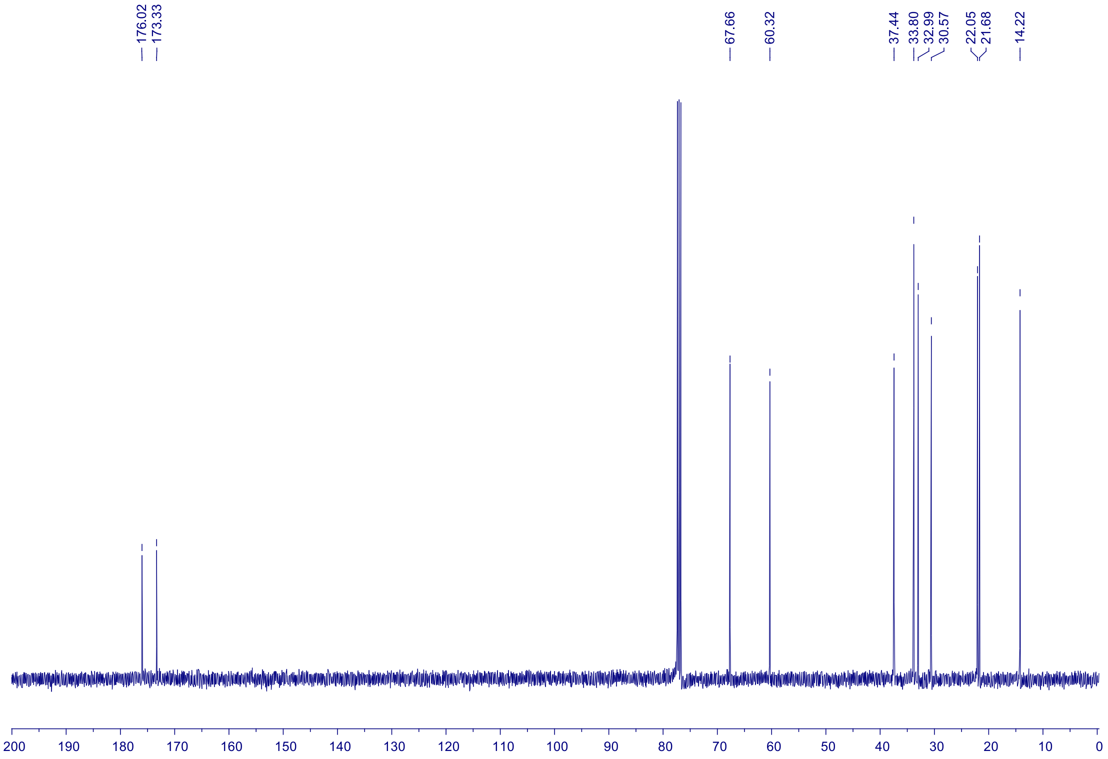

3m: ^{1}H NMR (400 MHz, $CDCl_3$)

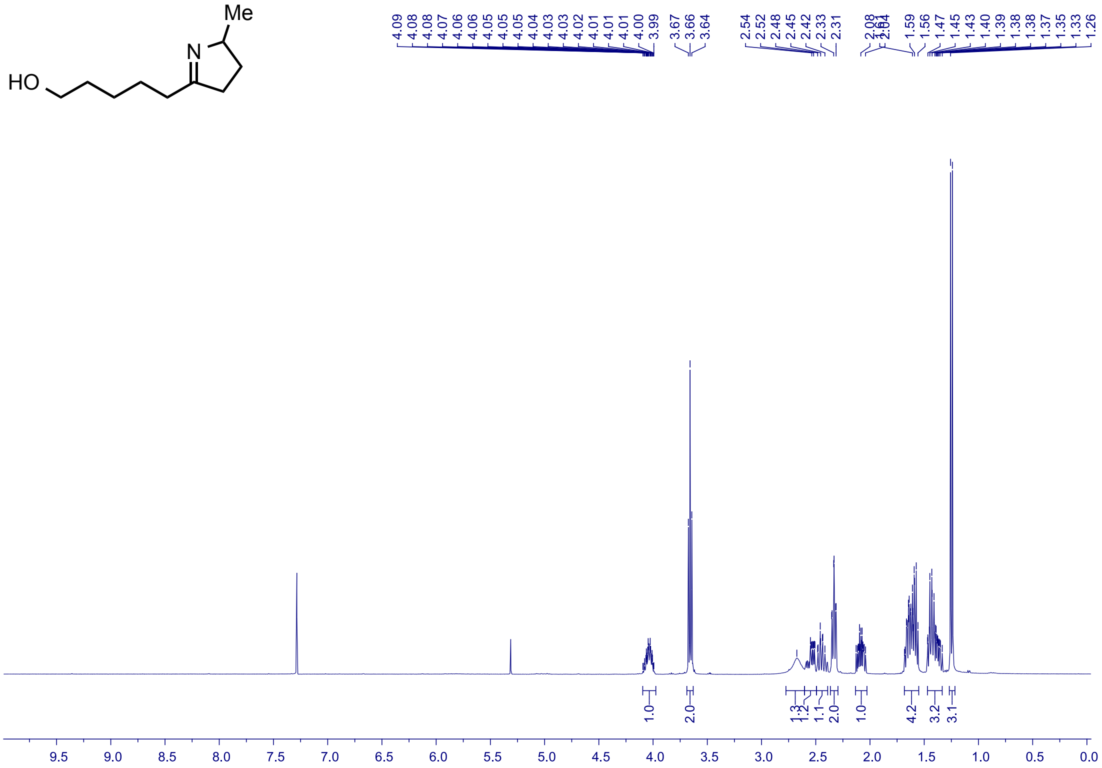

3m: ^{13}C NMR (101 MHz, $CDCl_3$)

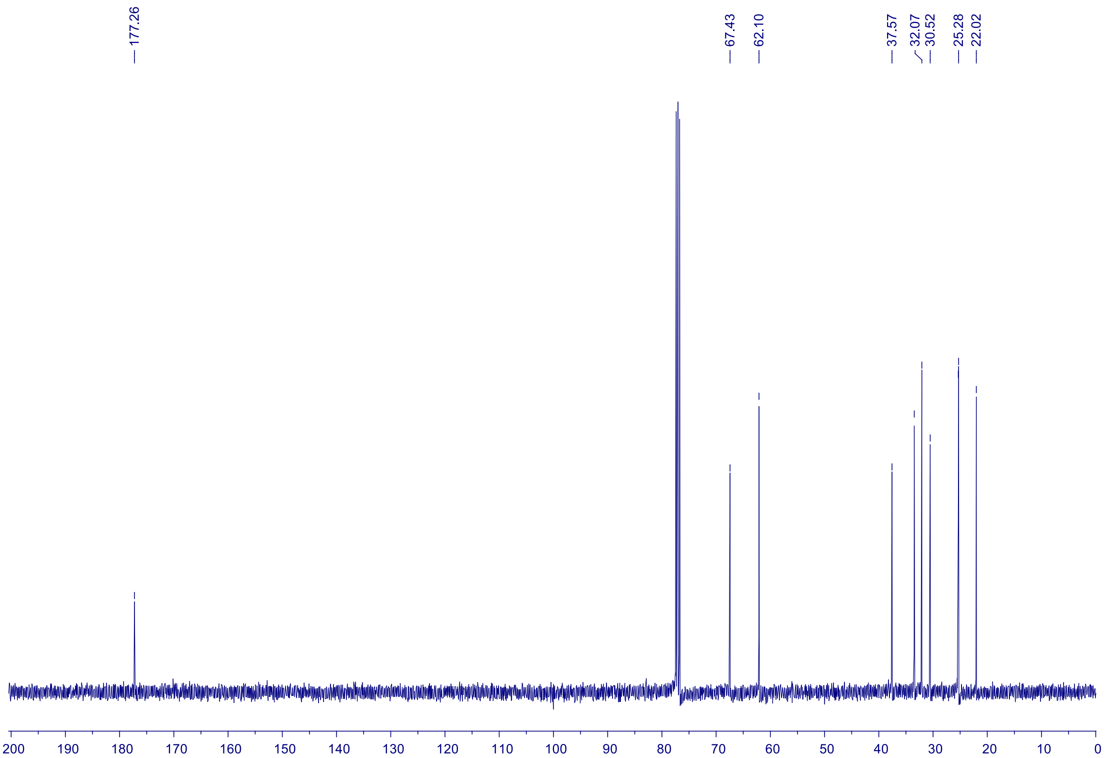

3n: ^{1}H NMR (400 MHz, $CDCl_3$)

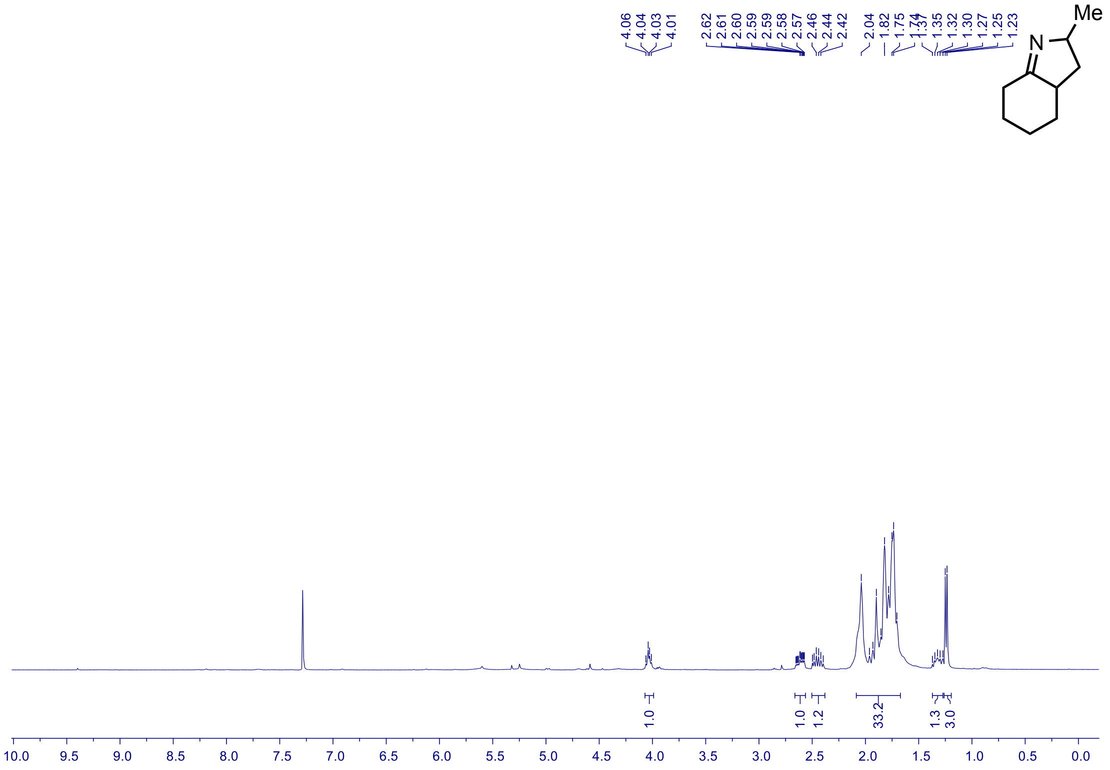

3n: ^{13}C NMR (101 MHz, $CDCl_3$)

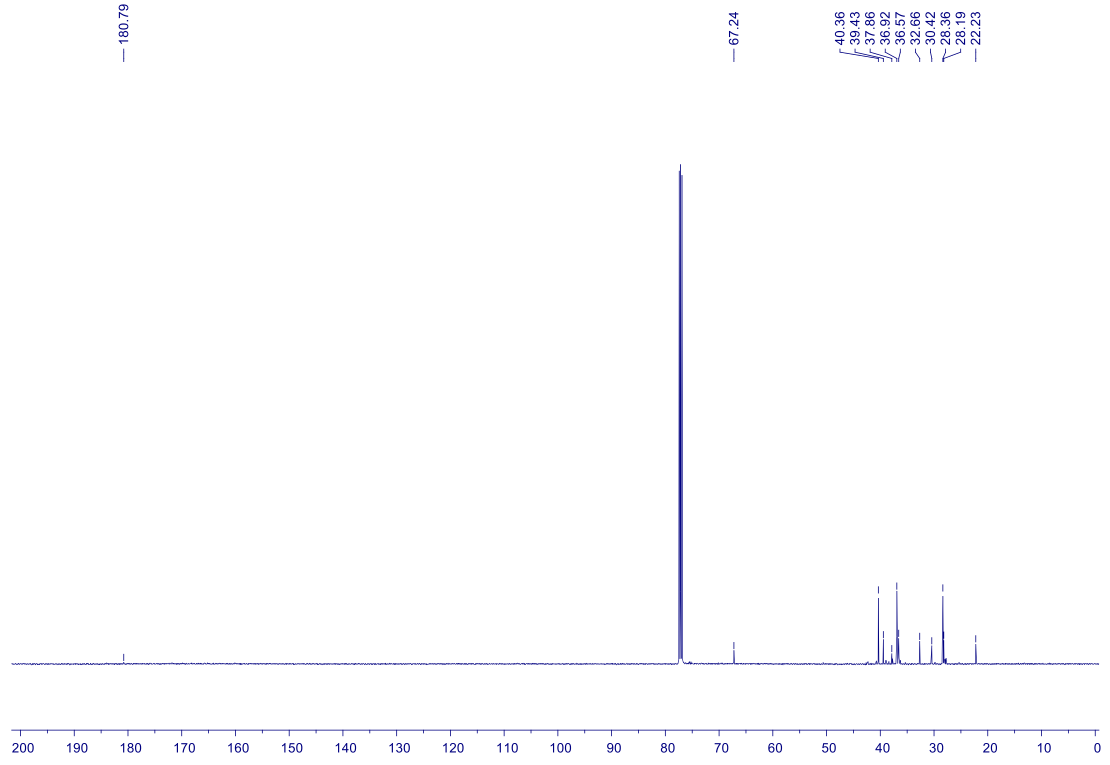

3p: ^{1}H NMR (400 MHz, $CDCl_3$)

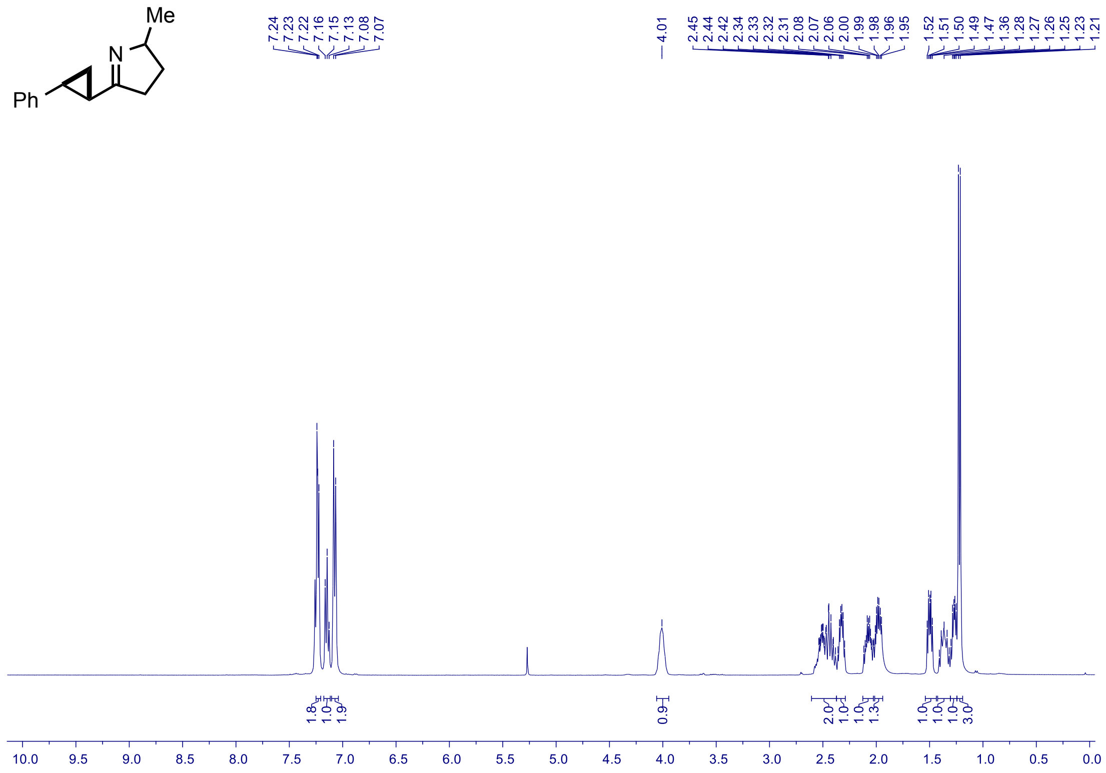

3p: ^{13}C NMR (101 MHz, $CDCl_3$)

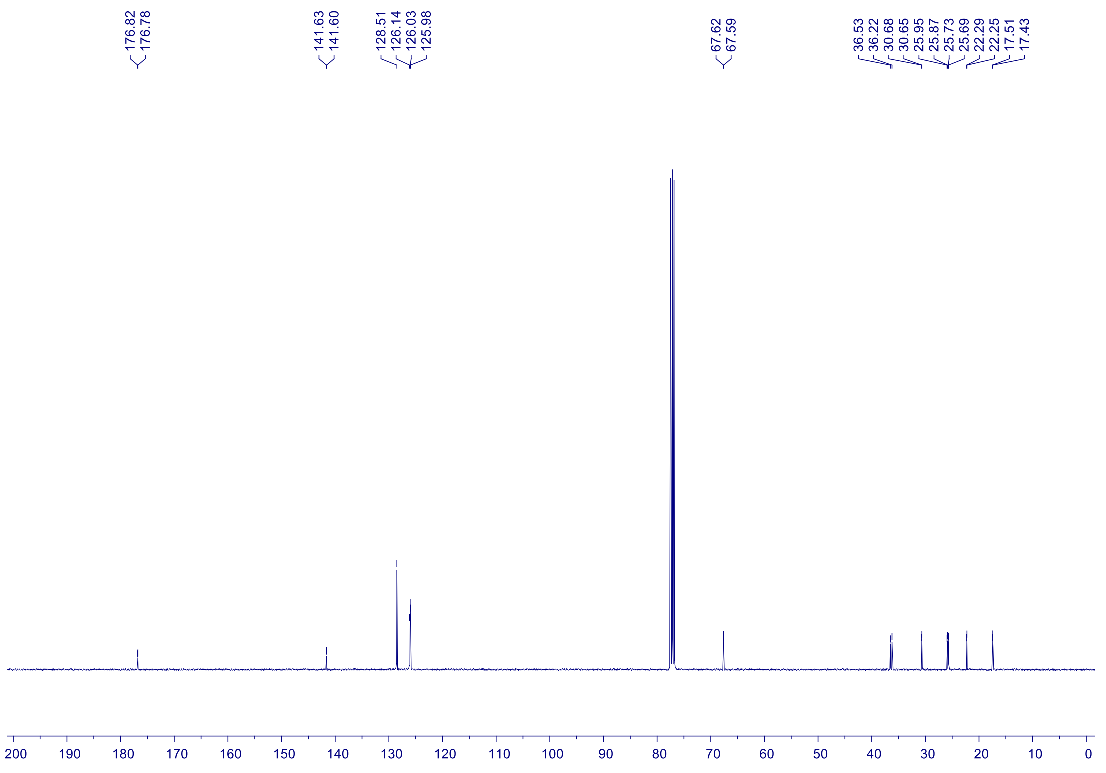

3q: ^{1}H NMR (400 MHz, $CDCl_3$)

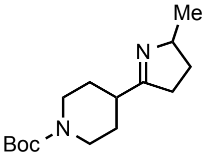

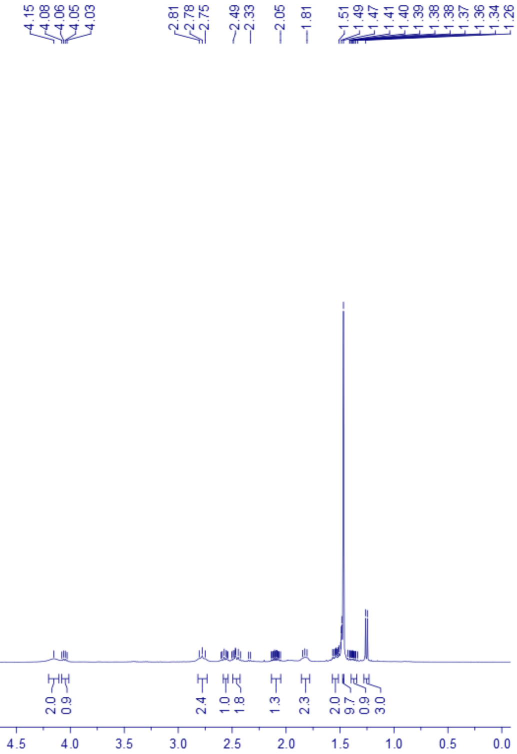

3q: ^{13}C NMR (101 MHz, $CDCl_3$)

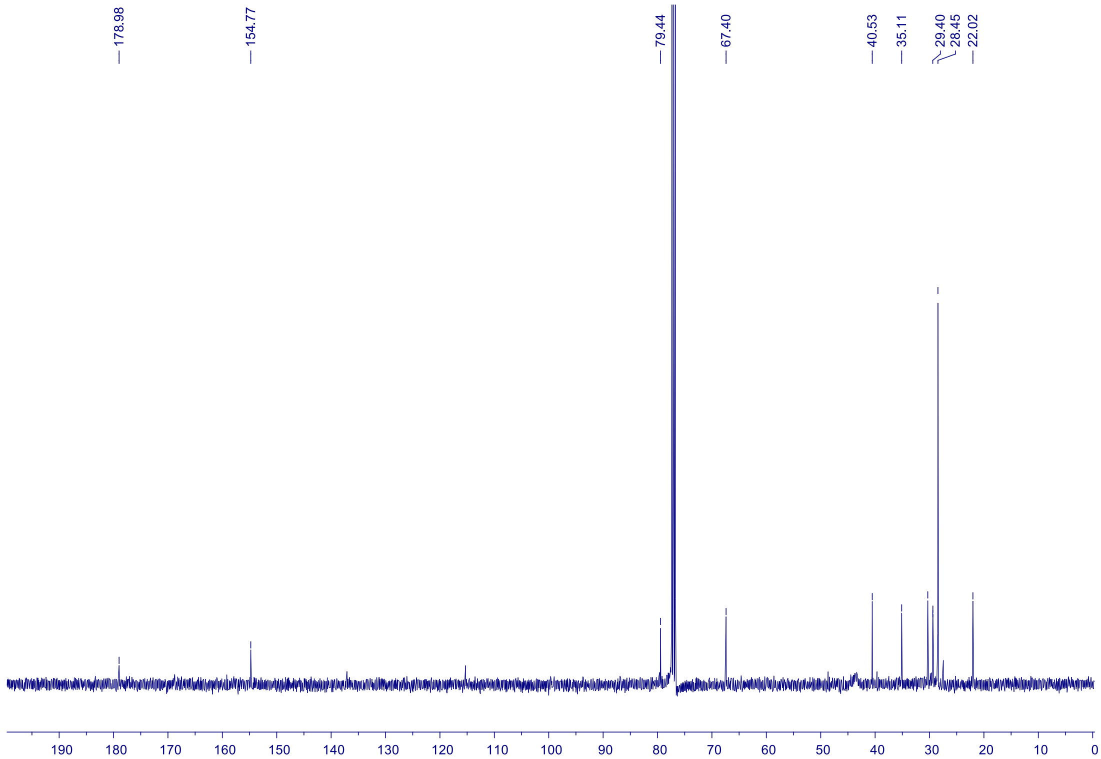

3r: ^{1}H NMR (400 MHz, $CDCl_3$)

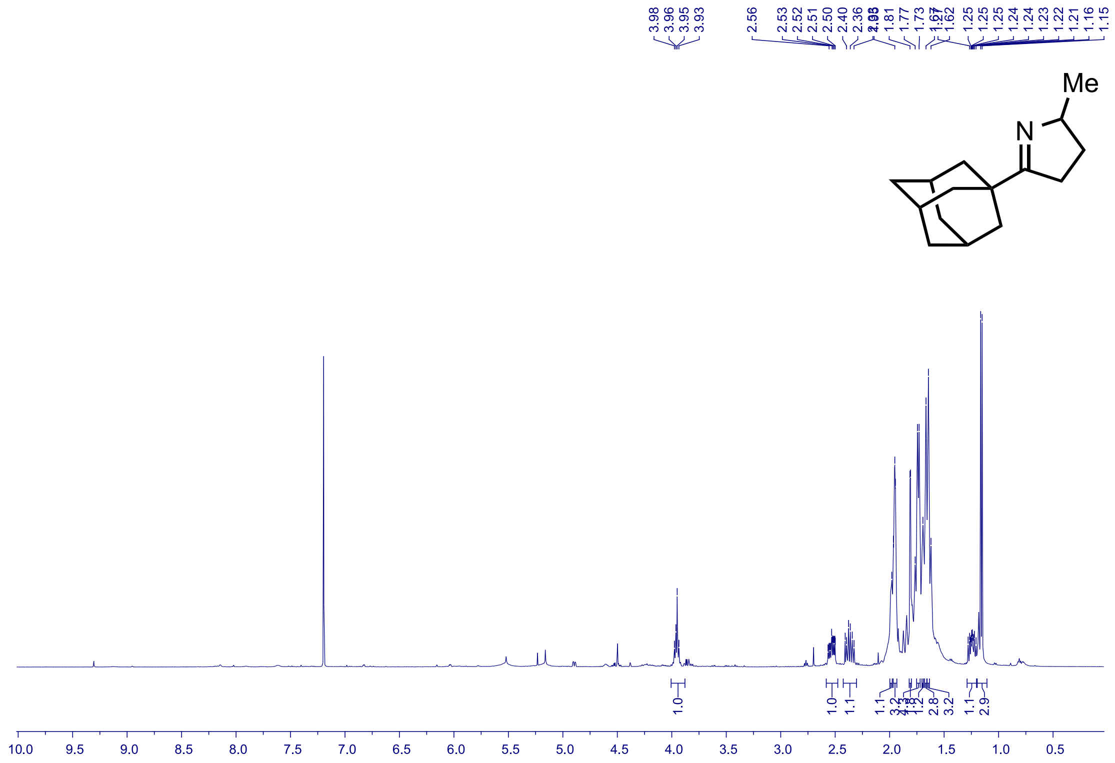

3r: ^{1}H NMR (400 MHz, $CDCl_3$)

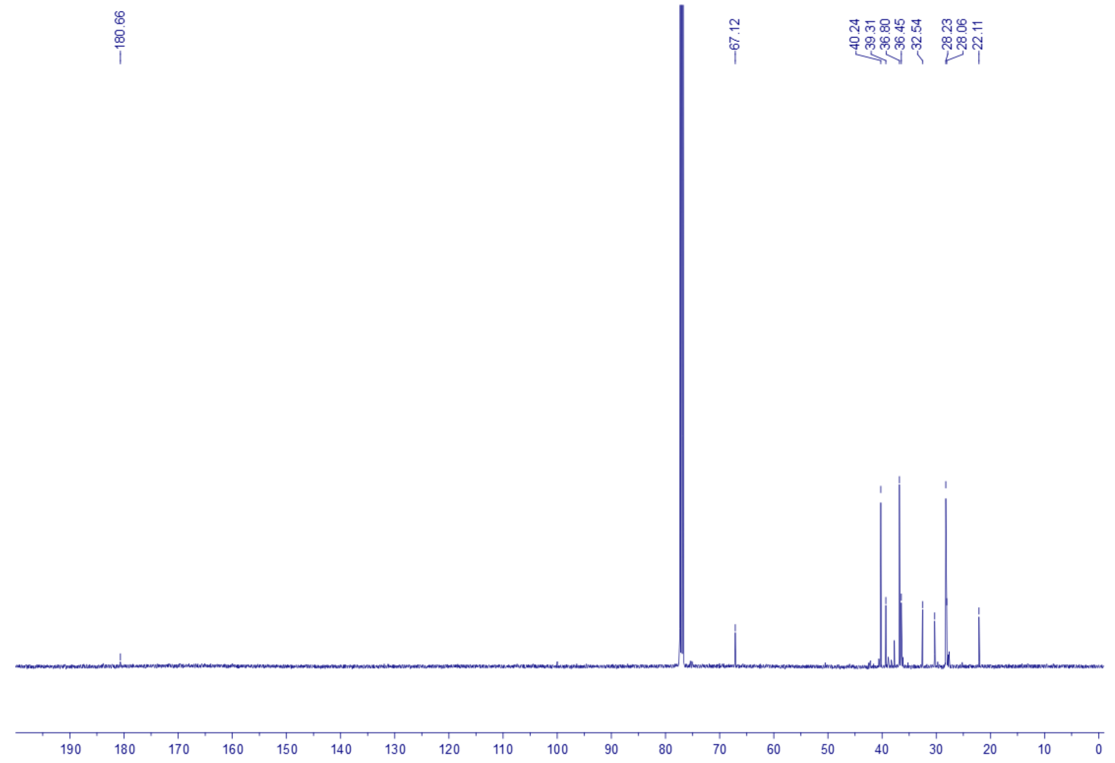

3t: ^{1}H NMR (400 MHz, $CDCl_3$)

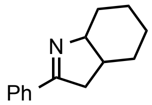

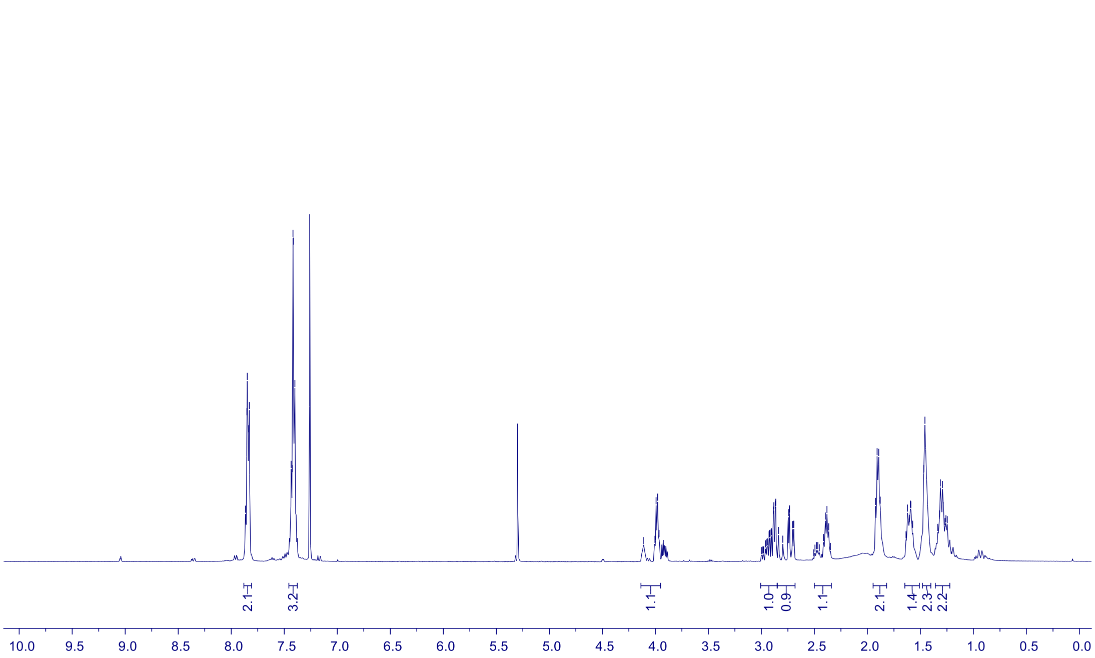

3t: ^{13}C NMR (101 MHz, $CDCl_3$)

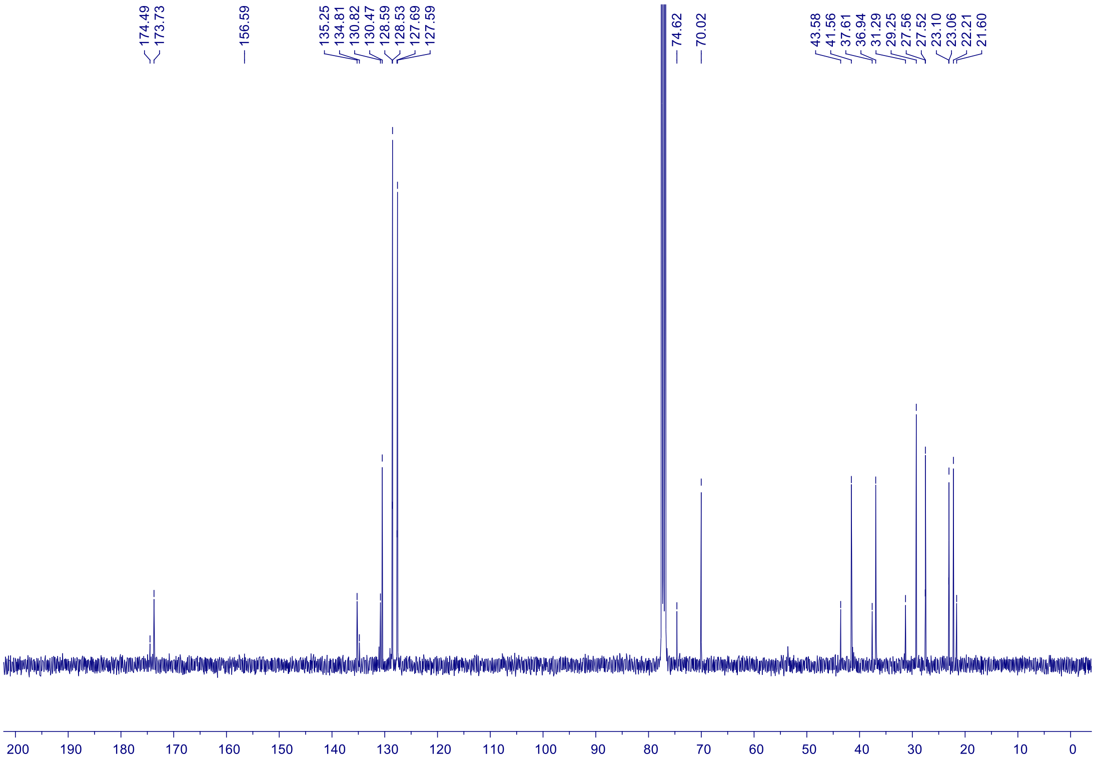

3u: ^{1}H NMR (400 MHz, $CDCl_3$)

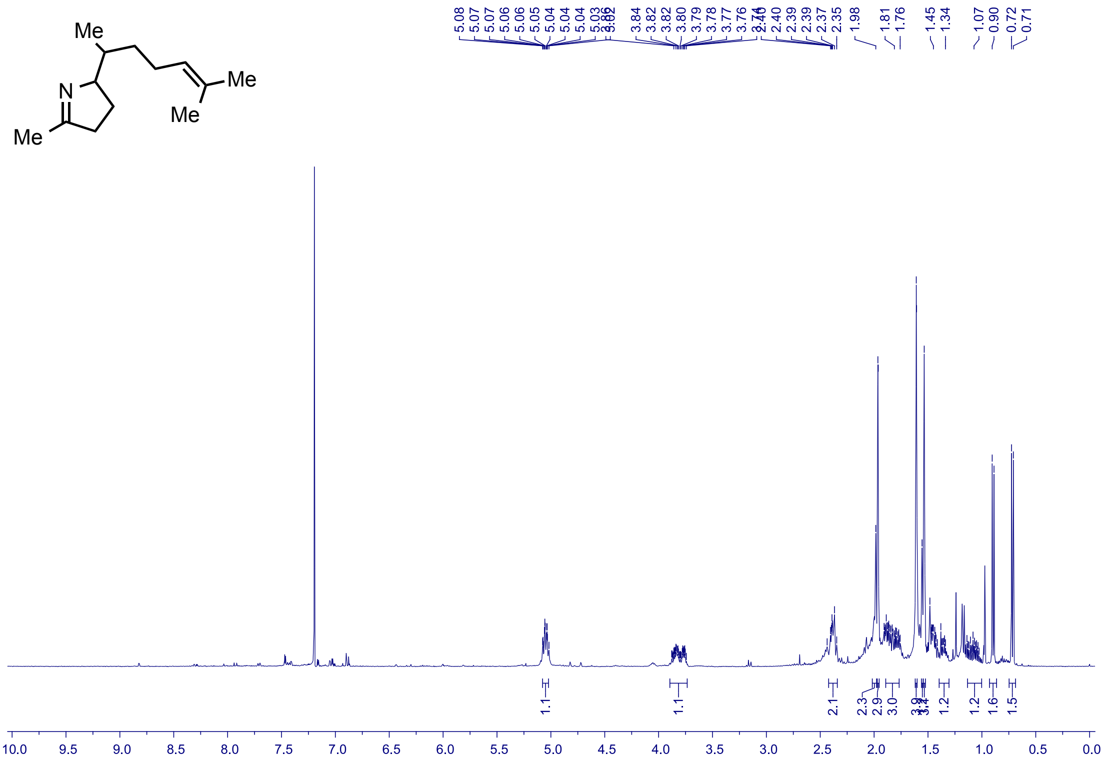

3u: ^{1}H NMR (400 MHz, $CDCl_3$)

4b: ^{1}H NMR (400 MHz, $CDCl_3$)

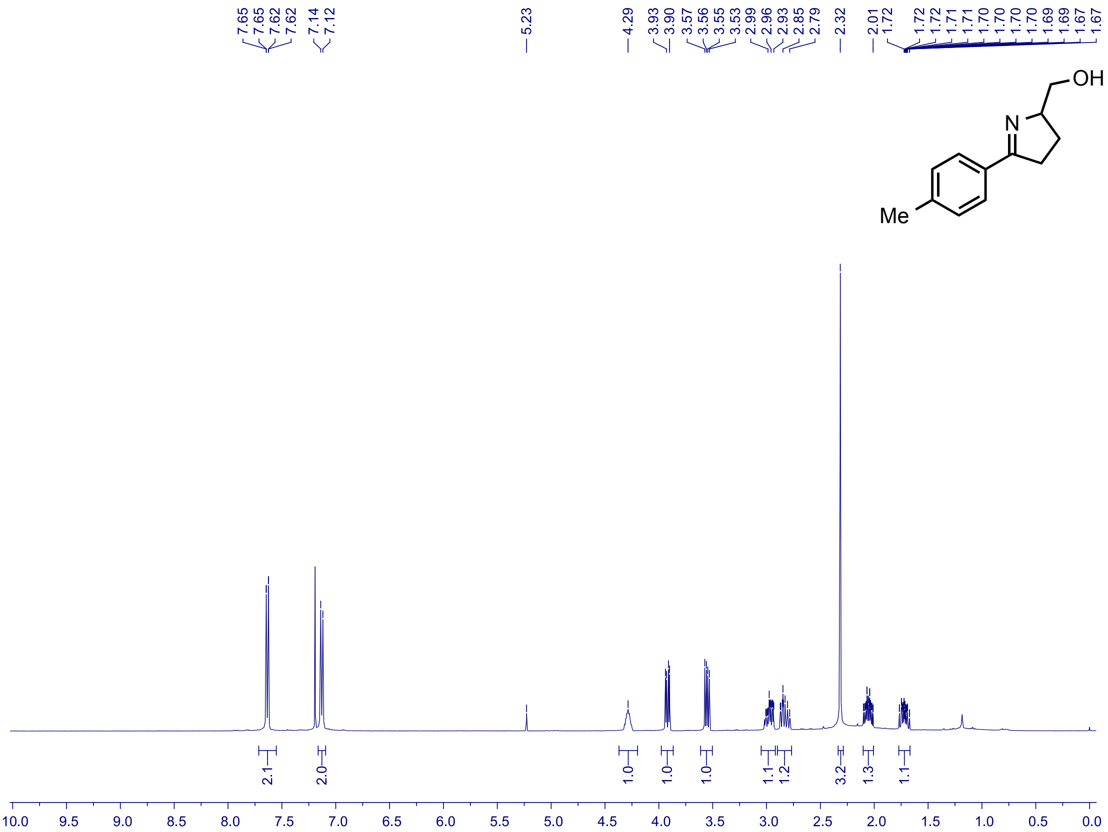

4b: ^{13}C NMR (101 MHz, $CDCl_3$)

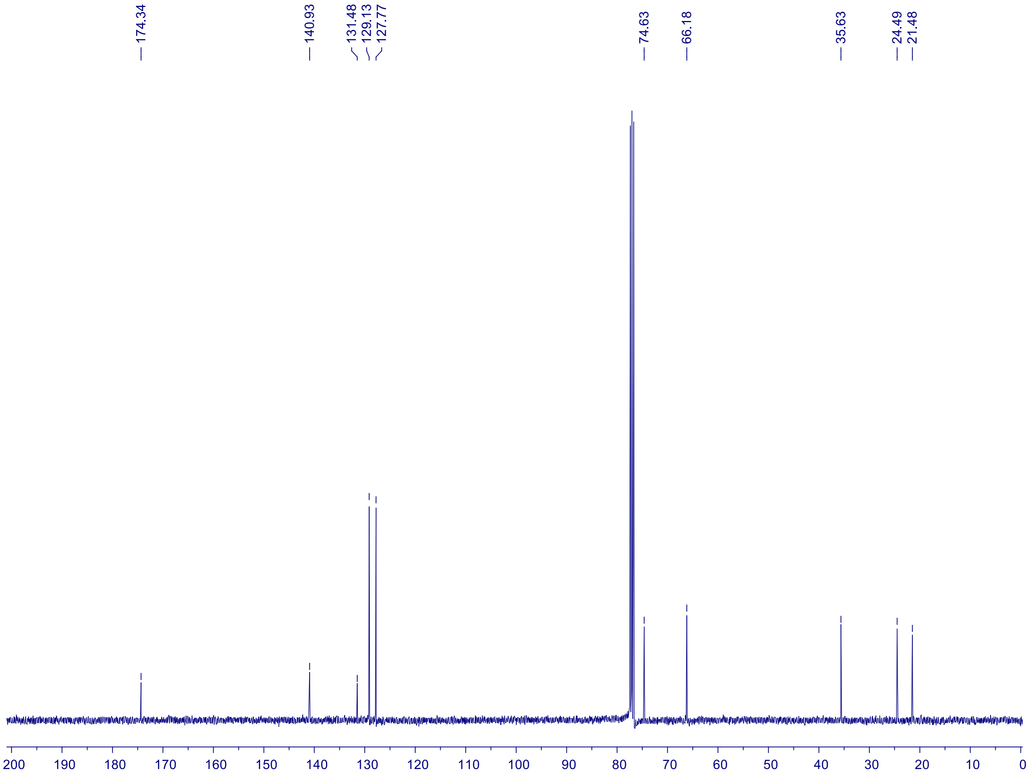

4c: ^{1}H NMR (400 MHz, $CDCl_3$)

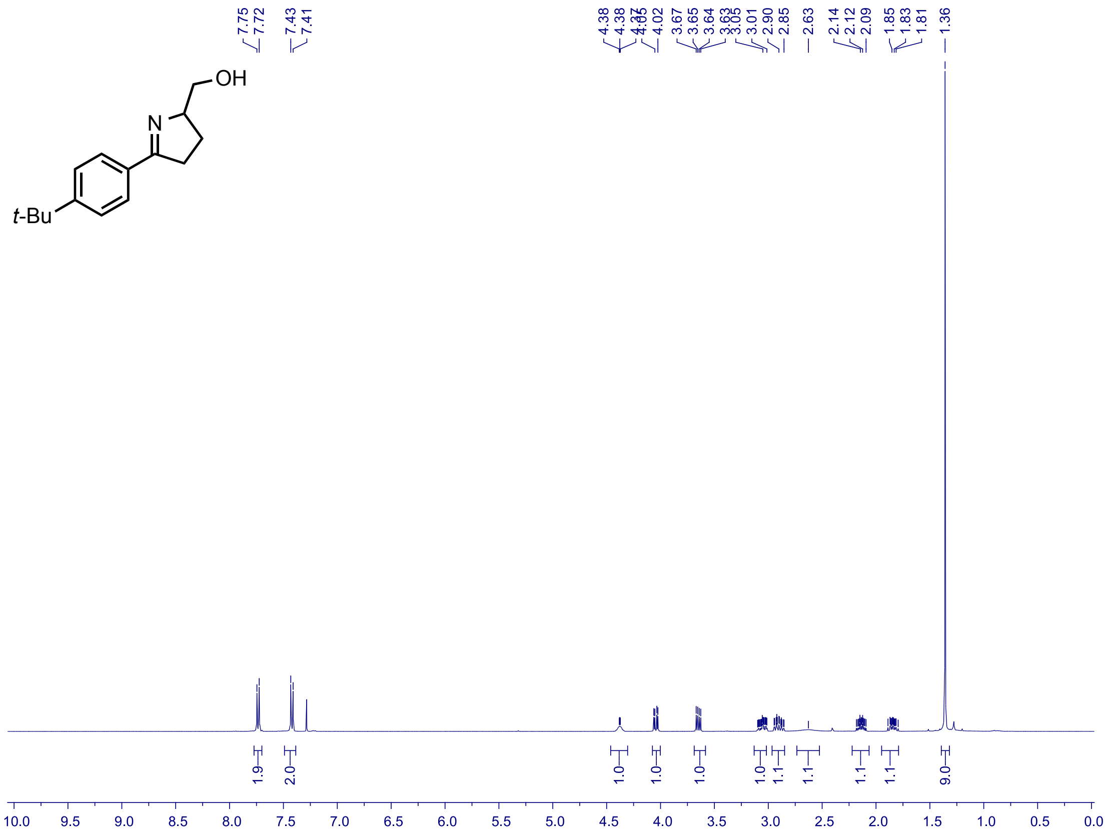

4c: ^{13}C NMR (101 MHz, $CDCl_3$)

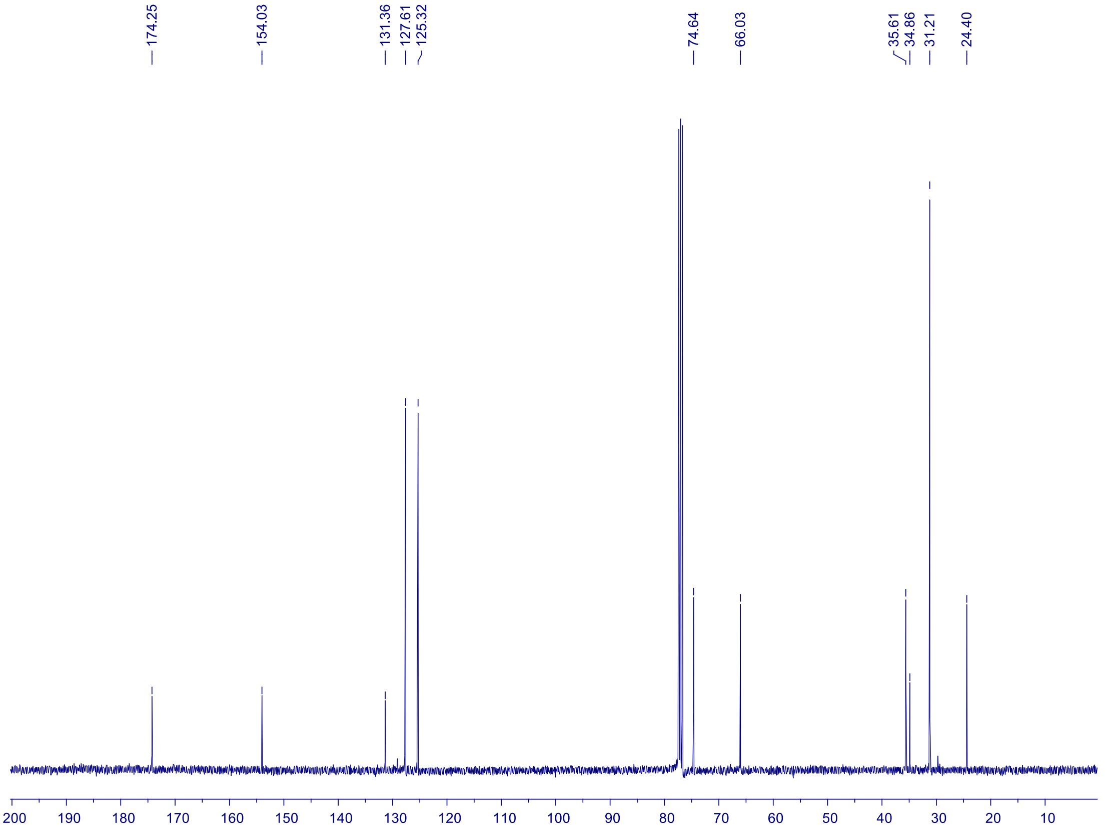

4d: ^{1}H NMR (400 MHz, $CDCl_3$)

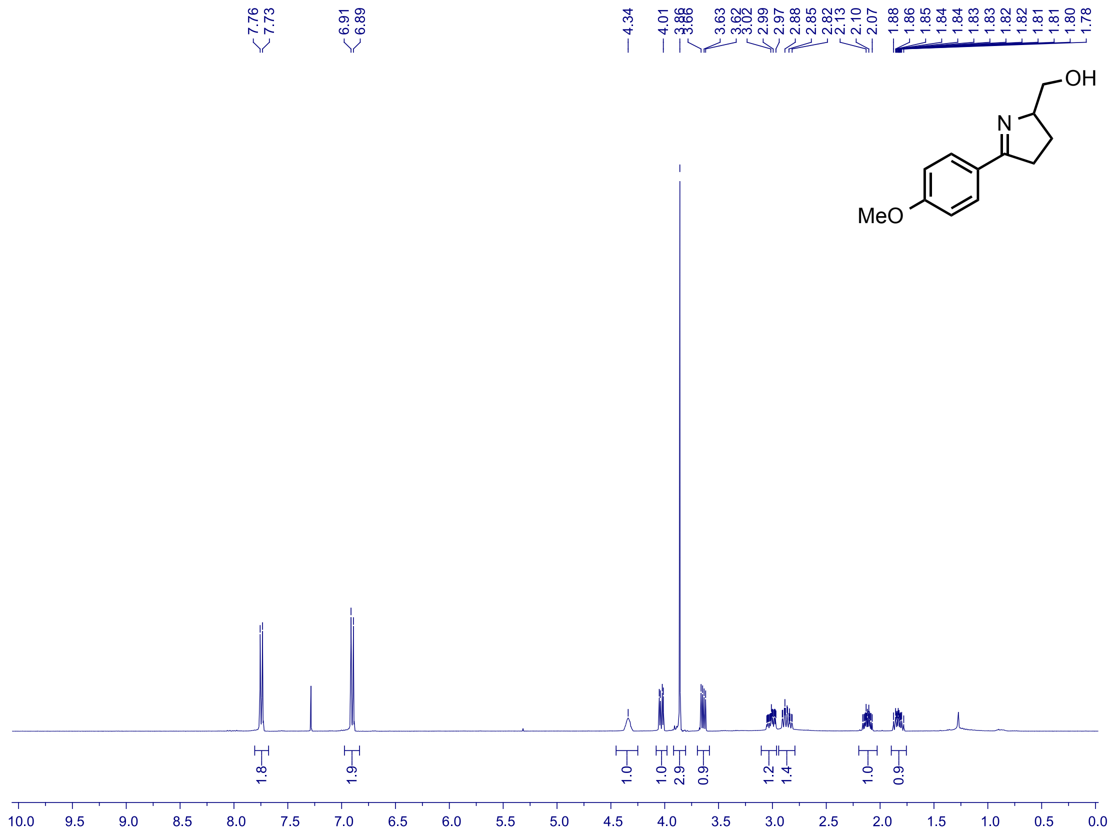

4d: ^{13}C NMR (101 MHz, $CDCl_3$)

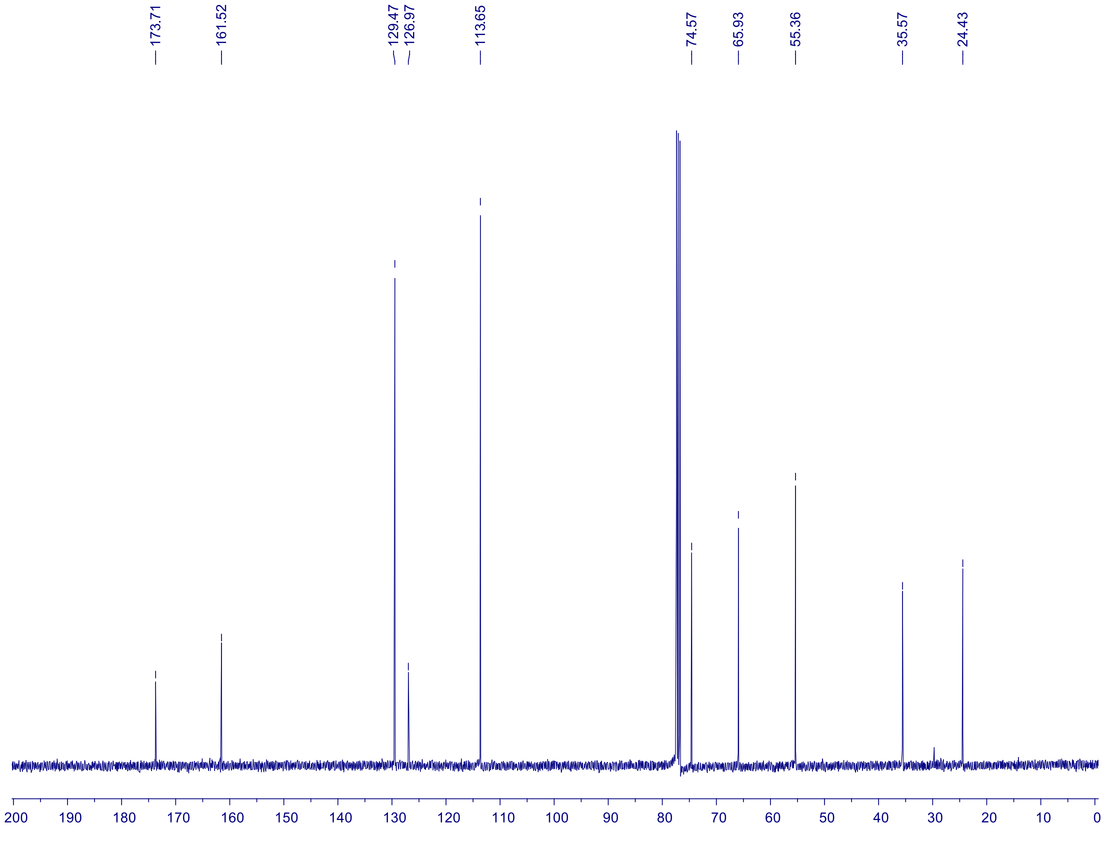

4e: ^{1}H NMR (400 MHz, $CDCl_3$)

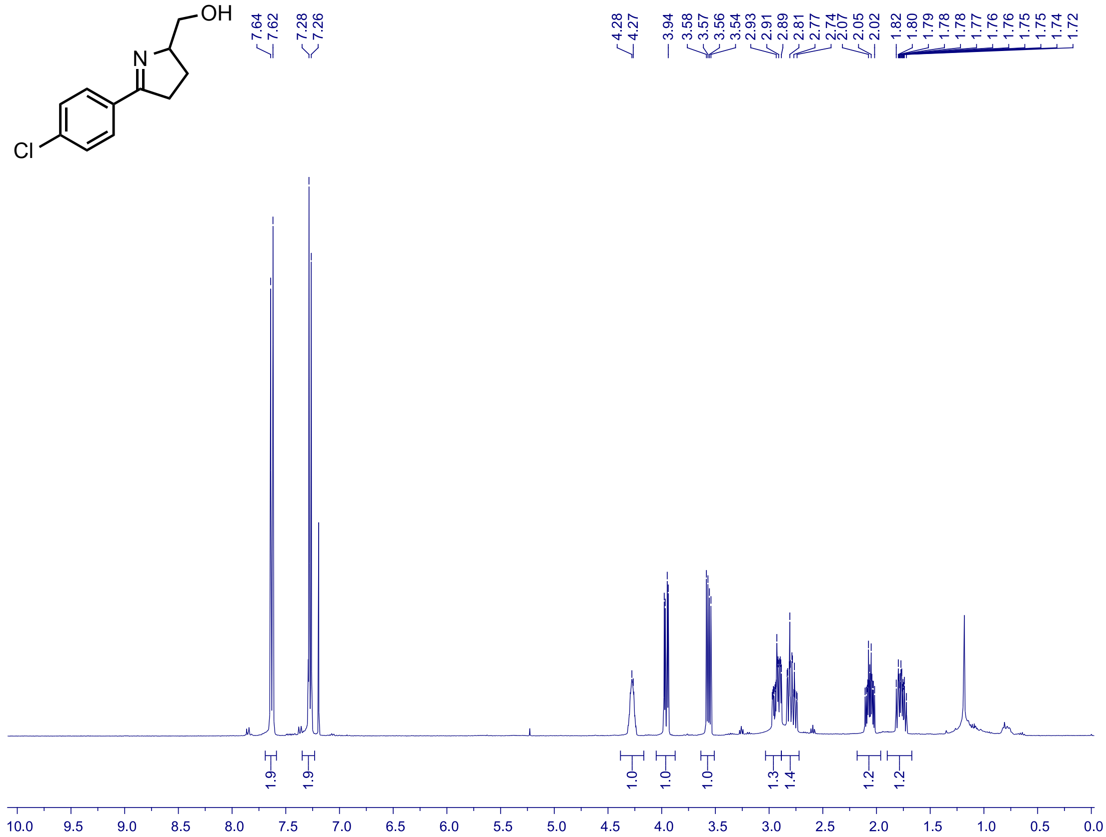

4e: ^{13}C NMR (101 MHz, $CDCl_3$)

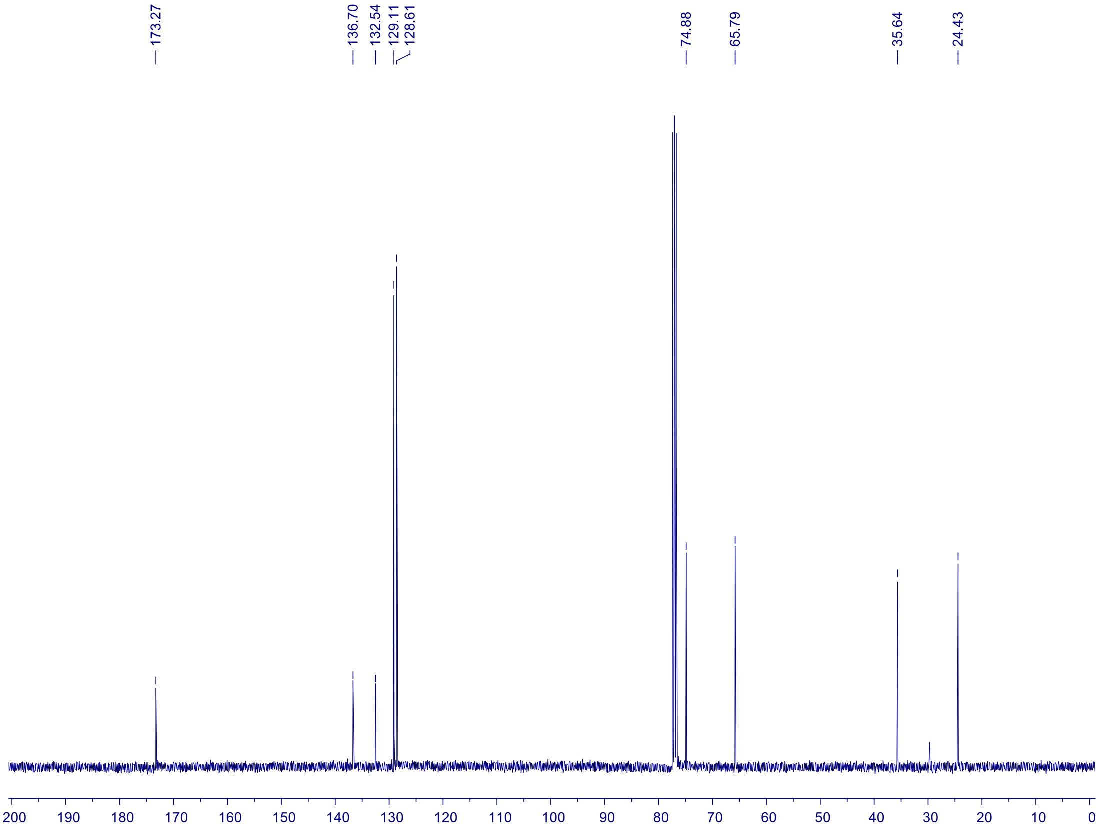

4f: ^{1}H NMR (400 MHz, $CDCl_3$)

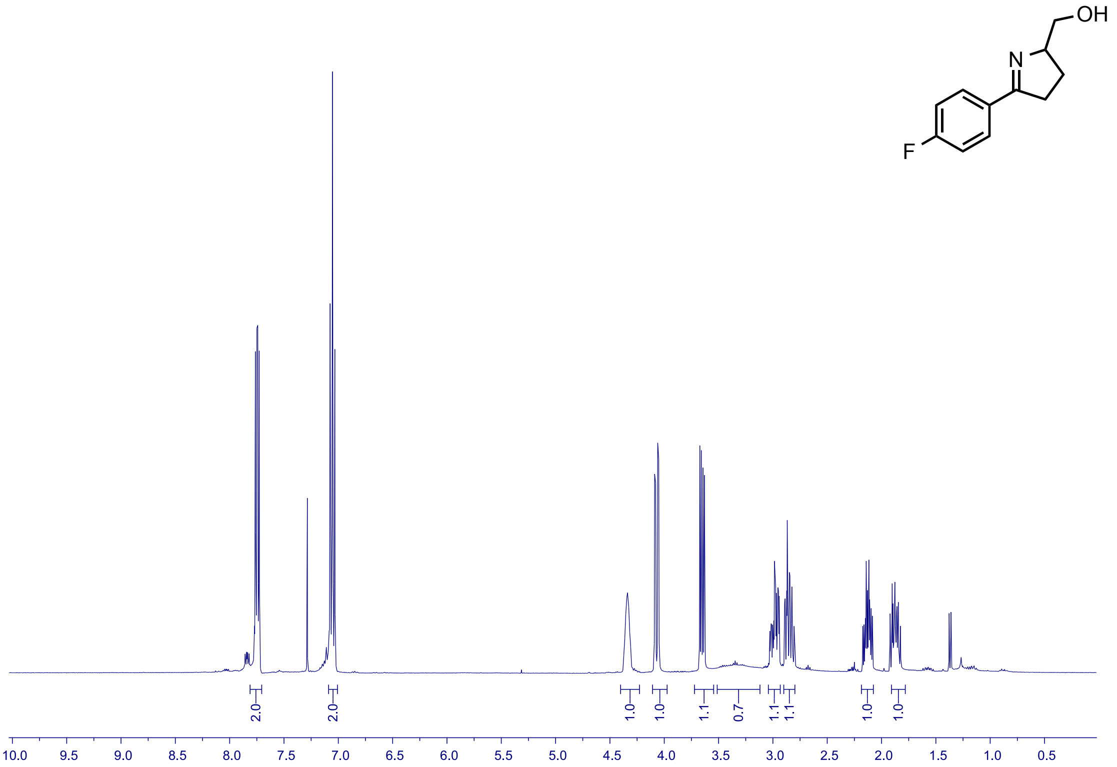

4f: ^{13}C NMR (101 MHz, $CDCl_3$)

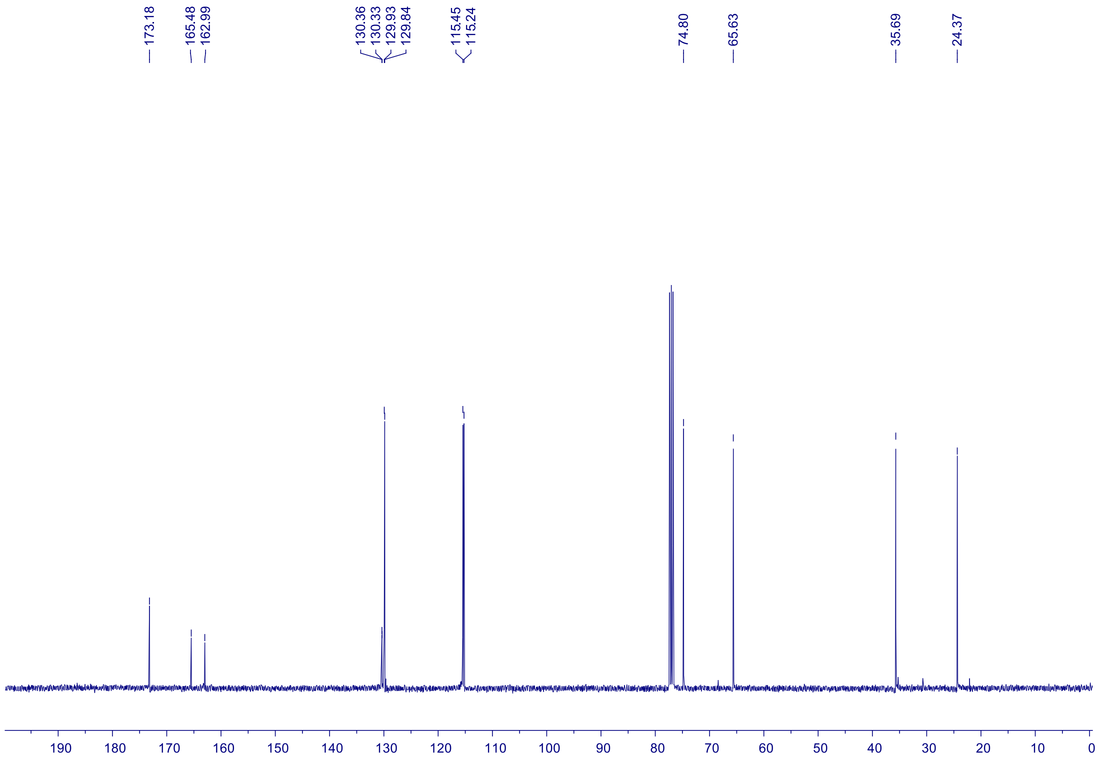

4f: ^{19}F NMR (376 MHz, $CDCl_3$)

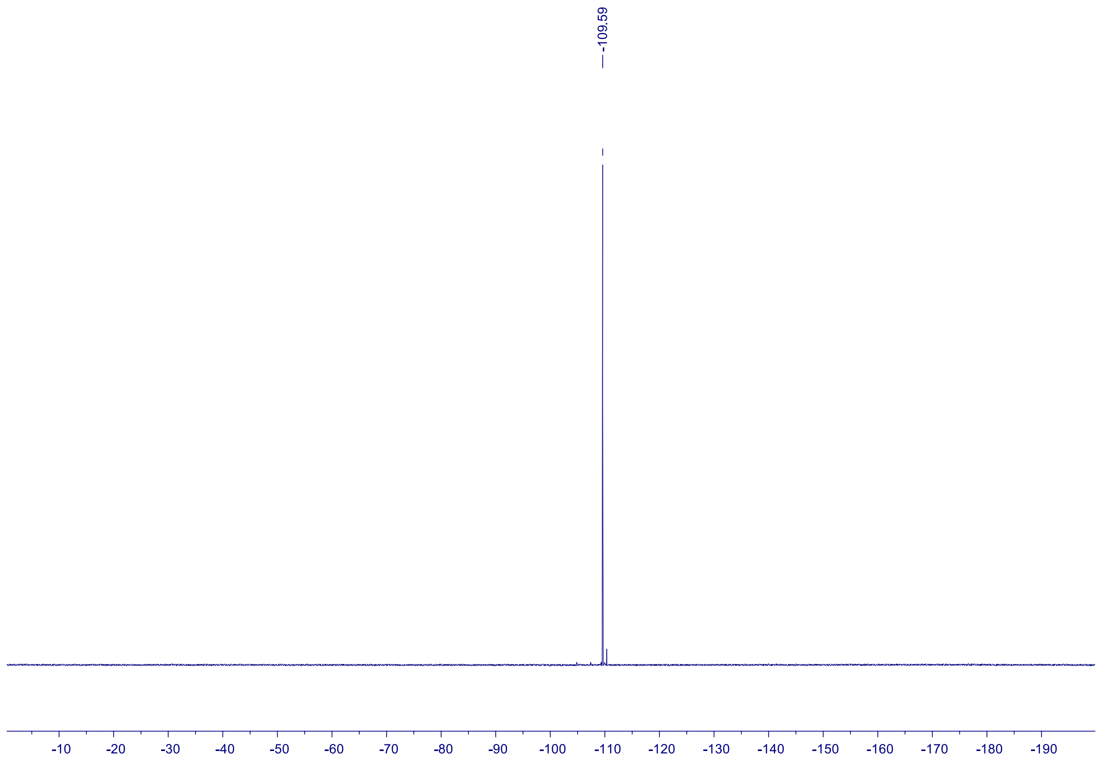

4g: ^{1}H NMR (400 MHz, $CDCl_3$)

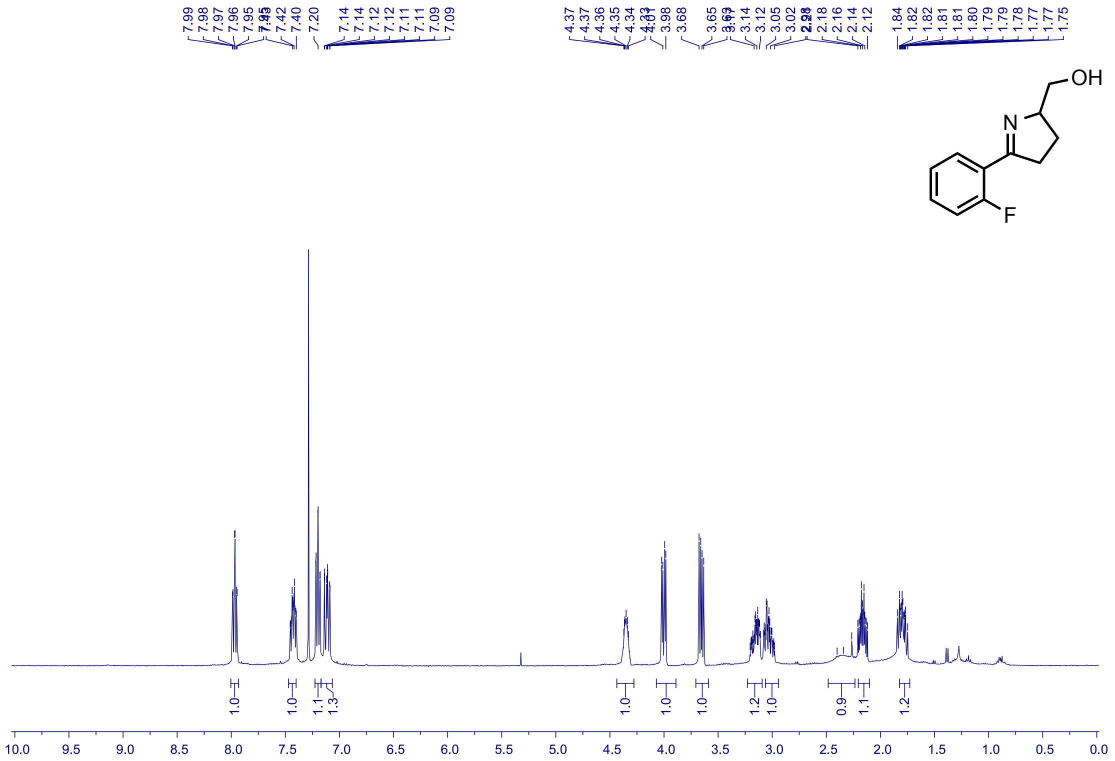

4g: ^{13}C NMR (101 MHz, $CDCl_3$)

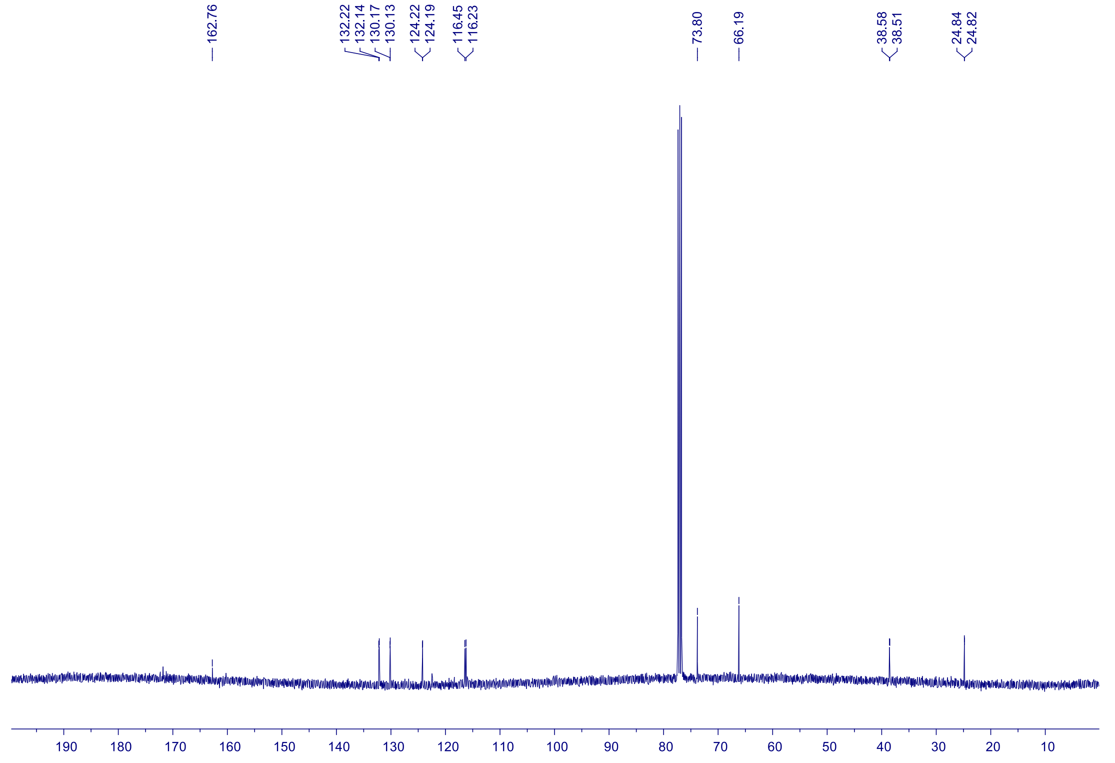

4g: ^{19}F NMR (376 MHz, $CDCl_3$)

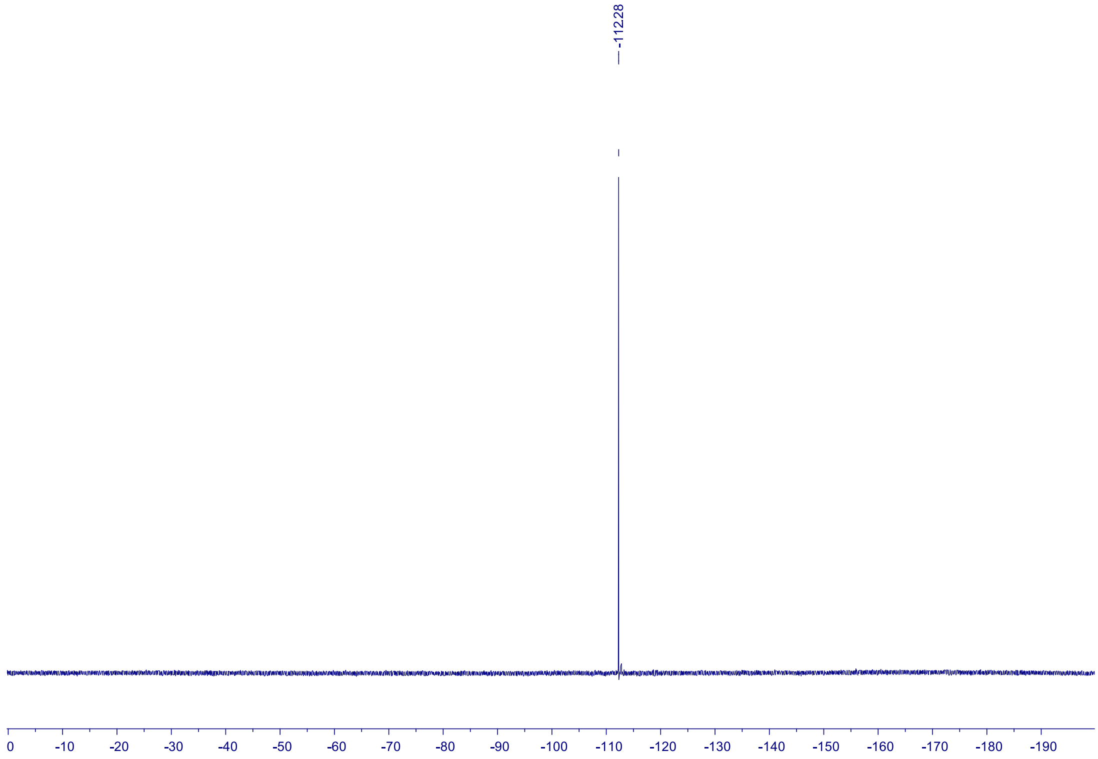

4h: ^{1}H NMR (400 MHz, $CDCl_3$)

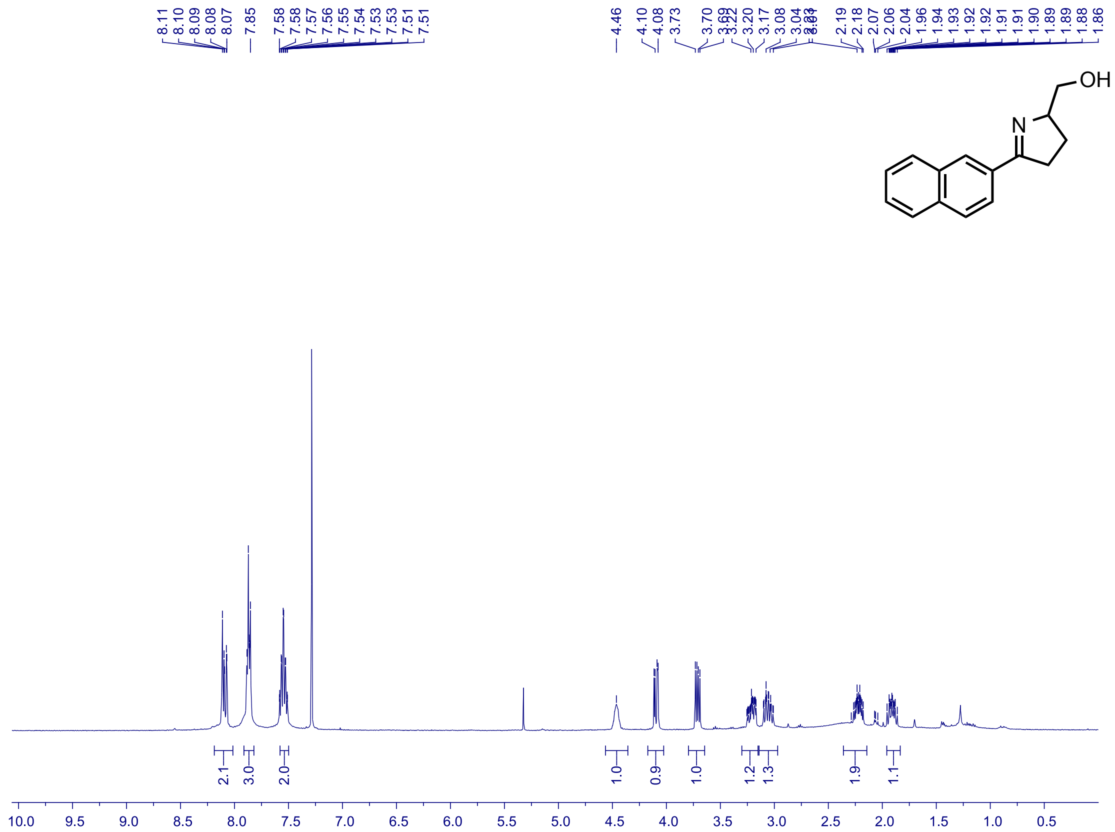

4h: ^{13}C NMR (101 MHz, $CDCl_3$)

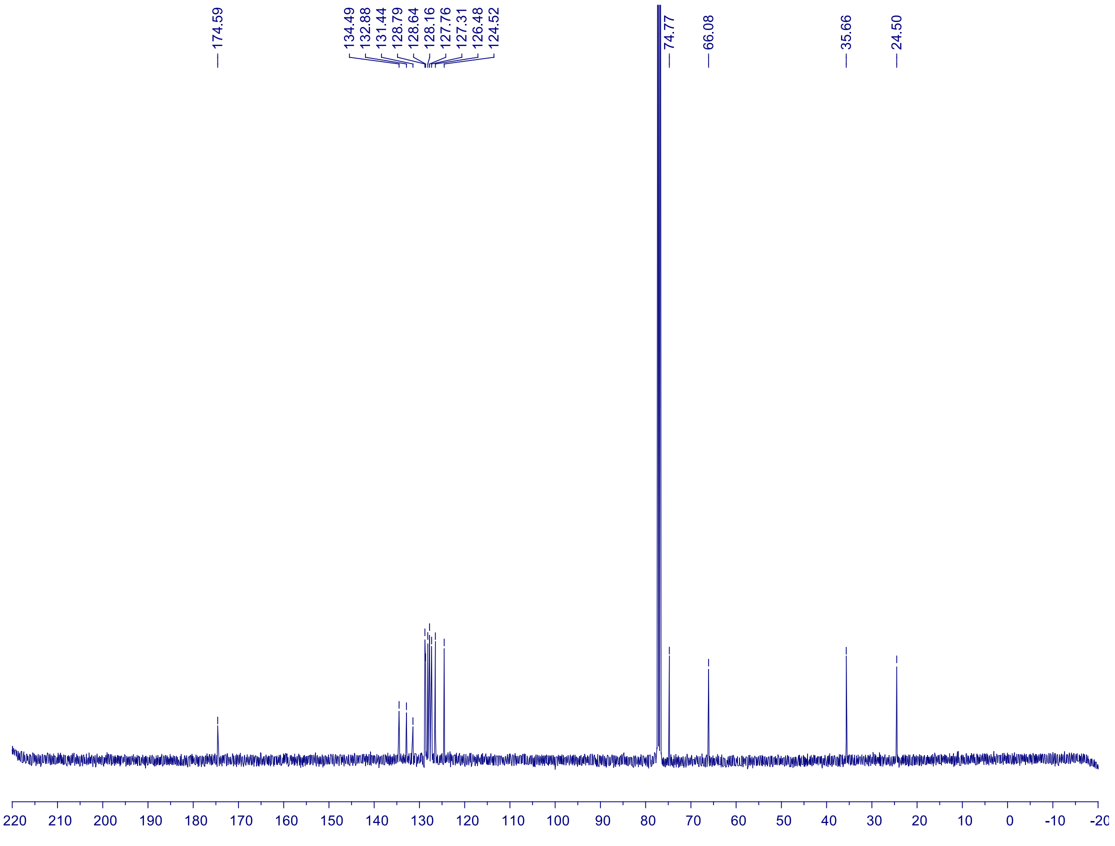

4i: ^{1}H NMR (400 MHz, $CDCl_3$)

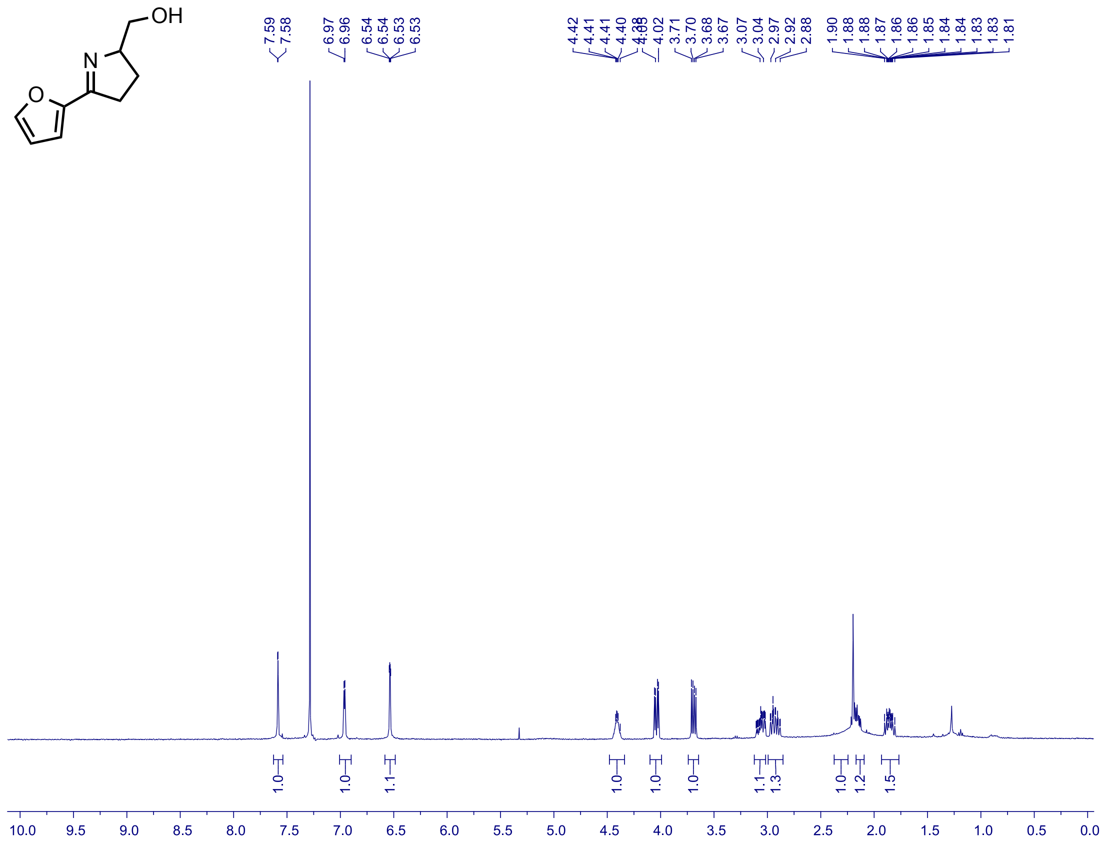

4i: ^{13}C NMR (101 MHz, $CDCl_3$)

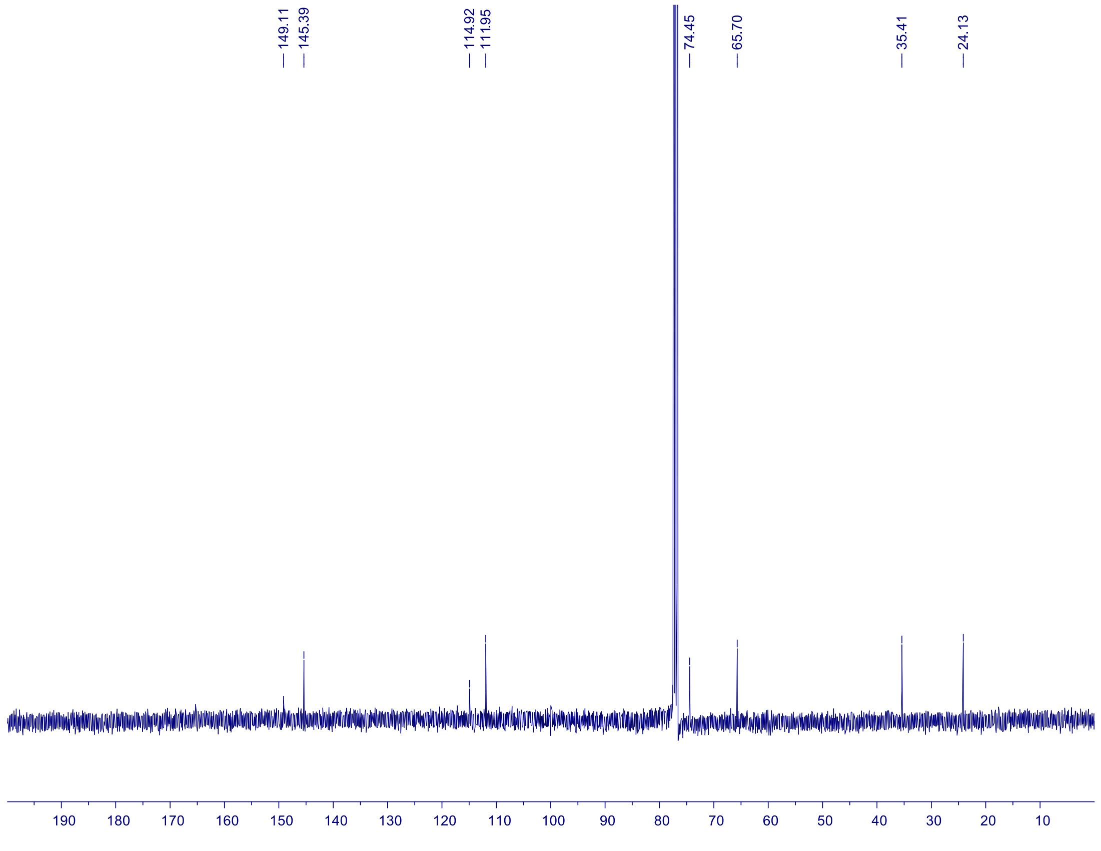

4j: ^{1}H NMR (400 MHz, $CDCl_3$)

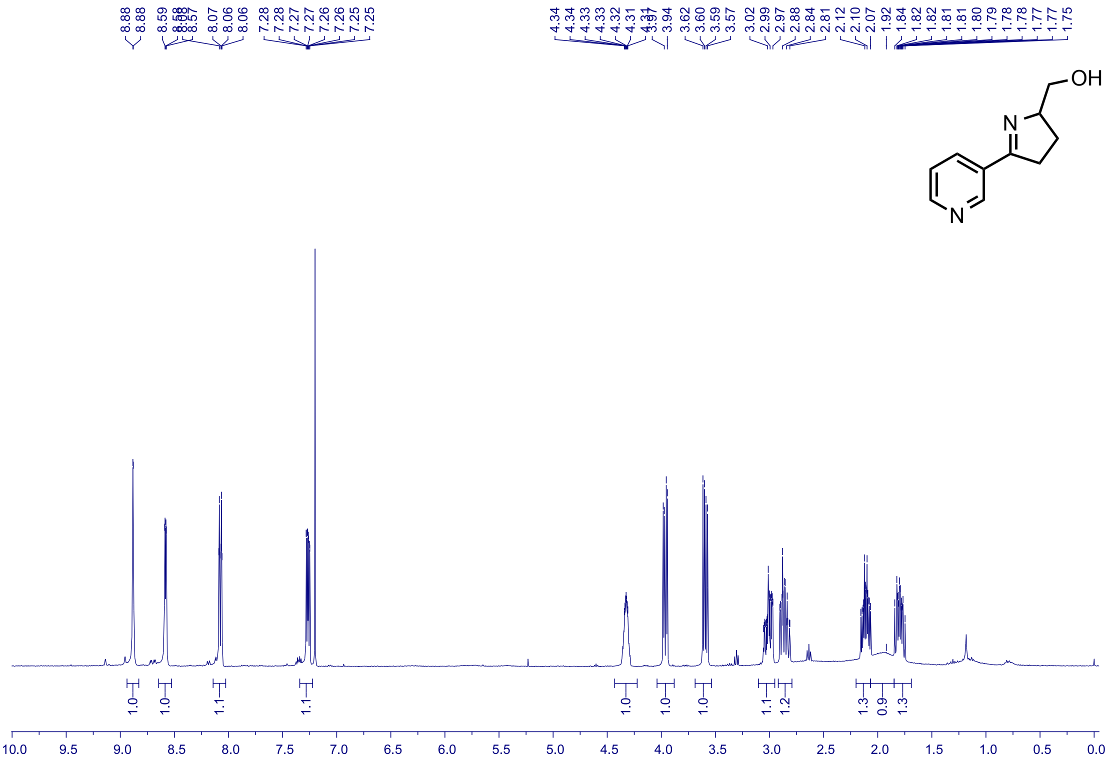

4j: ^{13}C NMR (101 MHz, $CDCl_3$

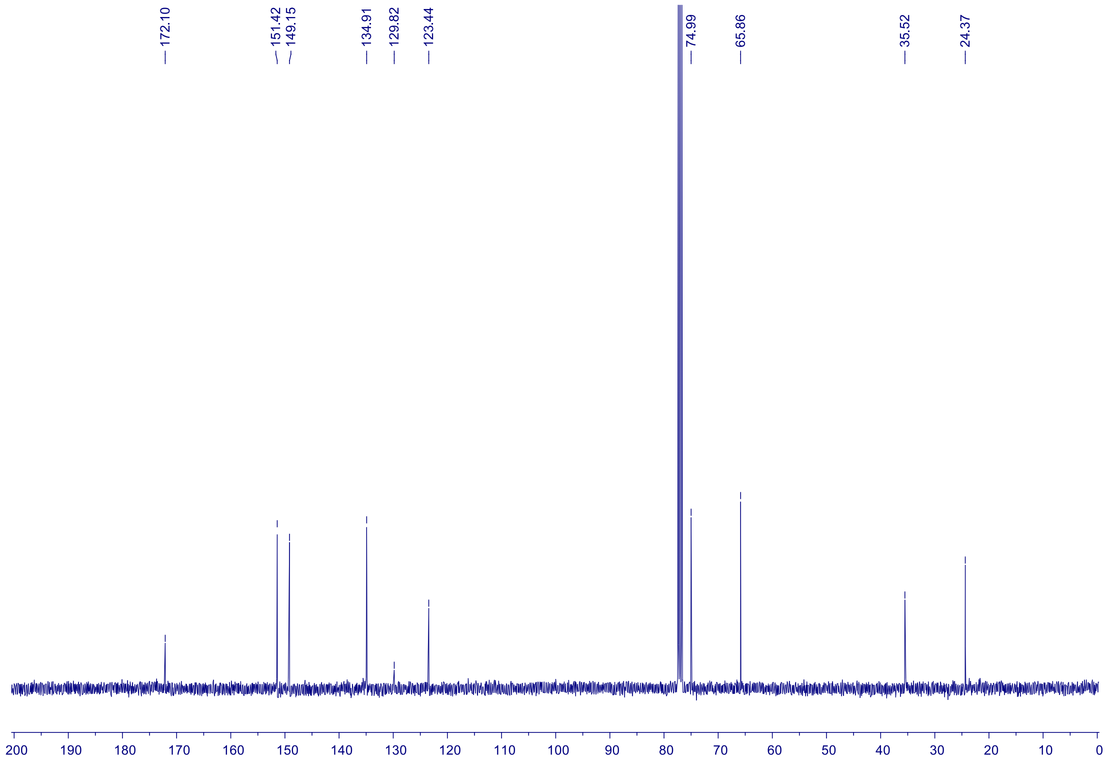

4k: ^{1}H NMR (400 MHz, $CDCl_3$)

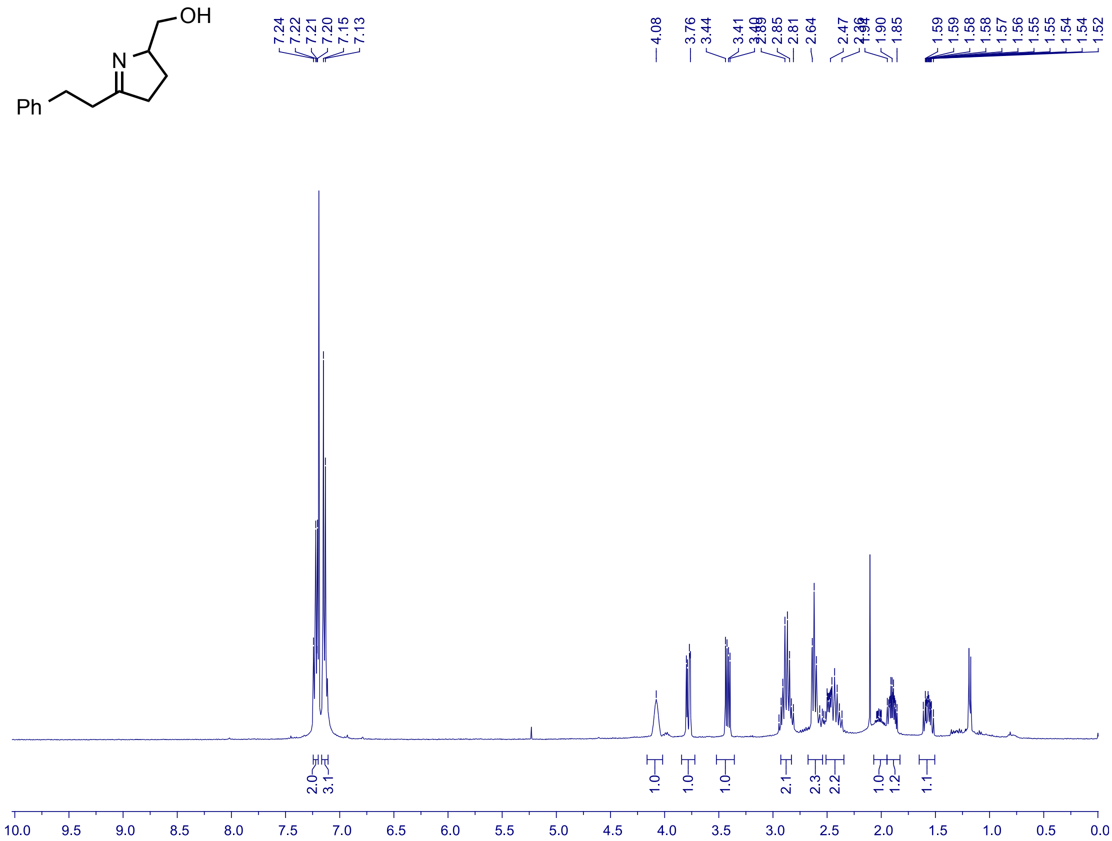

4k: ^{13}C NMR (101 MHz, $CDCl_3$)

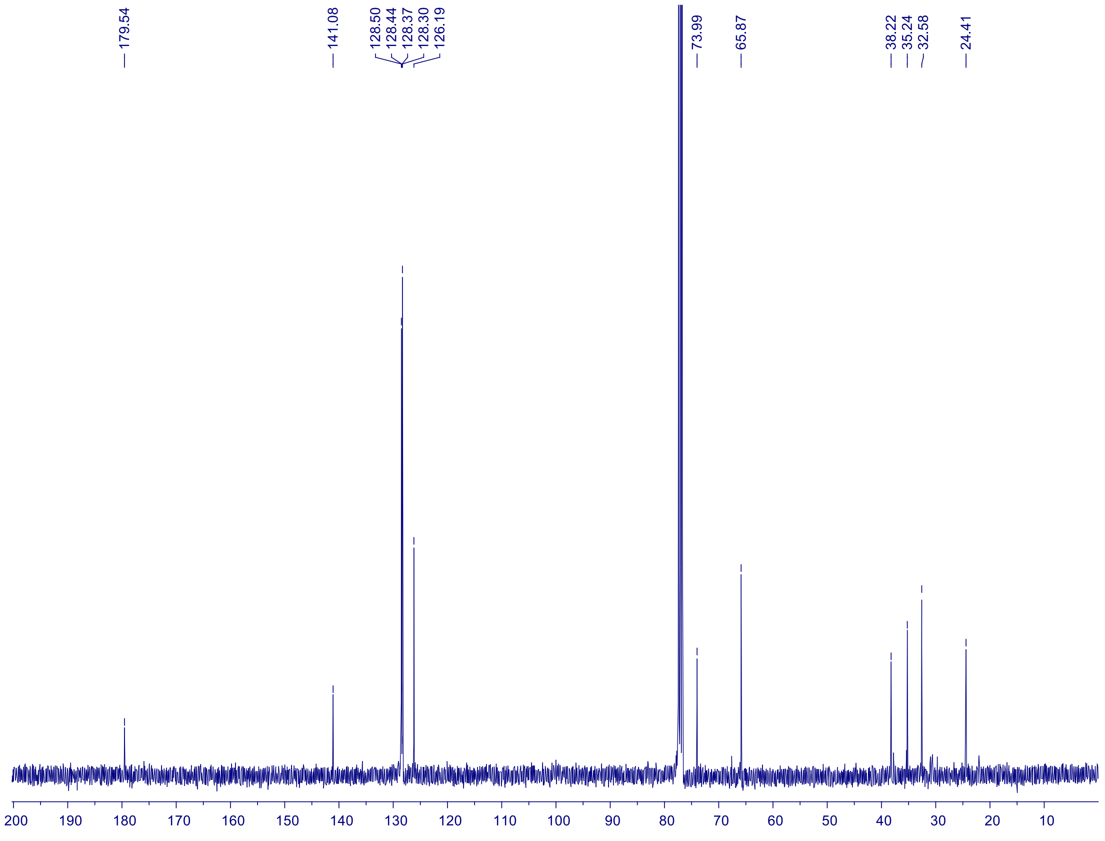

4l: ^{1}H NMR (400 MHz, CDCl$_3$)

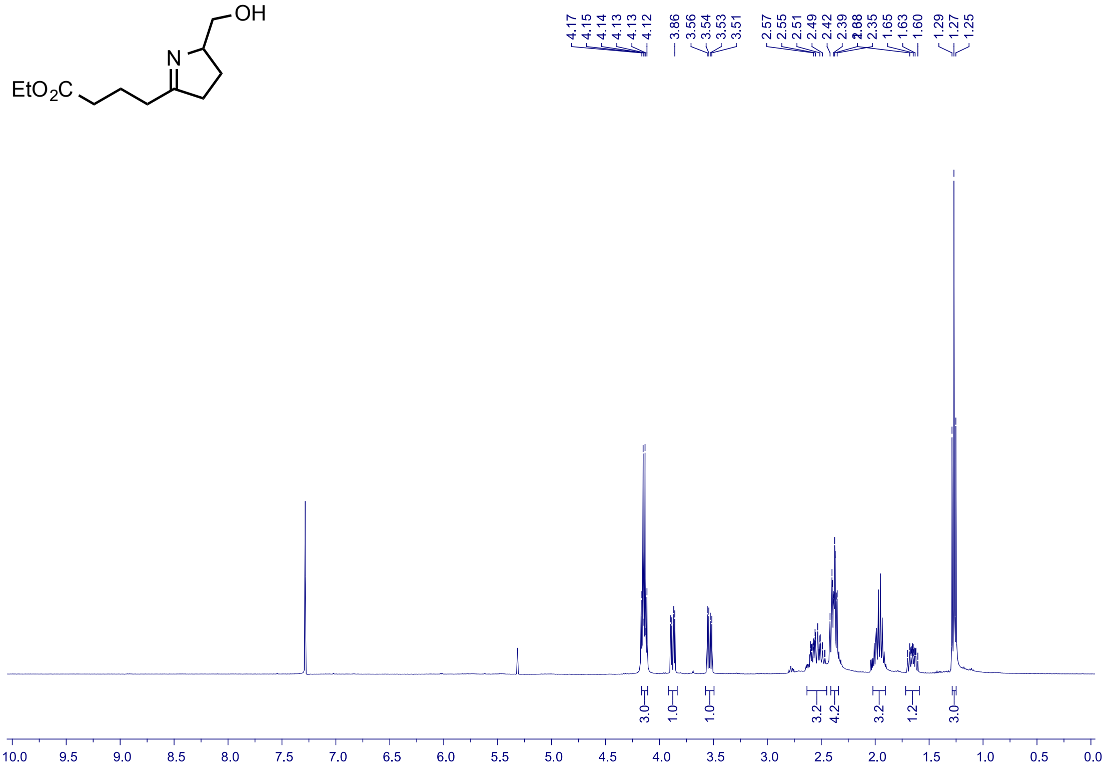

4l: ^{13}C NMR (101 MHz, CDCl$_3$)

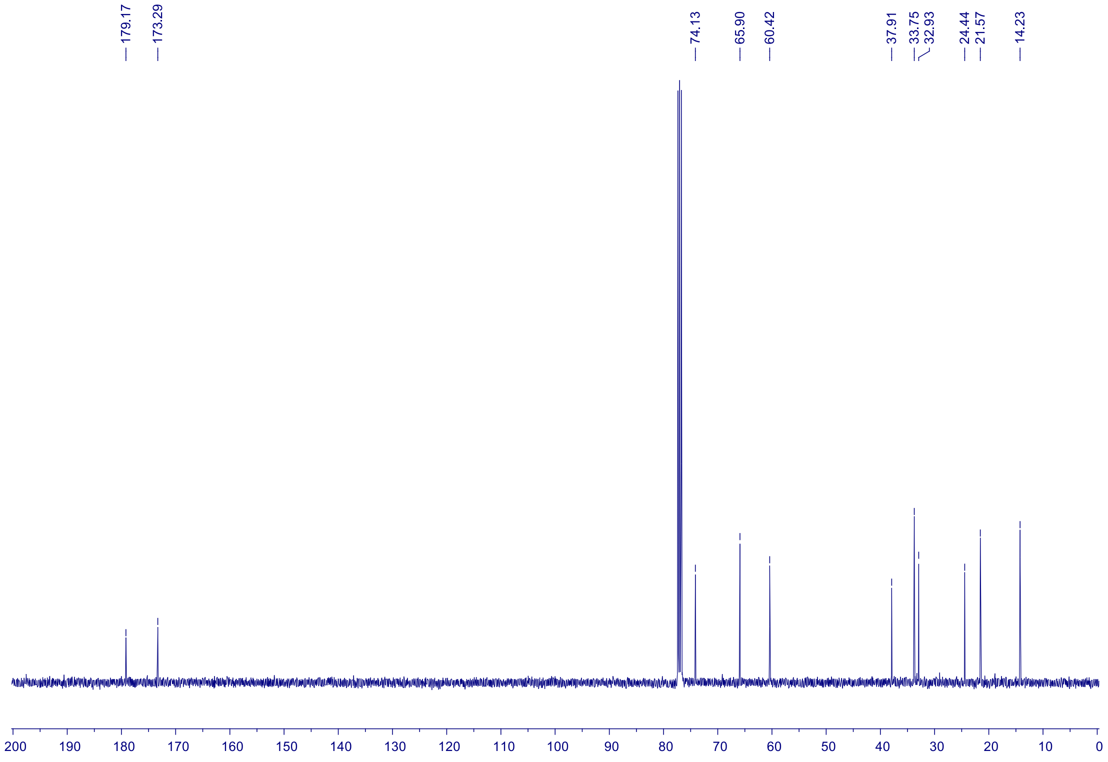

4m: ^{1}H NMR (400 MHz, $CDCl_3$)

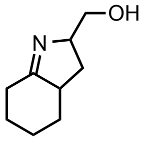

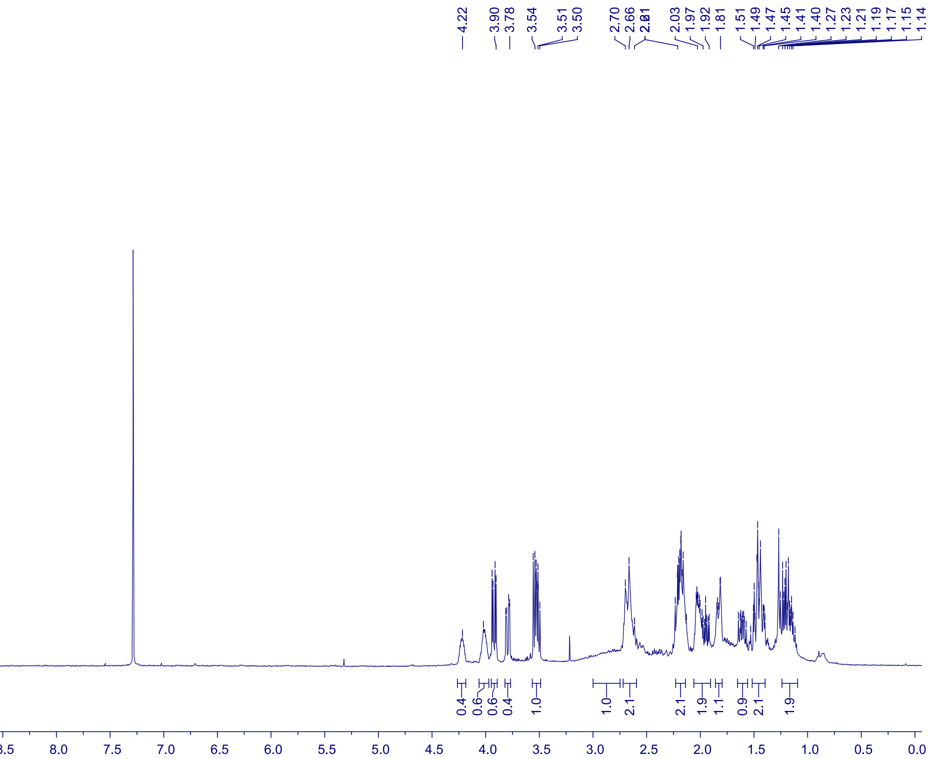

4m: ^{13}C NMR (101 MHz, $CDCl_3$)

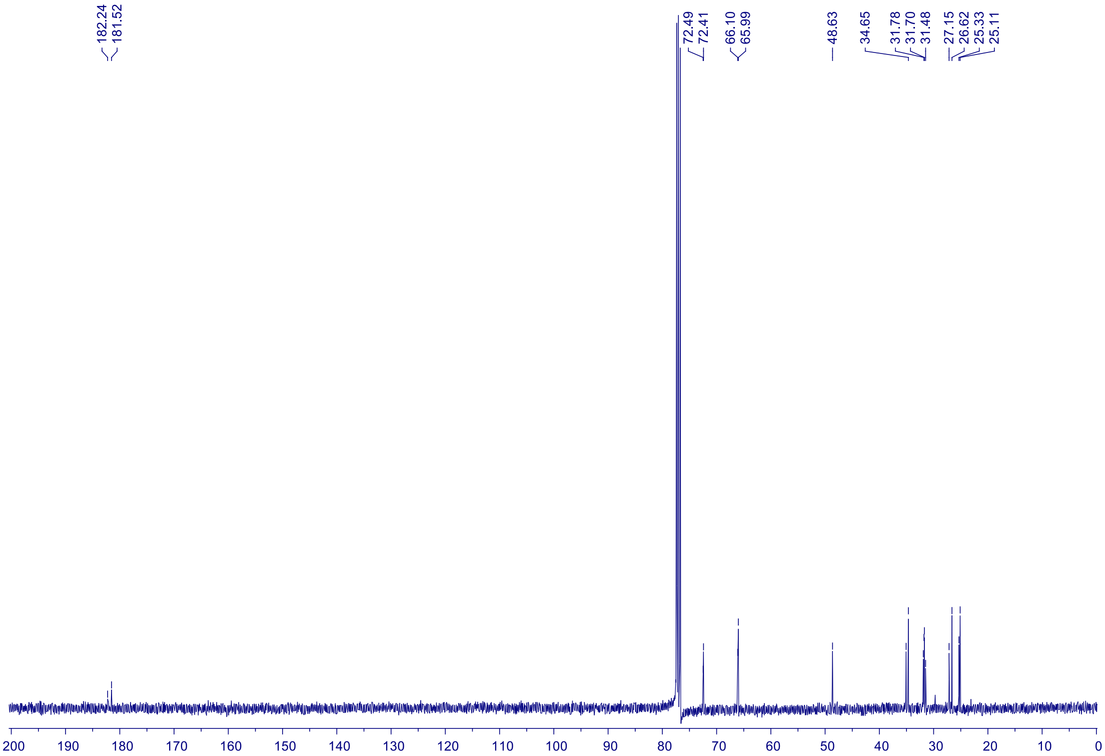

4n: ^{1}H NMR (400 MHz, CDCl$_3$)

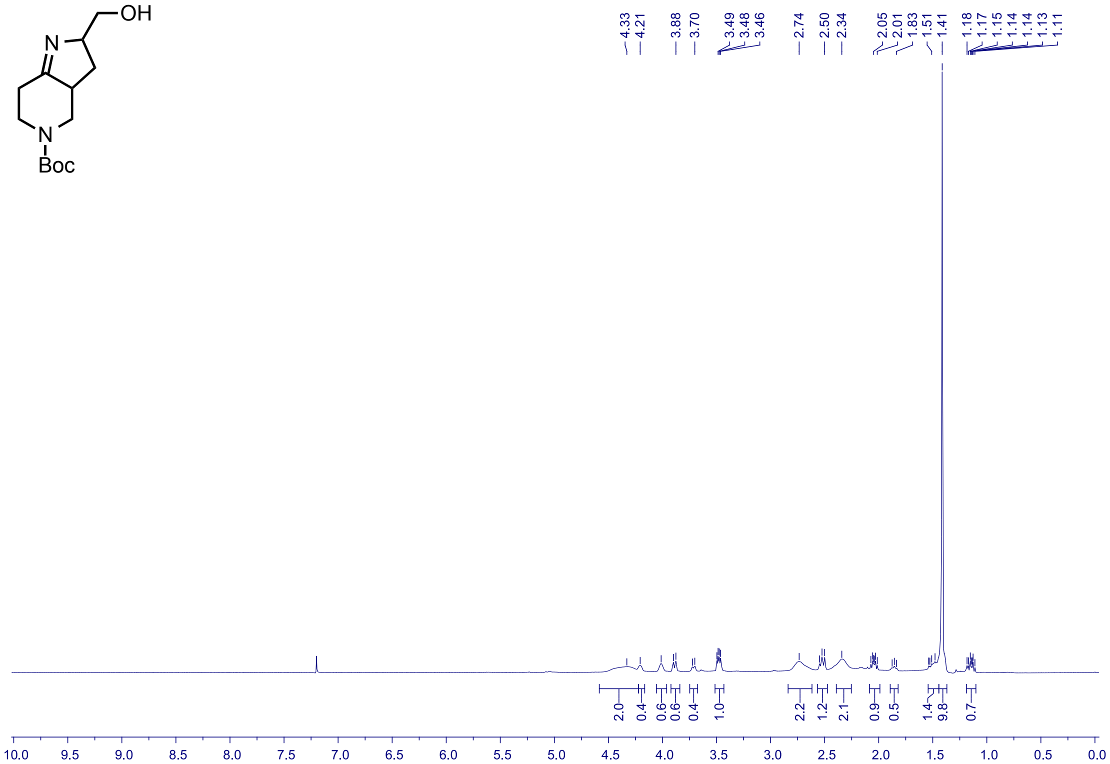

4n: ^{1}H NMR (400 MHz, CDCl$_3$)

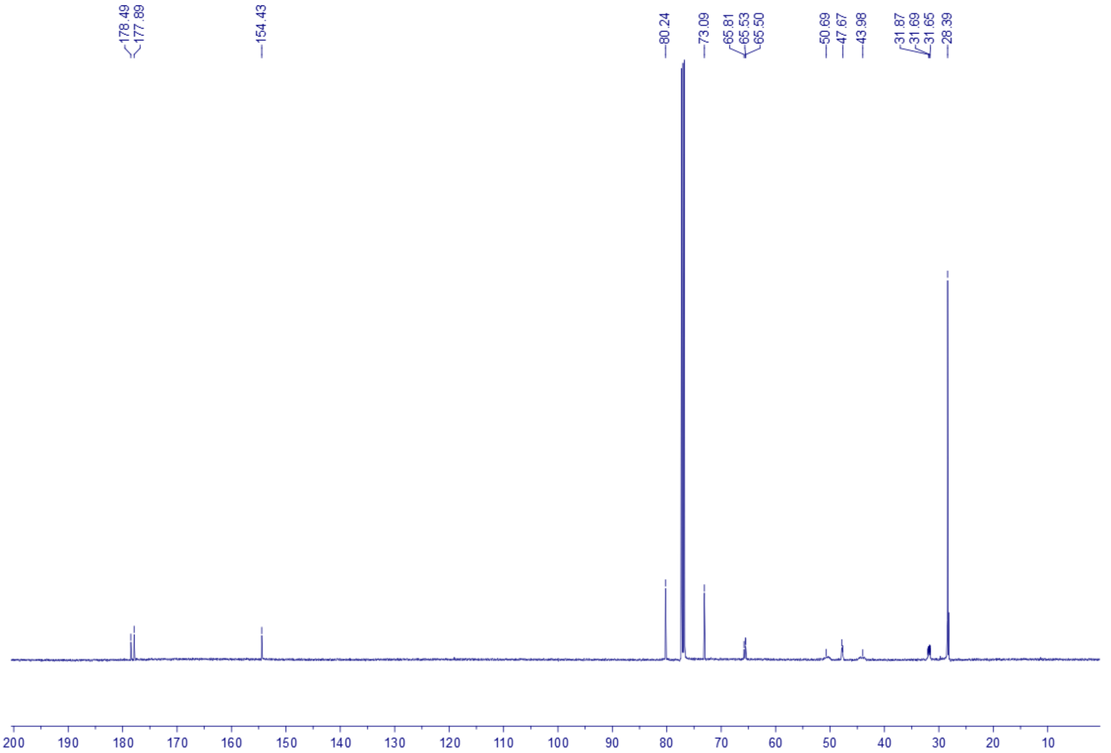

4o: ^{1}H NMR (400 MHz, CDCl$_3$)

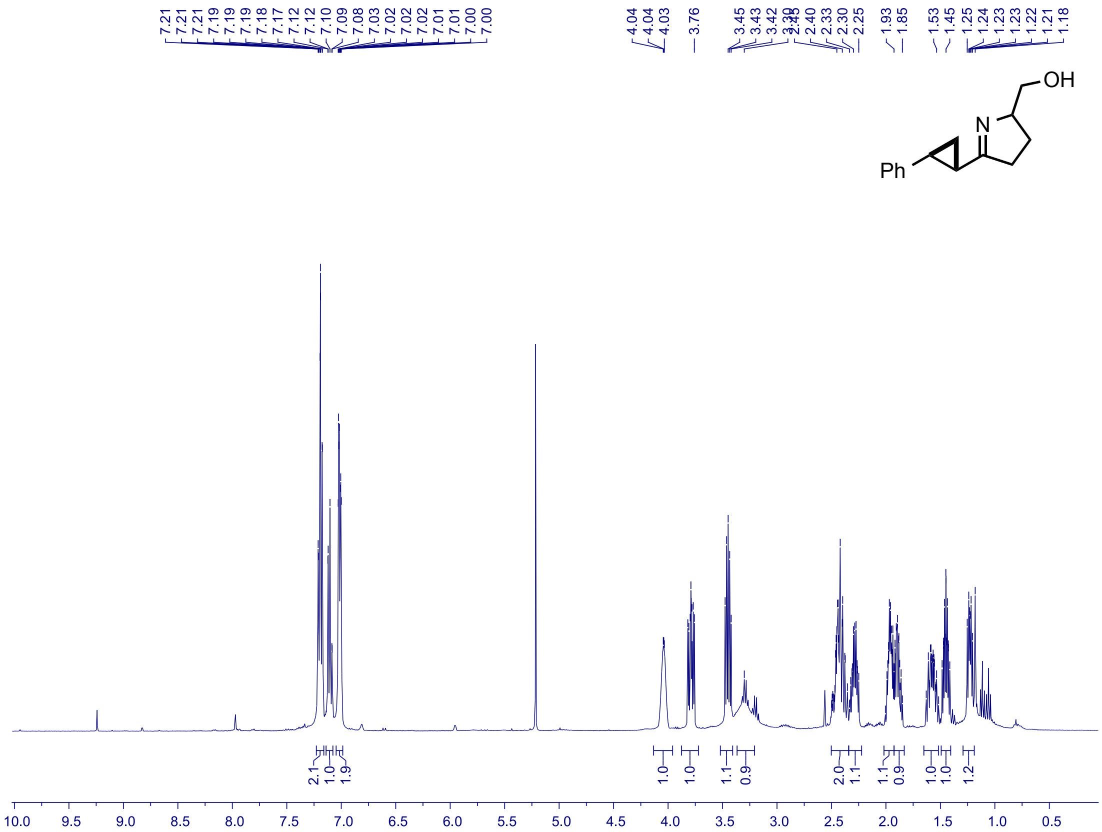

4o: ^{13}C NMR (101 MHz, CDCl$_3$)

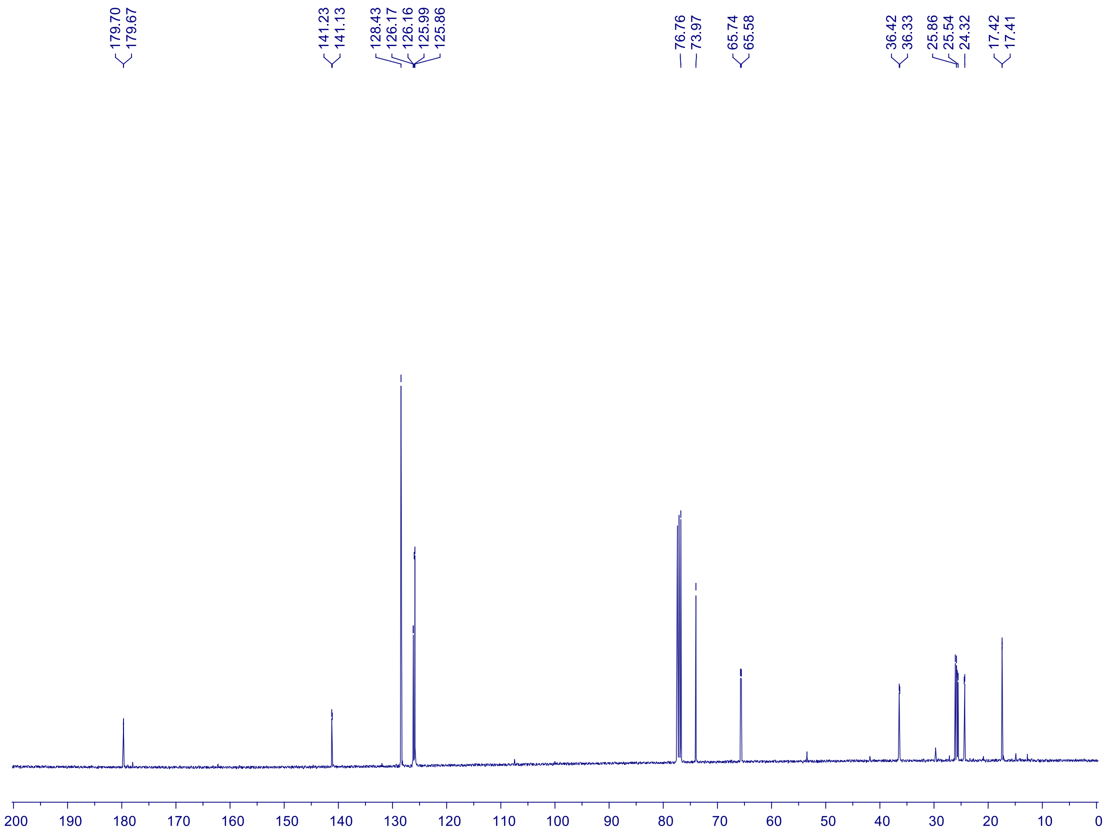

4p: ^{1}H NMR (400 MHz, $CDCl_3$)

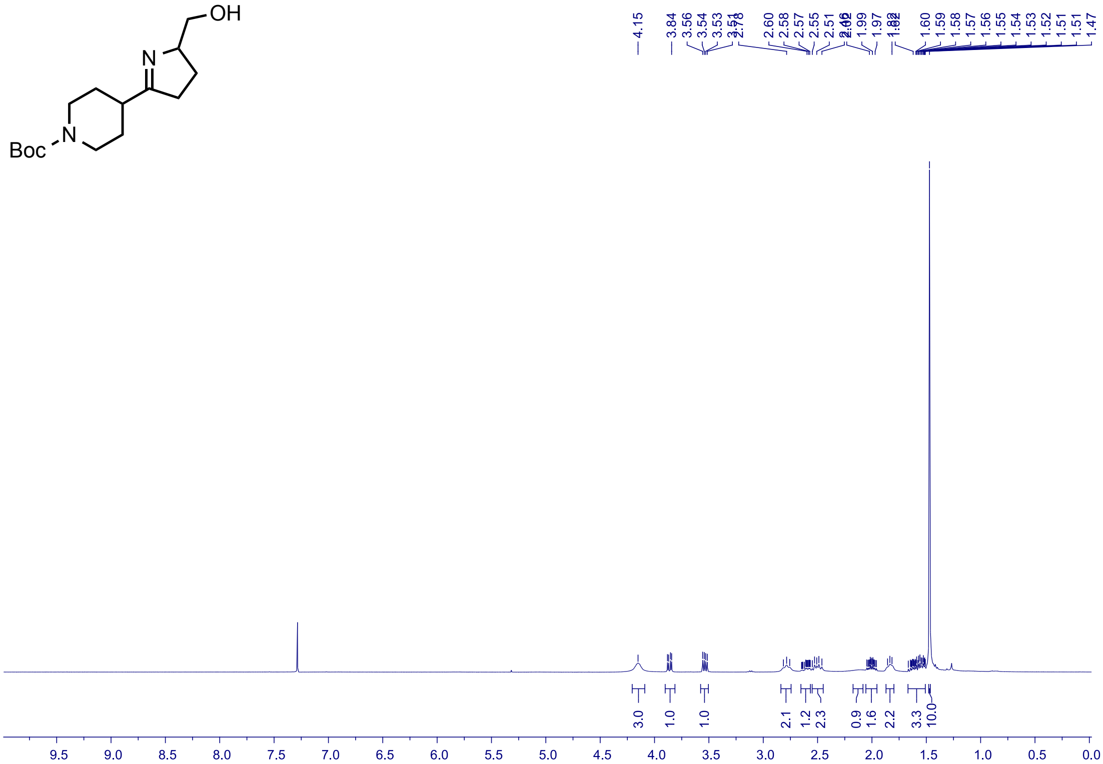

4p: ^{13}C NMR (101 MHz, $CDCl_3$)

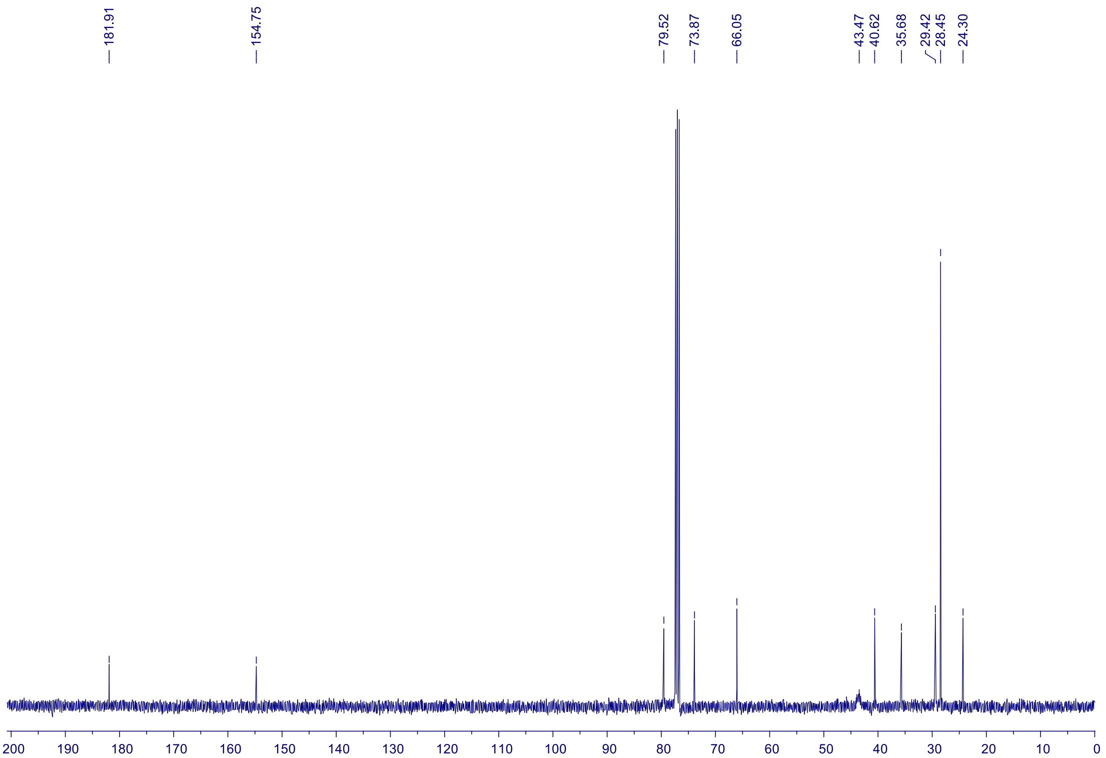

4q: ^{1}H NMR (400 MHz, $CDCl_3$)

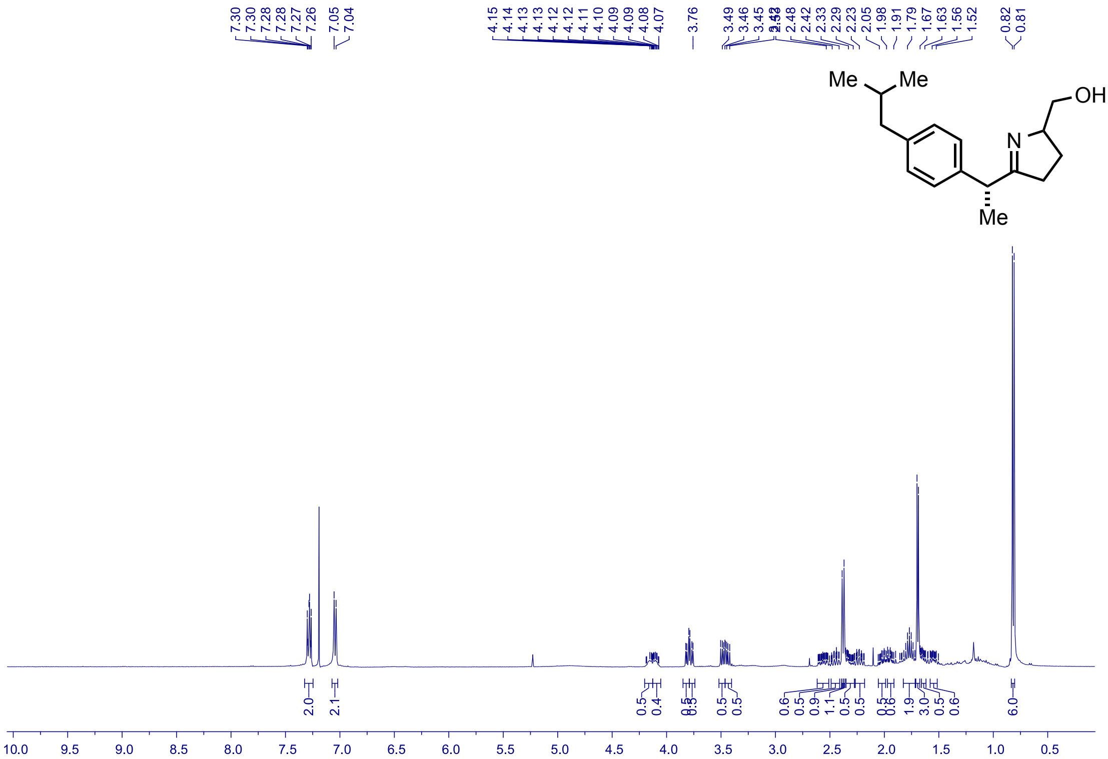

4q: ^{13}C NMR (101 MHz, $CDCl_3$)

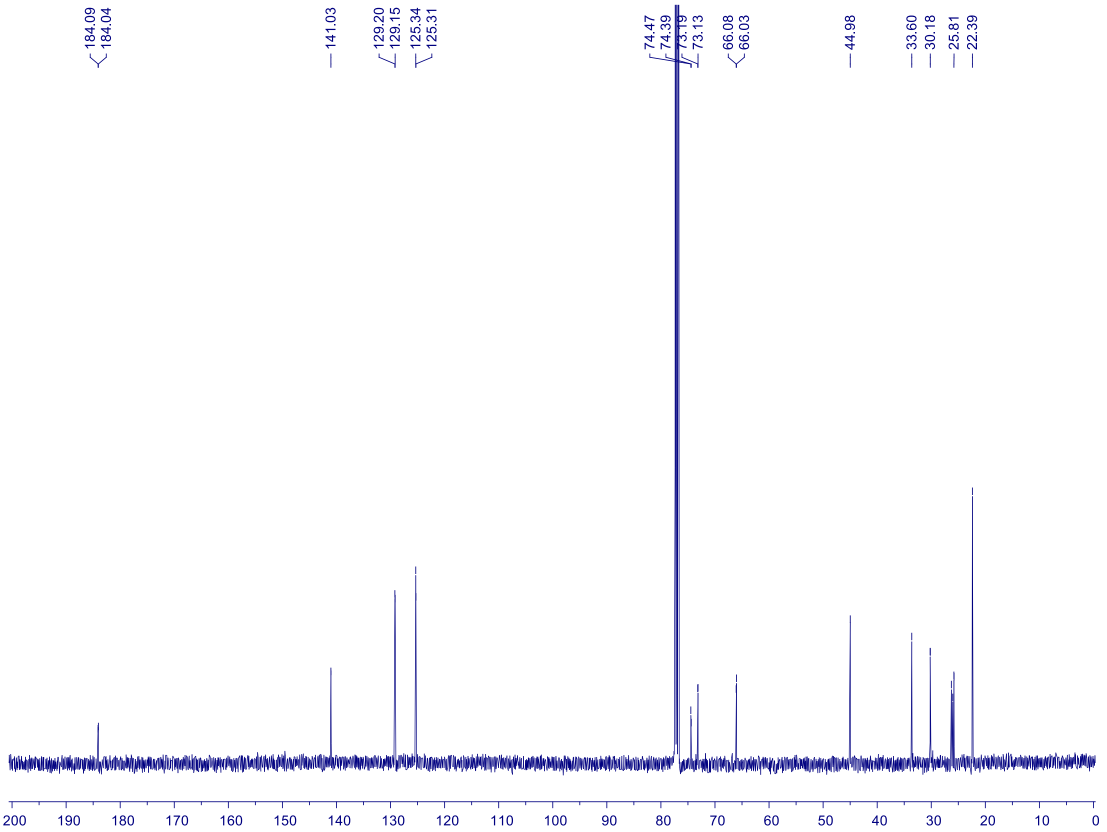

4r: ^{1}H NMR (400 MHz, $CDCl_3$)

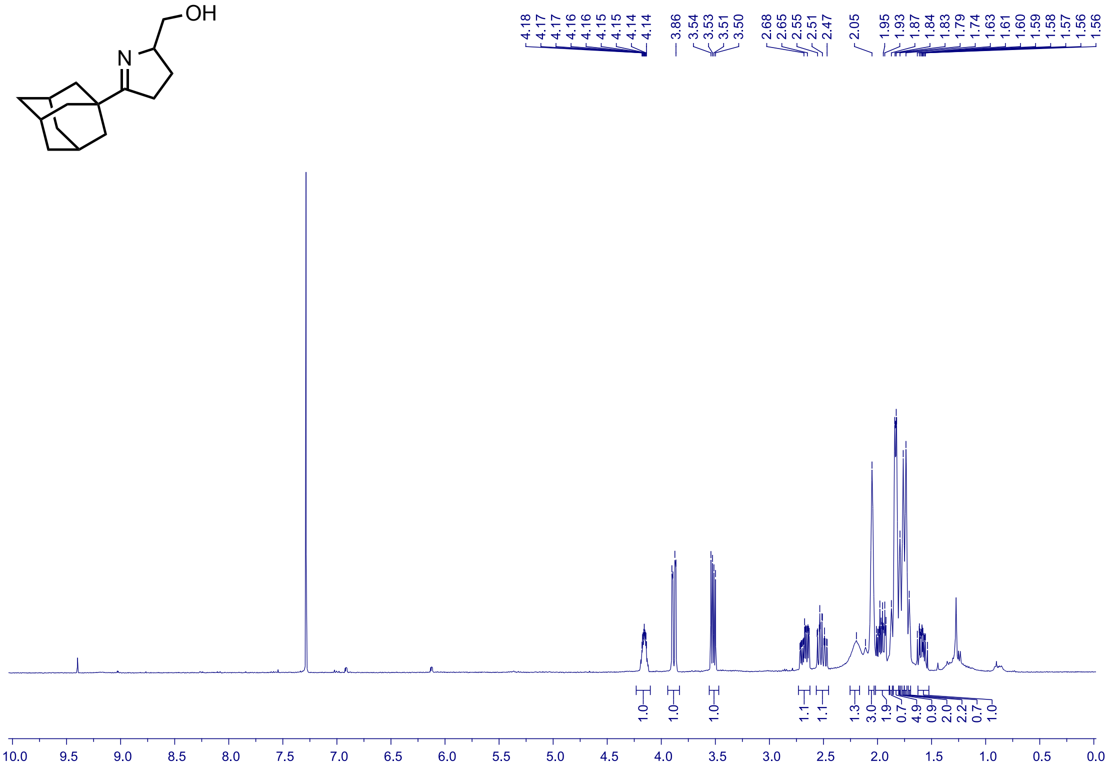

4r: ^{13}C NMR (101 MHz, $CDCl_3$)

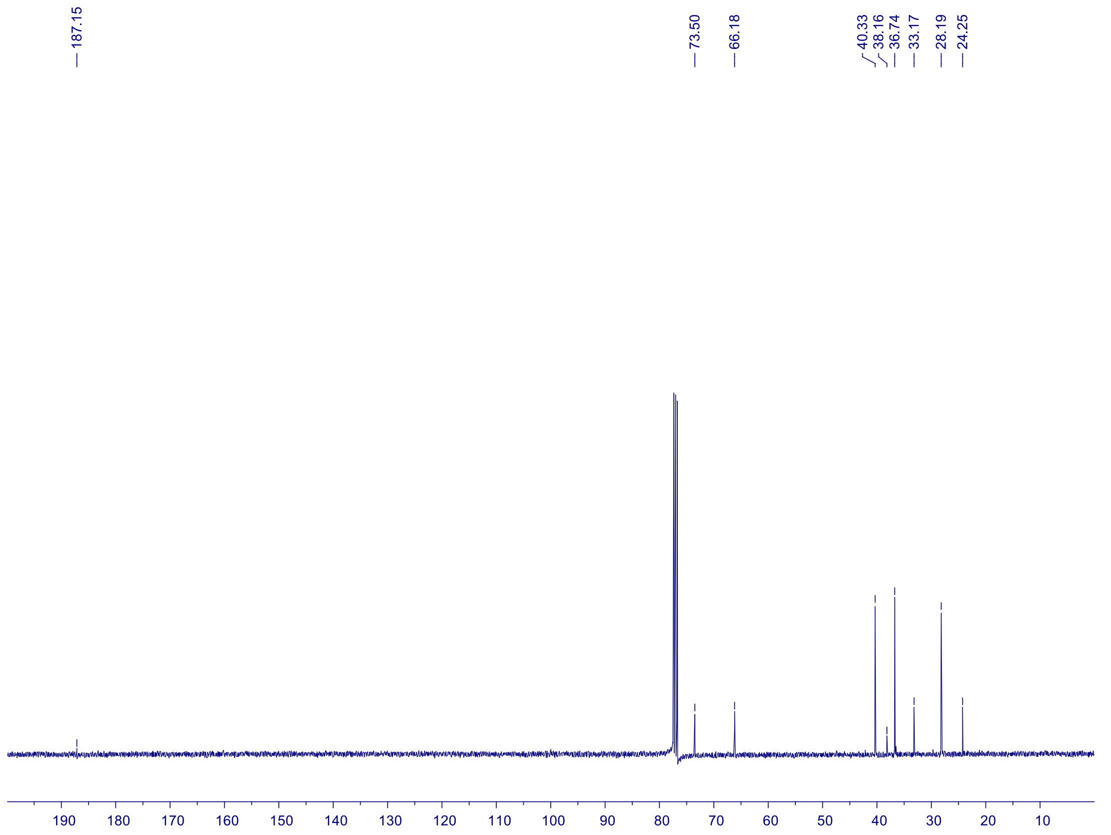

4s: ^{1}H NMR (400 MHz, $CDCl_3$)

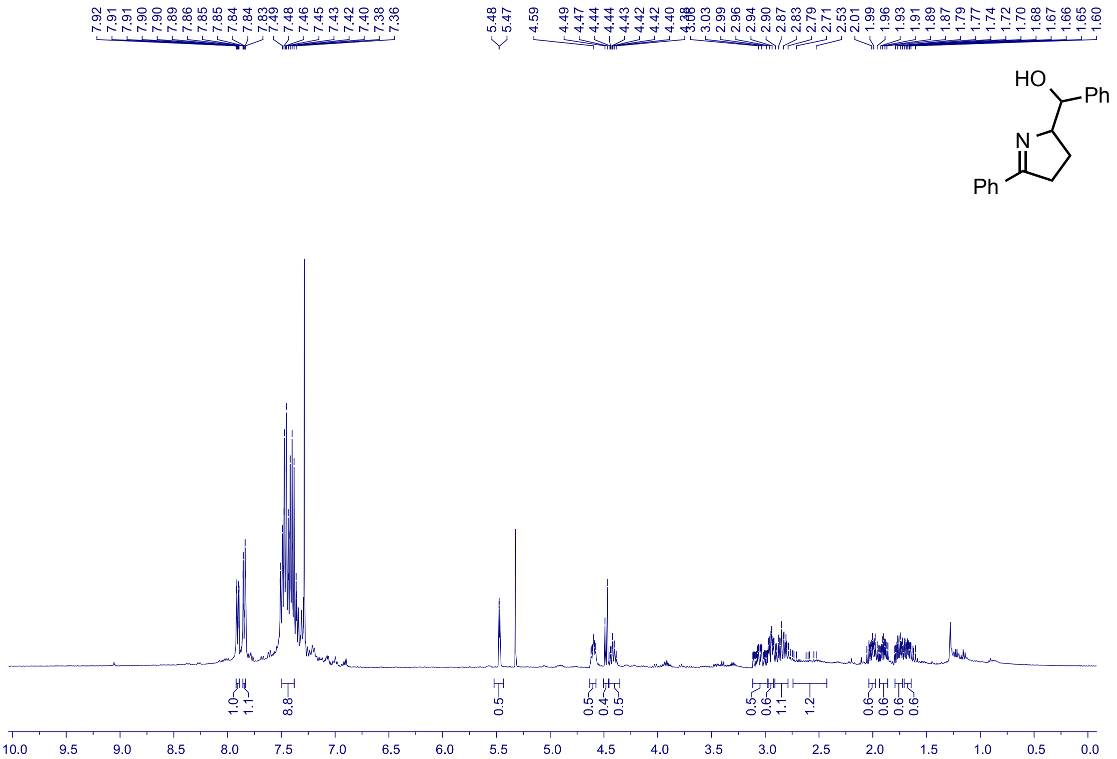

4s: ^{13}C NMR (101 MHz, $CDCl_3$)

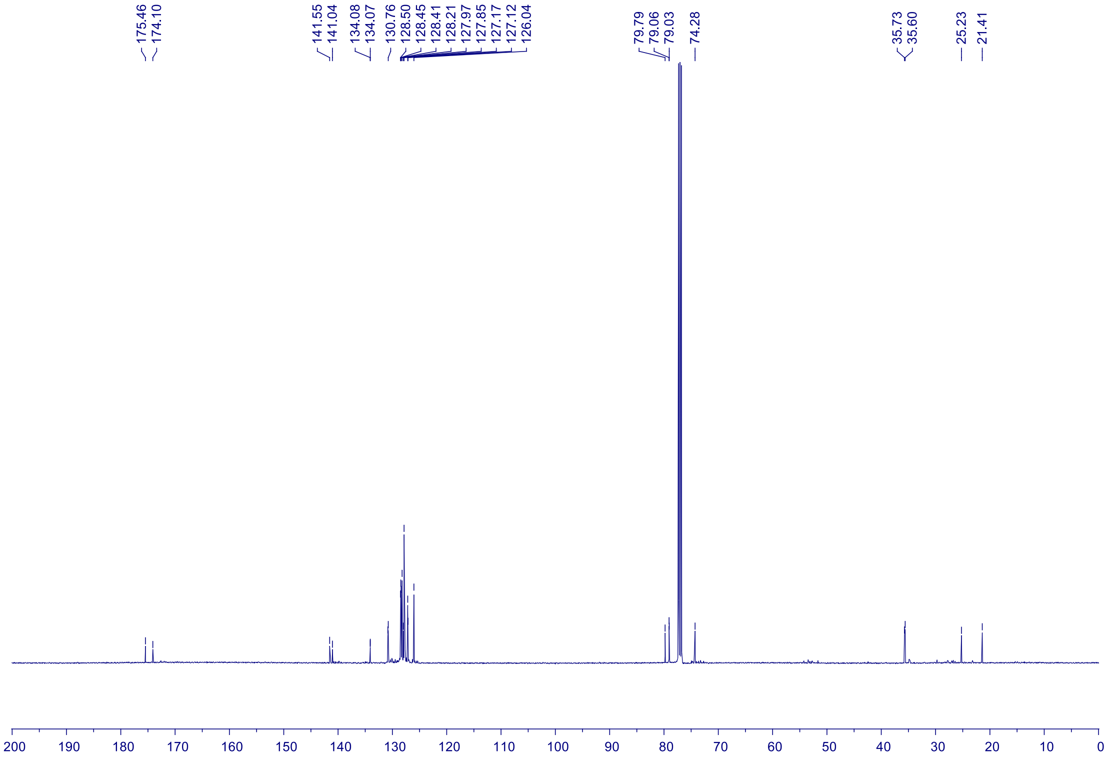

4t: ^{1}H NMR (400 MHz, $CDCl_3$)

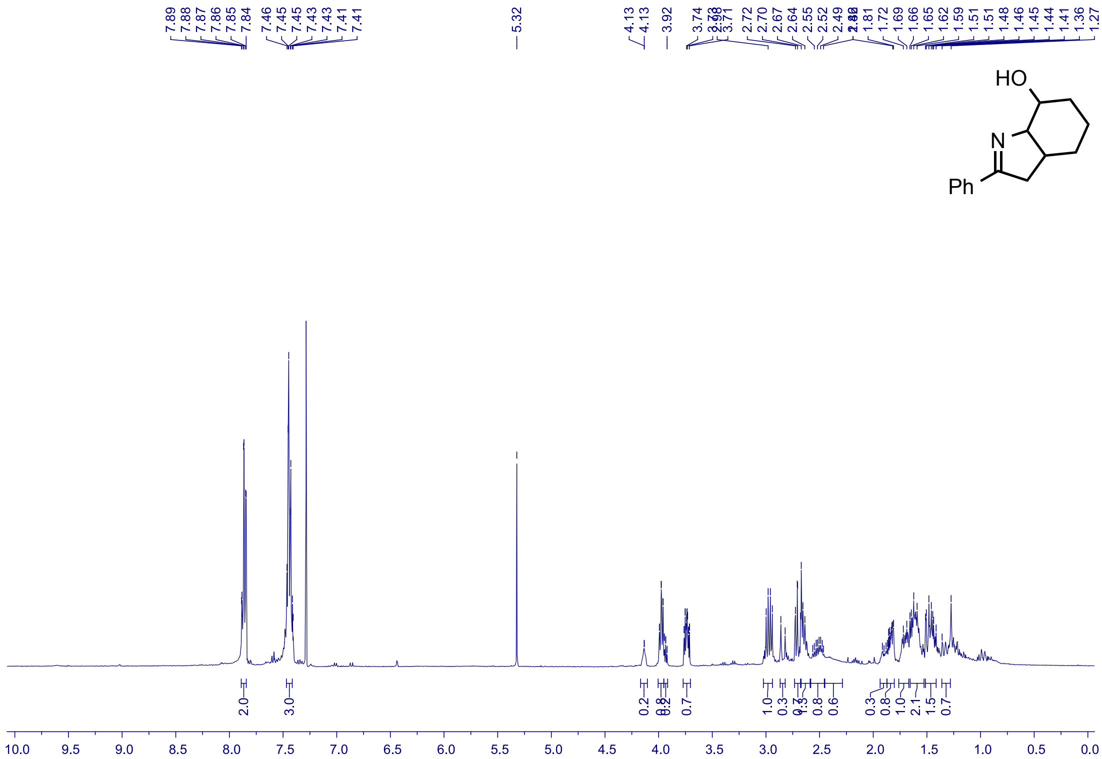

4t: ^{13}C NMR (101 MHz, $CDCl_3$)

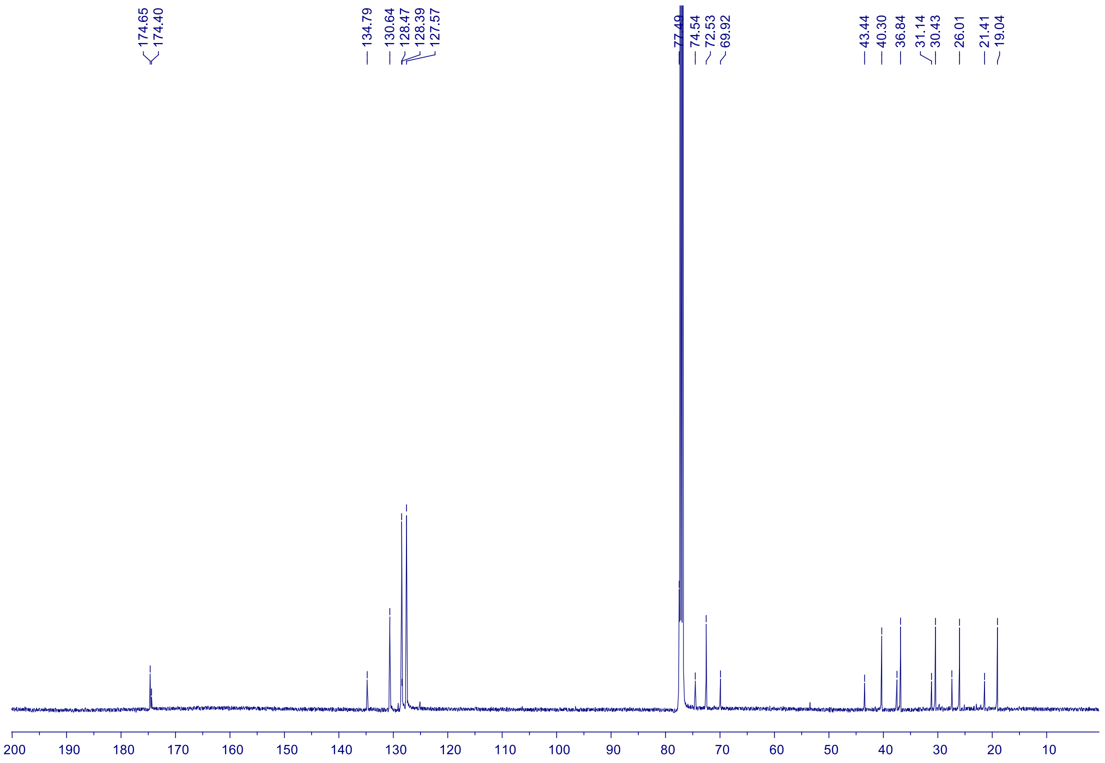

4u: ^{1}H NMR (400 MHz, $CDCl_3$)

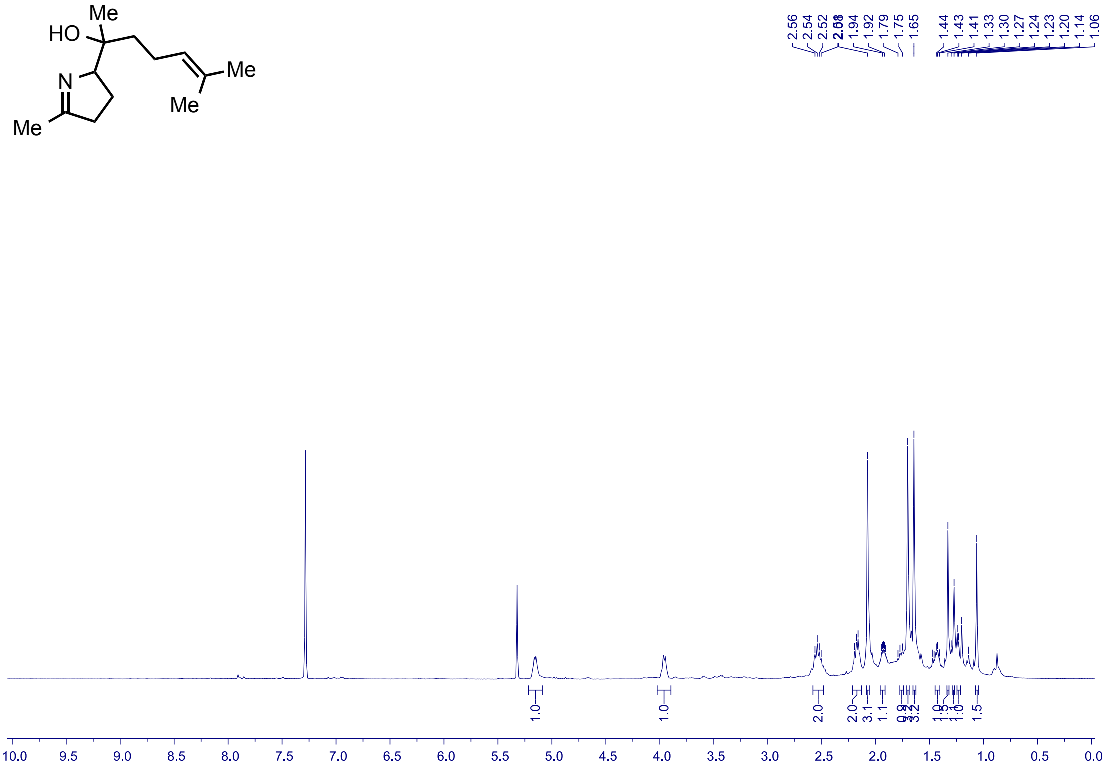

4u: ^{13}C NMR (101 MHz, $CDCl_3$)

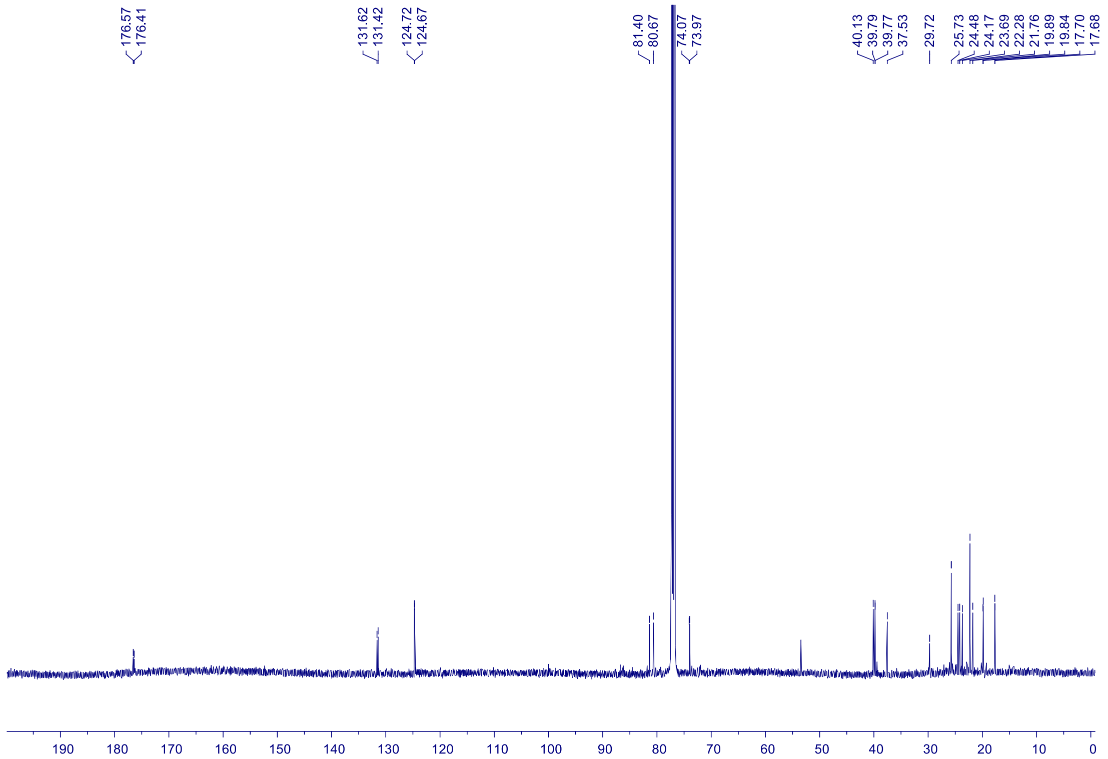

8 X-ray crystal structure

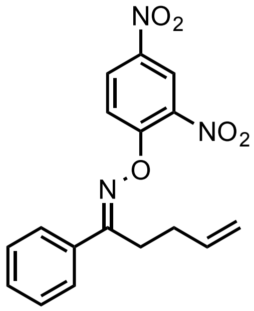

2a

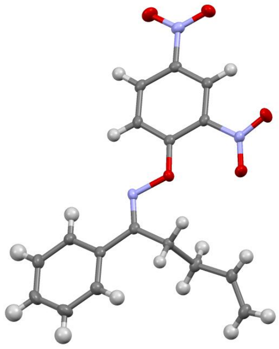

See attached .cif file.

checkCIF/PLATON report

You have not supplied any structure factors. As a result the full set of tests cannot be run.

THIS REPORT IS FOR GUIDANCE ONLY. IF USED AS PART OF A REVIEW PROCEDURE FOR PUBLICATION, IT SHOULD NOT REPLACE THE EXPERTISE OF AN EXPERIENCED CRYSTALLOGRAPHIC REFEREE.

No syntax errors found. CIF dictionary Interpreting this report

Datablock: s4221ma

```
Bond precision:  C-C = 0.0019 A          Wavelength=1.54178

Cell:            a=8.1517(1)     b=15.4941(2)      c=25.5428(3)
                 alpha=90        beta=95.610(1)    gamma=90
Temperature:     100 K

                Calculated                 Reported
Volume          3210.69(7)                 3210.69(7)
Space group     P 21/n                     P2(1)/n
Hall group      -P 2yn                     ?
Moiety formula  C17 H15 N3 O5              ?
Sum formula     C17 H15 N3 O5              C17 H15 N3 O5
Mr              341.32                     341.32
Dx,g cm-3       1.412                      1.412
Z               8                          8
Mu (mm-1)       0.891                      0.891
F000            1424.0                     1424.0
F000'           1428.91
h,k,lmax        10,19,31                   10,19,31
Nref            6318                       6308
Tmin,Tmax       0.772,0.991                0.884,0.991
Tmin'           0.772

Correction method= # Reported T Limits: Tmin=0.884 Tmax=0.991
AbsCorr = MULTI-SCAN

Data completeness= 0.998          Theta(max)= 72.120

R(reflections)= 0.0354( 5188)     wR2(reflections)= 0.0939( 6308)

S = 1.020                 Npar= 451
```

The following ALERTS were generated. Each ALERT has the format
test-name_ALERT_alert-type_alert-level.
Click on the hyperlinks for more details of the test.

Alert level G

```
PLAT005_ALERT_5_G No _iucr_refine_instructions_details  in the CIF     Please Do !
PLAT899_ALERT_4_G SHELXL97   is Deprecated and Succeeded by SHELXL       2014 Note
```

```
   0 ALERT level A = Most likely a serious problem - resolve or explain
   0 ALERT level B = A potentially serious problem, consider carefully
   0 ALERT level C = Check. Ensure it is not caused by an omission or oversight
   2 ALERT level G = General information/check it is not something unexpected

   0 ALERT type 1 CIF construction/syntax error, inconsistent or missing data
   0 ALERT type 2 Indicator that the structure model may be wrong or deficient
   0 ALERT type 3 Indicator that the structure quality may be low
   1 ALERT type 4 Improvement, methodology, query or suggestion
   1 ALERT type 5 Informative message, check
```

It is advisable to attempt to resolve as many as possible of the alerts in all categories. Often the minor alerts point to easily fixed oversights, errors and omissions in your CIF or refinement strategy, so attention to these fine details can be worthwhile. In order to resolve some of the more serious problems it may be necessary to carry out additional measurements or structure refinements. However, the purpose of your study may justify the reported deviations and the more serious of these should normally be commented upon in the discussion or experimental section of a paper or in the "special_details" fields of the CIF. checkCIF was carefully designed to identify outliers and unusual parameters, but every test has its limitations and alerts that are not important in a particular case may appear. Conversely, the absence of alerts does not guarantee there are no aspects of the results needing attention. It is up to the individual to critically assess their own results and, if necessary, seek expert advice.

Publication of your CIF in IUCr journals

A basic structural check has been run on your CIF. These basic checks will be run on all CIFs submitted for publication in IUCr journals (*Acta Crystallographica*, *Journal of Applied Crystallography*, *Journal of Synchrotron Radiation*); however, if you intend to submit to *Acta Crystallographica Section C* or *E*, you should make sure that full publication checks are run on the final version of your CIF prior to submission.

Publication of your CIF in other journals

Please refer to the *Notes for Authors* of the relevant journal for any special instructions relating to CIF submission.

PLATON version of 21/06/2015; check.def file version of 21/06/2015

9 References

1. Salahuddin, S.; renaudet, O.; Reymond, J.-L., *Org. Biomol. Chem.* **2004,** *2*, 1471.
2. Zhu, C.; Li, G.; Ess, D.; Falck, J. R.; Kurti, L., *J. Am. Chem. Soc.* **2012,** *134*, 18253.
3. Gaucher-Wieczorek, F. S.; Mailard, L. T.; Badet, B.; Durand, P., *J. Comb. Chem.* **2010,** *12*, 655.
4. Johnson, S. M.; Petrassi, H. M.; Palaninathan, S. K.; Mohamedmohaideen, N. N.; Purkey, H. E.; Nichols, C.; Chiang, K. P.; Wlakup, T.; Sacchettini, J. C.; Sharpless, K. B.; Kelly, J. W., *J. Med. Chem.* **2005,** *48*, 1576.
5. Jain, A. K.; Kumar, A.; Sarma, K. N., *J. Chem. Soc., Perkin Trans. 2* **1989**, 153.
6. De, P.; Nonappa; Pandurangan, K.; Maitra, U.; Wailes, S., *Org. Lett.* **2007,** *9*, 2767.
7. Takeda, N.; Miyata, O.; Naito, T., *E. J. Org. Chem.* **2007**, 1491.
8. Feng, X.-H.; Zhang, G.-Z.; Chen, C.-Q.; Yang, M.-Y.; Xu, X.-Y.; Huang, G.-S., *Synth. Commun.* **2009,** *39*, 1768.
9. Waser, J.; Gaspar, B.; Nambu, H.; Carreira, E. M., *J. Am. Chem. Soc.* **2006,** *128*, 11693.
10. Hok, S.; Schore, N. E., *J. Org. Chem.* **2006,** *71*, 1736.
11. Gomez-Suarez, A.; Gasperini, D.; Vummaleti, S. V. C.; Poater, A.; Cavallo, L.; Nolan, S. P., *ACS Catal.* **2014,** *4*, 2701.
12. Martinelli, J. R.; Watson, D. A.; Freckmann, D. M. M.; Barder, T. E.; Buchwald, S. L., *J. Org. Chem.* **2008,** *73*, 7102.
13. Murphy, J. A.; Commerureuc, A. G. J.; Snaddon, T. N.; McGuire, T. M.; Khan, T. A.; Hilser, K.; Dewis, M. L.; Carling, R., *Org. Lett.* **2005,** *7*, 1427.
14. Krishnamoorthy, R.; Lam, S. Q.; Manley, C. M.; Herr, R. J., *J. Org. Chem.* **2010,** *75*, 1251.
15. Niu, T.; Zhang, W.; Huang, D.; Xu, C.; Wang, H.; Hu, Y., *Org. Lett.* **2009,** *11*, 4474.
16. Davi, M.; Lebel, H., *Chem. Commun.* **2008**, 4974.
17. Wael, F. D.; Muccioli, G. G.; Lambert, D. M.; Sergent, T.; Schneider, Y.-J.; Rees, J.-F.; Marchand-Brynaert, J., *E. J. Med. Chem.* **2010,** *45*, 3564.
18. Portela-Cubillo, F.; Surgenor, B. A.; Aitken, R. A.; Walton, J. C., *J. Org. Chem.* **2008,** *73*, 8124.
19. Avilov, D. V.; Malusare, M. G.; Arslancan, E.; Dittmer§, D. C., *Org. Lett.* **2004,** *6*, 2225.
20. Pirtsch, M.; Paria, S.; Matsuno, T.; Isobe, H.; Reiser, O., *Chem. Eur. J.* **2012,** *18*, 7336.
21. Clive, D. L. J.; Pham, M. P., *J. Org. Chem.* **2009,** *74*, 1685.
22. Trost, B. M.; Lehr, K.; Michaelis, D. J.; Xu, J.; Buckl, A. K., *J. Am. Chem. Soc.* **2010,** *132*, 8915.
23. Hansford, K. A.; Dettwiler, J. E.; Lubell, W. D., *Org. Lett.* **2003,** *5*, 4887.
24. Patil, N. T.; Pahadi, N. K.; Yamamoto, Y., *Synthesis* **2004,** *13*, 2186.
25. Fox, D. J.; Pedersen, D. S.; Warren, S., *Chem. Commun.* **2004**, 2598.
26. Yang, C.-F.; Wang, J.-Y.; Tian, S.-K., *Chem. Commun.* **2011,** *47*, 8343.
27. Boev, V. I.; Moskalenko, A. I.; Belopukhov, S. L.; Przheval'skii, *Russ. J. Org. Chem.* **2015,** *51*, 493.

28. Cai, Y.; Jalan, A.; Kubosumi, A. R.; Castle, S. L., *Org. Lett.* **2015,** *17*, 488.
29. Uchiyama, K.; Hayashi, Y.; Narasaka, K., *Tetrahedron* **1999,** *55*, 8915.
30. Tsierkezos, N. G., *J. Solution Chem.* **2007,** *36*, 289.
31. Bard, A. J.; Faulker, L. R., *Electrochemical Methods: Fundamentals and Applications, 2nd Edition* **2001**, Wiley, New York.
32. Cismenia, M. A.; Yoon, T. P., *Chem. Sci.* **2015**, doi:10.1039/C5SC02185E.
33. Wahba, M. E. K.; El-Enany, N.; Belal, F., *Anal. Methods* **2014**, DOI: 10.1039/C3AY42093K.
34. Wang, D.-S.; Ye, Z.-S.; Chen, Q.-A.; Zhou, Y.-G.; Yu, C.-B.; Fan, H.-J.; Duan, Y., *J. Am. Chem. Soc.* **2011,** *133*, 8866.
35. O'Reilly, E.; Iglesias, C.; Ghisleri, D.; Hopwood, J.; Galman, J. L.; Lloyd, R. C.; Turner, N. J., *Angew. Chem. Int. Ed.* **2014,** *53*, 2447.
36. Kitamura, M.; Yoshida, M.; Kikuchi, T.; Narasaka, K., *Synthesis* **2003**, 2415.
37. Boivin, J. B.; Fouquet, E.; Zard, S. Z., *Tetrahedron* **1994,** *50*, 1745.
38. Portela-Cubillo, F.; Scott, J. S.; Walton, J. C., *J. Org. Chem.* **2008,** *73*, 5558.
39. Hirose, K., *J. Incl. Phenom. Mecro.* **2001,** *39*, 193.
40. Kitamura, M.; Shintaku, Y.; Kudo, D.; Okauchi, T., *Tetrahedron Lett.* **2010,** *51*, 4890.
41. , Gaussian 09, Revision B.01: M. J. Frisch, G. W. Trucks, H. B. Schlegel, G. E. Scuseria, M. A. Robb, J. R. Cheeseman, G. Scalmani, V. Barone, B. Mennucci, G. A. Petersson, H. Nakatsuji, M. Caricato, X. Li, H. P. Hratchian, A. F. Izmaylov, J. Bloino, G. Zheng, J. L. Sonnenberg, M. Hada, M. Ehara, K. Toyota, R. Fukuda, J. Hasegawa, M. Ishida, T. Nakajima, Y. Honda, O. Kitao, H. Nakai, T. Vreven, J. A. Montgomery, Jr., J. E. Peralta, F. Ogliaro, M. Bearpark, J. J. Heyd, E. Brothers, K. N. Kudin, V. N. Staroverov, T. Keith, R. Kobayashi, J. Normand, K. Raghavachari, A. Rendell, J. C. Burant, S. S. Iyengar, J. Tomasi, M. Cossi, N. Rega, J. M. Millam, M. Klene, J. E. Knox, J. B. Cross, V. Bakken, C. Adamo, J. Jaramillo, R. Gomperts, R. E. Stratmann, O. Yazyev, A. J. Austin, R. Cammi, C. Pomelli, J. W. Ochterski, R. L. Martin, K. Morokuma, V. G. Zakrzewski, G. A. Voth, P. Salvador, J. J. Dannenberg, S. Dapprich, A. D. Daniels, O. Farkas, J. B. Foresman, J. V. Ortiz, J. Cioslowski, and D. J. Fox, Gaussian, Inc., Wallingford CT, 2010.
42. (a) Becke, A. D., *J. Chem. Phys.* **1993,** *98*, 5648; (b) Stephens, P. J.; Devlin, F. J.; Chabalowski, C. F.; Frish, M. J., *J. Chem. Phys.* **1994,** *98*, 11623.
43. Grimme, S., *J. Comput. Chem.* **2006,** *27*, 1787.
44. Marenich, A. V.; Cramer, C. J.; Truhlar, D. G., *J. Phys. Chem. B.* **2009,** *113*, 6378.
45. Yanai, T.; Tew, D.; Handy, N., *Chem. Phys. Lett.* **2004,** *393*, 51.
